# Exploring Novel Therapeutic Targets in Breast Cancer via Comprehensive Omics Profiling and Experimental Verification

**DOI:** 10.3390/biology14040405

**Published:** 2025-04-11

**Authors:** Shengjun Chai, Jiayong Cui, Yinuo Sun, Xiaowu Wang, Chunmei Cai

**Affiliations:** 1 Research Center for High Altitude Medicine, Qinghai University Medical College, Xining 810008, China; ys221002100819@qhu.edu.cn (S.C.); ys221002100823@qhu.edu.cn (J.C.); 19902747732@163.com (Y.S.); 2 Key Laboratory of the Ministry of High Altitude Medicine, Qinghai University Medical College, Xining 810008, China; 3 Key Laboratory of Applied Fundamentals of High Altitude Medicine (Qinghai-Utah Joint Key Laboratory of Plateau Medicine), Qinghai University Medical College, Xining 810008, China; 4 Laboratory for High Altitude Medicine of Qinghai Province, Qinghai University Medical College, Xining 810008, China; 5 Department of Surgical Oncology, The Affiliated Hospital of Qinghai University, Xining 810001, China; 6 Department of Economics, School of Finance and Economics, Qinghai University, Xining 810016, China

**Keywords:** Mendelian randomization, breast cancer, ATOH8, DNASE2, experimental validation

## Abstract

Background: Breast cancer is the leading cause of cancer-related deaths among women worldwide. Deciphering the molecular mechanisms of breast cancer is crucial for developing targeted therapeutic approaches. Methods: This study analyzed gene expression profiles from the Gene Expression Omnibus (GEO) database to identify differentially expressed genes (DEGs) in breast cancer. Mendelian randomization (MR) analysis was then employed using publicly available eQTL databases to evaluate potential causal relationships between these DEGs and breast cancer. Enrichment analyses were further conducted to explore their functional significance. Furthermore, external validation of co-expressed genes was conducted using The Cancer Genome Atlas (TCGA) database. In vitro functional assays and drug sensitivity analyses were performed on selected target genes to validate their roles in breast cancer pathogenesis and treatment. Results: A total of 1052 upregulated and 1380 downregulated genes were identified in breast cancer. Additionally, MR analysis revealed 12 significant co-expressed genes potentially contributing to breast cancer pathogenesis. These genes were primarily enriched in lipid metabolism and immune responses via regulating microRNA functions and AMPK signaling. Validation through the TCGA database confirmed differential expression of these genes in breast cancer tissues. Strikingly, functional assays of the less-reported genes DNASE2 and ATOH8 demonstrated their involvement in breast cancer pathogenesis through modulating proliferation, migration, and invasion of cancer cells. Notably, several commonly used clinical drugs for breast cancer management, such as 5-Fluorouracil, exhibited dramatically increased sensitivity to DNASE2 and ATOH8 expression. Conclusions: Our study provides novel insights into the molecular basis of breast cancer pathogenesis and identifies promising therapeutic strategies for this condition.

## Simple Summary

Breast cancer remains a leading cause of cancer-related deaths in women globally, with current therapies often limited by resistance and lack of broad applicability. This study aimed to identify novel molecular targets for breast cancer treatment by integrating multi-omics data and experimental validation. Using gene expression datasets from public databases, we identified 1052 upregulated and 1380 downregulated genes in breast cancer. Through Mendelian randomization analysis, 12 key genes were found to be causally linked to breast cancer pathogenesis, including DNASE2 and ATOH8, which were further validated in external datasets. Functional experiments revealed that ATOH8 suppresses breast cancer cell proliferation, migration, and invasion, while DNASE2 promotes these processes. Drug sensitivity analysis highlighted that drugs like 5-Fluorouracil show enhanced efficacy in patients with high DNASE2 or ATOH8 expression. These findings uncover new therapeutic targets and provide insights into personalized treatment strategies for breast cancer.

## 1. Introduction

Breast cancer is a complex and heterogeneous disease, ranking among the most prevalent cancer types in women worldwide [1,2,3]. The primary characteristic of this disease is the uncontrolled growth of cells of mammary epithelial tissue, which not only leads to tumor formation but also enables these tumors to spread to other parts of the body, resulting in metastasis [4,5]. Numerous molecular and cellular mechanisms, closely associated with the development of breast cancer, have gradually been identified, including exosomes [6,7,8]. Due to either the scarcity or suboptimal efficacy of targeted therapies directed towards these mechanisms, it is regrettable that the mortality rate of breast cancer remains high. Therefore, elucidating novel pathogenic mechanisms and developing targeted therapeutic approaches for breast cancer has become an urgent issue.

Molecular targeted therapy represents a promising advancement in breast cancer management [9,10,11,12]. Unlike traditional chemotherapeutic drugs that typically target rapidly dividing cancerous and normal cells, leading to severe side effects, molecular targeted drugs are designed to selectively recognize the unique markers of cancer cells [13,14]. This specificity enables precise eradication of cancer cells, substantially reducing damage to healthy cells [15]. Currently, several molecular markers specific to breast cancer, such as HER2, estrogen receptor (ER), and progesterone receptor (PR), have been identified [16,17,18,19]. Targeted drugs for these markers, such as Herceptin (for HER2-positive breast cancer) and aromatase inhibitors (for ER-positive breast cancer), have proven effective in clinical settings [20,21,22]. This treatment approach, by accurately identifying and targeting specific molecular markers, can directly intervene in the growth and survival pathways of breast cancer cells, thereby enhancing the efficacy and selectivity of the treatment. It is important to note that while targeted therapies can enhance the precision and efficacy of breast cancer management, they are ineffective against tumors lacking specific markers. Approximately 15% to 20% of breast cancer cases are classified as triple-negative breast cancer (TNBC), which lacks estrogen receptor (ER), progesterone receptor (PR), and HER2 expression [23]. Additionally, around 15% of TNBC patients harbor mutations in the BRCA1 or BRCA2 genes [24]. These cases are resistant to current targeted therapies, thus limiting the broad applicability of these treatments. Therefore, identifying additional tumor-specific biomarkers is crucial for developing novel and effective therapeutic strategies to improve the prognosis of breast cancer patients.

In this study, we analyzed microarray datasets, obtained from the Gene Expression Omnibus (GEO) database, to identify differentially expressed genes (DEGs) between breast cancer and normal tissue samples. Mendelian randomization (MR) analysis was then employed on expression quantitative trait loci (eQTLs) to evaluate the correlation and causality between these DEGs and breast cancer pathogenesis. Additionally, Gene Ontology (GO) and Kyoto Encyclopedia of Genes and Genomes (KEGG) enrichment analyses were utilized to explore potential functional pathways and mechanisms associated with these genetic variations. Furthermore, the Protein–Protein Interaction (PPI) network analysis was performed to identify the five most significantly associated genes, and ROC curves and a nomogram were subsequently constructed to calculate disease risk scores. Finally, we conducted external validation using The Cancer Genome Atlas (TCGA) database and further performed functional assays and drug sensitivity analyses on ATOH8 and DNASE2, additional tumor-specific markers identified by MR analysis. Through these methods, this study identified 12 significant co-expressed genes that potentially contributed to breast cancer pathogenesis. These genes were primarily enriched in lipid metabolism and immune responses via regulating microRNA functions and AMPK signaling. The functional assays proved the contribution of DNASE2 and ATOH8 in the progression of breast cancer by modulating the proliferation, migration, and invasion of cancer cells. Drug sensitivity analysis indicated significant correlations between several commonly used clinical drugs for breast cancer treatment and DNASE2/ATOH8 expression. These findings uncover a novel molecular basis for breast cancer and support the development of innovative therapeutic approaches.

## 2. Materials and Methods

### 2.1. Data Collection

Gene expression profiles and clinical phenotype information pertaining to “breast cancer”, “gene expression”, and “Homo sapiens” were retrieved through the analysis of microarray datasets. The corresponding gene expression data, along with platform probe annotations, are available for download from the GEO database (https://www.ncbi.nlm.nih.gov/geo/) (accessed on 1 July 2024). The selection criteria for breast cancer-related datasets include: (i) a minimum of six samples, with at least three cases and three controls; (ii) samples that have not been subjected to chemical treatment or genetic modification; and (iii) the accessibility of raw data or microarray-based gene expression profiles in the GEO database.

### 2.2. Detection of DEGs

The datasets GSE109169, GSE113865, GSE139038, and GSE205185 were processed and preprocessed using R software (version 4.2.2), with individual dataset corrections applied. The merged dataset, consisting of 57 normal and 86 breast cancer samples, was subjected to batch correction and differential expression analysis. DEGs were identified using the classical Bayesian approach of the “limma” package, setting significance thresholds at *p* < 0.05 and logFoldChange (LogFC) > 0.585. Heatmaps and volcano plots of DEGs were generated using the “pheatmap” package. Data normalization and standardization were conducted based on gene expression matrices and annotation files obtained from the GEO database. Principal Component Analysis (PCA) was performed using the “prcomp” function to mitigate batch effects and enhance visualization, thereby aiding further identification and validation of key genes that distinguish breast cancer samples from healthy controls.

### 2.3. eQTL Analysis of Exposure Data

Westra et al. conducted the most extensive meta-analysis of eQTL data, including peripheral blood eQTL data from 5311 European individuals [25]. The eQTL data used in this study were obtained from the GWAS Catalog (https://gwas.mrcieu.ac.uk/) (accessed on 1 July 2024). Using the R package “TwoSampleMR”, we selected strongly associated single nucleotide polymorphisms (SNPs) with *p*-values < 5 × 10^−8^ as instrumental variables. Linkage disequilibrium was assessed with an r^2^ threshold of <0.001, and clumping was performed with a distance of 10,000 kb. SNPs showing weak associations or insufficient explanatory power for the phenotypic variation, defined by an F-test value > 10, were excluded.

### 2.4. Ascertainment of Outcome Data

The outcome data were obtained from the GWAS Catalog (https://gwas.mrcieu.ac.uk/) (accessed on 1 July 2024), specifically from the GWAS summary datasets (IEU). The specific GWAS ID utilized in this study was ebi-a-GCST90018799, which includes data from 17,389 cases and 240,341 controls of European ancestry, encompassing 24,133,589 SNPs. All GWAS summary statistics cited in this study are publicly accessible and available for download. Additionally, ethical approval for this study was based on the original analyses conducted.

### 2.5. MR Analysis

The “TwoSampleMR” package (0.6.1) was used for Mendelian randomization (MR) analysis, employing the Inverse Variance Weighted (IVW) method to explore the causal relationship between specific genes and breast cancer. Additional methods, including MR-Egger, simple mode, weighted median, and weighted mode, were applied to assess causal effects [26,27]. The identification of disease-associated genes followed a three-step approach: (i) Initially, genes with *p*-values < 0.05 in the IVW analysis were considered; (ii) Genes were further refined by evaluating the consistency of odds ratios across at least three MR methods; and (iii) Genes exhibiting pleiotropy with *p*-values < 0.05 were excluded.

Throughout this process, cross-analysis identified co-expressed genes between disease-associated genes and DEGs, including both upregulated and downregulated genes. Subsequently, MR analysis was conducted on all intersecting genes to determine their causal relationships with the disease. The analysis also included tests for heterogeneity, pleiotropy, and sensitivity to missing data, ensuring the robustness of the results. To visualize and substantiate the findings, scatter plots, forest plots, and funnel plots were created and examined.

### 2.6. GO/KEGG Enrichment Analysis

The “clusterProfiler” R package, with a filtering criterion of a *p*-value of less than 0.05, was employed for functional annotation of co-expressed genes using GO terms and KEGG pathways.

### 2.7. Immune Cell Analysis

CIBERSORT was utilized to analyze the infiltration levels of 22 types of immune cells in breast cancer and to examine the correlation between co-expressed genes in breast cancer and the infiltration of these immune cells.

### 2.8. Differential Analysis of the TCGA Database

R software (version 4.2.2) was utilized to verify the differences in co-expressed genes between the tumor and normal tissue samples of patients with breast cancer, retrieved from the TCGA-BRCA database, which was used as an external validation cohort. These findings from the TCGA-BRCA database were then compared with the MR analysis results.

### 2.9. Establishing Protein–Protein Interaction Networks and Nomogram Models

In this study, we constructed protein–protein interaction networks (PPI) to deepen our understanding of intracellular protein interactions. These networks were built based on the STRING database (https://cn.string-db.org/) (accessed on 1 July 2024), with the interaction confidence threshold set at 0.15 and other parameters left at default. Visualization of the networks was accomplished using Cytoscape software (version 3.10.2). Additionally, we used the GeneMANIA platform (https://GeneMANIA.org/) (accessed on 1 July 2024) to further analyze the PPI networks. To assess the risk of breast cancer, we constructed a nomogram model using the “rms” software package (6.8.1). Moreover, we utilized the “ROC” software package (1.18.5) to draw ROC curves to evaluate the diagnostic effectiveness of the proposed biomarkers.

### 2.10. Gene Set Enrichment Analysis (GSEA) Enrichment Analysis

The GSEA enrichment analysis was employed to further explore which relevant functions or pathways were enriched with varying expression levels of a particular gene. In GSEA, a *p*-value of less than 0.05 was considered statistically significant.

### 2.11. Cells and Culture Conditions

The human breast cancer cell lines MCF-7 and MDA-MB-231 were obtained from Servicebio (Wuhan, China). All breast cancer cell lines were cultured in Dulbecco’s modified Eagle’s medium (DMEM) from Servicebio, supplemented with 10% fetal bovine serum (FBS) from VivaCell (Shanghai, China) and 1% penicillin/streptomycin from Boster (Wuhan, China), at 37 °C with 5% CO_2_.

### 2.12. Cell Transfection

Transfection experiments were conducted on MCF-7 and MDA-MB-231 cells in the exponential growth phase. Before transfection, 1 × 10^6^ breast cancer cells were cultured in each well of a 6-well plate containing 2 mL of complete medium for 24 h to achieve 60–70% confluence. Small interfering RNAs (siRNAs) targeting ATOH8 (Si-ATOH8) and DNase2 (Si-Dnase2), along with their respective controls, were purchased from Sangon Biotech (Shanghai, China). Following the manufacturer’s instructions, the cells were transfected with Lipofectamine RNAiMAX reagent (Invitrogen, Carlsbad, CA, USA). The sequences of the siRNAs used were as follows:

*ATOH8 siRNA*
5′-GGUGCCGUGCUACUCAUAUTT-3′
*DNase2 siRNA*
5′-CAAGAACCCUGGAACAGCAGCAUCA-3′

### 2.13. Quantitative Real-Time Polymerase Chain Reaction (qRT-PCR)

Total RNA was extracted from cultured cell lines using Trizol reagent (Invitrogen, Carlsbad, CA, USA), and complementary cDNA was synthesized using FastKing gDNA Dispelling RT SuperMix (TIANGEN, Beijing, China). qRT-PCR was performed with QuantiNova SYBR PCR Mix Kit (QIAGEN, Hilden, Germany). Expression data were normalized to the endogenous reference gene Tubulin to control for variability in expression levels. Relative quantification calculations were performed using 2^−ΔCT^. The sequences of the primers used were as follows:
*ATOH8* forward: 5′-CAGGTGCCGTGCTACTCATA-3′;*ATOH8* reverse: 5′-AGTCACTCCTTGCGCTTCTT-3′;*DNase2* forward: 5′-TCGCCTTCCTGCTCTACAAT-3′;*DNase2* reverse: 5′-CCCATCTTCGAGAACTGAGC-3′;*β-Tubulin* forward: 5′-CTCTGAAGCTGACCACACCA-3′;*β-Tubulin* reverse: 5′-GCCAGGCATAAAGAAATGGA-3′.

### 2.14. Cell Proliferation Assay

Cell proliferation was evaluated using the Cell Titer 96 Aqueous One Solution Cell Proliferation Assay kit (Promega, Madison, WI, USA). Transfected cells were seeded in a 96-well plate at a density of 4 × 10^3^ cells per well in a volume of 100 μL. Subsequently, 20 μL of MTS solution was added to each well, and the cells were incubated at a set of time points: 0, 24, 48, and 72 h. The absorbance was measured at 490 nm using a microplate spectrophotometer (Tecan, Zürich, Switzerland).

### 2.15. Cell Wound Healing Assay

A total of 1 × 10^5^ cells per well were plated in a 6-well plate using the complete medium. Upon reaching full confluence, wounds were created through the monolayers by using a sterile pipette tip. The cells were then rinsed multiple times with phosphate-buffered saline to clear away debris and cultured in a medium containing 2% FBS. Healing was observed via microscopy (ZEISS, Oberkochen, Germany) and quantified using ImageJ software (1.54d).

### 2.16. Transwell Migration and Invasion Assays

Cells were initially treated with trypsin and then suspended in a serum-free medium. Subsequently, they were added to the upper chamber (#3422, Corning, NY, USA), either with or without Matrigel (#356234, Corning, NY, USA), which was diluted at a 1:6 ratio, for migration and invasion assays, respectively. The lower chamber was supplemented with a complete medium containing 20% FBS. After 48 h of incubation, the cells were fixed with methanol and subsequently stained with crystal violet. Finally, cells were photographed via microscopy (ZEISS, Oberkochen, Germany) and quantified using ImageJ software.

### 2.17. Analysis of Drug Sensitivity in Risk Subtypes

The “pRRophetic” R package (0.5) was used to assess drug sensitivity differences between high and low gene expression groups based on breast cancer RNA-sequencing (RNA-seq) data obtained from the Genomics of Drug Sensitivity in Cancer (GDSC) database. Drug sensitivity was quantified by the half-maximal inhibitory concentration (IC50) values.

### 2.18. Statistical Analysis

Data analysis was carried out using GraphPad Prism 9.0 (GraphPad Software). The results are expressed as the mean ± standard deviation (SD). Group differences were evaluated using Student’s *t*-test, with statistical significance defined as *p* < 0.05.

## 3. Results

### 3.1. Overview of the Four GEO Datasets

**Figure 1:**
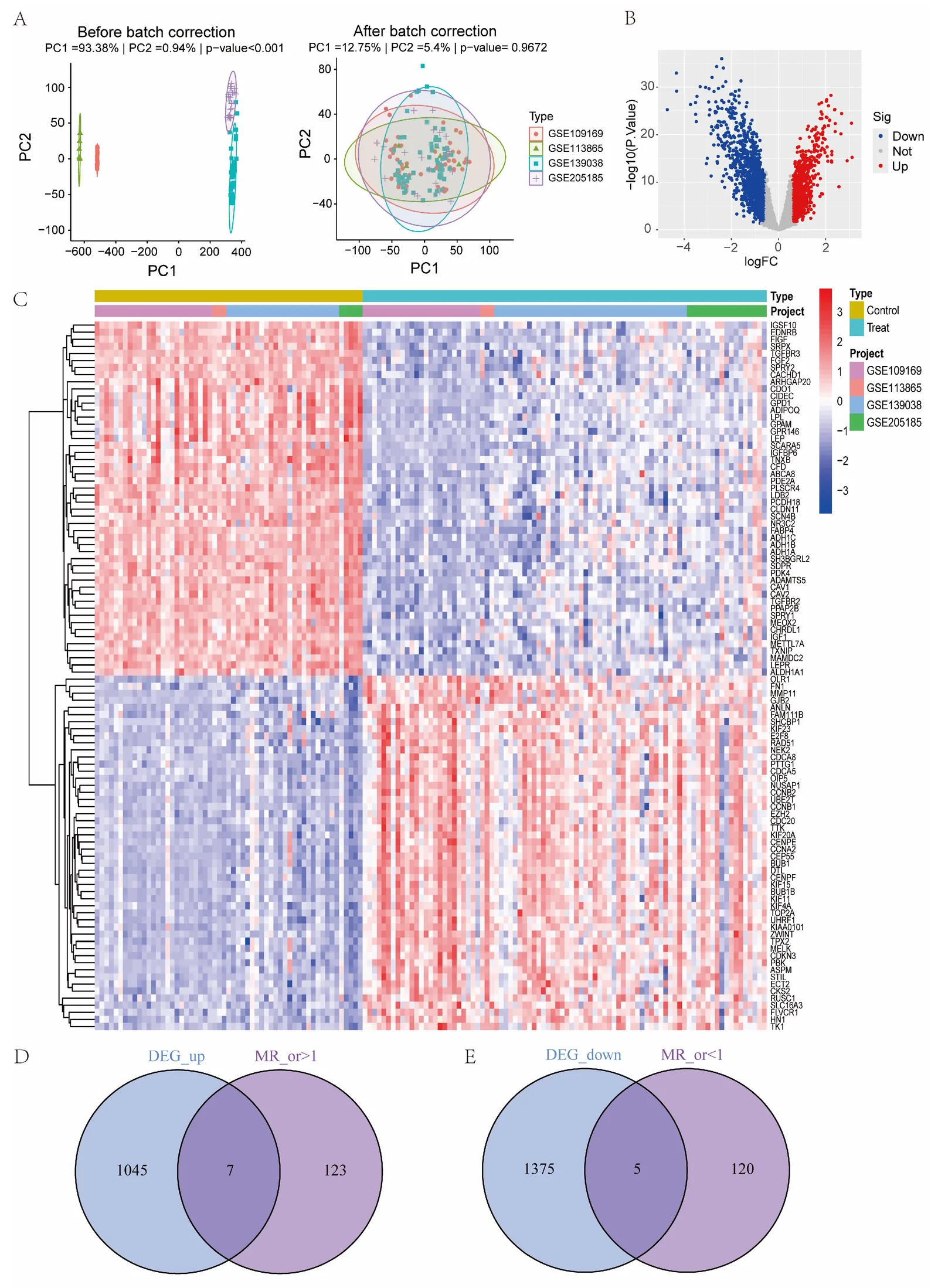
GEO data processing and integration. (**A**) Before batch correction and after batch correction. (**B**) Volcano plot for differential expression analysis of GSE109169, GSE113865, GSE139038, and GSE205185. (**C**) Heatmap for differential expression analysis of GSE109169, GSE113865, GSE139038, and GSE205185. (**D**) Venn diagram revealed 7 upregulated co-expressed genes. (**E**) Venn diagram revealed 5 downregulated co-expressed genes.

This study obtained four breast cancer microarray datasets from the GEO database to serve as the experimental group. Collectively, these datasets include 86 patients with breast cancer and 57 healthy controls. Detailed information about the datasets is presented in Table 1. Using R version 4.2.2, we normalized and merged the expression values of each gene across the datasets and then employed PCA to mitigate batch effects. Before batch correction, the variance explained by the first two principal components was as follows: PC1 (93.38%) and PC2 (0.94%), and the *p*-value obtained from the differential analysis was less than 0.001. As illustrated in Figure 1A, before batch correction, there were pronounced batch effects among the four breast cancer gene datasets. After correction, the variance explained by PC1 was 12.75% and by PC2 was 5.4%, with the *p*-value from the differential analysis being 0.9672. PCA analysis confirmed that all samples within the datasets achieved acceptable homogeneity.

### 3.2. DEG Identification

The differential expression analysis revealed that a smaller *p*-value indicates higher reliability in gene ranking for identifying significantly differentially expressed genes. Ultimately, we identified 1052 upregulated and 1380 downregulated DEGs (Supplementary Table S1). The volcano plot, integrating the merged microarray datasets from the GEO datasets, shows the fold change and *p*-values of gene expression between 57 normal and 86 breast cancer samples (Figure 1B). In addition, the heatmap represents detailed information regarding the top 50 upregulated and top 50 downregulated DEGs (Figure 1C).

### 3.3. MR Analysis

We utilized eQTL data to investigate the impact of risk single nucleotide polymorphisms (SNPs) on mRNA expression in breast cancer. Following our screening, we identified 26,152 SNPs suitable for use as instrumental variables. (Supplementary Table S2). Given that the F-statistics for all these SNPs exceeded the threshold of 10, they met our pre-defined selection criteria and showed no signs of linkage disequilibrium (r^2^ < 0.001). Hence, these SNPs were not regarded as weak instrumental factors. Subsequently, employing the two-sample MR analysis based on the above chosen SNPs, we identified 255 breast cancer-associated genes (Supplementary Table S3). Further cross-analysis identified genes co-expressed with disease-related genes and DEGs, which included seven upregulated genes (OLR1, ADIPOR1, CEACAM6, DNASE2, CD53, BMF, and CAMP) and five downregulated genes (PIK3R1, PNPLA2, ATOH8, TTC23, and CWF19L2) (Figure 1D,E).

**Figure 2:**
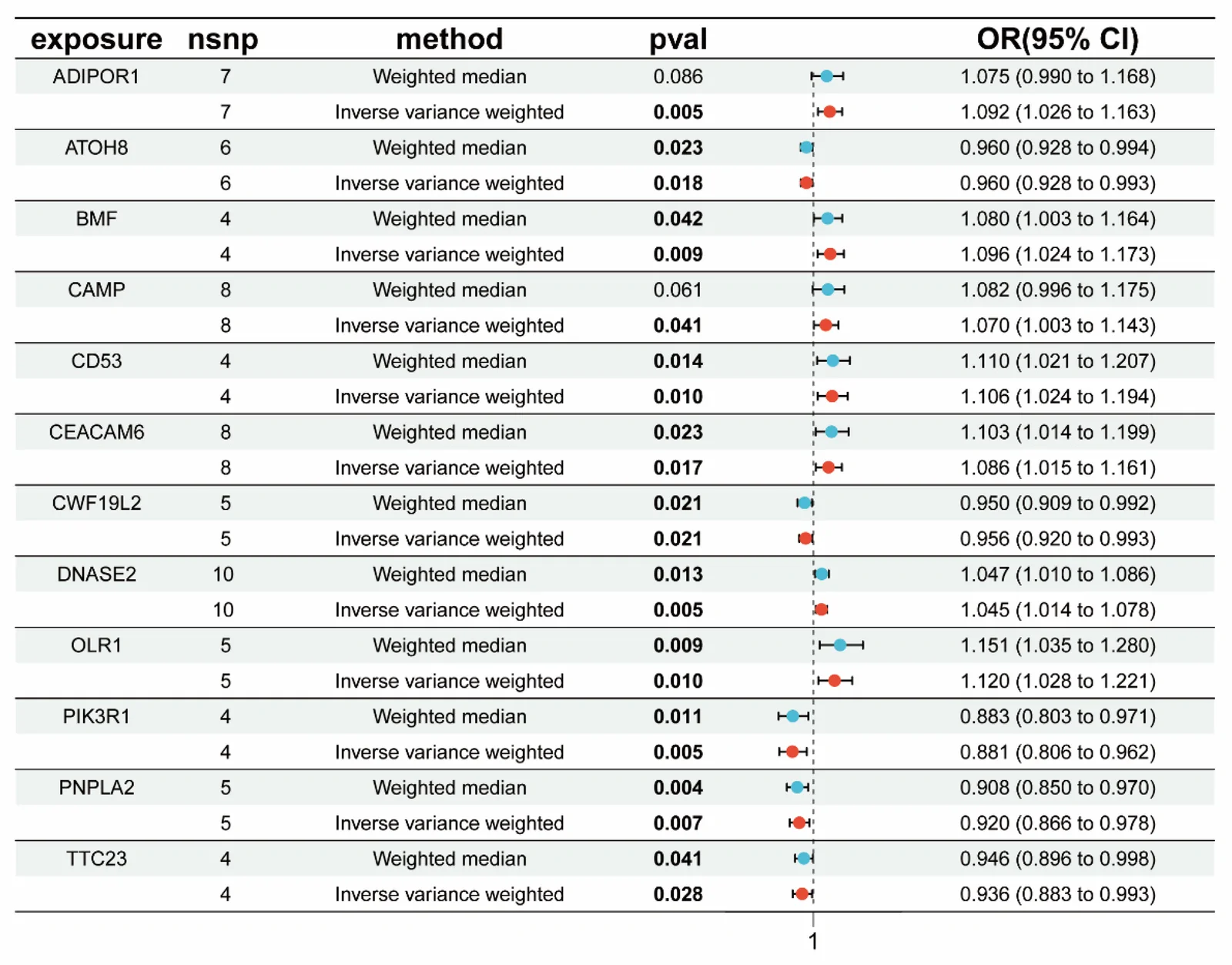
Forest plot of MR for co-expressed genes. In this plot, the results of the inverse variance weighted algorithm and the weighted median algorithm for the co-expressed genes are presented. The *p*-values and odds ratios (ORs) derived from these two methods are clearly shown. It is indicates that an OR value exceeding 1 signifies a risk factor for breast cancer, whereas an OR value less than 1 serves as a protective factor. Notably, our primary reference is the outcome of the IVW algorithm, which plays a crucial role in guiding the interpretation of the relationship between the co-expressed genes and breast cancer risk.

The primary MR analysis using the IVW approach demonstrated that all seven upregulated co-expressed genes have a significant positive causal association with breast cancer (Figure 2). Specifically, the odds ratios (ORs) and their corresponding 95% confidence intervals (CIs) were as follows: OLR1 (OR = 1.120; 95% CI: [1.028~1.221]; *p* = 0.010), ADIPOR1 (OR = 1.092; 95% CI: [1.026~1.163]; *p* = 0.005), CEACAM6 (OR = 1.086; 95% CI: [1.014~1.161]; *p* = 0.017), DNASE2 (OR = 1.045; 95% CI: [1.014~1.078]; *p* = 0.005), CD53 (OR = 1.106; 95% CI: [1.024~1.194]; *p* = 0.010), BMF (OR = 1.096; 95% CI: [1.024~1.173]; *p* = 0.009), and CAMP (OR = 1.070; 95% CI: [1.003~1.142]; *p* = 0.041). These seven upregulated co-expressed genes were identified as risk factors for breast cancer. Conversely, the five downregulated co-expressed genes showed a significant negative correlation with breast cancer, with ORs and CIs as follows: PIK3R1 (OR = 0.881; 95% CI: [0.806~0.962]; *p* = 0.005), PNPLA2 (OR = 0.920; 95% CI: [0.866~0.978]; *p* = 0.007), ATOH8 (OR = 0.960; 95% CI: [0.928~0.993]; *p* = 0.018), TTC23 (OR = 0.936; 95% CI: [0.883~0.992]; *p* = 0.028), and CWF19L2 (OR = 0.956; 95% CI: [0.919~0.993]; *p* = 0.021). These findings indicate a protective effect of the five downregulated co-expressed genes on breast cancer.

Additionally, MR-Egger, simple mode, weighted median, and weighted mode methods were applied to validate the causal effects. All methods consistently indicated an increased risk of breast cancer (OR > 1) for the seven upregulated genes, and a decreased risk of breast cancer (OR < 1) for the five downregulated genes (Figure 1). Tests for heterogeneity and pleiotropy among the co-expressed genes showed no statistical significance (*p*-values > 0.05), indicating that there were no potentially influential SNP driving the causal link and the results were robust (Figure S2). Detailed gene-specific information, including scatter plots, forest plots, funnel plots, and sensitivity analysis (leave-one-out), is provided in Supplementary Figure S1.

### 3.4. GO and KEGG Enrichment Analysis

**Figure 3:**
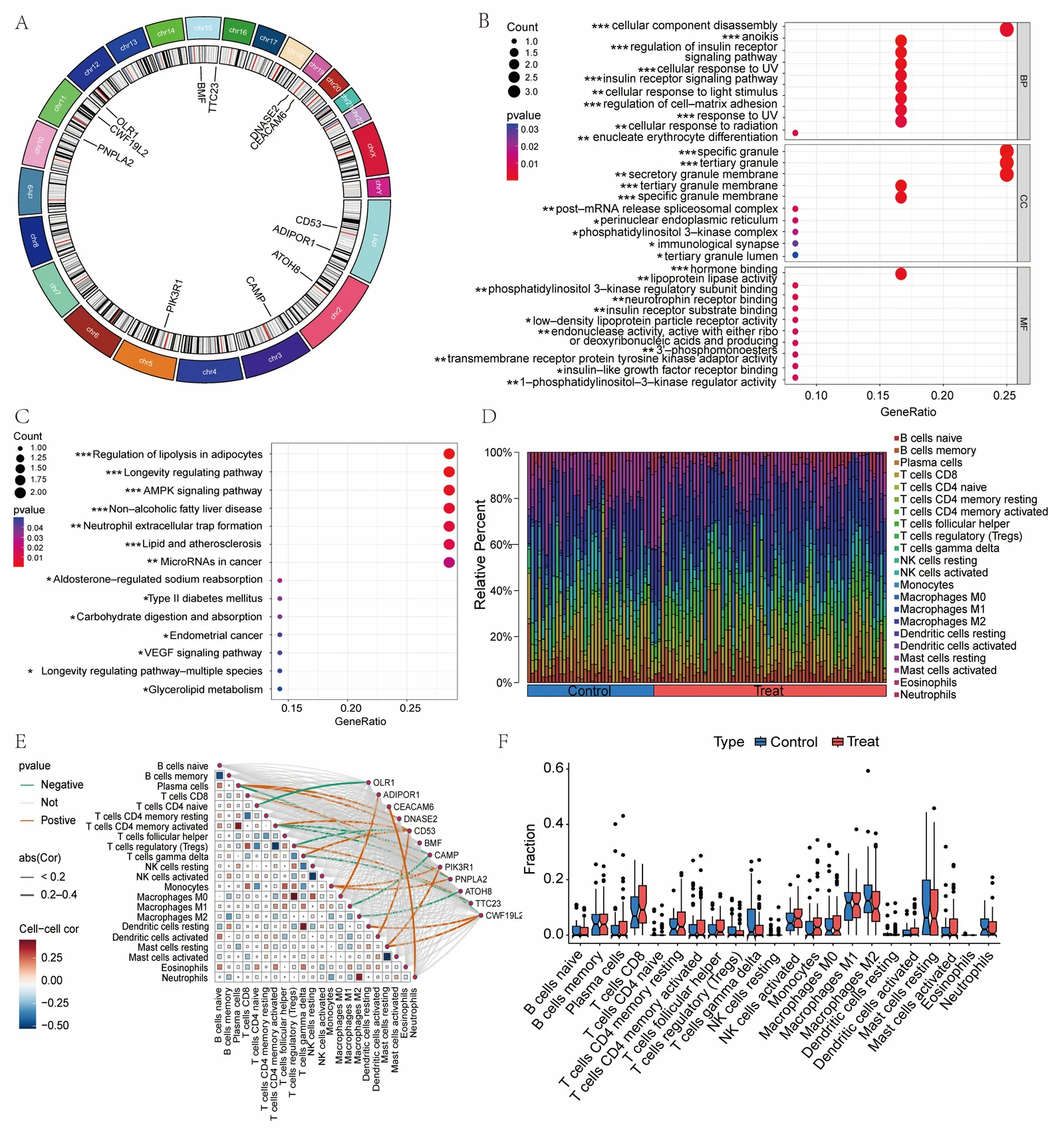
Analysis of chromosomal locations, functional pathways, and immune infiltration for co-expressed genes. (**A**) Chromosomal localization of co-expressed genes. (**B**) GO enrichment analysis of co-expressed genes. (**C**) KEGG enrichment analysis of co-expressed genes. (**D**,**F**) Comparison of immune cell proportions between breast cancer and control groups, displaying differences in 22 types of immune cells. (**E**) Heatmap displaying the correlations between 22 types of immune cells and co-expressed genes. * *p* < 0.05, ** *p* < 0.01, *** *p* < 0.005.

To better understand the chromosomal distribution of the genes, we visualized the co-expressed genes using a circus plot (Figure 3A). We then performed GO and KEGG analyses to explore the potential functions of these genes. GO analysis revealed that the co-expressed genes were primarily involved in cellular component disassembly, specific granules, tertiary granules, and secretory granule membranes (Figure 3B). KEGG enrichment analysis revealed that these genes predominantly influenced various pathways involved in lipid metabolism and immune responses, such as MicroRNAs in cancer, lipid and atherosclerosis, neutrophil extracellular trap formation, non-alcoholic fatty liver disease, AMPK signaling pathway, and regulation of lipolysis in adipocytes (Figure 3C). Detailed data regarding these analyses can be found in Supplementary Table S4.

### 3.5. Evaluation of Immune Cell Infiltration in Breast Cancer

The functional and pathway analysis of the co-expressed genes in breast cancer indicates a potential link between breast cancer pathogenesis and neutrophil extracellular trap formation. To further investigate this association, we employed the CIBERSORT algorithm to analyze immune cell characteristics and explore the correlation between co-expressed genes and immune cell infiltration in breast cancer. Specifically, the CIBERSORT algorithm allowed us to estimate the relative abundance of 22 different immune cell types across each sample. Figure 3D presents the distribution of these immune cell types in the breast cancer samples.

Next, we performed a correlation analysis between the expression levels of co-expressed genes and the immune cell types. Analysis of the correlation with 22 immune cell types (Figure 3E) revealed that the co-expressed gene OLR1 was negatively correlated with T cells CD4 naive; ADIPOR1 was positively correlated with Macrophages M0; CEACAM6 was positively correlated with Neutrophils; DNASE2 was positively correlated with Plasma cells; CD53 showed positive correlations with Plasma cells, T cells CD4 memory activated, T cells gamma delta, and Dendritic cells activated, and a negative correlation with T cells regulatory (Tregs); CAMP was negatively correlated with T cells CD4 memory activated and Macrophages M1; PIK3R1 was positively correlated with Monocytes and Mast cells resting; PNPLA2 showed positive correlations with T cells CD8 and Monocytes, and a negative correlation with T cells CD4 memory activated; ATOH8 was negatively correlated with Macrophages M2; TTC23 was positively correlated with T cells regulatory (Tregs) and negatively with T cells gamma delta; and CWF19L2 was positively correlated with Dendritic cells resting and Mast cells resting, and negatively with Plasma cells. These correlations suggest that the expression of these genes may influence immune cell dynamics within the tumor microenvironment.

However, when we compared the immune infiltration between breast cancer samples and control samples using violin plots (Figure 3F), we did not observe any statistically significant differences in immune cell infiltration. This indicates that, despite the observed correlations between co-expressed genes and immune cells, immune cell infiltration levels between breast cancer and control samples were not markedly different.

### 3.6. Validation of Results Using TCGA Data

**Figure 4:**
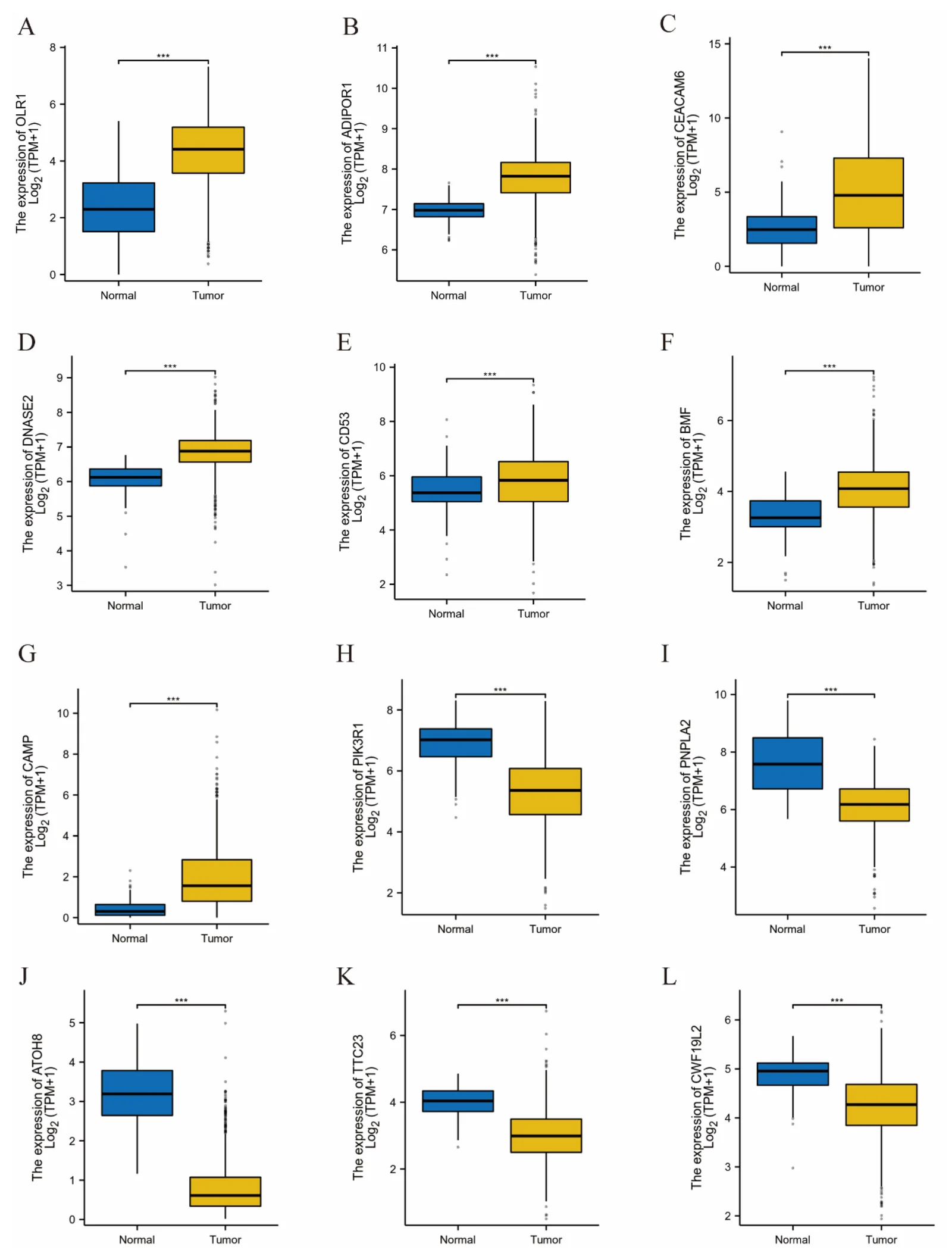
Validation of expression levels of 12 co-expressed genes in the TCGA Database. (**A**–**G**) Genes OLR1, ADIPOR1, CEACAM6, DNASE2, CD53, BMF, and CAMP display significantly elevated expression levels in breast cancer samples relative to healthy controls. (**H**–**L**) Conversely, genes PIK3R1, PNPLA2, ATOH8, TTC23, and CWF19L2 exhibit dramatically lower expression levels in breast cancer samples compared to healthy controls. * *p* < 0.05, ** *p* < 0.01, *** *p* < 0.005.

We then conducted an external validation to confirm the differential expression of the co-expressed genes identified in our MR analysis between breast cancer and adjacent non-cancerous tissue samples from the TCGA database. The results demonstrated that the expression levels of OLR1, ADIPOR1, CEACAM6, DNASE2, CD53, BMF, and CAMP were significantly elevated in breast cancer samples compared to healthy controls (*p* < 0.05) (Figure 4A–G). In contrast, the expression levels of PIK3R1, PNPLA2, ATOH8, TTC23, and CWF19L2 were dramatically lower in breast cancer samples than in healthy controls (*p* < 0.05) (Figure 4H–L). Therefore, the validation by the external TCGA dataset enhanced the credibility of our MR analysis.

### 3.7. Construction of PPI Networks and Nomogram Model

**Figure 5:**
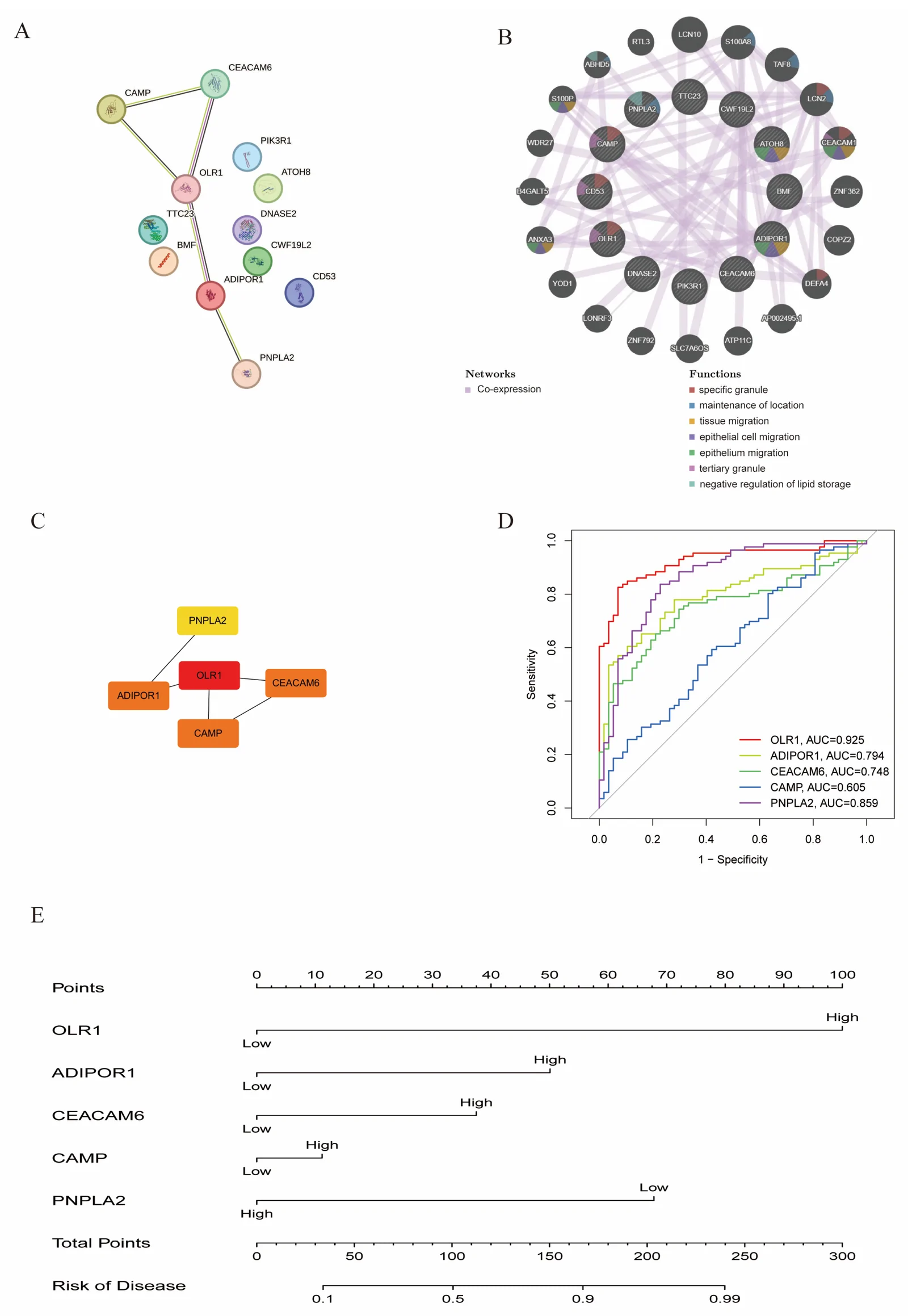
Construction of PPI network and nomogram for predicting breast cancer risk. (**A**) Using STRING to construct the PPI network. The interactions involved in this construction encompass Textmining, Experiments, Databases, Co-expression, Neighborhood, Gene Fusion, and Co-occurrence. (**B**) Establishing the PPI network using GeneMANIA, with each circle colored to indicate the functional pathways involved by each gene. (**C**) Identifying the core genes of the interaction network using the degree algorithm. (**D**) Evaluating the diagnostic efficacy of the nomogram model and each co-expressed gene with ROC curves. (**E**) Nomogram model for co-expressed genes.

We used the STRING online tool (https://cn.string-db.org/) (accessed on 1 July 2024) to construct a Protein–Protein Interaction (PPI) network composed of overlapping core genes. The types of interactions included in this network are as follows: Textmining, Experiments, Databases, Co-expression, Neighborhood, Gene Fusion, and Co-occurrence. We set the minimum required interaction score to 0.15, and the result is presented in Figure 5A. Furthermore, we employed the GeneMANIA platform (https://GeneMANIA.org/) (accessed on 1 July 2024) to develop another PPI network, which not only included the 12 intersecting genes from the STRING analysis but also incorporated an additional 20 potential interacting genes (Figure 5B). Interactions within this network predominantly manifested as co-expression. Functional analysis of this network provided valuable insights into the intersecting genes and their associated genes. The functional analysis of the PPI network primarily focused on specific granules and migration, aligning with the above GO and KEGG enrichment analyses. These findings provided valuable guidance for our further investigation into these genes.

Subsequently, we employed Cytoscape software (v3.10.2) to determine the most closely related genes among the PPI networks. The genes CEACAM6, CAMP, PNPLA2, OLR1, and ADIPOR1 were specifically identified and highlighted (Figure 5C). The color intensity corresponded to the magnitude of the scores, with darker hues indicating higher values. We then calculated the Receiver Operating Characteristic (ROC) curves for these five central genes to assess their diagnostic performance. The Area Under the Curve (AUC) values for CEACAM6, CAMP, PNPLA2, OLR1, and ADIPOR1 were 0.748, 0.605, 0.859, 0.925, and 0.794, respectively (Figure 5D). Finally, we developed a columnar line chart model to predict the risk of breast cancer occurrence associated with these five central genes (Figure 5E). Collectively, these findings delineated a significant promotional effect of OLR1 on breast cancer progression, with the darkest color, highest AUC value, and greatest risk, while a notable protective function of PNPLA2 (Figure 5C–E). Additionally, previous research has confirmed the roles of these five central genes (CEACAM6 [28], CAMP [29], PNPLA2 [30], OLR1 [31], and ADIPOR1 [32]) in breast cancer, which were consistent with our predictions of risk and protective factors. Other co-expressed genes such as BMF [33], CD53 [34], PIK3R1 [35], and CWF19L2 [36] have also been reported in breast cancer studies. Given the challenges associated with experimental studies on TTC23, we have decided to further investigate the biological functions of two genes, ATOH8 and DNASE2, which have not been extensively studied in breast cancer.

### 3.8. In Vitro Functional Investigation into the Roles of DNASE2 and ATOH8 in Breast Cancer Development

**Figure 6:**
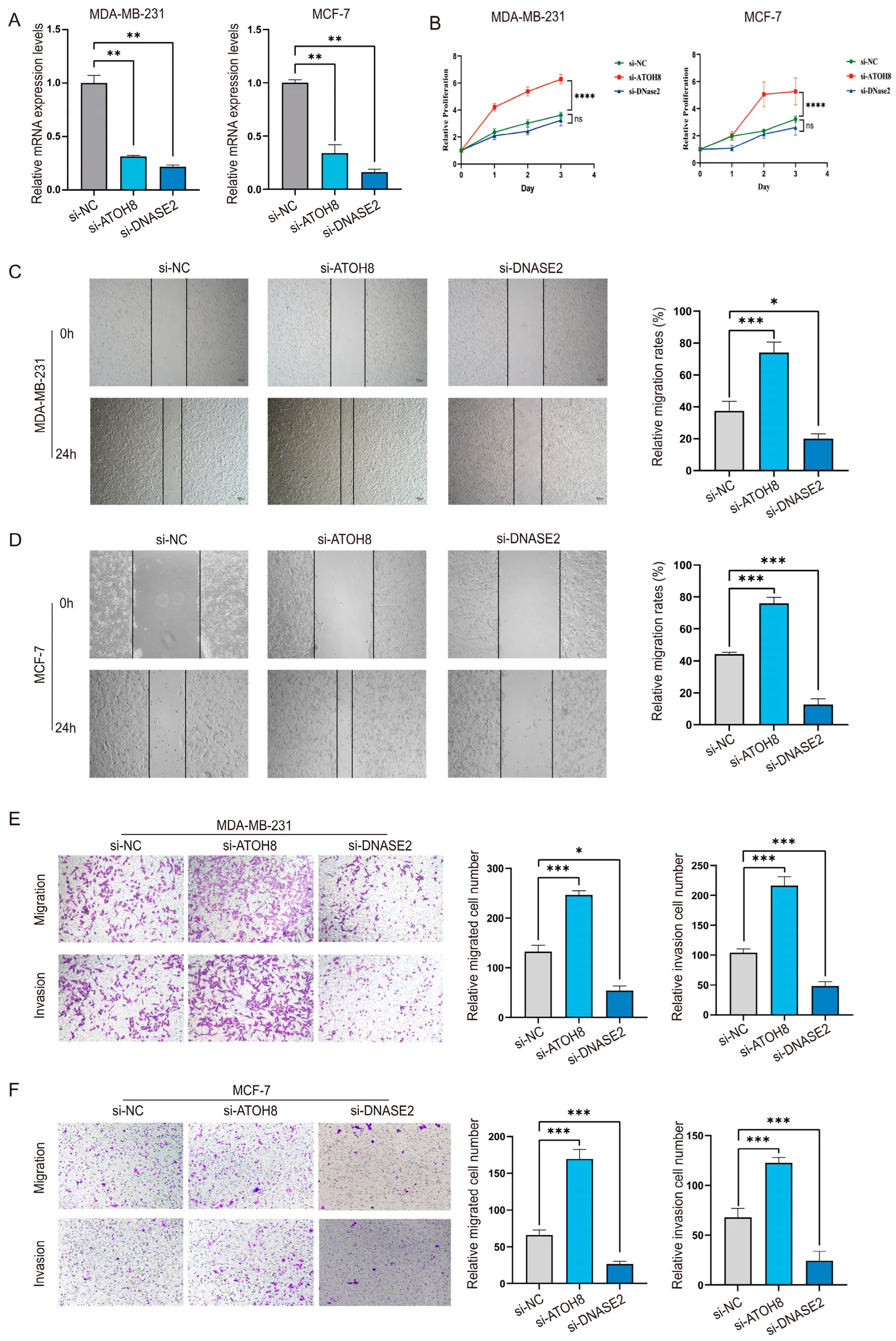
Cellular functional experiments for DNASE2 and ATOH8. (**A**) Validation of DNASE2 and ATOH8 knockdown via siRNA. (**B**) Using the MTS assay to assess cell proliferation of DNASE2 and ATOH8 in MDA-MB-231 and MCF-7 cell lines after siRNA treatment. (**C**,**D**) Evaluating the migration ability of MDA-MB-231 and MCF-7 cells after silencing DNASE2 and ATOH8 through scratch assay. (**E**,**F**) Assessing the migration and invasion capabilities of cells via Transwell assay after silencing DNASE2 and ATOH8. “ns” indicates “not significant”, * *p* < 0.05, ** *p* < 0.01, *** *p* < 0.005, **** *p* < 0.001.

Considering the lack of research on the genes DNASE2 and ATOH8 in the context of breast cancer, we deemed it necessary to further elucidate their functions in breast cancer progression. Functional studies were conducted to assess the phenotypic effects of siRNA-mediated knockdown of DNASE2 and ATOH8 with siRNA in the breast cancer cell lines MCF-7 and MDA-MB-231. The expression of both DNASE2 and ATOH8 was obviously reduced in these cell lines (Figure 6A). MTS assays revealed that compared to the negative control (NC), the knockdown of ATOH8 significantly enhanced the proliferative activity of both MCF-7 and MDA-MB-231 cells, whereas silencing DNASE2 did not show remarkable changes (Figure 6B). Scratch assays demonstrated silencing ATOH8 obviously facilitated wound healing, while knockdown DNASE2 had the opposite effect, implicating the promotive and inhibitory roles of DNASE2 and ATOH8 in the migration of breast cancer cells, respectively (Figure 6C,D). Additionally, the Transwell assays also demonstrated that the knockdown of DNASE2 reduced, while the knockdown of ATOH8 enhanced, the migration and invasion capabilities of both cell lines (Figure 6E,F), further confirming their distinct roles in cell migration. In summary, our findings revealed that DNASE2 significantly promoted the migration and invasion of breast cancer cells, whereas ATOH8 dramatically inhibited proliferation, migration, and invasion. These results suggested that DNASE2 and ATOH8 exhibited risk and protective factors, respectively, for breast cancer pathogenesis, which was consistent with our MR predictions.

### 3.9. GSEA Enrichment Analysis

**Figure 7:**
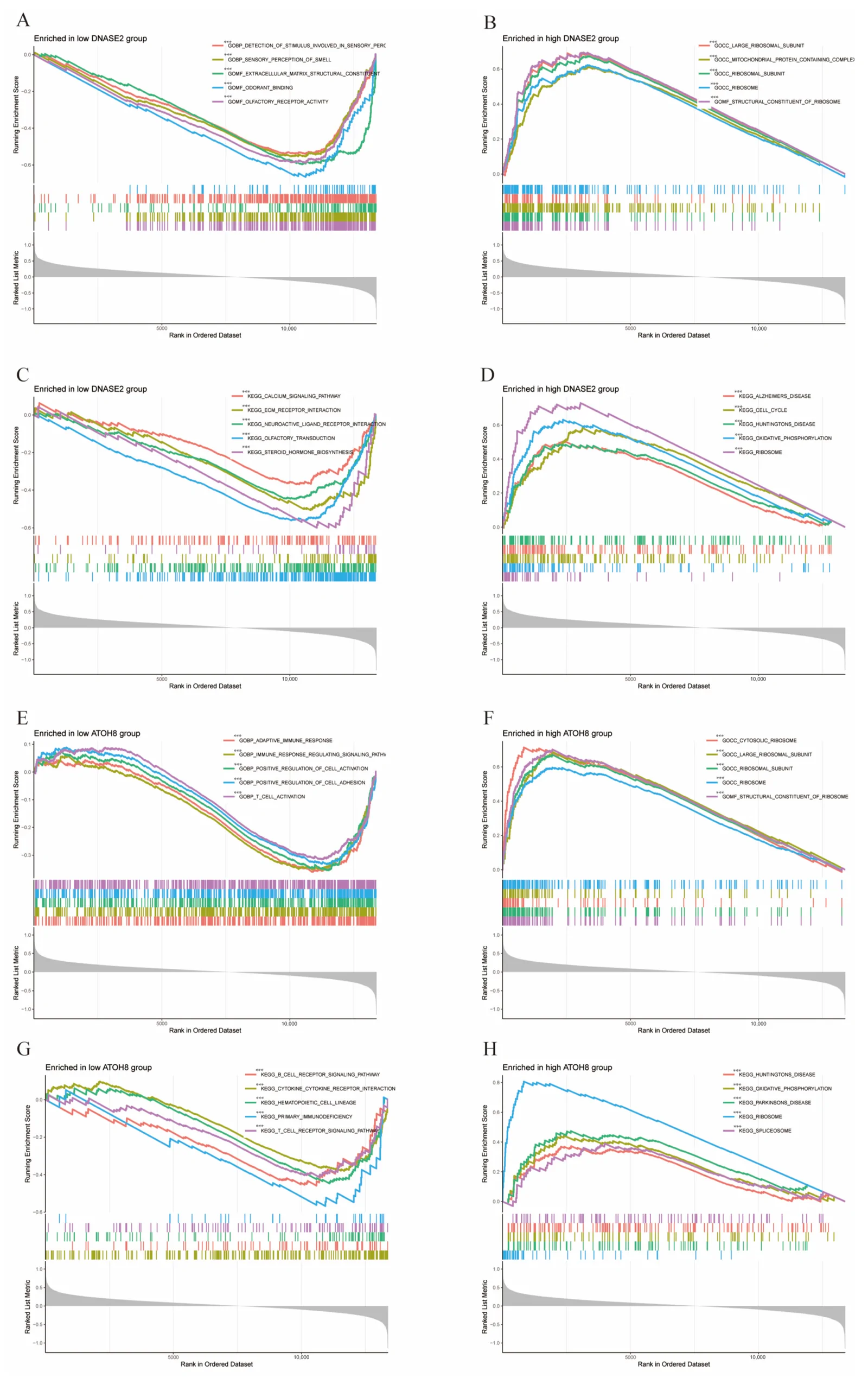
GSEA analysis of the differential effects of DNASE2 and ATOH8 expression on biological functions and pathways in breast cancer. (**A**) Top 5 active biological functions in the DNASE2 low expression group. (**B**) Top 5 active biological functions in the DNASE2 high expression group. (**C**) Top 5 active pathways in the DNASE2 low expression group. (**D**) Top 5 active pathways in the DNASE2 high expression group. (**E**) Top 5 active biological functions in the ATOH8 low expression group. (**F**) Top 5 active biological functions in the ATOH8 high expression group. (**G**) Top 5 active pathways in the ATOH8 low expression group. (**H**) Top 5 active pathways in the ATOH8 high expression group. * *p* < 0.05, ** *p* < 0.01, *** *p* < 0.005.

GSEA was used to assess whether the functions or pathways related to co-expressed genes were enriched at either end of the ranking, reflecting upregulation or downregulation trends. We then applied GSEA to further explore the functional activities and pathways linked to the expression profiles of DNASE2 and ATOH8. Our findings indicated that low expression of DNASE2 was strongly correlated with sensory functions, calcium signaling pathways, and interactions with receptor ligands. (Figure 7A,C). Conversely, high expression of DNASE2 was significantly associated with the structure and function of mitochondria and ribosomes, as well as Alzheimer’s disease (Figure 7B,D). Additionally, low expression of ATOH8 was obviously linked to cellular functions and the regulation of immune cells (Figure 7E,G). In contrast, high expression of ATOH8 was closely related to the structure and function of ribosomes and Parkinson’s disease (Figure 7F,H). These insights provided a novel perspective on the roles of these genes in physiological and pathological processes in breast cancer.

### 3.10. Drug Sensitivity Between High and Low Expression Groups of ATOH8 and DNASE2 Genes

**Figure 8:**
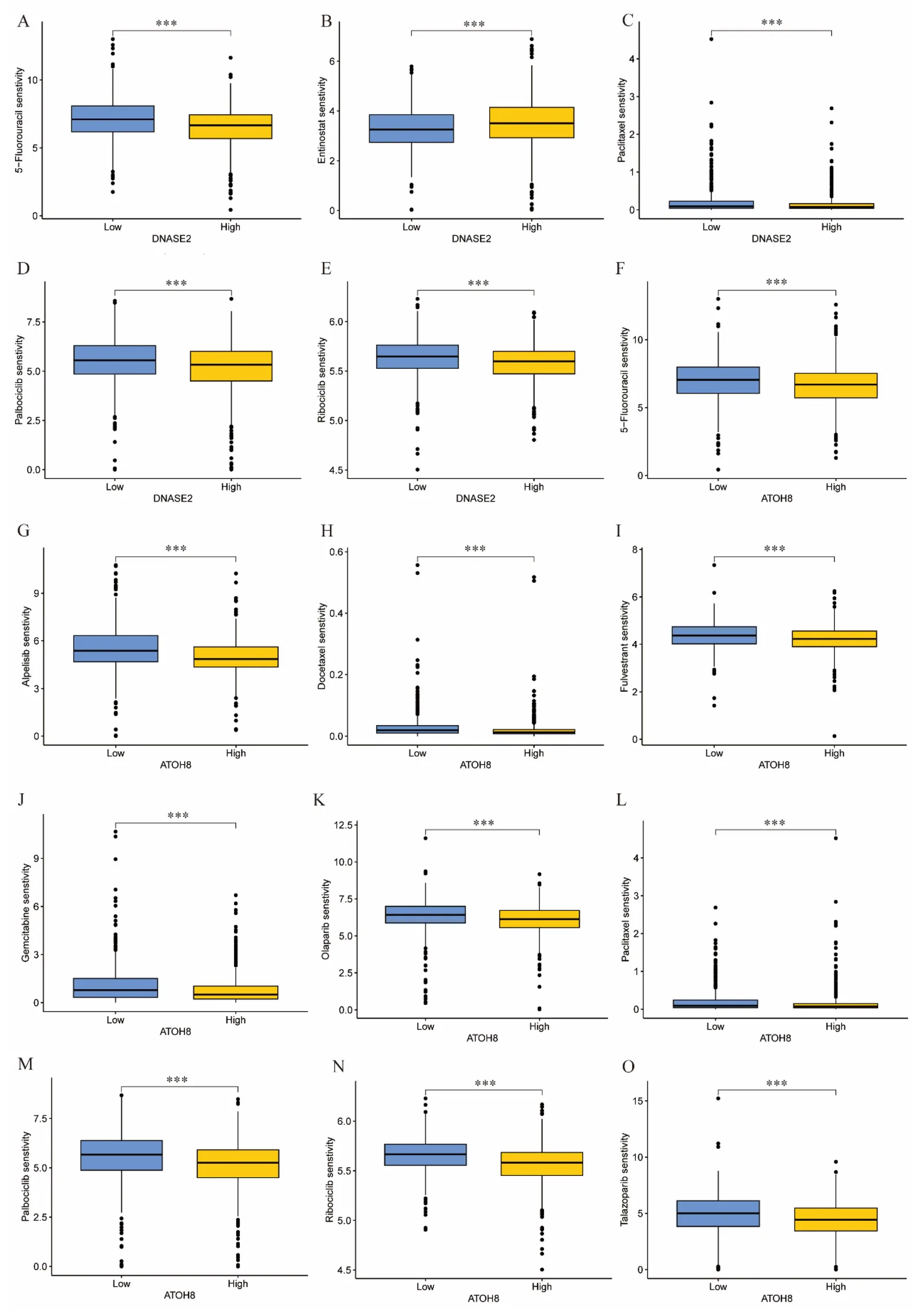
Analysis of drug sensitivity for DNASE2 and ATOH8. (**A**–**E**) For DNASE2, the high-expression group demonstrated increased sensitivity to 5-Fluorouracil, paclitaxel, palbociclib, and ribociclib, while the low-expression group was more sensitive to Entinostat. (**F**–**O**) Regarding ATOH8, drugs like 5-Fluorouracil and alpelisib showed higher sensitivity in the high-expression group. * *p* < 0.05, ** *p* < 0.01, *** *p* < 0.005.

Furthermore, we conducted drug sensitivity analyses to explore the relationship between commonly used clinical drugs and the expression levels of ATOH8 and DNASE2 genes. Significant statistical differences in sensitivity were observed across a variety of commonly used clinical drugs (Figure 8 and Figure S2). Specifically, the group with high DNASE2 expression exhibited increased sensitivity to 5-Fluorouracil, paclitaxel, palbociclib, and ribociclib, while the DNASE2 low expression group showed greater sensitivity to Entinostat (Figure 8A–E). Additionally, drugs such as 5-Fluorouracil and alpelisib, among others, exhibited higher sensitivity in the ATOH8 high expression group (Figure 8F,O). Importantly, the aforementioned drugs, which exhibited significant differences in sensitivity between high and low gene expression groups, are commonly utilized in clinical practice for breast cancer treatment. For analysis of other drugs that are less commonly used or unapproved in clinical settings, please refer to Supplementary Figure S2. These findings hold substantial significance for guiding clinical medication practices to optimize treatment plans and enhance therapeutic efficacy.

## 4. Discussion

Breast cancer is the most prevalent malignancy among women globally and remains a leading cause of cancer-related mortality [37]. Despite advances in diagnosis and treatment over the past decades, the heterogeneity in therapeutic responses, the emergence of resistance to existing treatments, and the limit of broad-spectrum applicability of targeted therapy underscore the urgent need for continued research to discover new molecular targets [9]. Our in-depth analysis has offered valuable insights into the molecular mechanisms underlying breast cancer. By analyzing GEO datasets, we identified 1052 upregulated and 1380 downregulated DEGs. Subsequent MR analysis revealed key genes potentially associated with breast cancer, culminating in the identification of 12 co-expressed genes, encompassing seven upregulated genes (OLR1, ADIPOR1, CEACAM6, DNASE2, CD53, BMF, and CAMP) and five downregulated genes (PIK3R1, PNPLA2, ATOH8, TTC23, and CWF19L2). Through GO and KEGG enrichment analyses, we elucidated that these co-expressed genes were primarily enriched in lipid metabolism and immune responses via regulating microRNA functions and AMPK signaling, highlighting their potential as therapeutic targets. Furthermore, external validation through the TCGA database confirmed the differential expression of these co-expressed genes in breast cancer tissues, further supporting the reliability of our results. Additionally, the nomogram model using the STRING tool might provide an effective tool for clinically predicting the risk of breast cancer.

Except for ATOH8, DNASE2, and TTC23, the involvement of the other nine co-expressed genes has been reported in breast cancer [28,29,30,31,32,33,34,35,36]. Given the challenges associated with experimental studies on TTC23, we deemed it necessary to further elucidate the biological functions of DNASE2 and ATOH8 in the context of breast cancer. Functional validation experiments revealed that ATOH8 strongly inhibited proliferation, migration, and invasion of breast cancer cells, whereas DNASE2 significantly enhanced migration and invasion of cellular behaviors, indicating the inhibitory and promotive roles of ATOH8 and DNASE2 in the pathogenesis of breast cancer, respectively.

ATOH8, a transcription factor, is known to regulate cell differentiation and proliferation, and in this study, its expression appeared to suppress tumor progression by inhibiting cell migration and invasion. ATOH8 has previously been reported to inhibit carcinogenic Ras-driven lung tumorigenesis [38]. However, other research indicated that ATOH8 promoted vascular survival of colorectal cancer cells through transcriptional activation of HK2-mediated glycolysis [39]. Notably, recent studies have identified a novel isoform of ATOH8, ATOH8-V1, which was highly expressed in breast cancer and promoted metastasis by regulating RhoC [40]. Furthermore, ATOH8 has been found to bind with SMAD3, forming a transcriptional complex that activates the TGF-β signaling pathway, induces cellular senescence, and suppresses tumor transformation [38]. However, a bispecific antibody targeting TGF-β/PD-L1, developed by Youzhi You Bio, has shown enhanced antitumor activity in triple-negative breast cancer models, suggesting that combined targeting of TGF-β and PD-L1 could be an effective therapeutic strategy [41]. Therefore, TGF-β plays both inhibitory and promotive roles in breast cancer. The role of ATOH8 in different cancers, or even in different subtypes within the same cancer, is complex and multifaceted, making it an intriguing target for further investigation.

In contrast, DNASE2, an intracellular deoxyribonuclease, primarily targets and degrades cytoplasmic DNA [42]. In liver cancer research, evidence suggested that the knockdown of DNASE2 suppressed proliferation and promoted apoptosis of liver cancer cells, indicating the inhibitory function of DNASE2 in the development of liver cancer [43]. Additionally, studies have indicated that the activation of the STING pathway is associated with breast cancer drug resistance, and inhibiting this pathway may enhance the effectiveness of conventional chemotherapy [44]. Tumor-derived DNA, such as DNA from dying tumor cells, can activate the cGAS pathway, which implies that DNASE2 may be involved in this process. Our study suggests that DNASE2 may promote tumor cell growth and could potentially play a role in enhancing the invasive and migratory behaviors of breast cancer cells, highlighting its potential involvement in the progression of breast cancer.

In addition, the contrasting effects of ATOH8 and DNASE2 on cell proliferation and migration further underscore their potential as therapeutic targets in breast cancer. ATOH8’s inhibitory effect on cell migration and invasion contrasts with DNASE2’s promotion of these cellular processes, suggesting that their distinct roles could be leveraged in developing targeted therapies to modulate breast cancer progression.

Furthermore, we conducted GSEA and drug sensitivity analysis, laying the groundwork for further exploration of the biological mechanisms of these genes and providing novel insights for future research directions and clinical medication practices.

In summary, our extensive research into breast cancer has revealed significant insights into its molecular mechanisms, identifying novel therapeutic targets and potential drugs. However, despite these advancements, our findings remain preliminary and require further validation through additional experiments and clinical trials to confirm the mechanisms and therapeutic potential of these genes. Therefore, although our study provides new directions for breast cancer treatment, it is crucial to enhance and expand research across various aspects to fully comprehend the complexity of breast cancer.

## 5. Conclusions

This study comprehensively analyzed omics data and conducted experimental verification to explore new breast cancer therapeutic targets. By analyzing GEO database gene expression profiles, numerous DEGs were identified, and MR analysis pinpointed 12 co-expressed genes linked to breast cancer.

GO and KEGG enrichment analyses showed these genes were mainly involved in lipid metabolism and immune response-related pathways. TCGA database validation confirmed their differential expression in breast cancer tissues, and PPI network and nomogram model construction aided in understanding gene relationships and cancer risk prediction.

Functional assays on DNASE2 and ATOH8 revealed their opposing roles in breast cancer cell behavior, consistent with MR analysis predictions. Drug sensitivity analysis indicated significant differences in drug responses based on their expression levels, offering a basis for personalized treatment.

In summary, this study uncovered new molecular mechanisms and therapeutic strategies for breast cancer. However, as the findings are preliminary, further in-depth research and large-scale clinical trials are required to fully explore gene functions and their therapeutic potential, aiming to develop more effective treatments for breast cancer patients.

## Figures and Tables

**Table 1:** Characteristics of the four GEO datasets.

GSE ID	Samples	Tissues	Platform	Experiment Type
GSE109169	25 cases and 25 controls	Breast cancer	GPL5175	13 January 2018
GSE113865	3 cases and 3 controls	Breast cancer	GPL10558	30 April 2023
GSE139038	41 cases and 24 controls	Breast cancer	GPL27630	18 October 2019
GSE205185	17 cases and 5 controls	Breast cancer	GPL21158	1 June 2022

## Data Availability

The data that support the findings of this study are available from the corresponding author upon reasonable request.

## Supplementary Materials

The following supporting information can be downloaded at: https://www.mdpi.com/article/10.3390/biology14040405/s1, Figure S1: Scatter plots, forest plots, funnel plots and sensitivity analyses generated by Mendelian randomization of breast cancer and eQTL; Figure S2: Supplementary part of drug sensitivity analysis of ATOH8 and DNASE2 genes; Table S1: Differential gene expression profile in breast cancer; Table S2: Results extracted after Mendelian randomization of eQTL; Table S3: Results of Mendelian randomization of eQTL and breast cancer; Table S4: Results of GO and KEGG analyses.

## Abbreviations

DEGs	Differentially expressed genes
eQTL	Expression quantitative trait loci
GO	Gene Ontology
KEGG	Kyoto Encyclopedia of Genes and Genomes
MR	Mendelian randomization
PPI	Protein–protein interaction
LogFC	LogFoldChange
PCA	Principal component analysis
IVW	Inverse variance weighted
ROC	Receiver operator characteristic
