# Correction: Chai et al. Exploring Novel Therapeutic Targets in Breast Cancer via Comprehensive Omics Profiling and Experimental Verification. *Biology* 2025, *14*, 405

**DOI:** 10.3390/biology15161440

**Published:** 2026-08-21

**Authors:** Shengjun Chai, Jiayong Cui, Yinuo Sun, Xiaowu Wang, Chunmei Cai

**Affiliations:** 1 Research Center for High Altitude Medicine, Qinghai University Medical College, Xining 810008, China; ys221002100819@qhu.edu.cn (S.C.); ys221002100823@qhu.edu.cn (J.C.); 19902747732@163.com (Y.S.); 2 Key Laboratory of the Ministry of High Altitude Medicine, Qinghai University Medical College, Xining 810008, China; 3 Key Laboratory of Applied Fundamentals of High Altitude Medicine (Qinghai-Utah Joint Key Laboratory of Plateau Medicine), Qinghai University Medical College, Xining 810008, China; 4 Laboratory for High Altitude Medicine of Qinghai Province, Qinghai University Medical College, Xining 810008, China; 5 Department of Surgical Oncology, The Affiliated Hospital of Qinghai University, Xining 810001, China; 6 Department of Economics, School of Finance and Economics, Qinghai University, Xining 810016, China

## Error in Figure

In the original publication [1], there were errors in Figure 6C–F. During the preparation and assembly of the figure, the label “si-DNASE2” was inadvertently misspelled as “si-NDASE2” in Figure 6C–F. In addition, the image presented in Figure 6F was incorrectly selected during the post-experimental figure organization and layout process. Following a comprehensive re-examination of the original experimental records and source image files, the labels in Figure 6C–F have been corrected from “si-NDASE2” to “si-DNASE2”, and Figure 6F has been replaced with the correct image derived from the original experimental materials. The corrected Figure 6 appears below. These corrections concern only the labeling and presentation of the figure and do not affect the underlying experimental data, the interpretation of the results, or the scientific conclusions of the article. The authors state that the scientific conclusions are unaffected. This correction was approved by the Academic Editor. The original publication has also been updated.

## Figures and Tables

**Figure 6:**
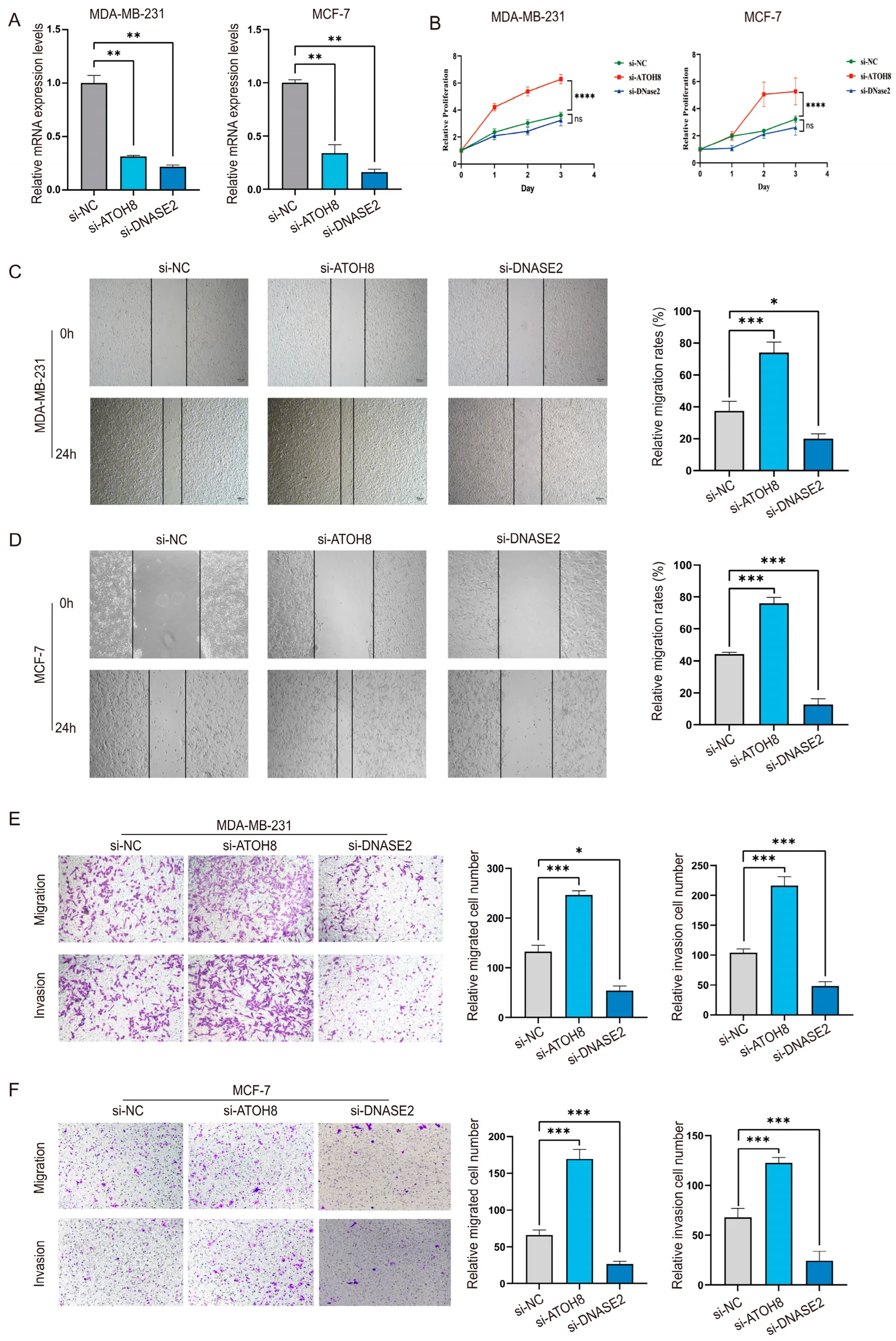
Cellular functional experiments for DNASE2 and ATOH8. (**A**) Validation of DNASE2 and ATOH8 knockdown via siRNA. (**B**) Using the MTS assay to assess cell proliferation of DNASE2 and ATOH8 in MDA-MB-231 and MCF-7 cell lines after siRNA treatment. (**C**,**D**) Evaluating the migration ability of MDA-MB-231 and MCF-7 cells after silencing DNASE2 and ATOH8 through scratch assay. (**E**,**F**) Assessing the migration and invasion capabilities of cells via Transwell assay after silencing DNASE2 and ATOH8. “ns” indicates “not significant”, * *p* < 0.05, ** *p* < 0.01, *** *p* < 0.005, **** *p* < 0.001.

## References

[1] Chai S., Cui J., Sun Y., Wang X., Cai C. (2025). Exploring Novel Therapeutic Targets in Breast Cancer via Comprehensive Omics Profiling and Experimental Verification. Biology.

