# Supplementary material for: Exploring Novel Therapeutic Targets in Breast Cancer via Comprehensive Omics Profiling and Experimental Verification
Source: Biology (Basel). 2025 Apr 11;14(4):405. doi: 10.3390/biology14040405 (PMC12025194; doi:10.3390/biology14040405)
Supplement: Supplementary file 1 [file biology-14-00405-s001.zip › Supplementary Figure S1.pdf]

Exploring Novel Therapeutic Targets in Breast Cancer via Comprehensive Omics Profiling and Experimental Verification

Supplementary Figure 1 Scatter plots, forest plots, funnel plots and sensitivity analyses generated by Mendelian randomization of breast cancer and eQTL.

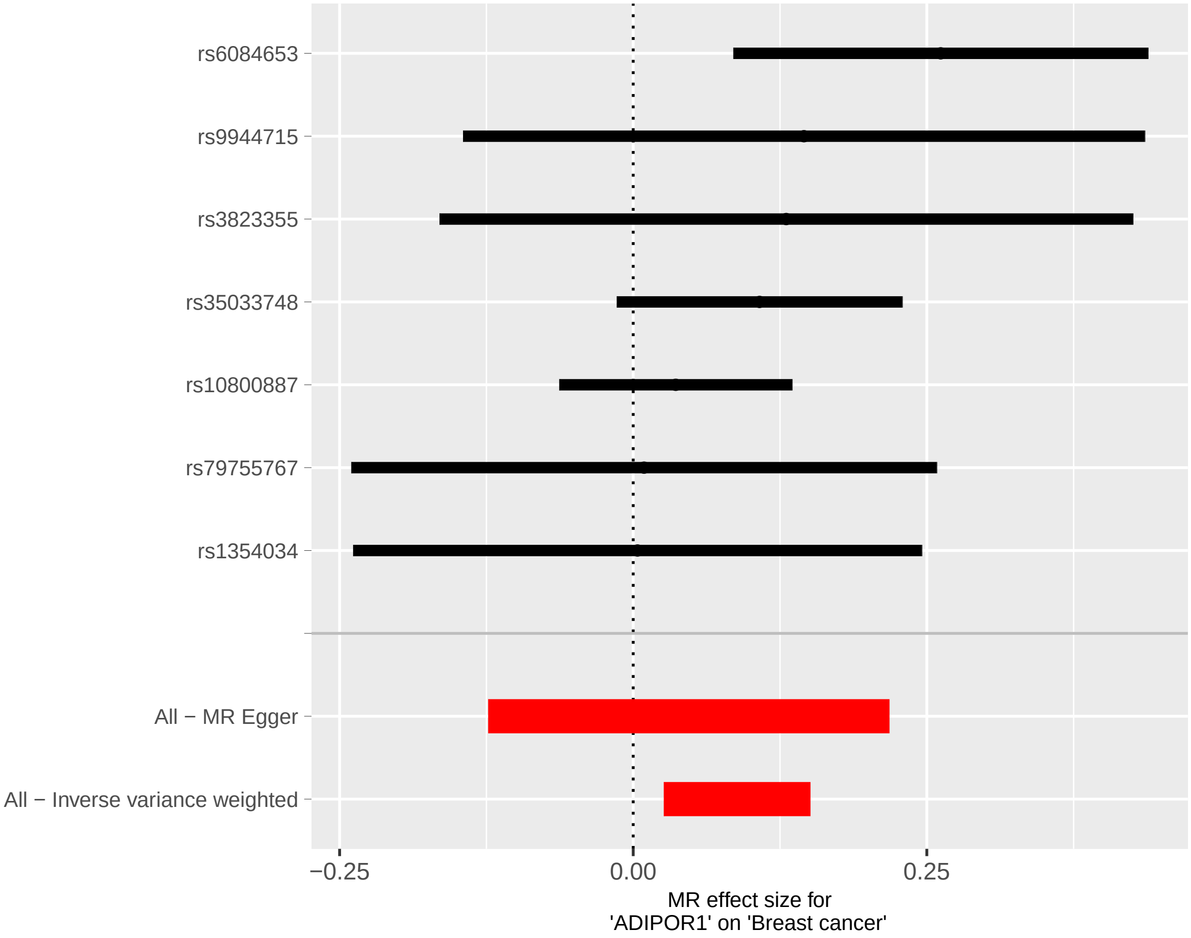

rs6084653
rs9944715
rs3823355
rs35033748
rs10800887
rs79755767
rs1354034
All − MR Egger
All − Inverse variance weighted
−0.25
0.00
0.25
MR effect size for
'ADIPOR1' on 'Breast cancer'

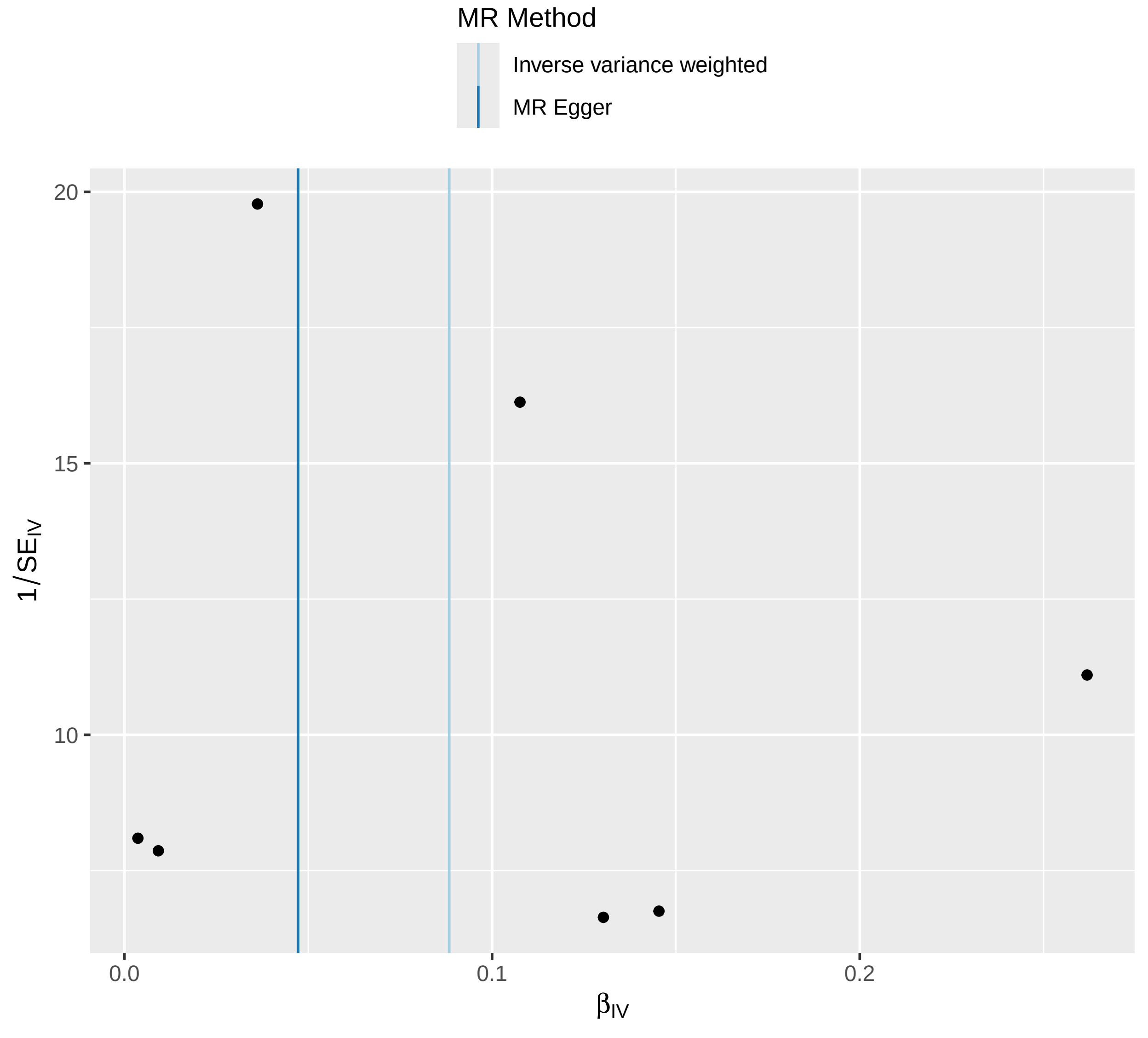

MR Method
Inverse variance weighted
MR Egger
20
15
10
0.0
0.1
0.2
1/SE_IV
β_IV

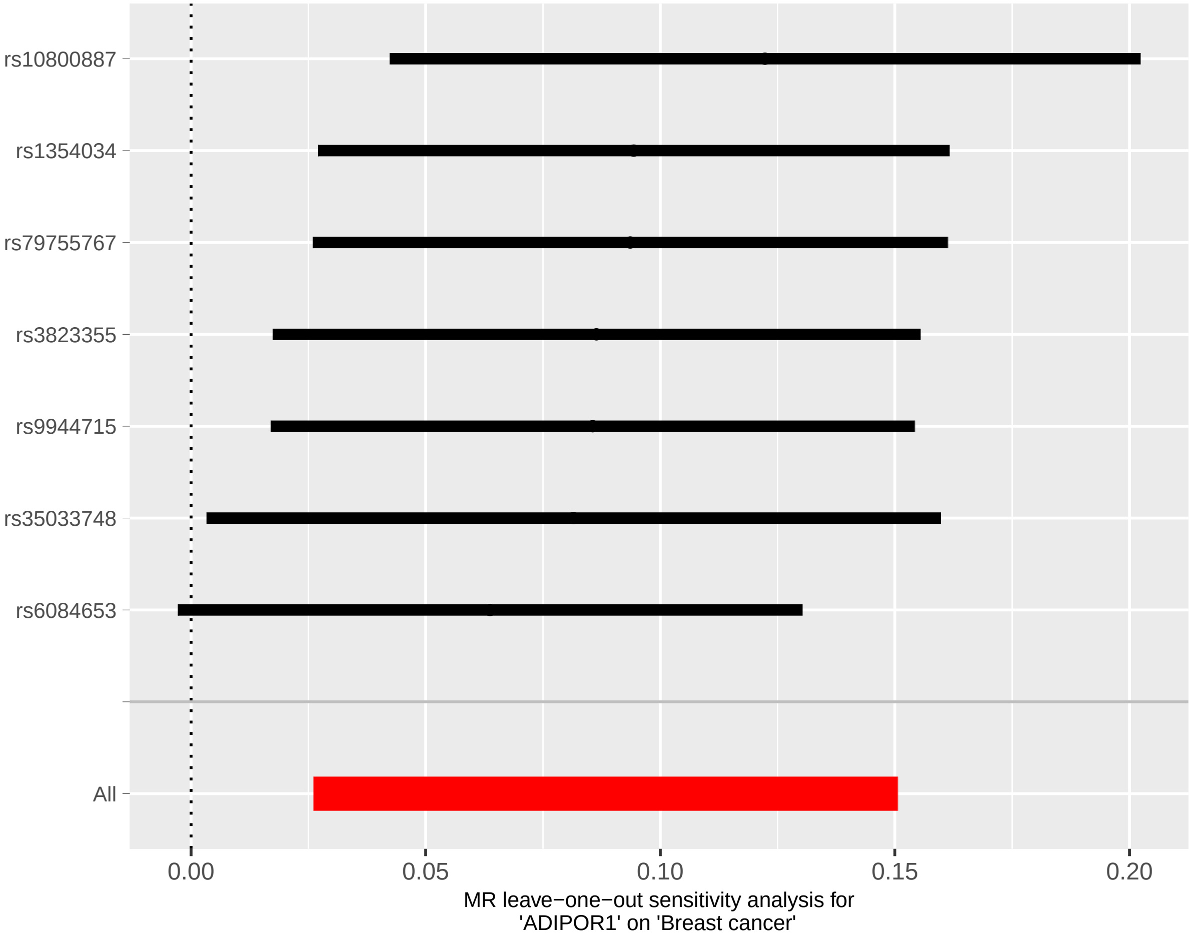

rs10800887
rs1354034
rs79755767
rs3823355
rs9944715
rs35033748
rs6084653
All
0.00
0.05
0.10
0.15
0.20
MR leave−one−out sensitivity analysis for
'ADIPOR1' on 'Breast cancer'

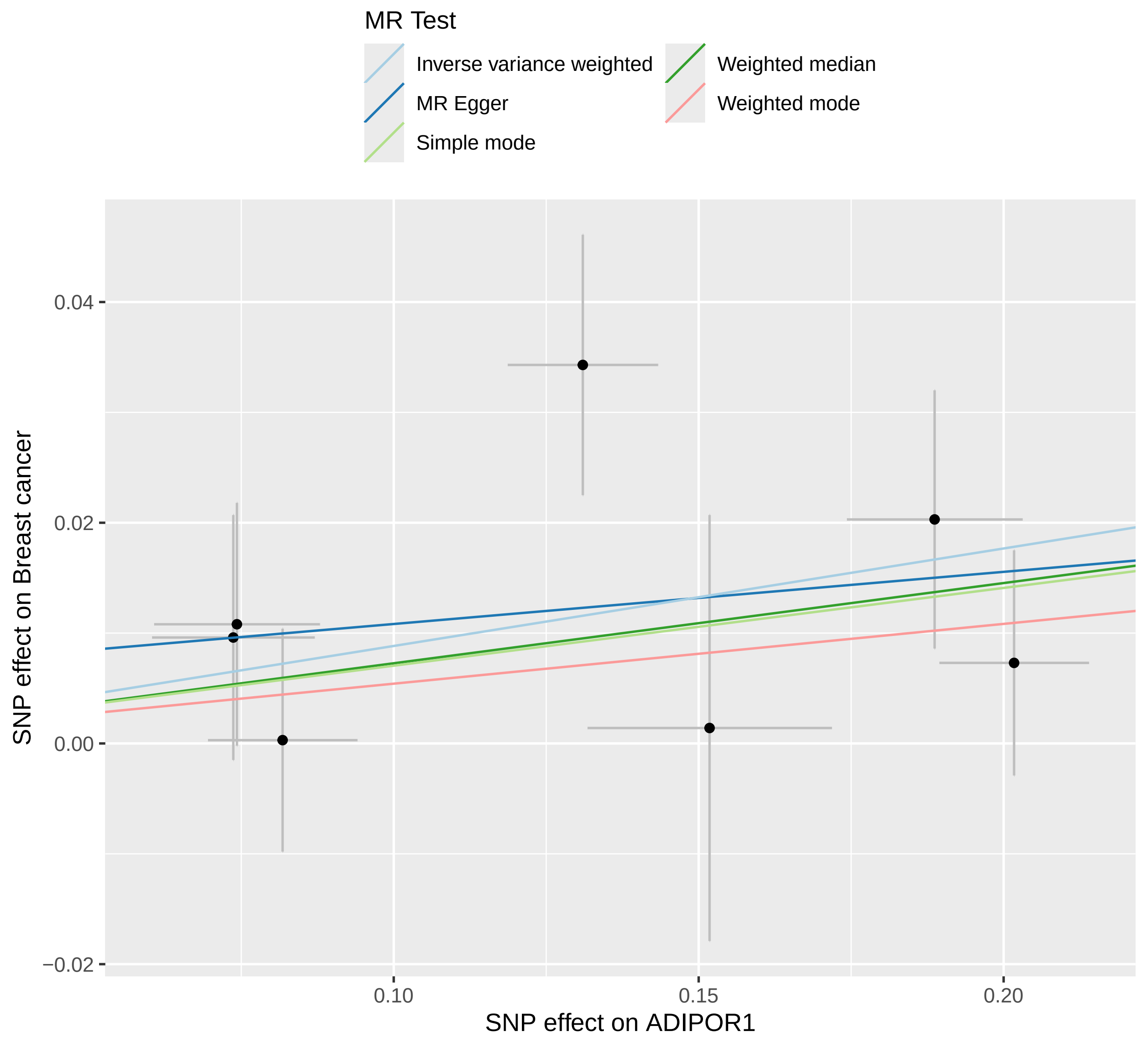
MR Test
Inverse variance weighted
MR Egger
Simple mode
Weighted median
Weighted mode
0.04
0.02
0.00
−0.02
0.10
0.15
0.20
SNP effect on Breast cancer
SNP effect on ADIPOR1

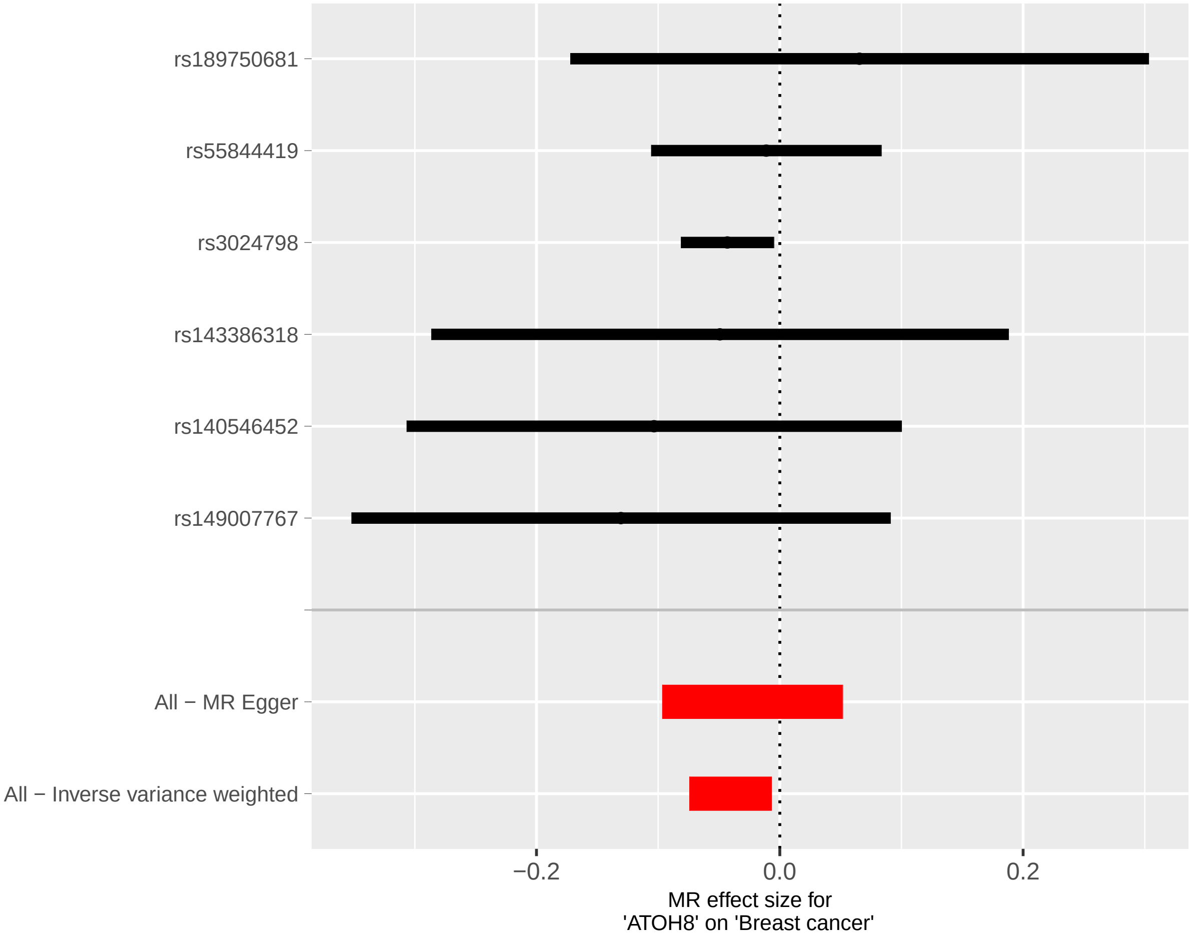
rs189750681
rs55844419
rs3024798
rs143386318
rs140546452
rs149007767
All − MR Egger
All − Inverse variance weighted
−0.2
0.0
0.2
MR effect size for
'ATOH8' on 'Breast cancer'

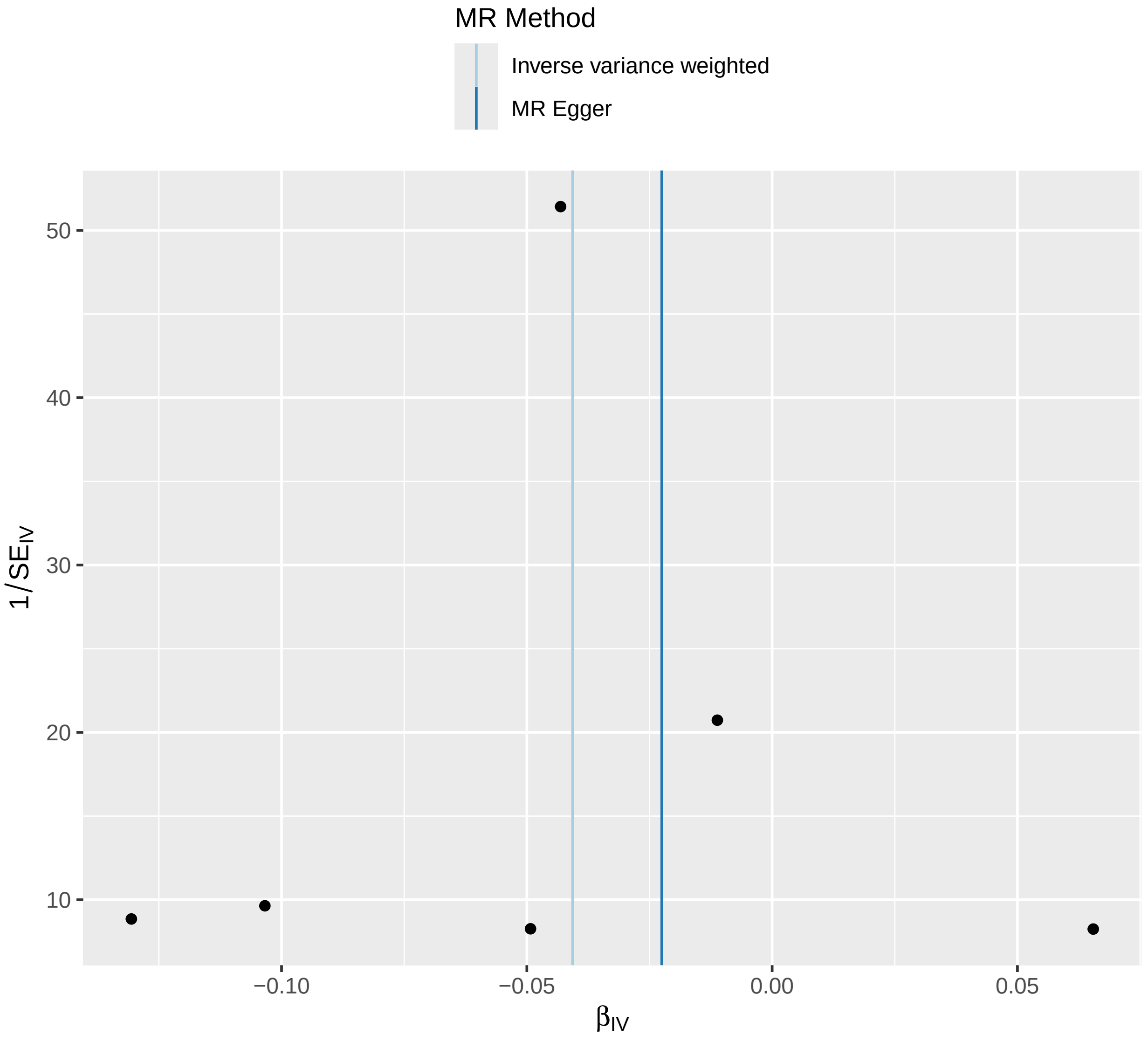
MR Method
Inverse variance weighted
MR Egger
1/SE$_{IV}$
50
40
30
20
10
−0.10
−0.05
0.00
0.05
β$_{IV}$

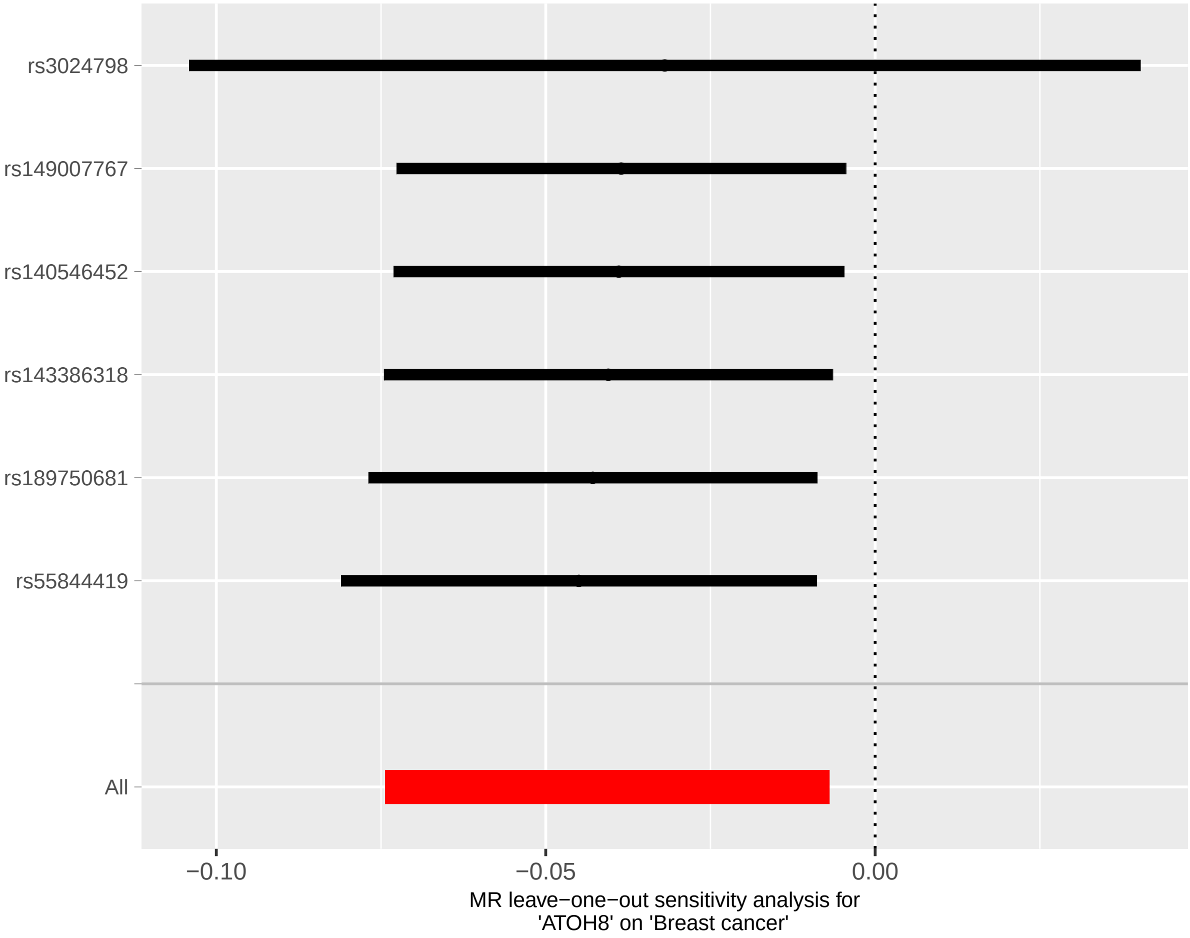
rs3024798
rs149007767
rs140546452
rs143386318
rs189750681
rs55844419
All
−0.10
−0.05
0.00
MR leave−one−out sensitivity analysis for
'ATOH8' on 'Breast cancer'

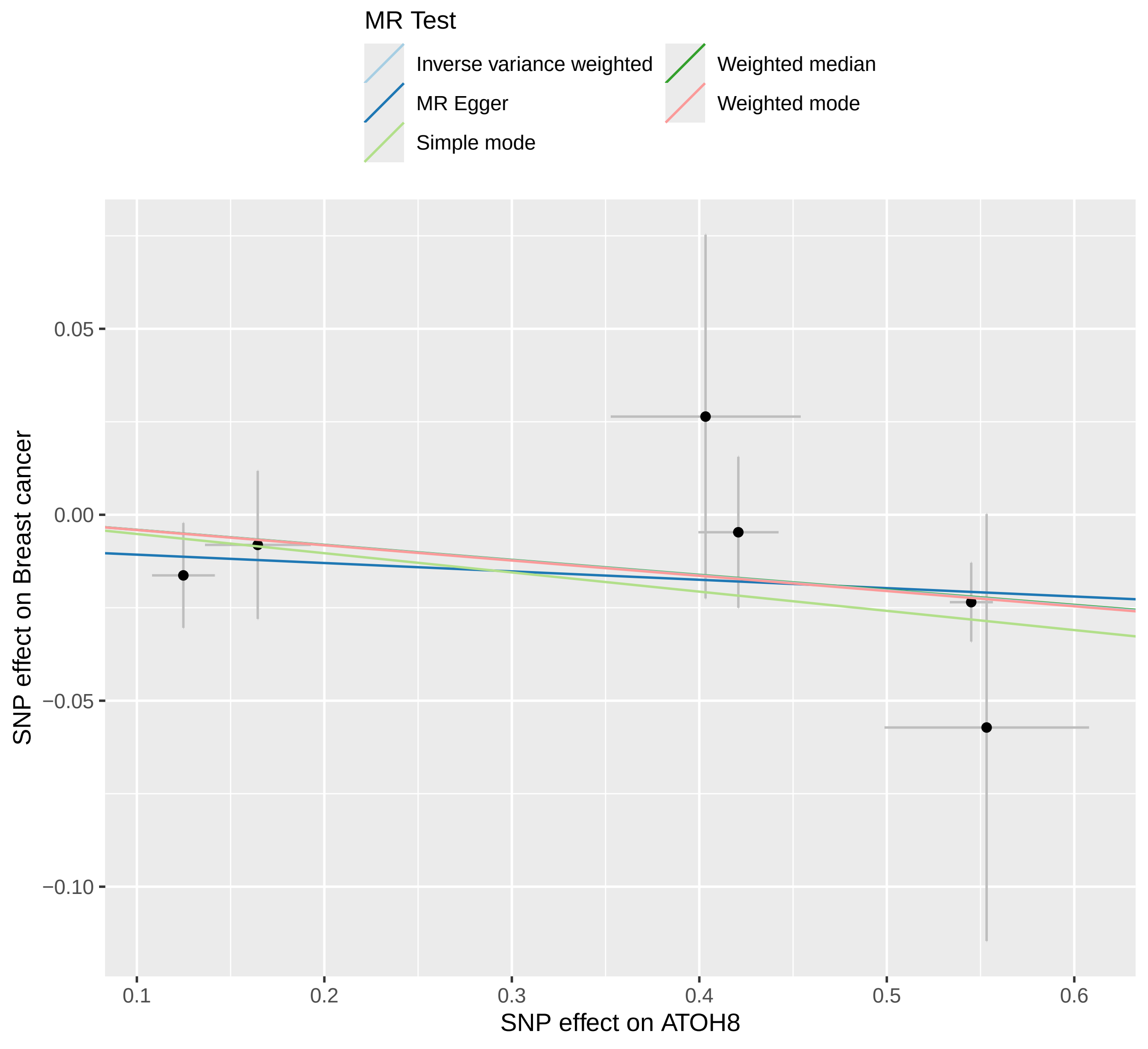
MR Test
Inverse variance weighted
MR Egger
Simple mode
Weighted median
Weighted mode
0.05
0.00
−0.05
−0.10
0.1
0.2
0.3
0.4
0.5
0.6
SNP effect on Breast cancer
SNP effect on ATOH8

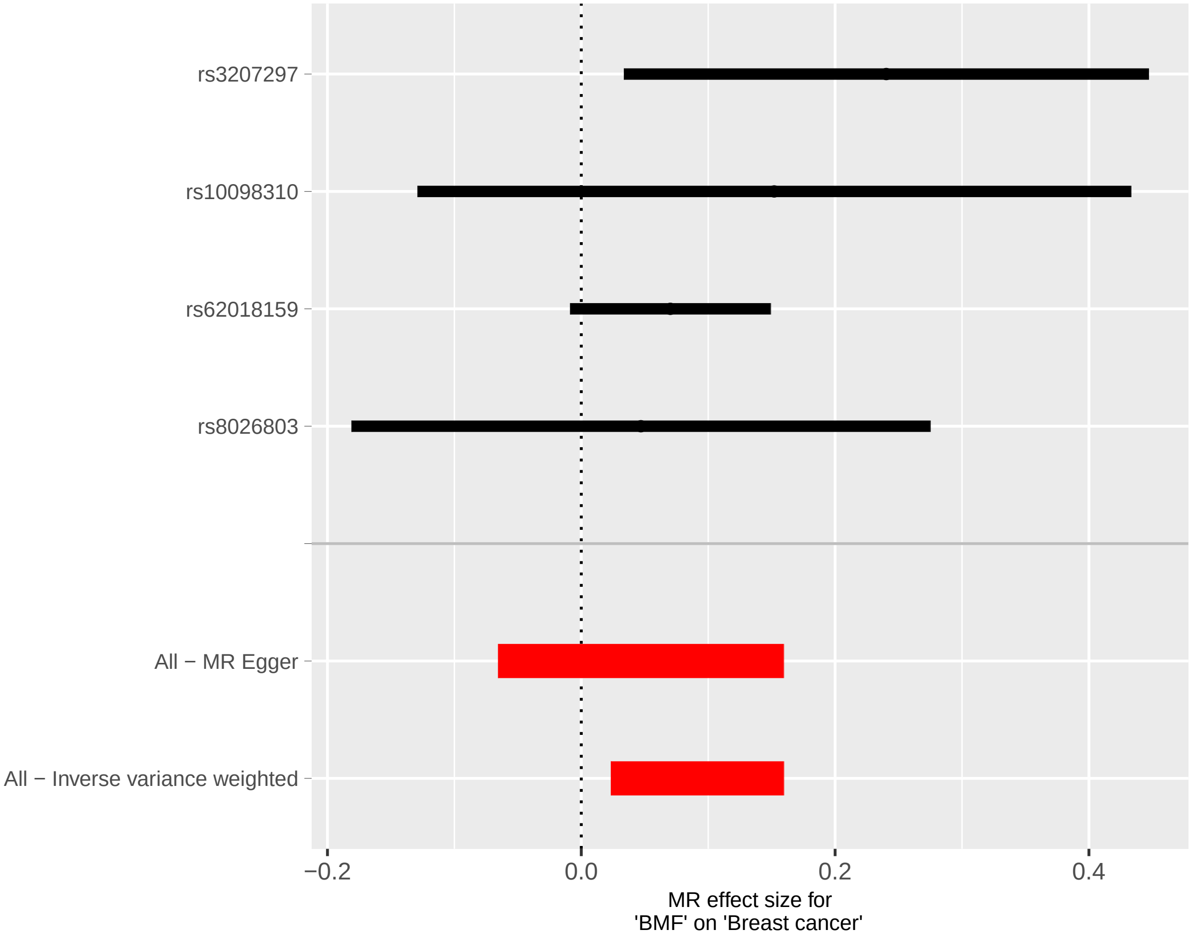

rs3207297
rs10098310
rs62018159
rs8026803
All − MR Egger
All − Inverse variance weighted
−0.2
0.0
0.2
0.4
MR effect size for
'BMF' on 'Breast cancer'

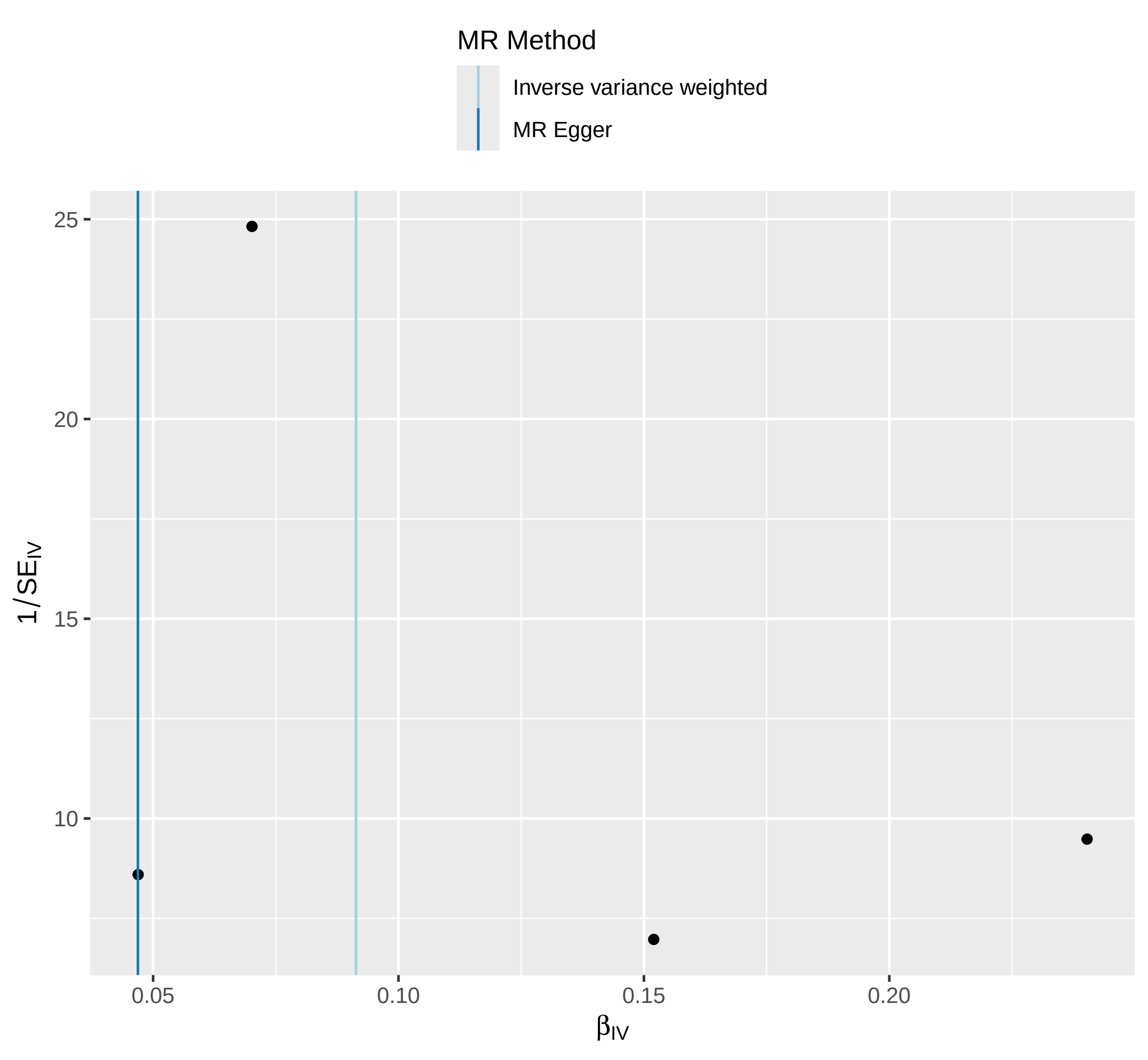

MR Method
Inverse variance weighted
MR Egger
25
20
15
10
$1/SE_{IV}$
0.05
0.10
0.15
0.20
β_{IV}

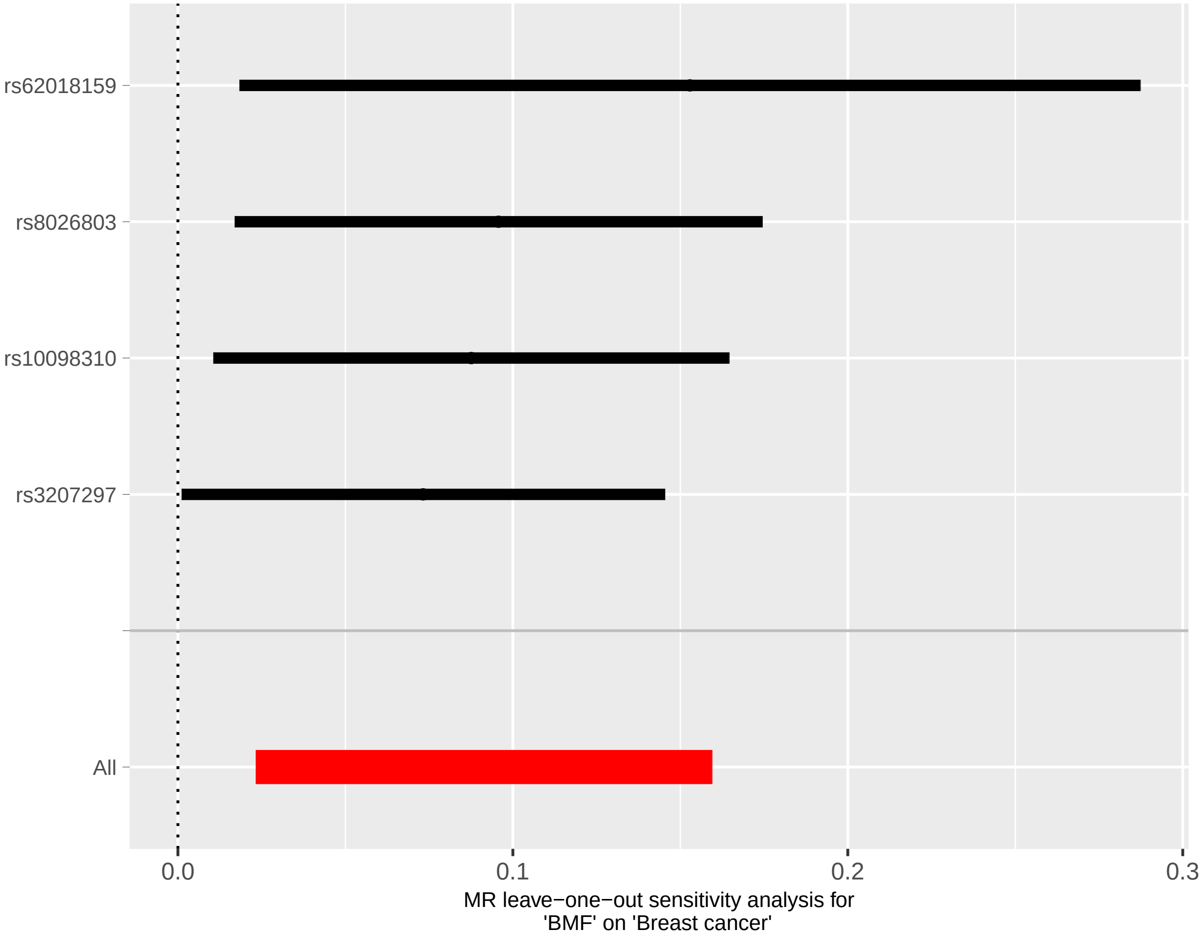
rs62018159
rs8026803
rs10098310
rs3207297
All
0.0
0.1
0.2
0.3
MR leave−one−out sensitivity analysis for
'BMF' on 'Breast cancer'

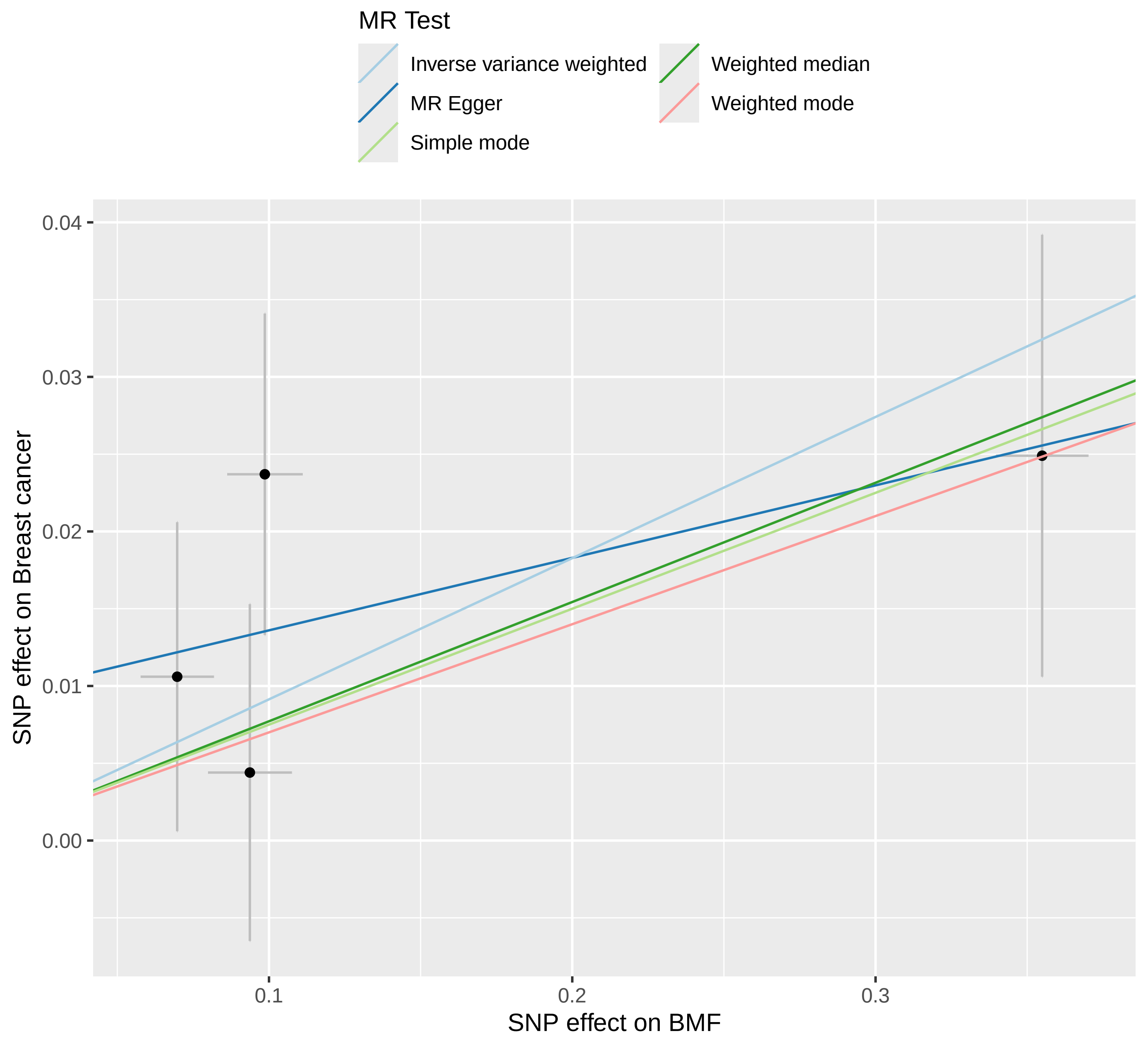

MR Test
Inverse variance weighted
MR Egger
Simple mode
Weighted median
Weighted mode
0.04
0.03
0.02
0.01
0.00
0.1
0.2
0.3
SNP effect on Breast cancer
SNP effect on BMF

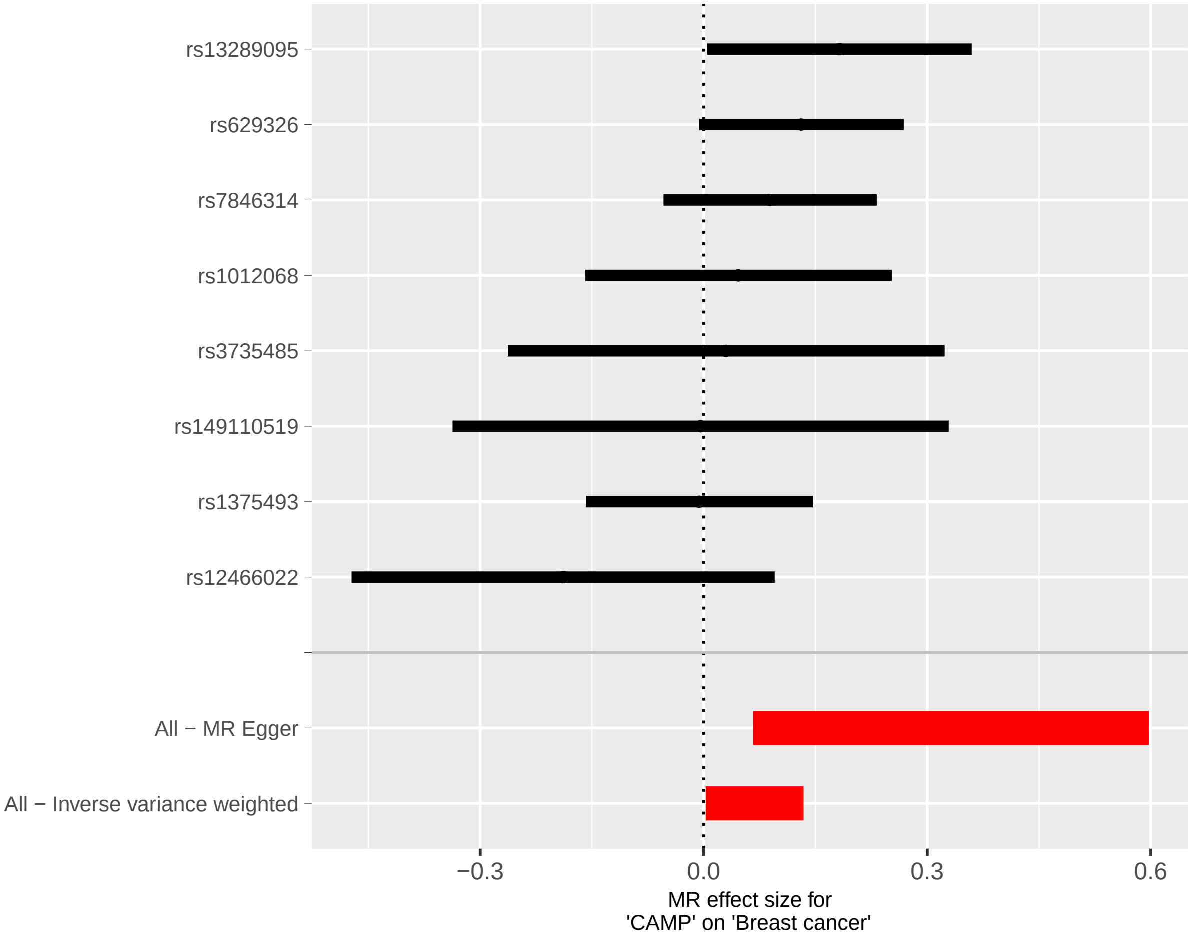
rs13289095
rs629326
rs7846314
rs1012068
rs3735485
rs149110519
rs1375493
rs12466022
All − MR Egger
All − Inverse variance weighted
−0.3
0.0
0.3
0.6
MR effect size for
'CAMP' on 'Breast cancer'

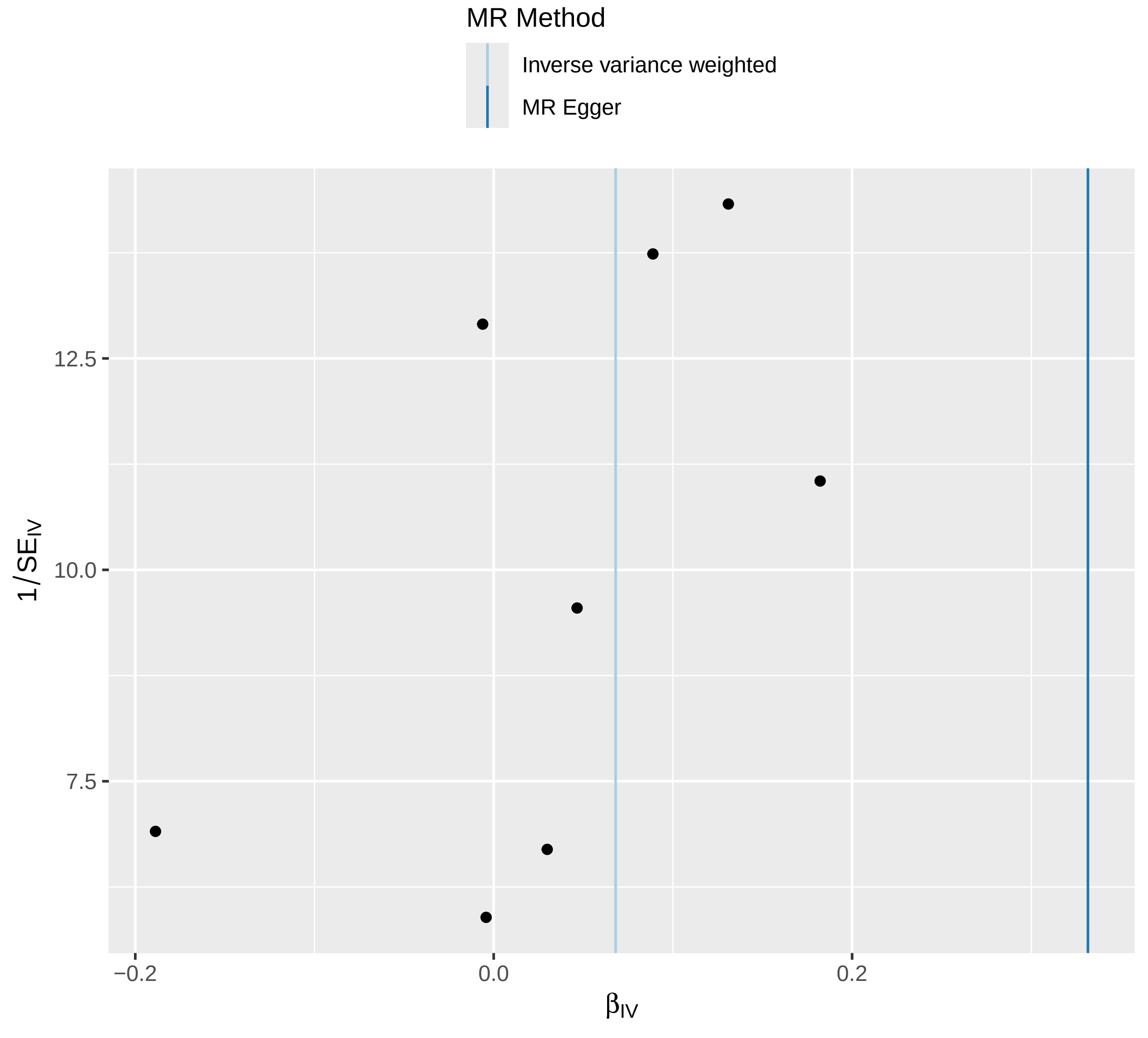

MR Method
Inverse variance weighted
MR Egger
12.5
10.0
7.5
1/SE_IV
−0.2
0.0
0.2
β_IV

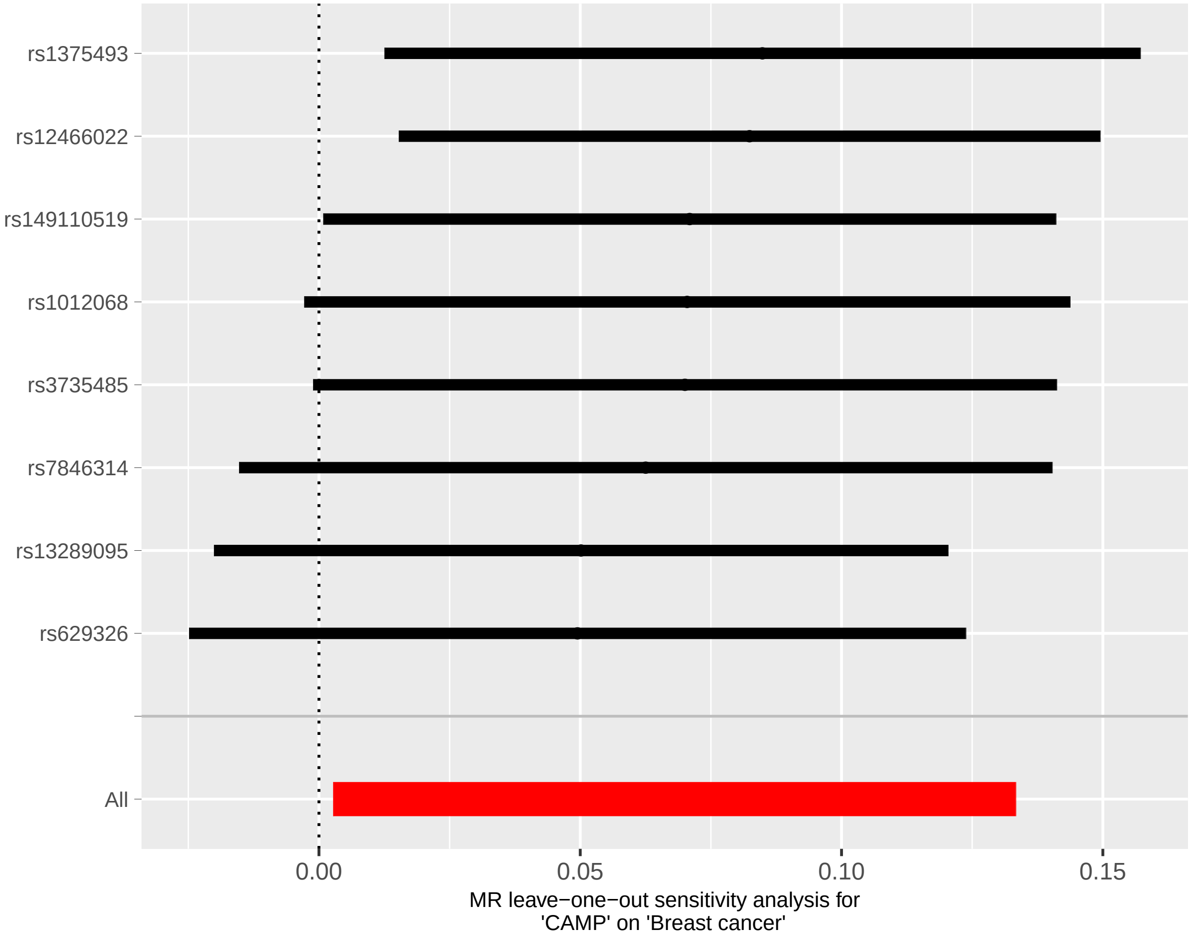
rs1375493
rs12466022
rs149110519
rs1012068
rs3735485
rs7846314
rs13289095
rs629326
All
0.00
0.05
0.10
0.15
MR leave−one−out sensitivity analysis for
'CAMP' on 'Breast cancer'

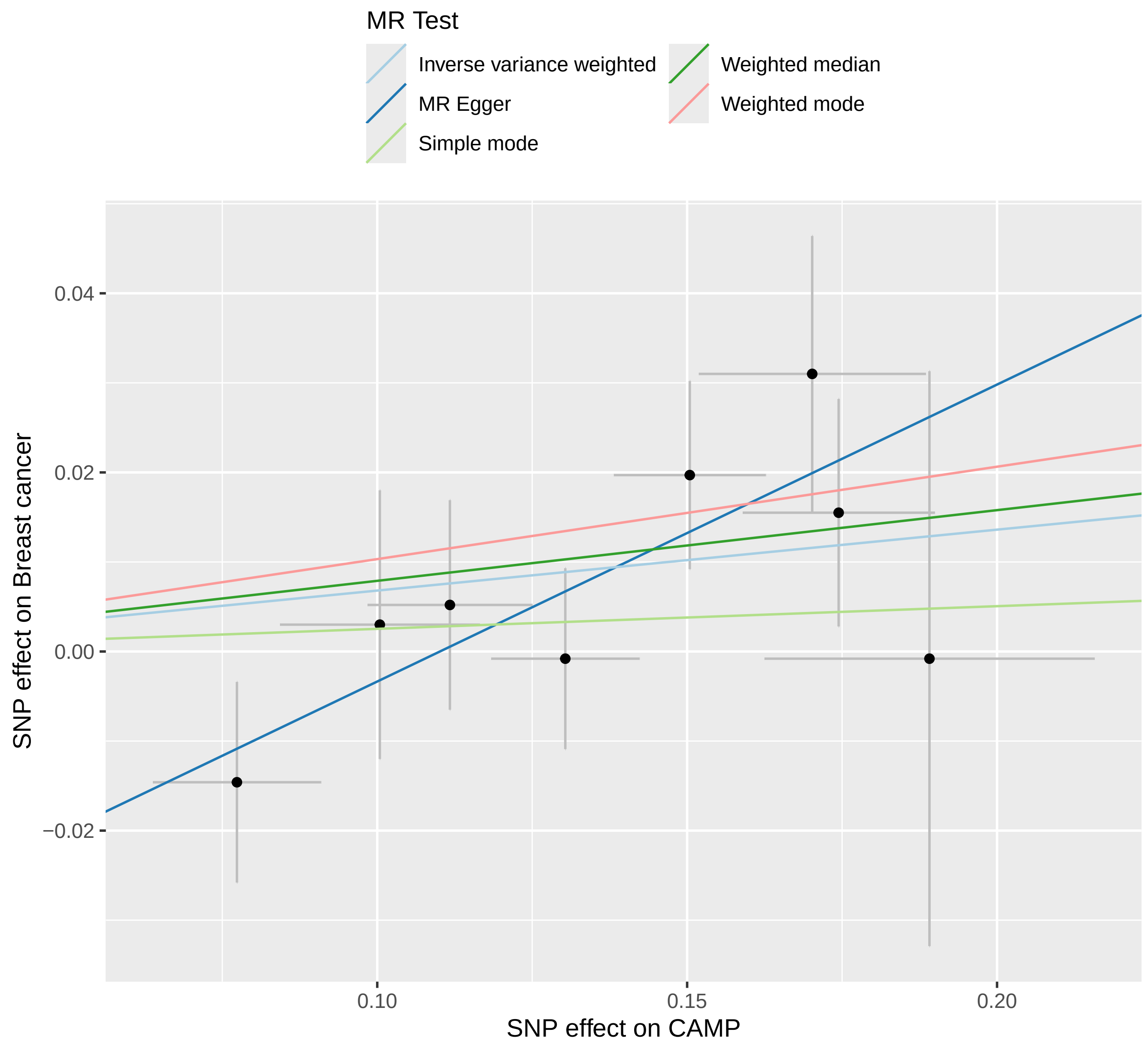
MR Test
Inverse variance weighted
MR Egger
Simple mode
Weighted median
Weighted mode
0.04
0.02
0.00
−0.02
SNP effect on Breast cancer
0.10
0.15
0.20
SNP effect on CAMP

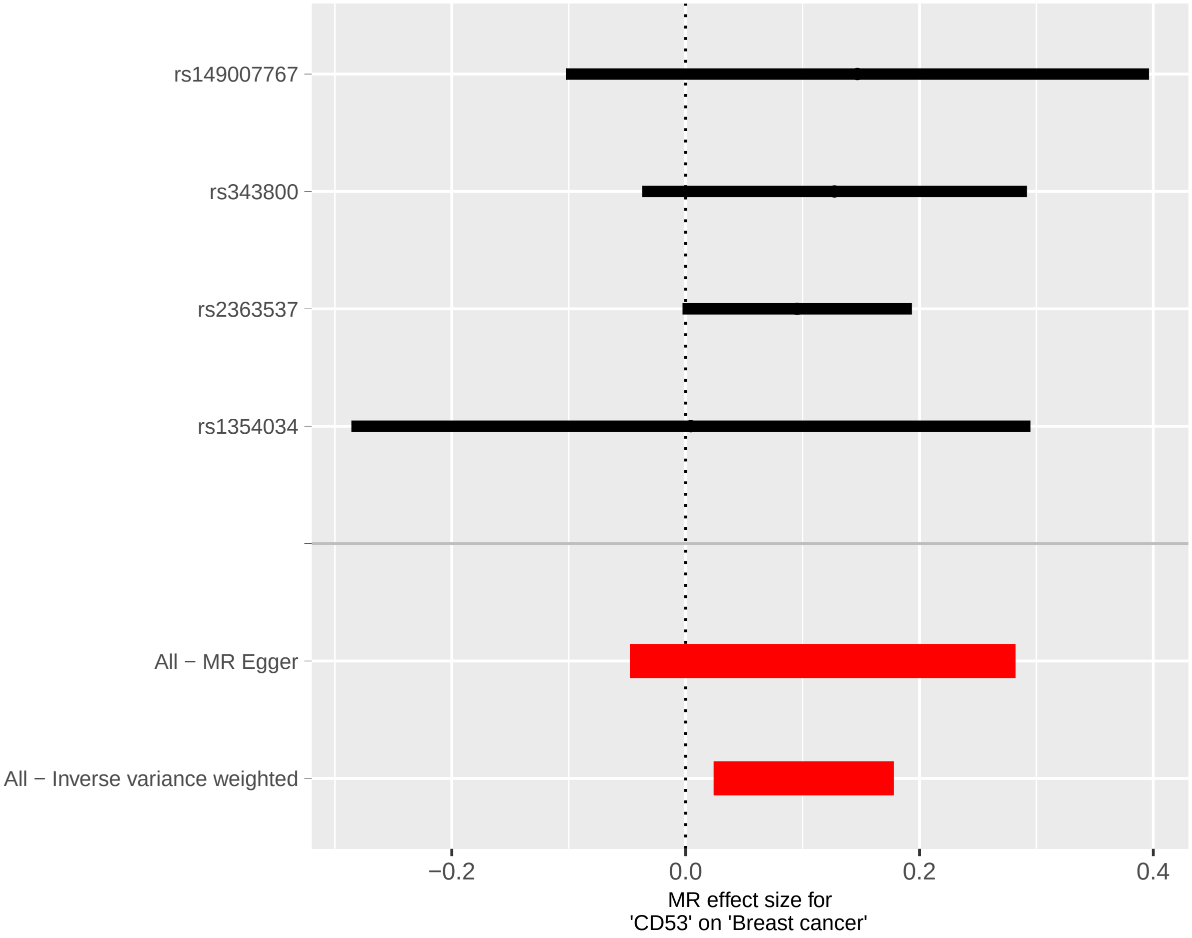

rs149007767
rs343800
rs2363537
rs1354034
All − MR Egger
All − Inverse variance weighted
−0.2
0.0
0.2
0.4
MR effect size for
'CD53' on 'Breast cancer'

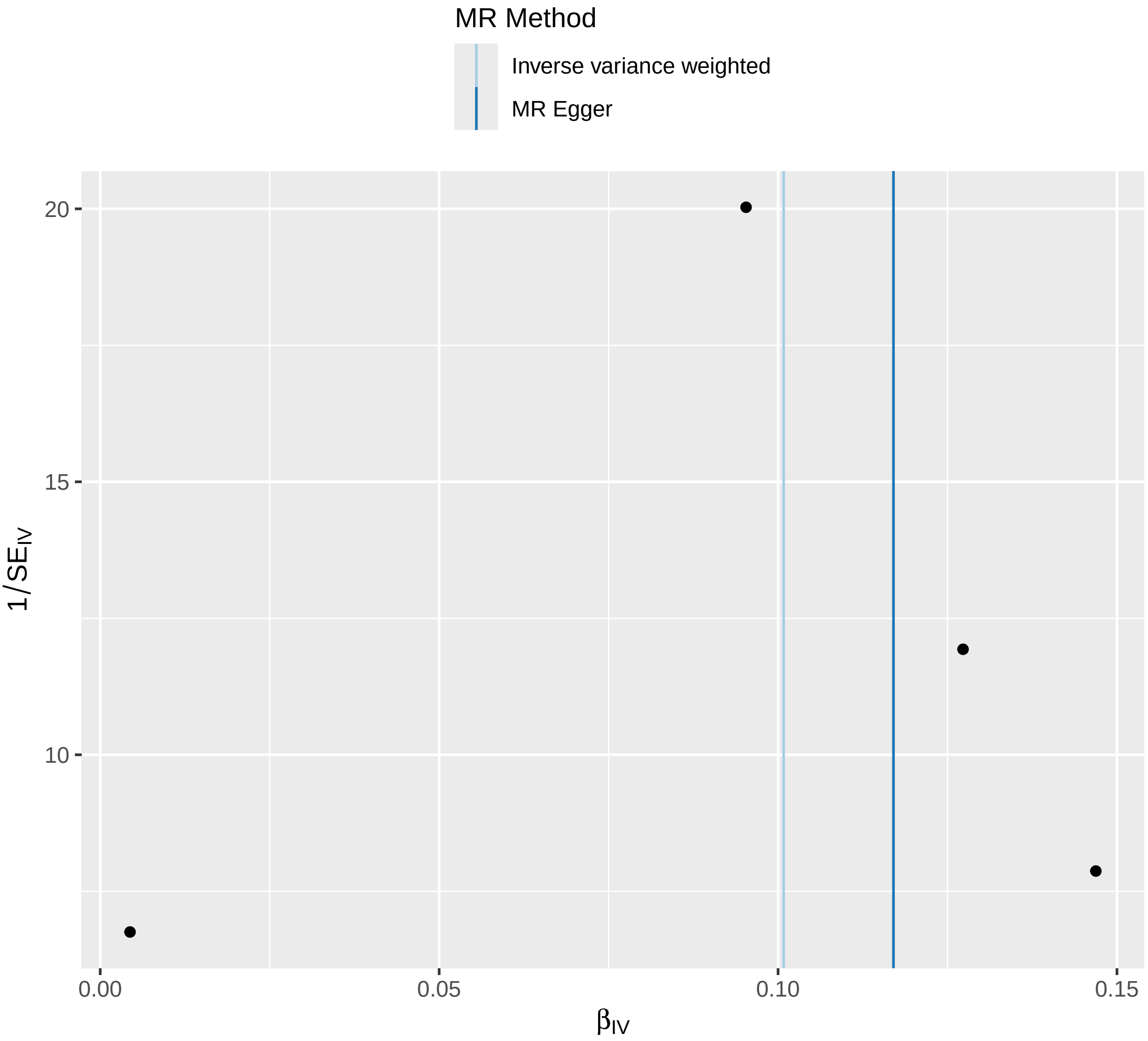
MR Method
Inverse variance weighted
MR Egger
20
15
10
1/SE$_{IV}$
0.00
0.05
0.10
0.15
β_{IV}

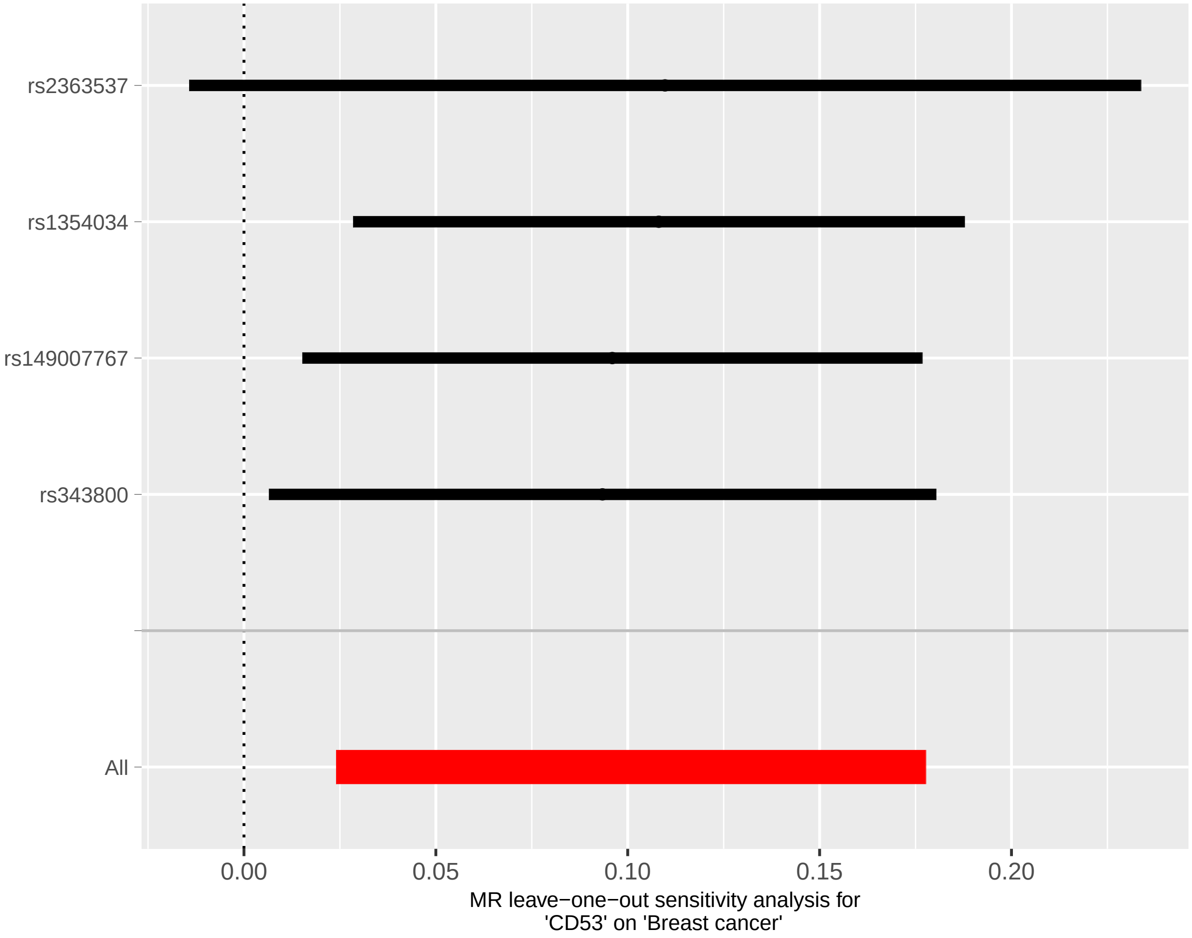
rs2363537
rs1354034
rs149007767
rs343800
All
0.00
0.05
0.10
0.15
0.20
MR leave−one−out sensitivity analysis for
'CD53' on 'Breast cancer'

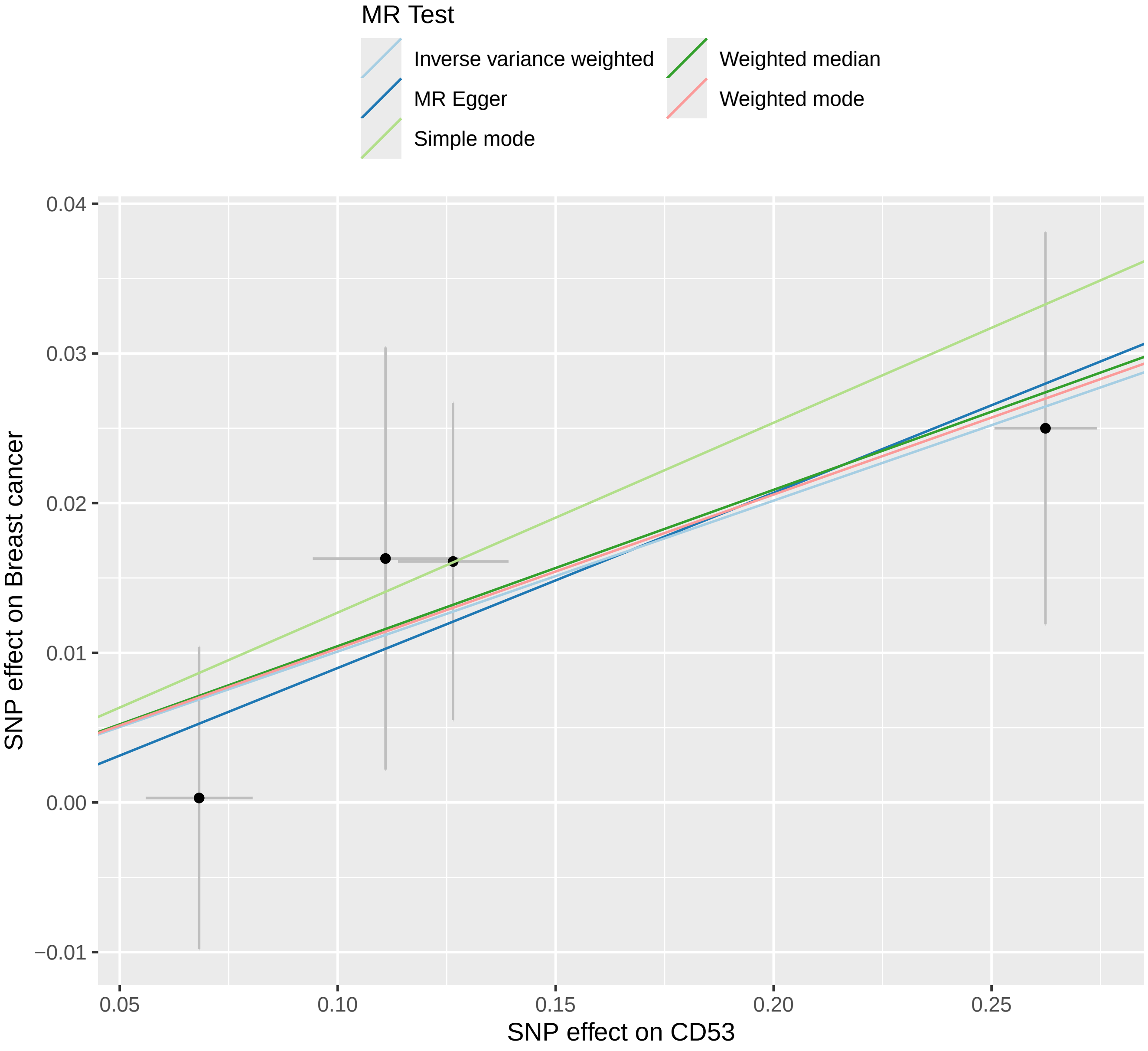
MR Test
Inverse variance weighted
MR Egger
Simple mode
Weighted median
Weighted mode
0.04
0.03
0.02
0.01
0.00
−0.01
0.05
0.10
0.15
0.20
0.25
SNP effect on Breast cancer
SNP effect on CD53

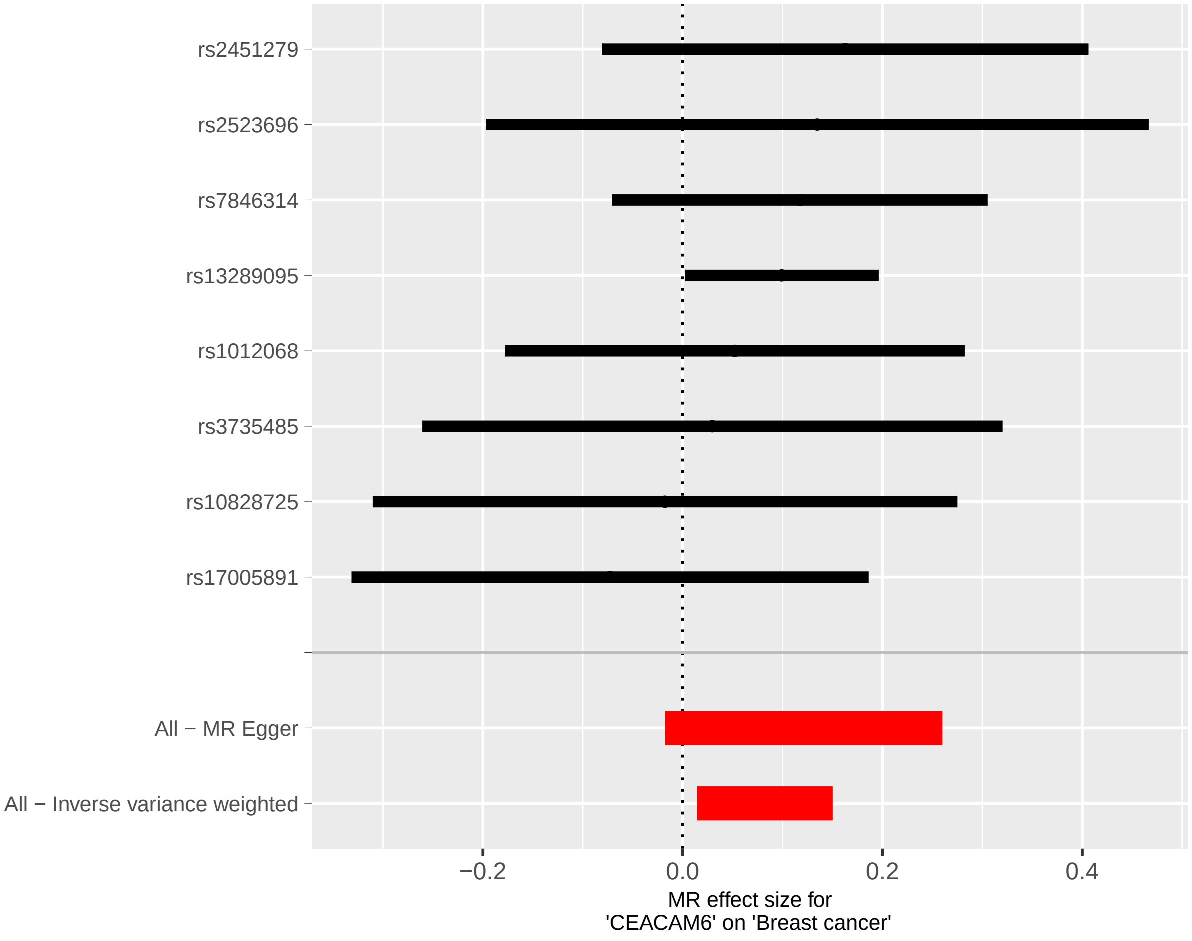
rs2451279
rs2523696
rs7846314
rs13289095
rs1012068
rs3735485
rs10828725
rs17005891
All − MR Egger
All − Inverse variance weighted
−0.2
0.0
0.2
0.4
MR effect size for
'CEACAM6' on 'Breast cancer'

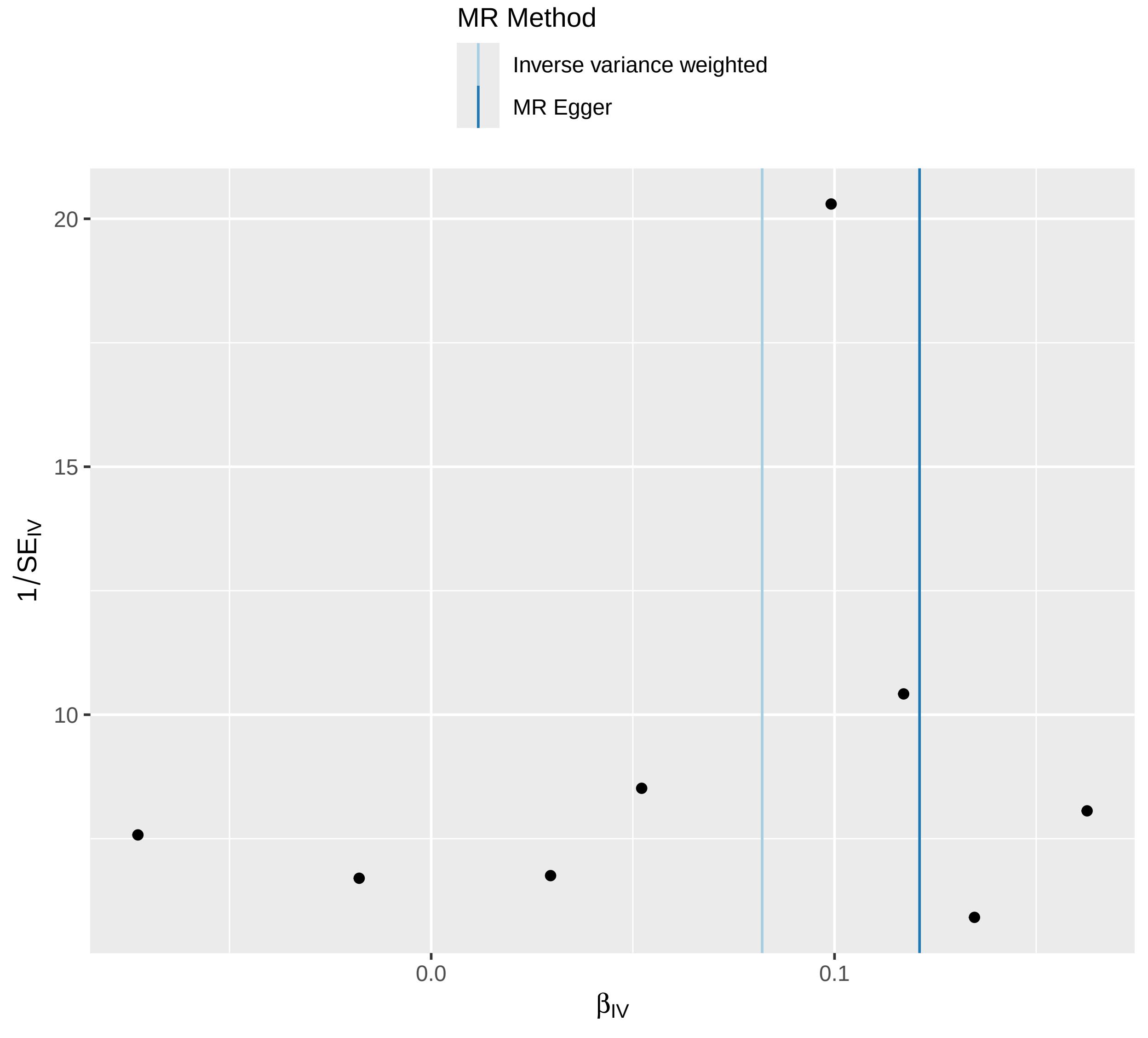
MR Method
Inverse variance weighted
MR Egger
20
15
10
0.0
0.1
1/SE_IV
β_IV

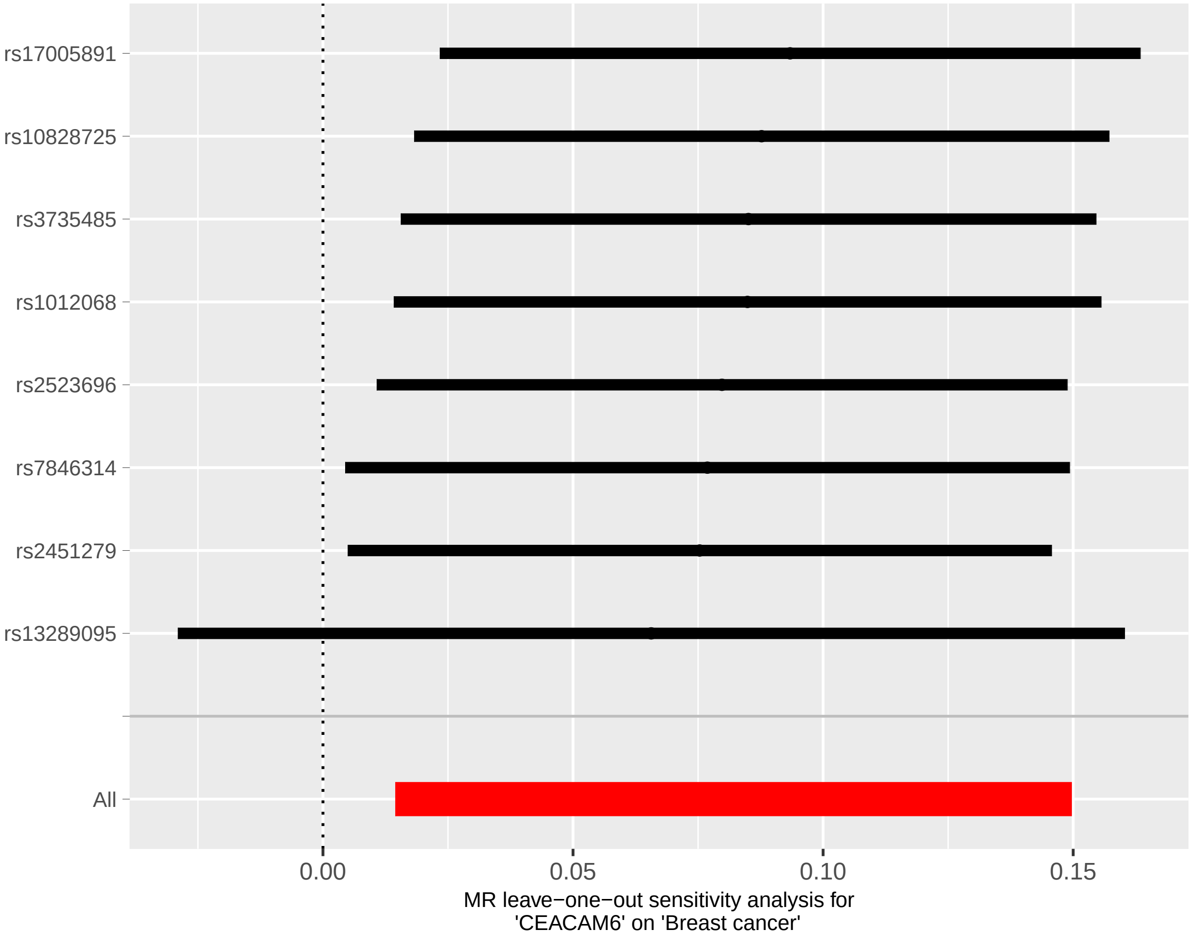
rs17005891
rs10828725
rs3735485
rs1012068
rs2523696
rs7846314
rs2451279
rs13289095
All
0.00
0.05
0.10
0.15
MR leave−one−out sensitivity analysis for
'CEACAM6' on 'Breast cancer'

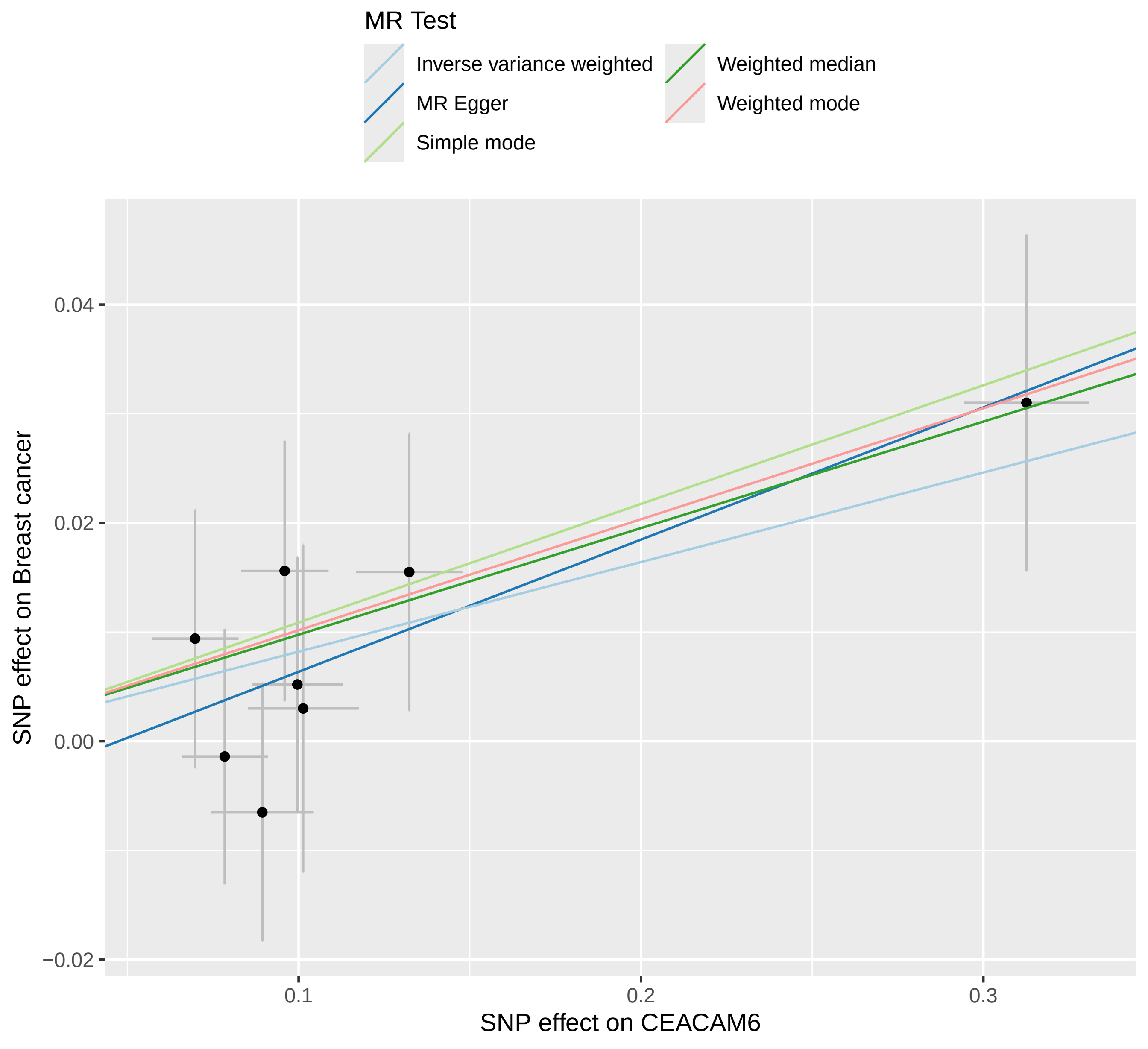
MR Test
Inverse variance weighted
MR Egger
Simple mode
Weighted median
Weighted mode
0.04
0.02
0.00
−0.02
0.1
0.2
0.3
SNP effect on Breast cancer
SNP effect on CEACAM6

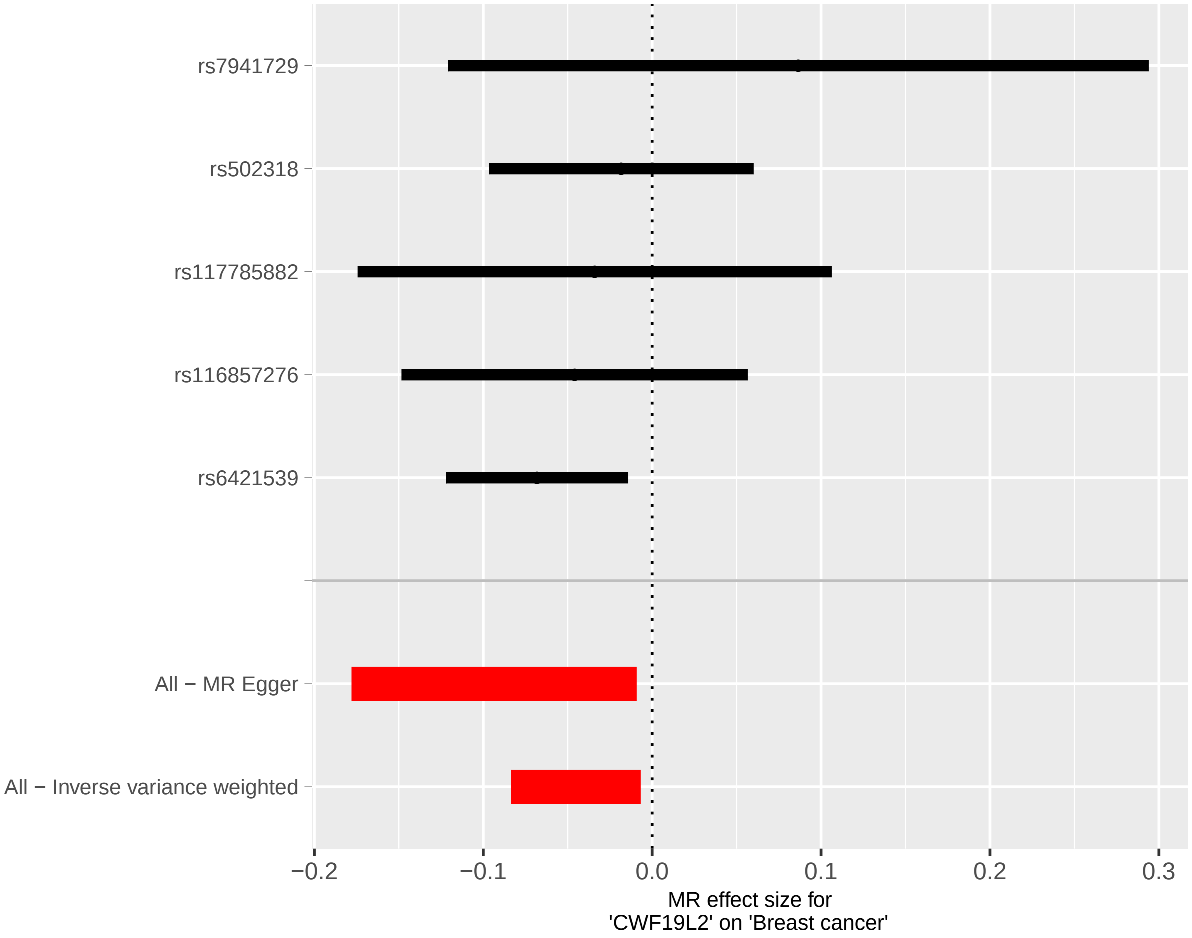

rs7941729
rs502318
rs117785882
rs116857276
rs6421539
All − MR Egger
All − Inverse variance weighted
−0.2
−0.1
0.0
0.1
0.2
0.3
MR effect size for
'CWF19L2' on 'Breast cancer'

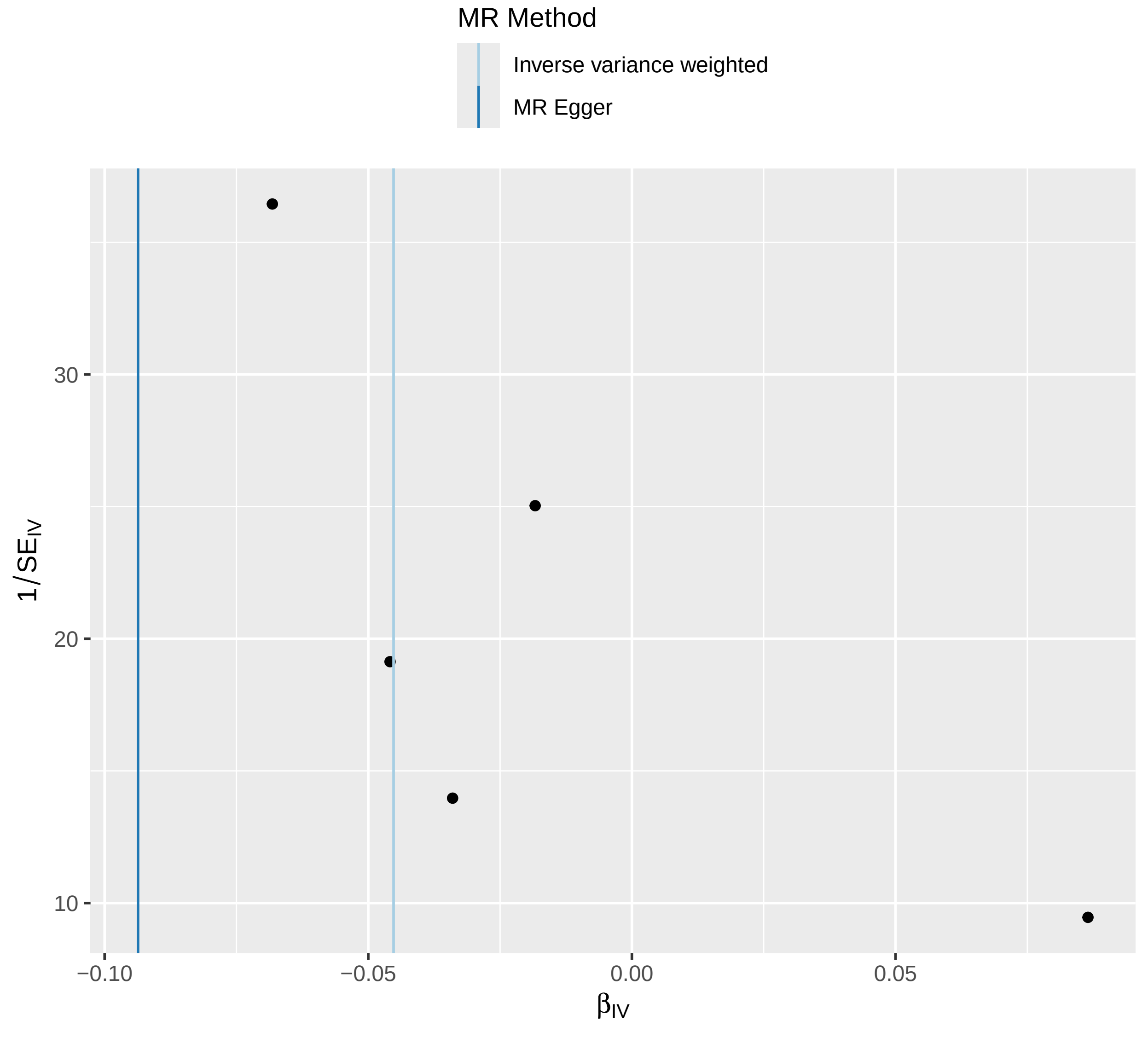
MR Method
Inverse variance weighted
MR Egger
30
20
10
$1/SE_{IV}$
−0.10
−0.05
0.00
0.05
β_{IV}

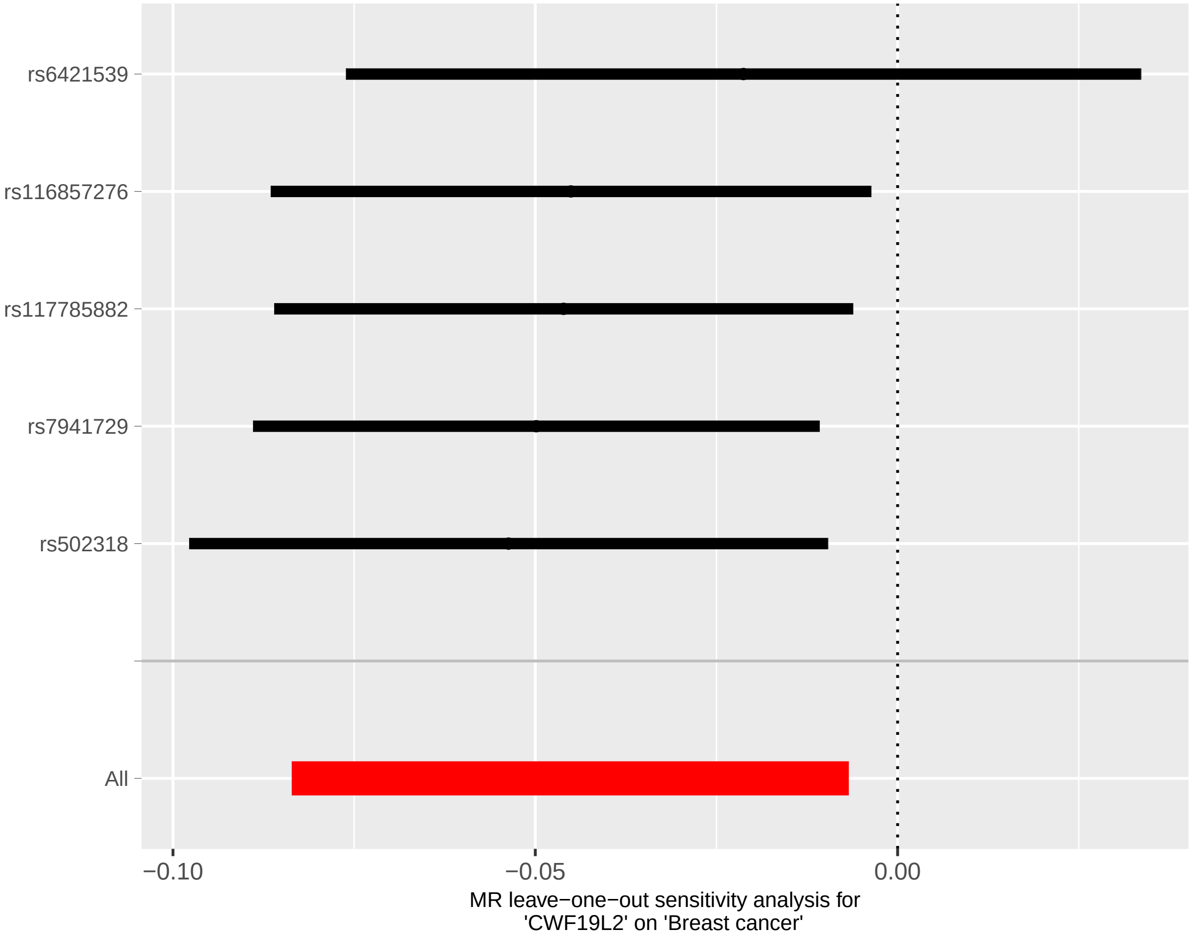
rs6421539
rs116857276
rs117785882
rs7941729
rs502318
All
−0.10
−0.05
0.00
MR leave−one−out sensitivity analysis for
'CWF19L2' on 'Breast cancer'

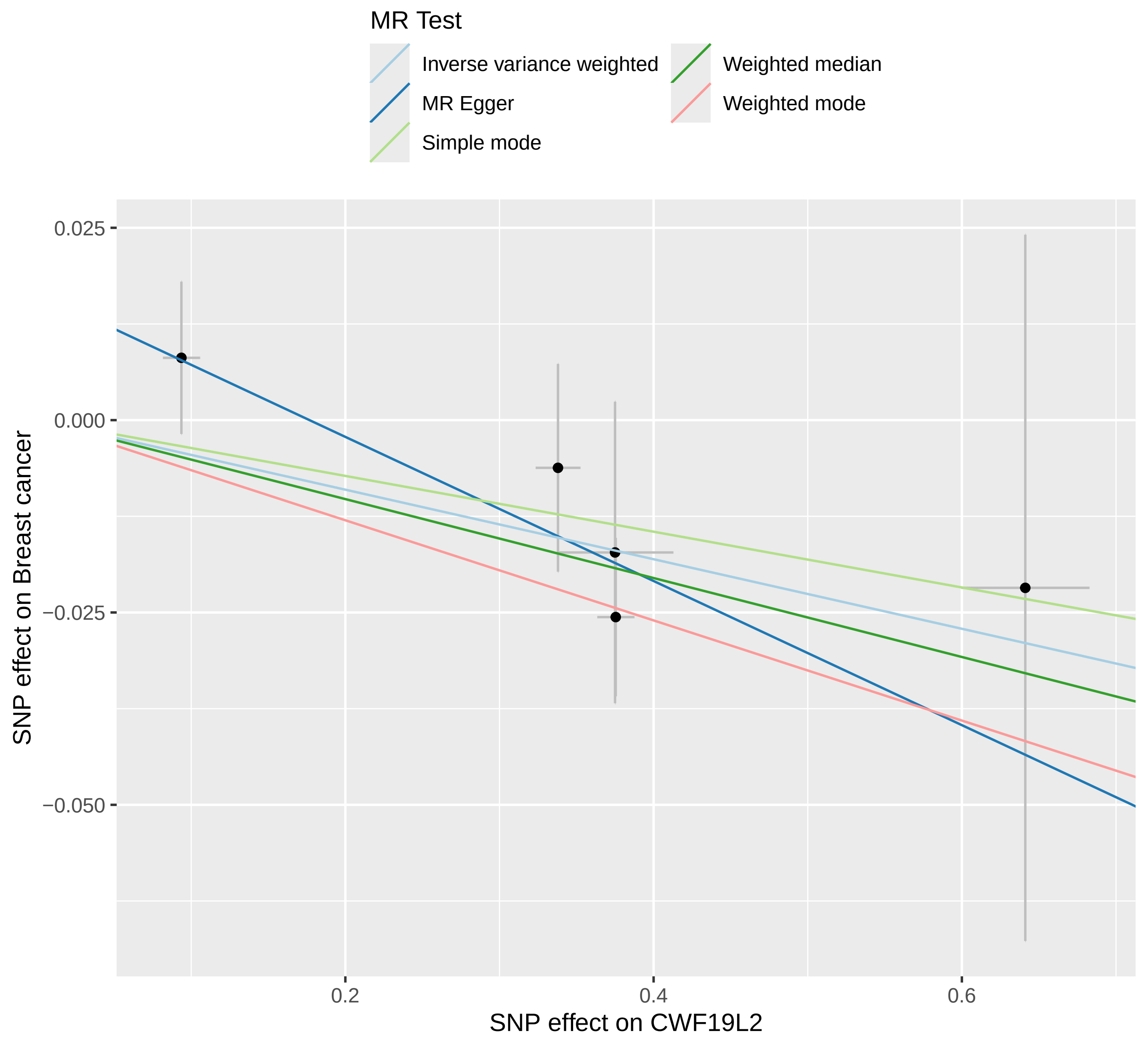
MR Test
Inverse variance weighted
MR Egger
Simple mode
Weighted median
Weighted mode
0.025
0.000
−0.025
−0.050
SNP effect on Breast cancer
0.2
0.4
0.6
SNP effect on CWF19L2

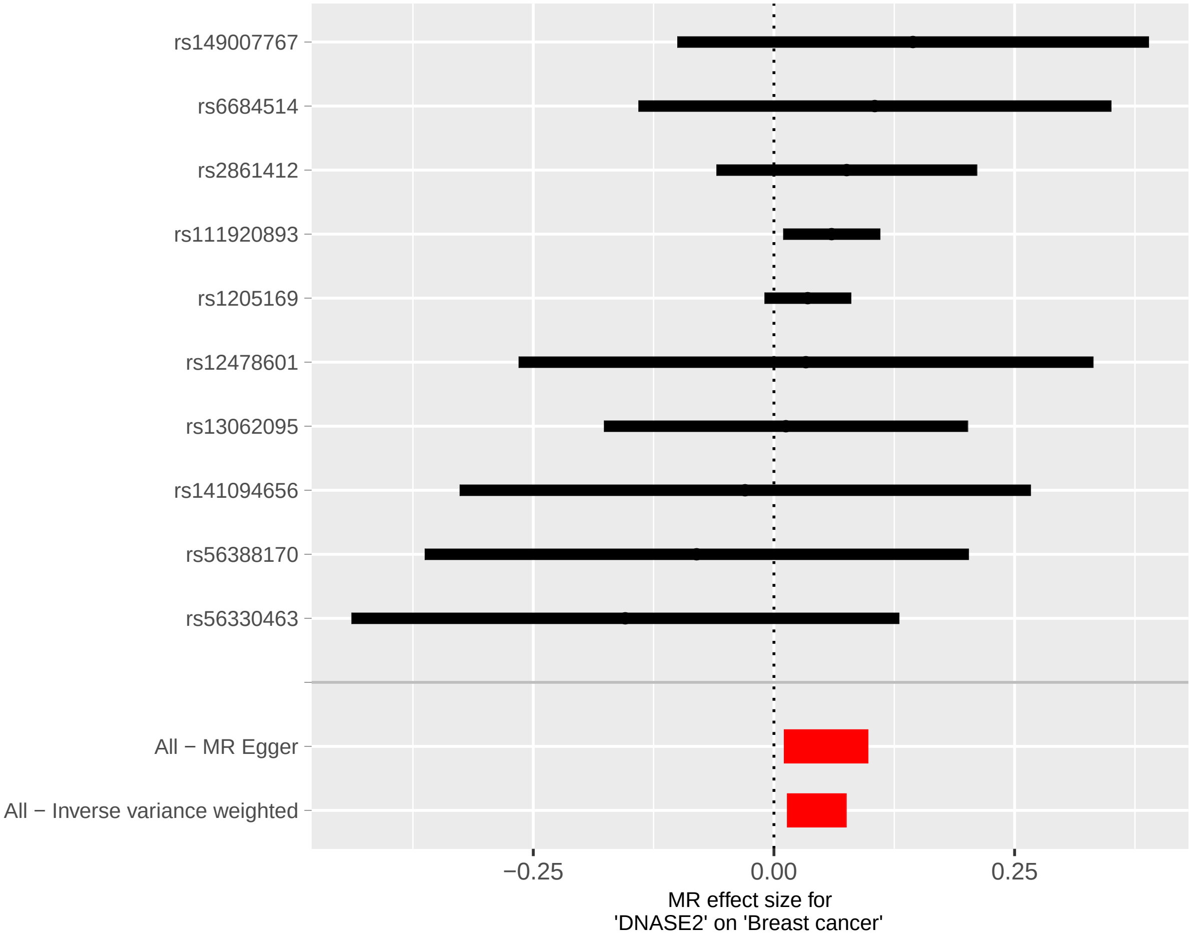
rs149007767
rs6684514
rs2861412
rs111920893
rs1205169
rs12478601
rs13062095
rs141094656
rs56388170
rs56330463
All − MR Egger
All − Inverse variance weighted
−0.25
0.00
0.25
MR effect size for
'DNASE2' on 'Breast cancer'

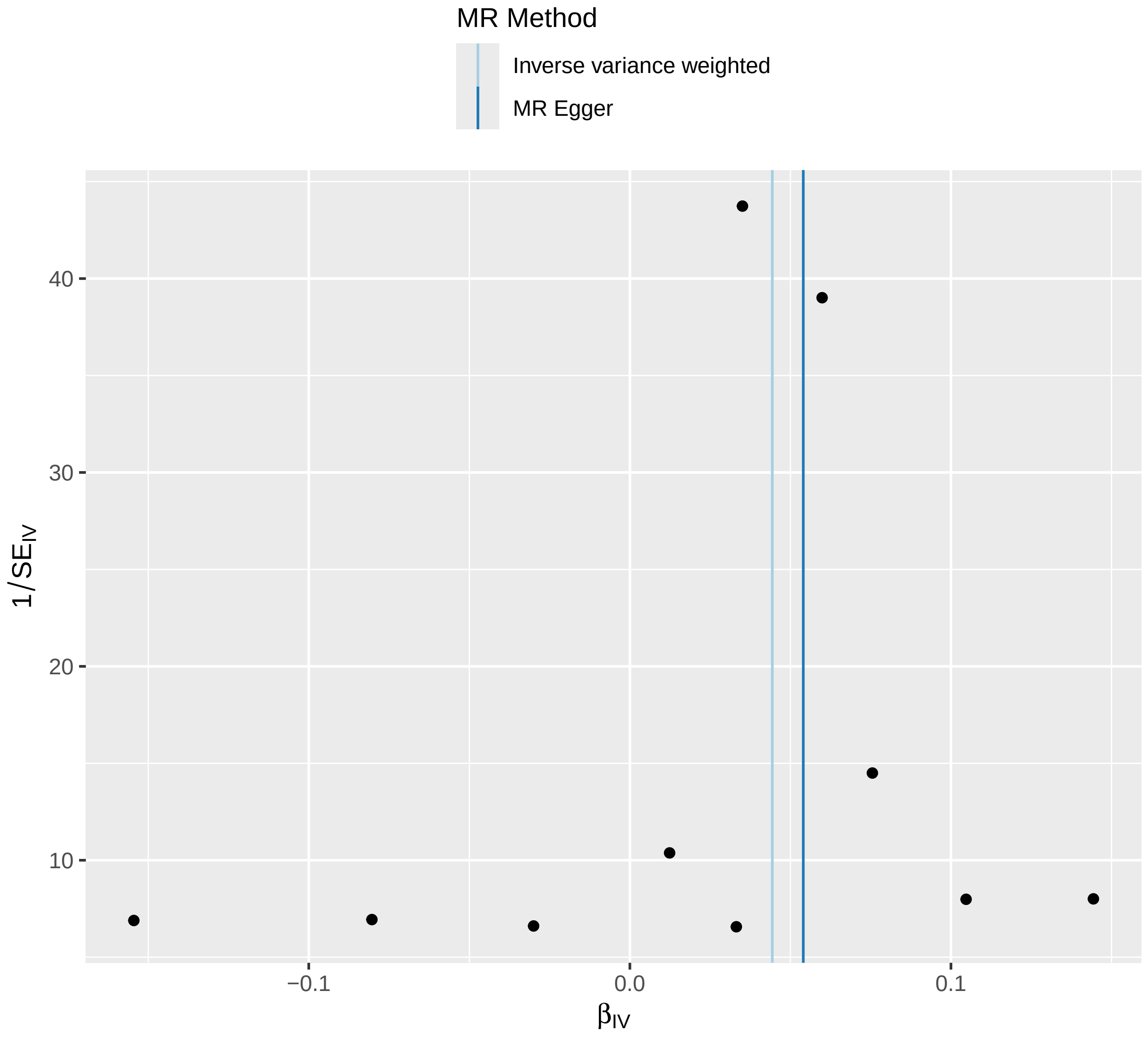

MR Method
Inverse variance weighted
MR Egger
40
30
20
10
1/SE_IV
−0.1
0.0
0.1
β_IV

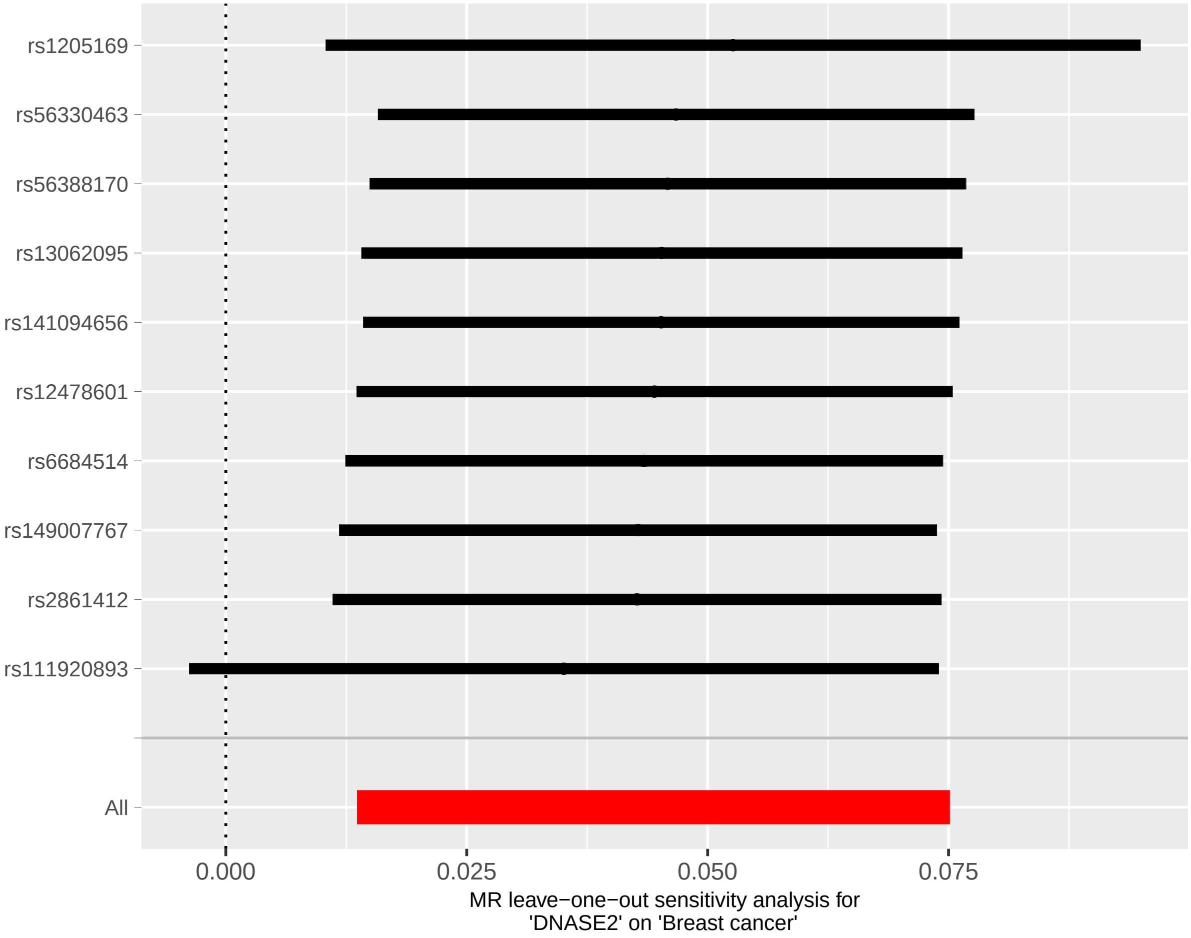
rs1205169
rs56330463
rs56388170
rs13062095
rs141094656
rs12478601
rs6684514
rs149007767
rs2861412
rs111920893
All
0.000
0.025
0.050
0.075
MR leave−one−out sensitivity analysis for
'DNASE2' on 'Breast cancer'

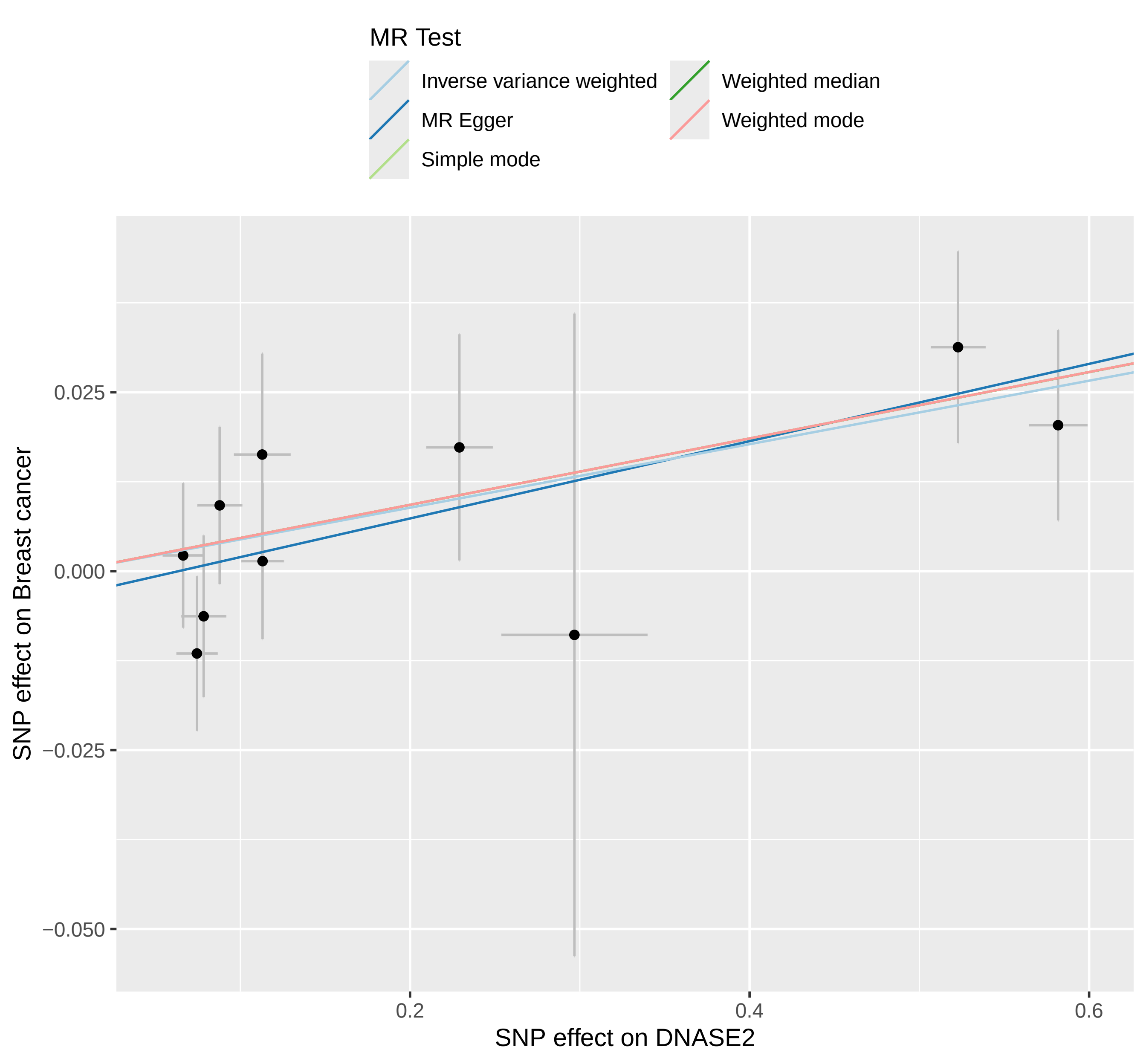

MR Test
Inverse variance weighted
MR Egger
Simple mode
Weighted median
Weighted mode
0.025
0.000
−0.025
−0.050
0.2
0.4
0.6
SNP effect on DNASE2
SNP effect on Breast cancer

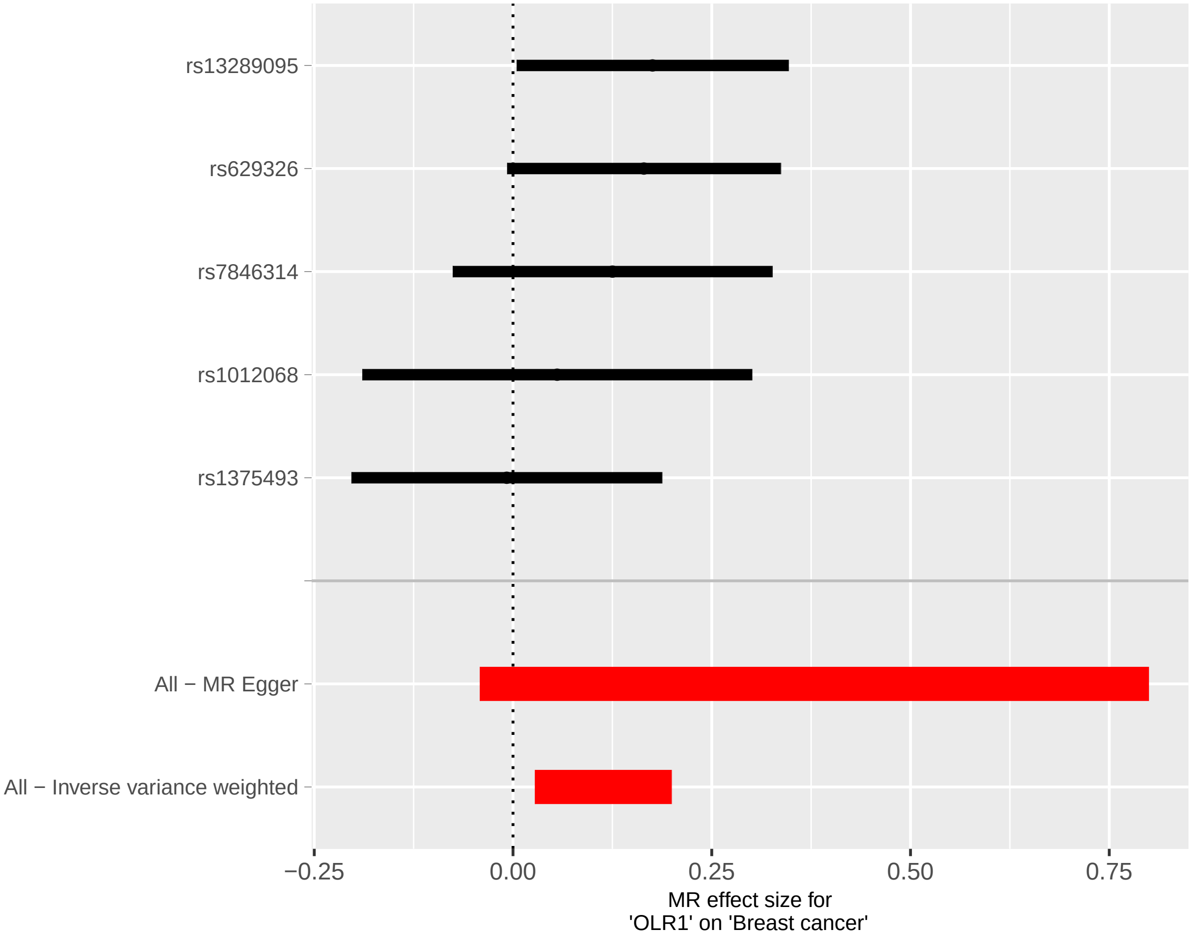
rs13289095
rs629326
rs7846314
rs1012068
rs1375493
All − MR Egger
All − Inverse variance weighted
−0.25
0.00
0.25
0.50
0.75
MR effect size for
'OLR1' on 'Breast cancer'

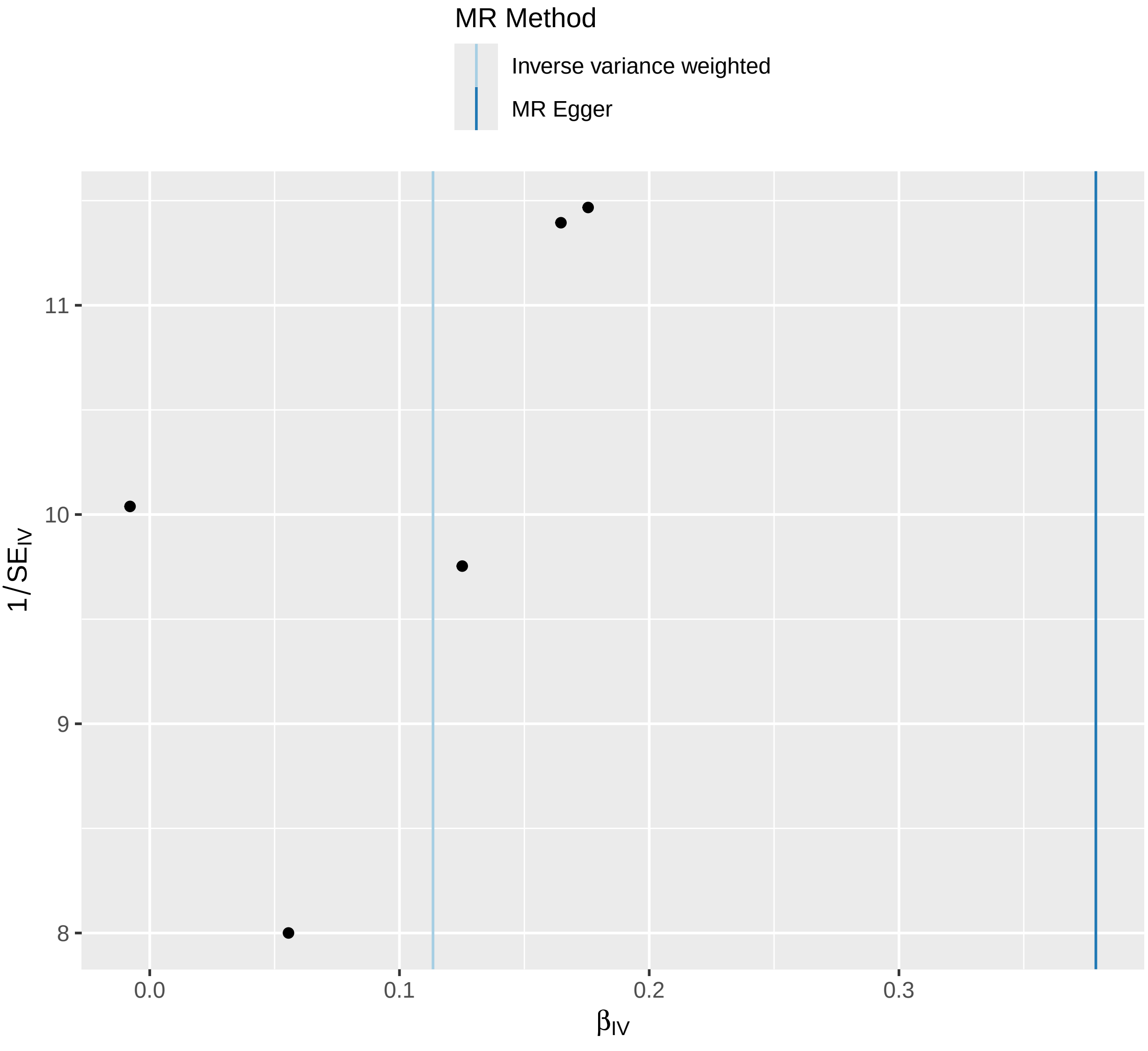
MR Method
Inverse variance weighted
MR Egger
11
10
9
8
1/SE_IV
0.0
0.1
0.2
0.3
β_IV

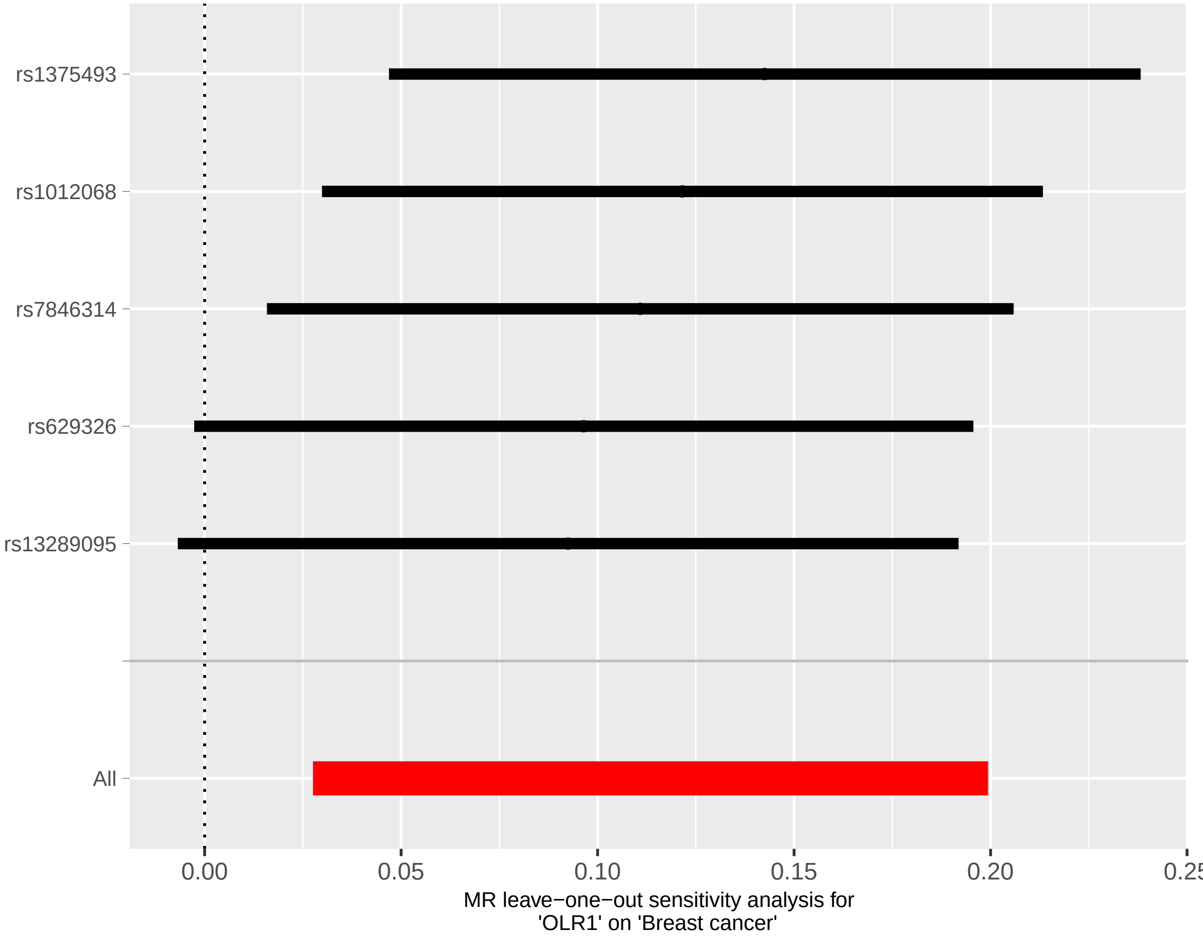
rs1375493
rs1012068
rs7846314
rs629326
rs13289095
All
0.00
0.05
0.10
0.15
0.20
0.25
MR leave−one−out sensitivity analysis for
'OLR1' on 'Breast cancer'

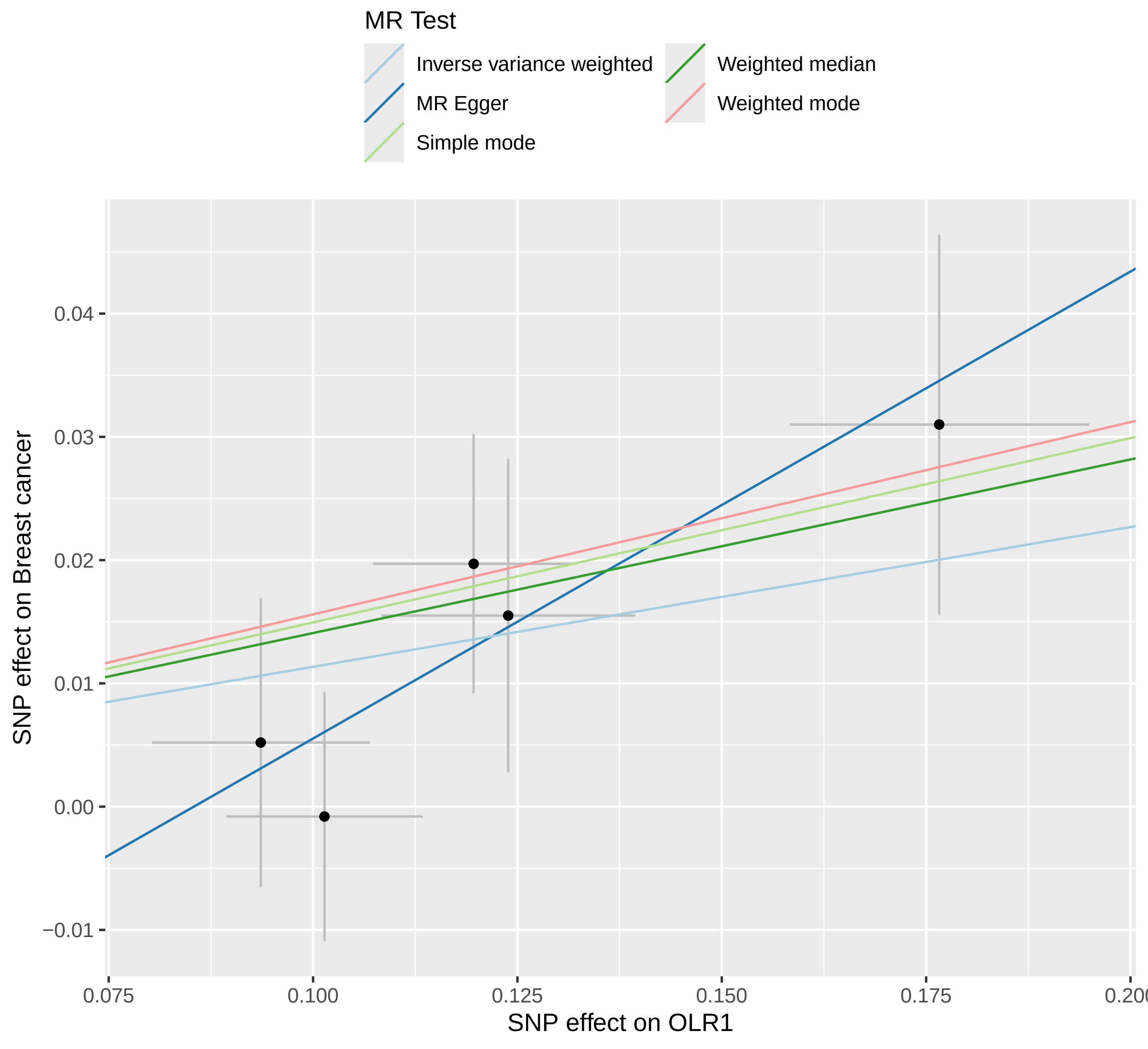
MR Test
Inverse variance weighted
MR Egger
Simple mode
Weighted median
Weighted mode
SNP effect on Breast cancer
0.04
0.03
0.02
0.01
0.00
−0.01
0.075
0.100
0.125
0.150
0.175
0.200
SNP effect on OLR1

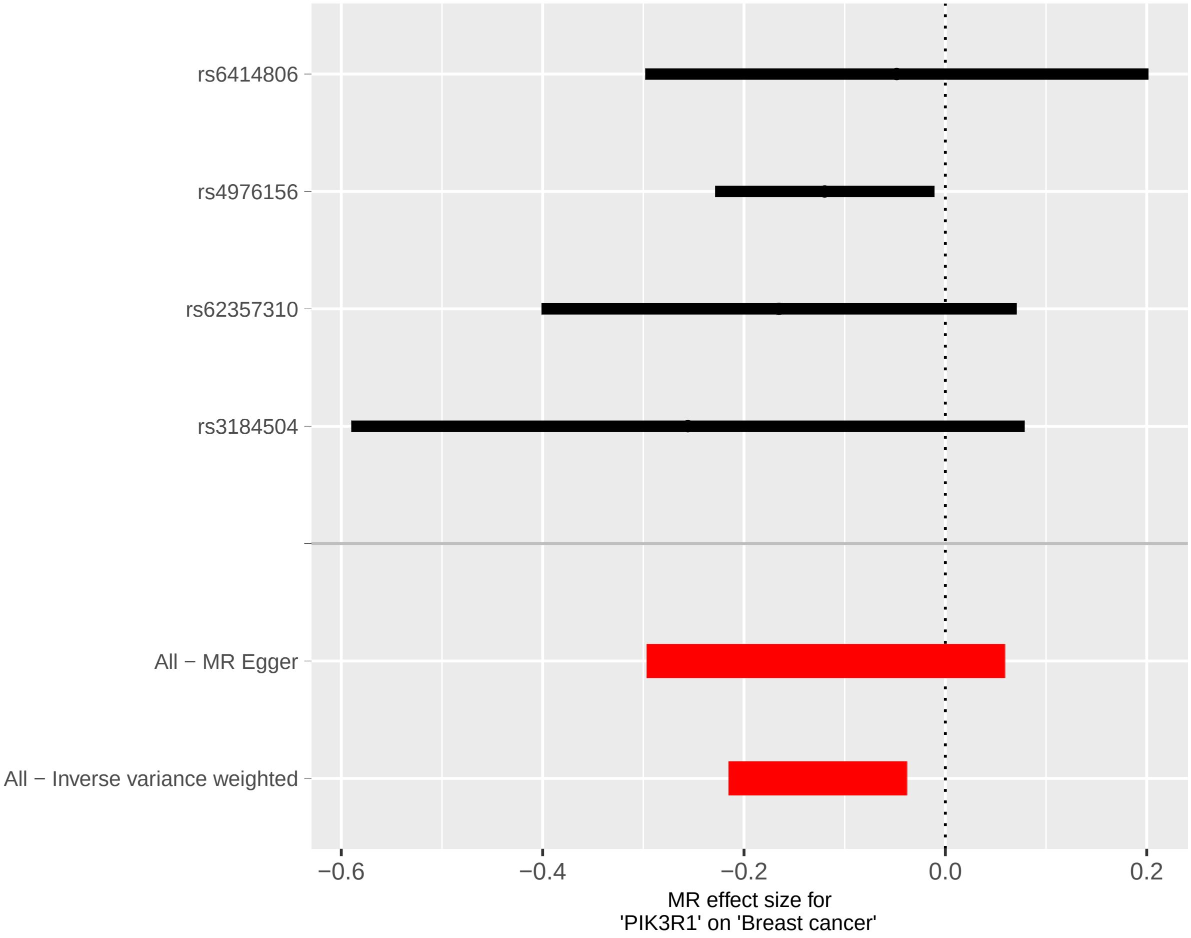
rs6414806
rs4976156
rs62357310
rs3184504
All − MR Egger
All − Inverse variance weighted
−0.6
−0.4
−0.2
0.0
0.2
MR effect size for
'PIK3R1' on 'Breast cancer'

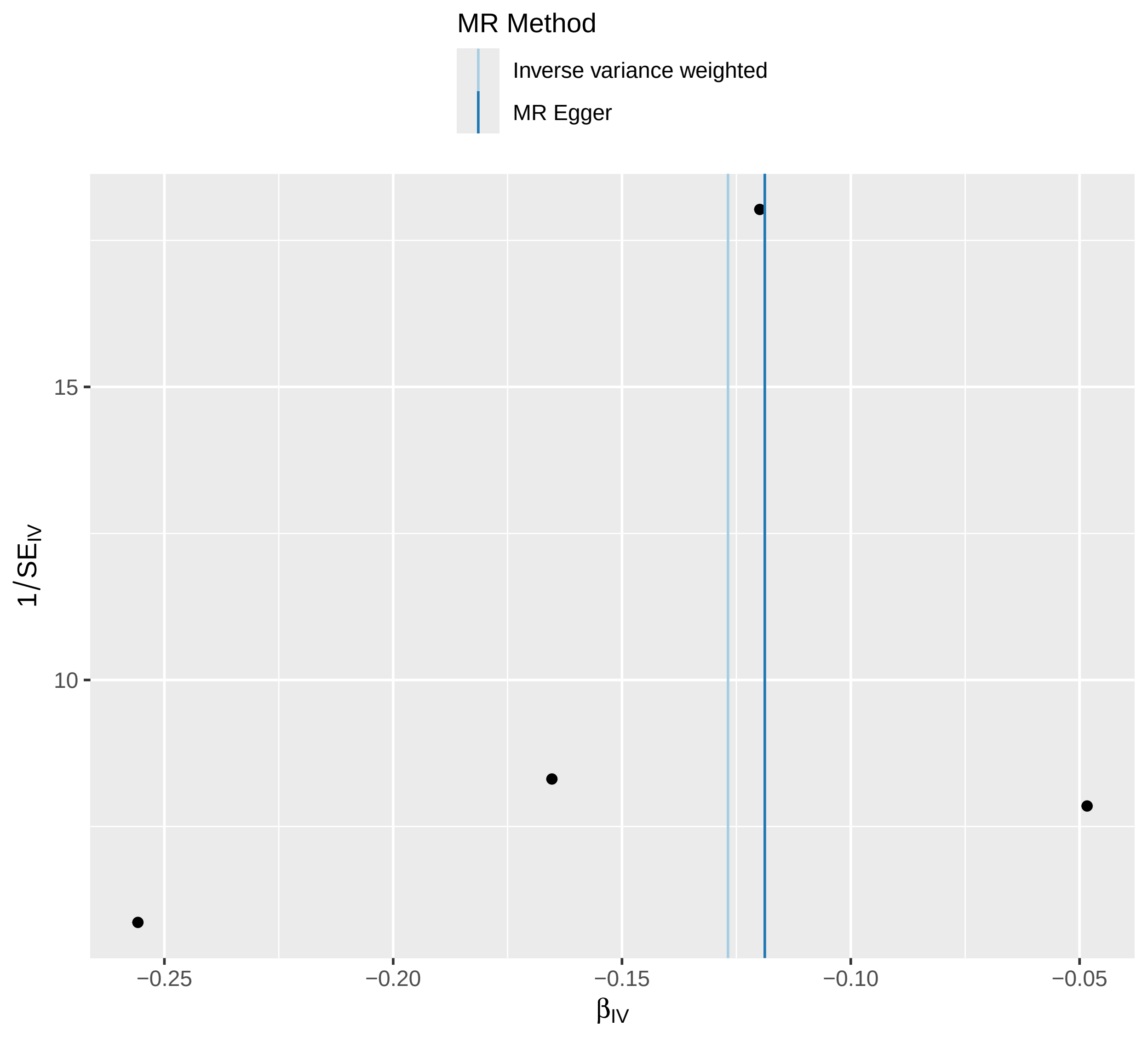

MR Method
Inverse variance weighted
MR Egger
15
10
1/SE$_{IV}$
−0.25
−0.20
−0.15
−0.10
−0.05
β$_{IV}$

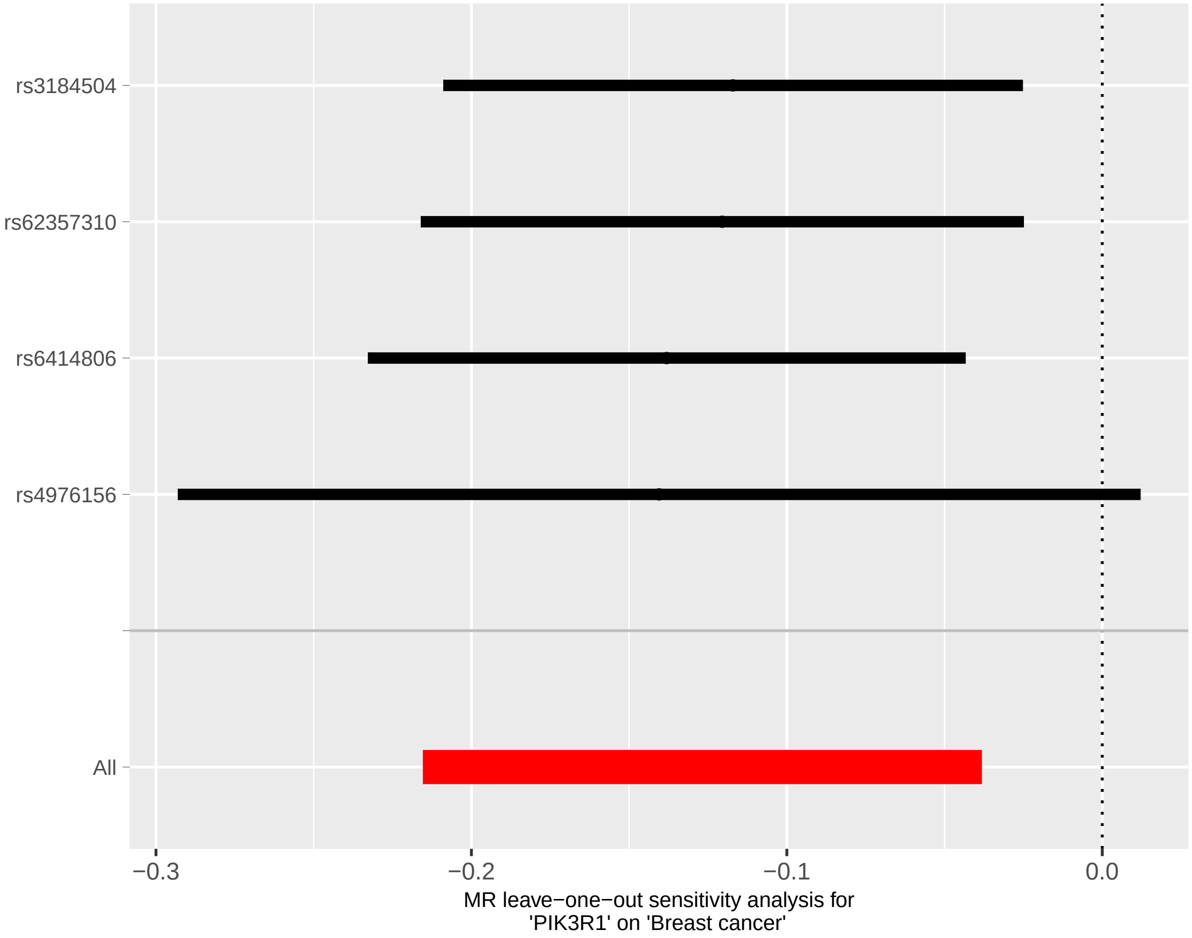
rs3184504
rs62357310
rs6414806
rs4976156
All
−0.3
−0.2
−0.1
0.0
MR leave−one−out sensitivity analysis for
'PIK3R1' on 'Breast cancer'

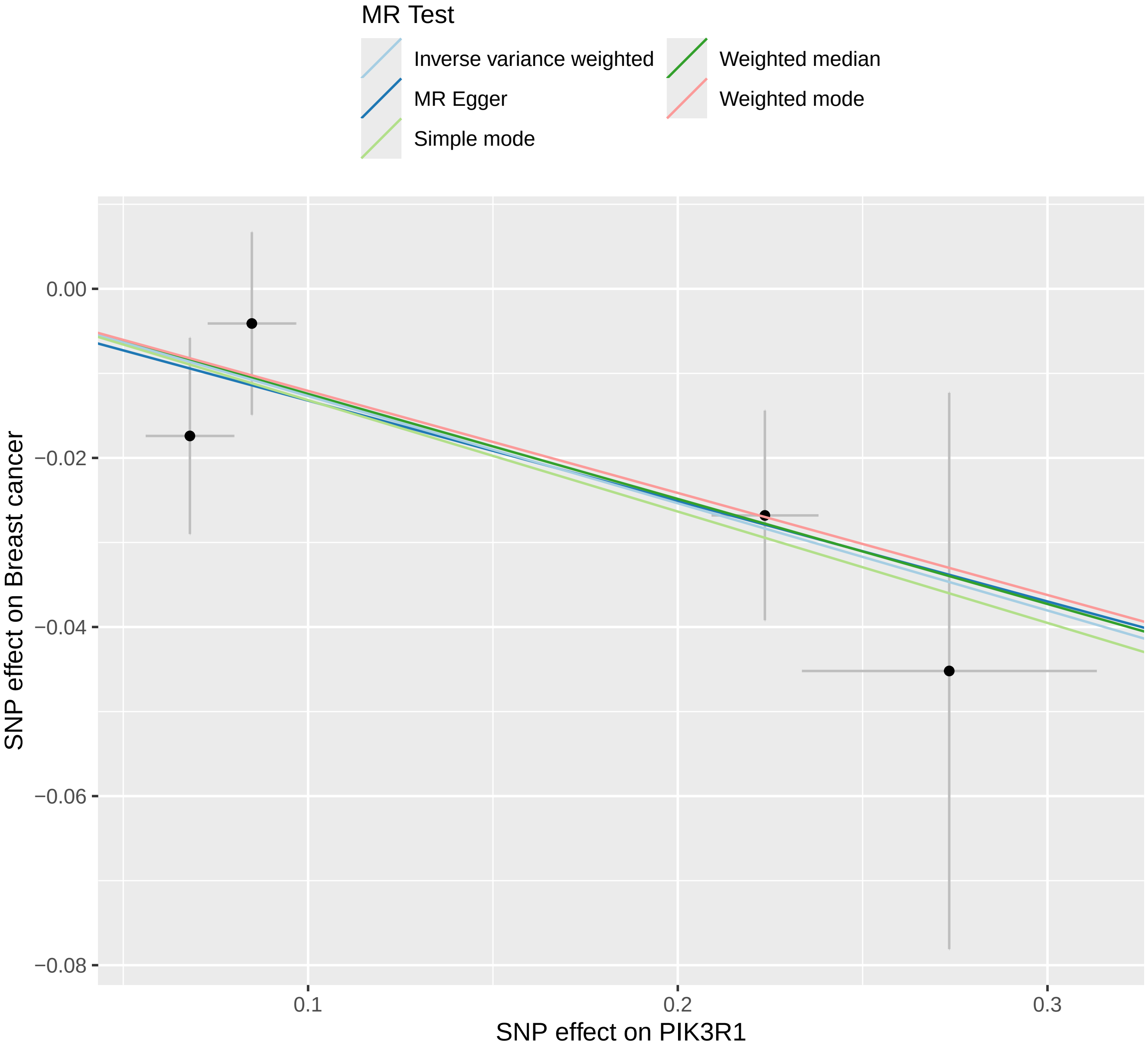
MR Test
Inverse variance weighted
MR Egger
Simple mode
Weighted median
Weighted mode
0.00
−0.02
−0.04
−0.06
−0.08
0.1
0.2
0.3
SNP effect on Breast cancer
SNP effect on PIK3R1

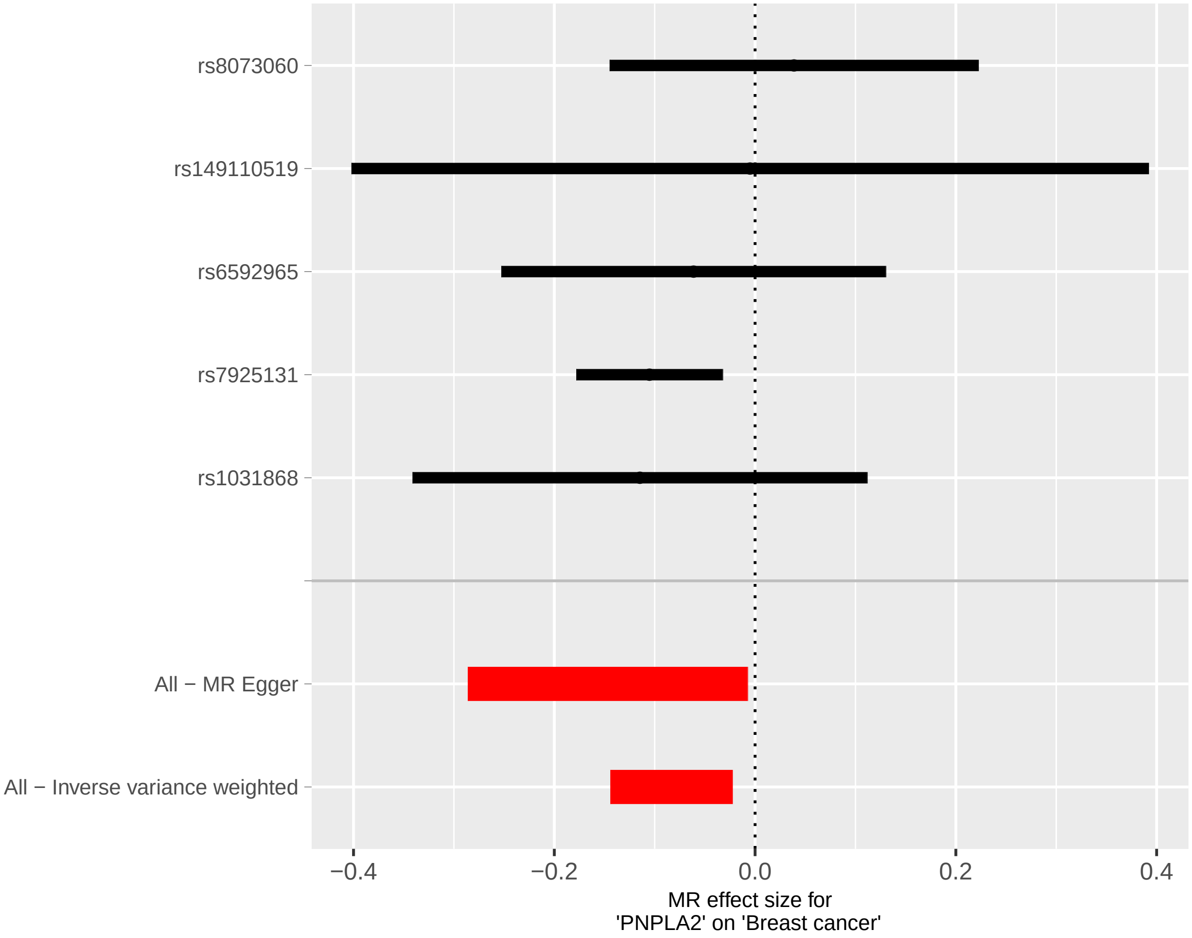

rs8073060
rs149110519
rs6592965
rs7925131
rs1031868
All − MR Egger
All − Inverse variance weighted
−0.4
−0.2
0.0
0.2
0.4
MR effect size for
'PNPLA2' on 'Breast cancer'

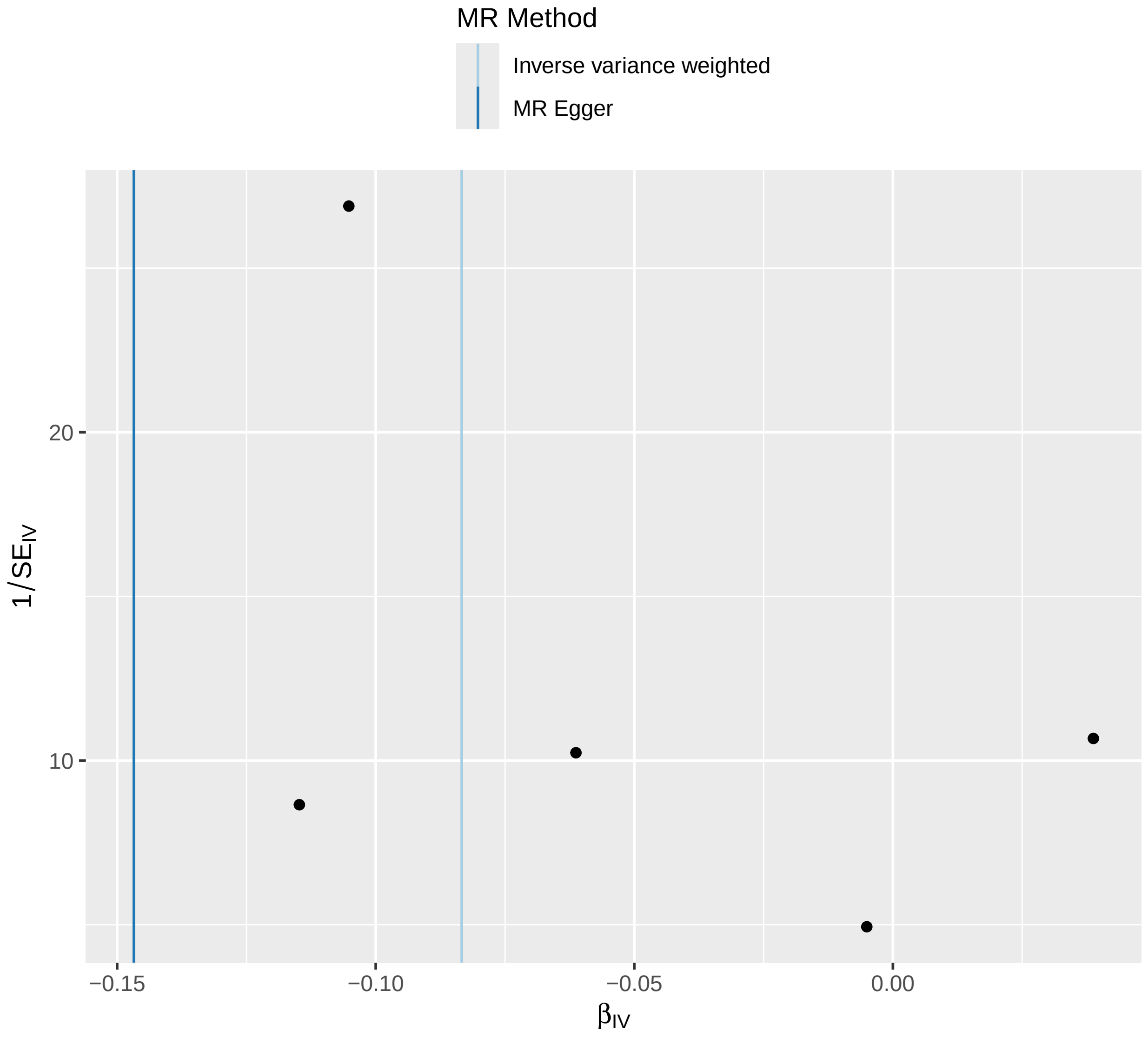
MR Method
Inverse variance weighted
MR Egger
20
10
1/SE$_{IV}$
−0.15
−0.10
−0.05
0.00
β$_{IV}$

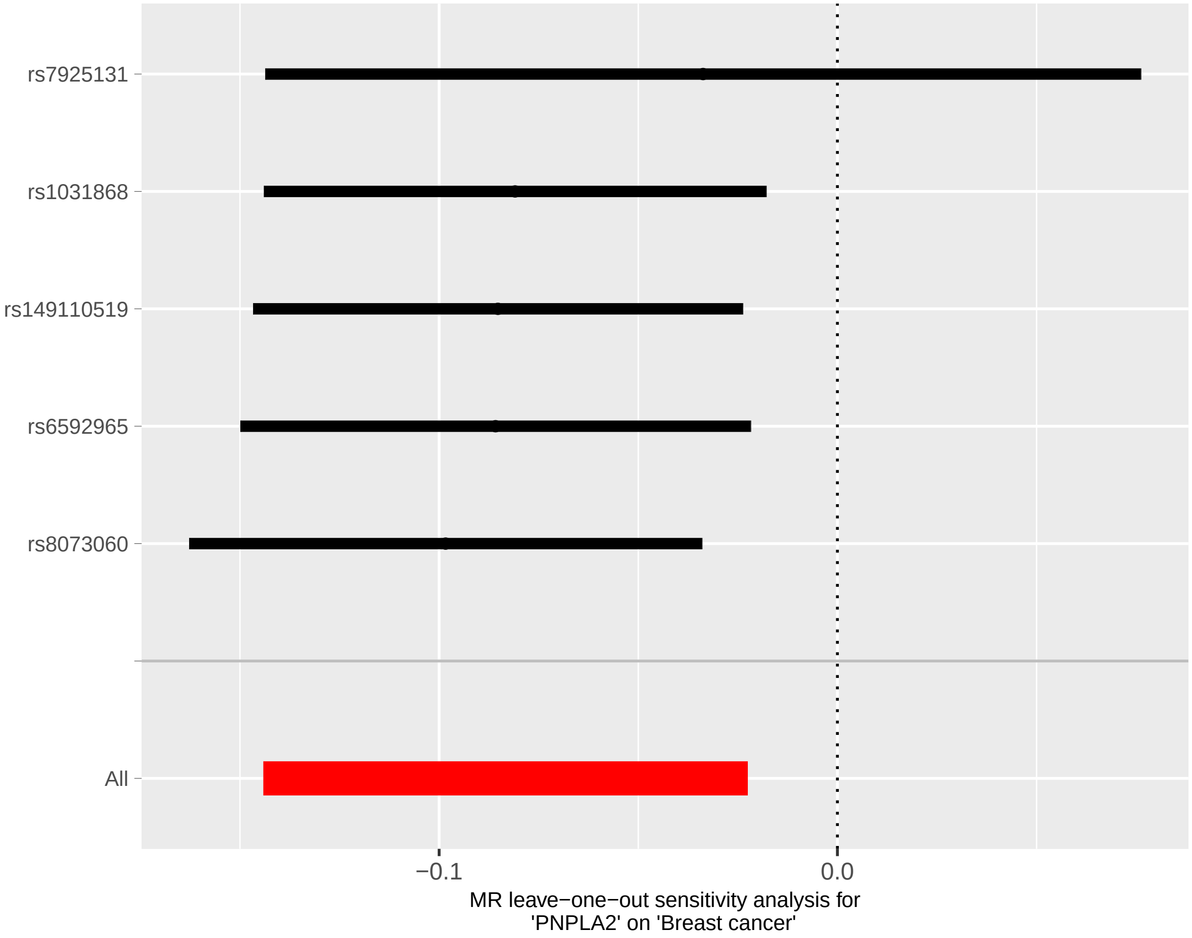

rs7925131
rs1031868
rs149110519
rs6592965
rs8073060
All
−0.1
0.0
MR leave−one−out sensitivity analysis for
'PNPLA2' on 'Breast cancer'

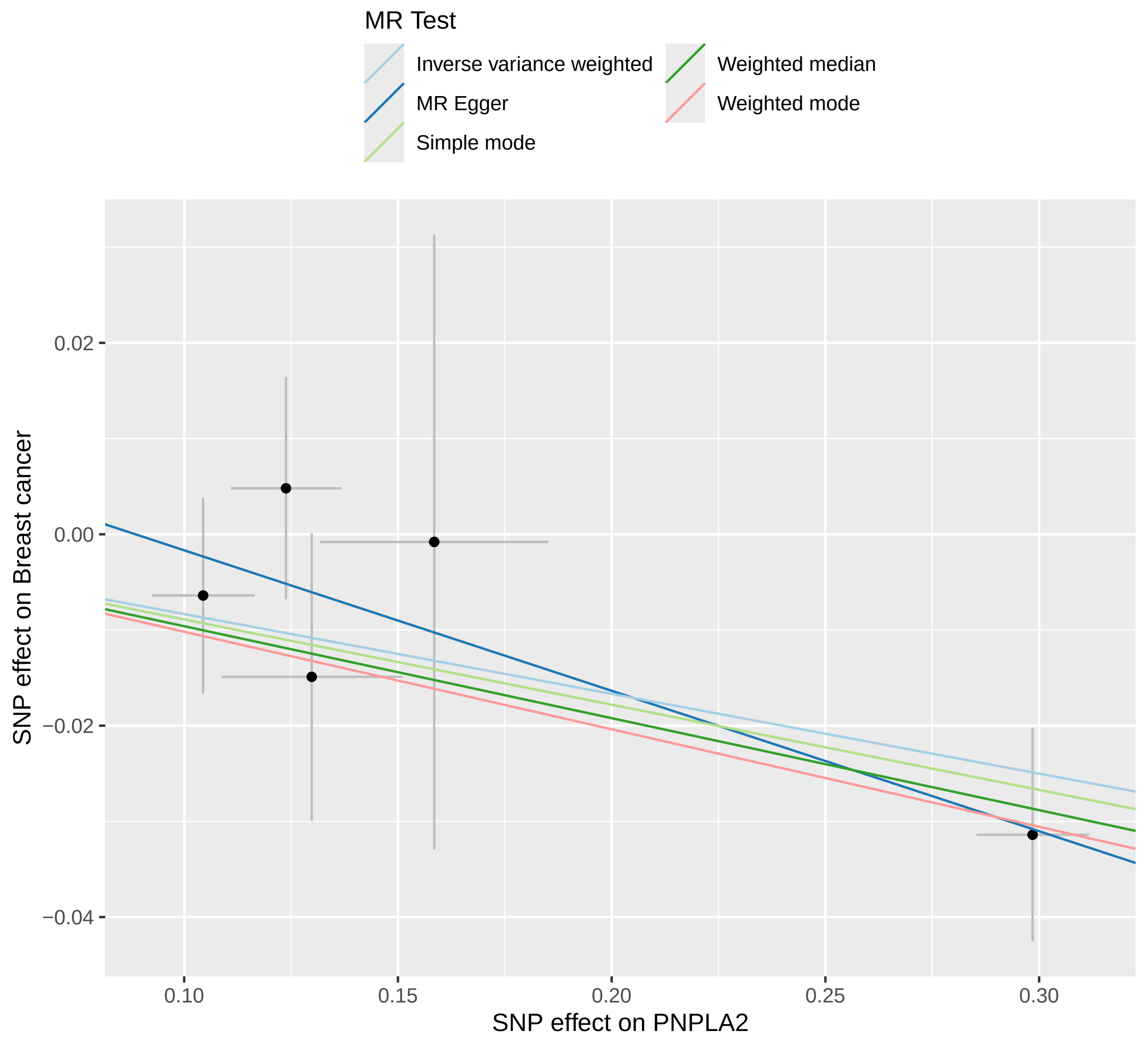
MR Test
Inverse variance weighted
MR Egger
Simple mode
Weighted median
Weighted mode
0.02
0.00
−0.02
−0.04
SNP effect on Breast cancer
0.10
0.15
0.20
0.25
0.30
SNP effect on PNPLA2

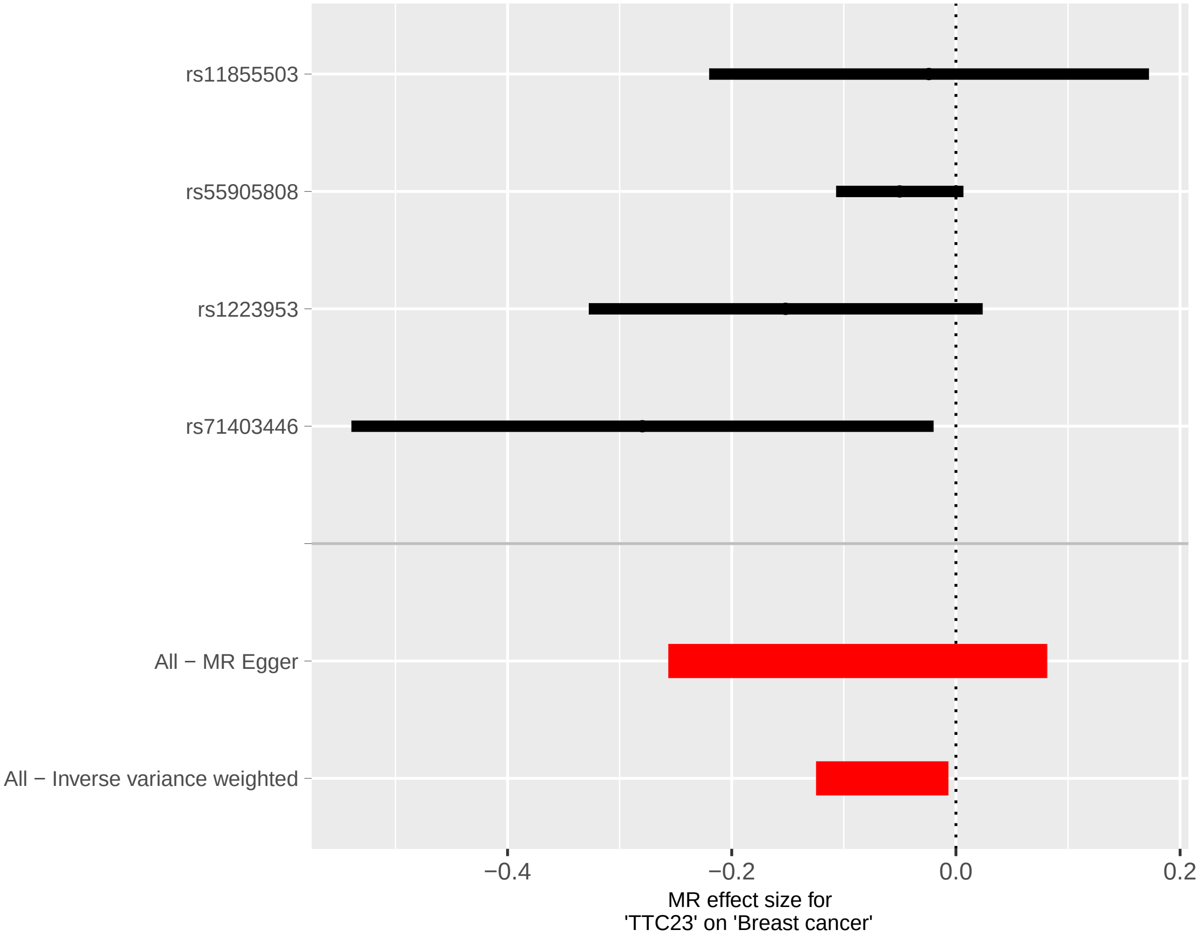
rs11855503
rs55905808
rs1223953
rs71403446
All − MR Egger
All − Inverse variance weighted
−0.4
−0.2
0.0
0.2
MR effect size for
'TTC23' on 'Breast cancer'

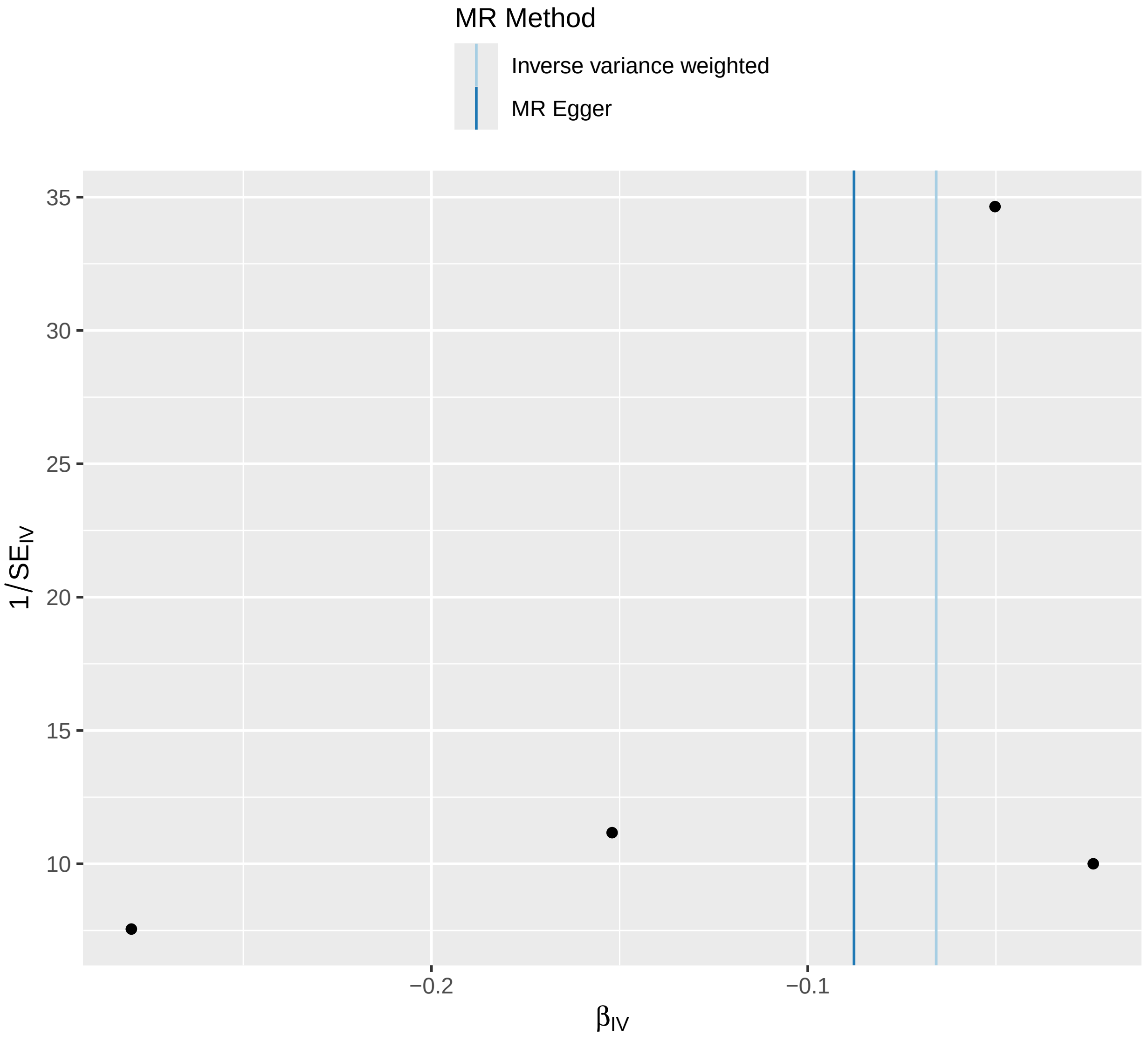
MR Method
Inverse variance weighted
MR Egger
35
30
25
20
15
10
1/SE$_{IV}$
−0.2
−0.1
β$_{IV}$

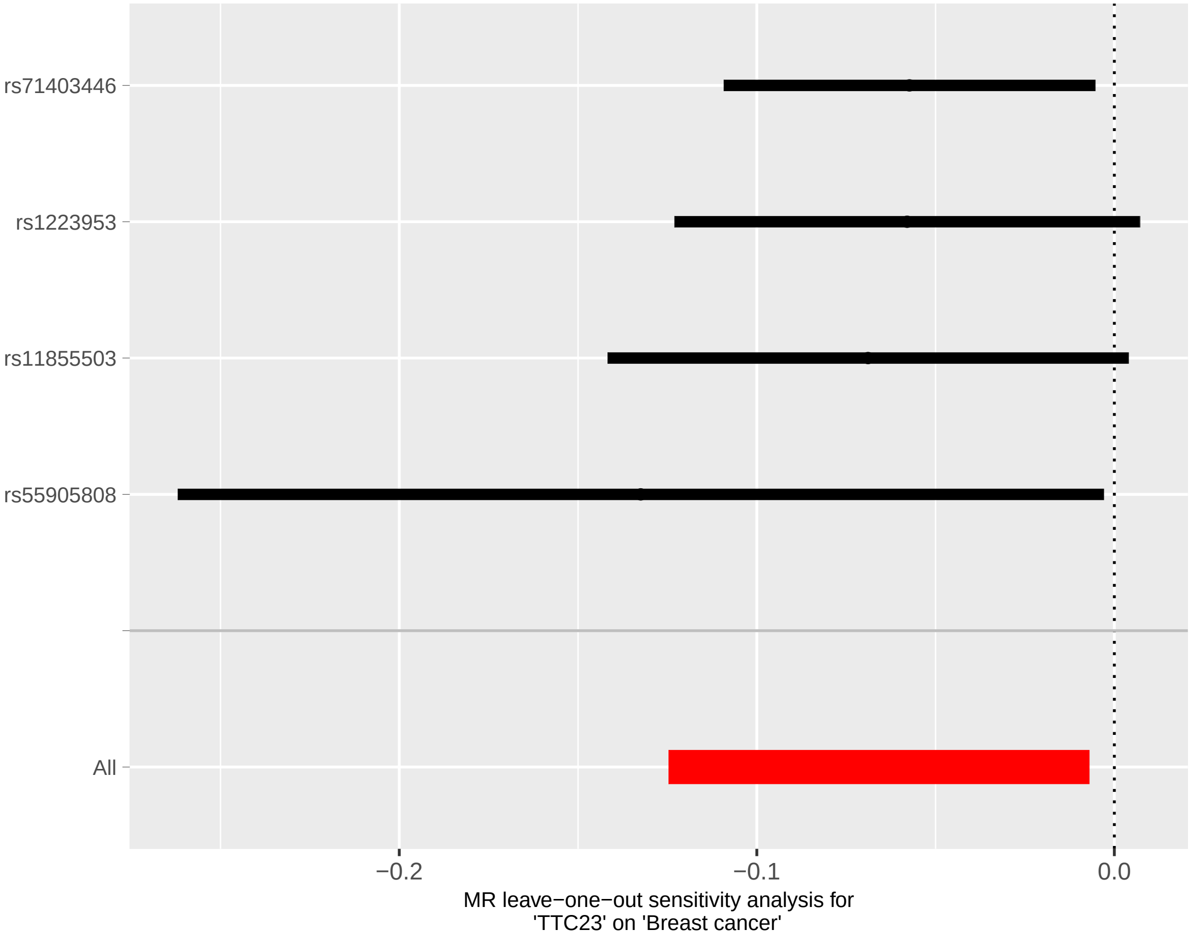

rs71403446
rs1223953
rs11855503
rs55905808
All
−0.2
−0.1
0.0
MR leave−one−out sensitivity analysis for
'TTC23' on 'Breast cancer'

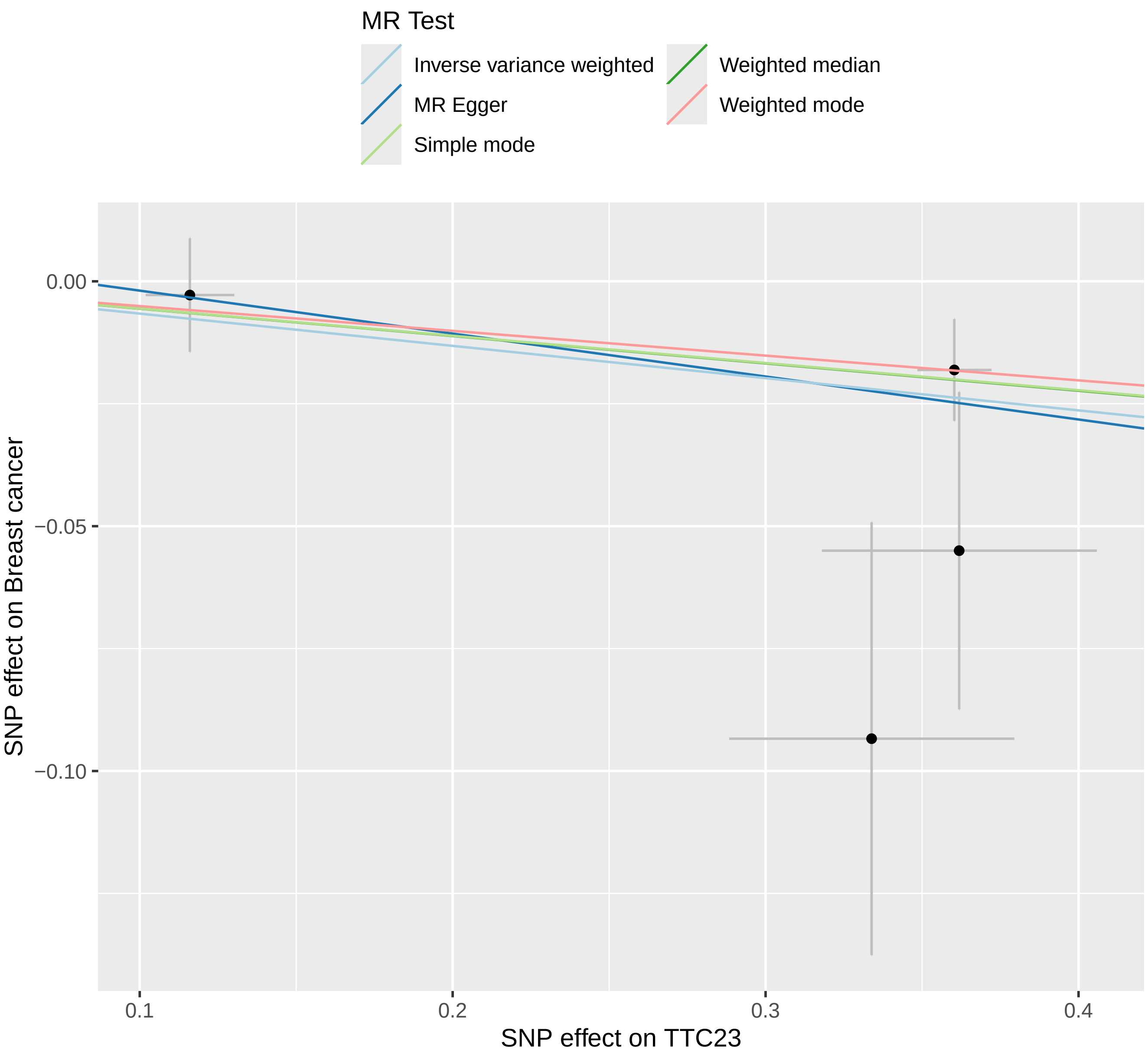
MR Test
Inverse variance weighted
MR Egger
Simple mode
Weighted median
Weighted mode
0.00
−0.05
−0.10
SNP effect on Breast cancer
0.1
0.2
0.3
0.4
SNP effect on TTC23
