# Supplementary material for: Exploring Novel Therapeutic Targets in Breast Cancer via Comprehensive Omics Profiling and Experimental Verification
Source: Biology (Basel). 2025 Apr 11;14(4):405. doi: 10.3390/biology14040405 (PMC12025194; doi:10.3390/biology14040405)
Supplement: Supplementary file 1 [file biology-14-00405-s001.zip › Supplementary Figure S2.pdf]

Exploring Novel Therapeutic Targets in Breast Cancer via Comprehensive Omics Profiling and Experimental Verification

Supplementary Figure 2 Supplementary part of drug sensitivity analysis of ATOH8 and DNASE2 genes

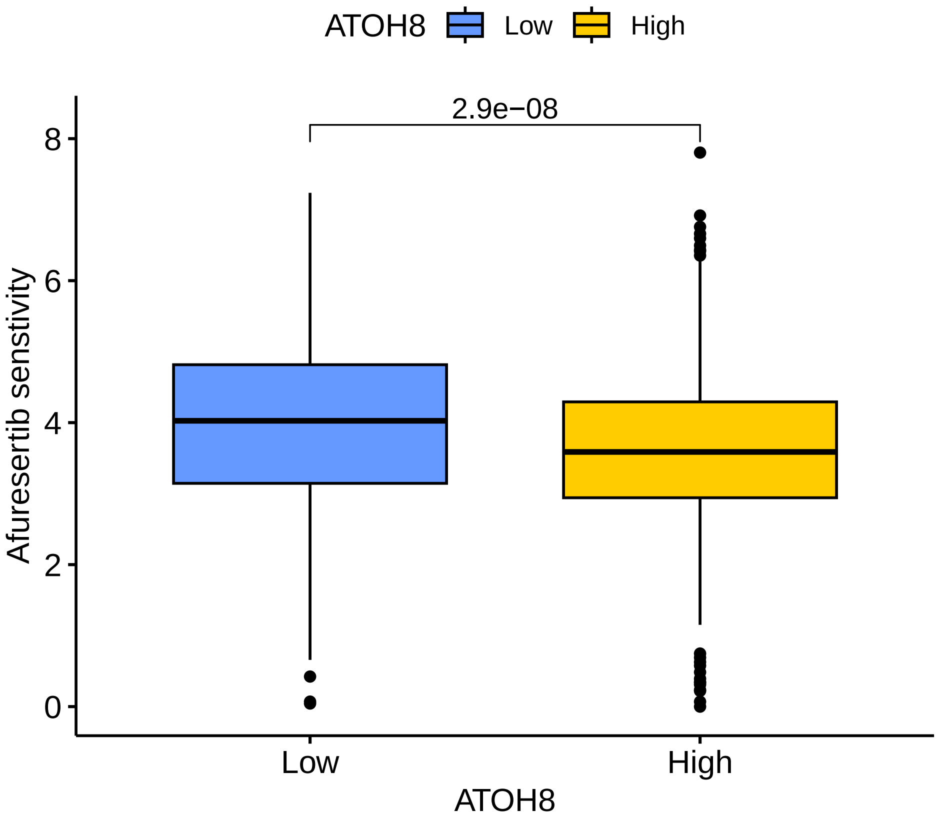
ATOH8
Low
High
2.9e−08
8
6
4
2
0
Afuresertib senstivity
Low
High
ATOH8

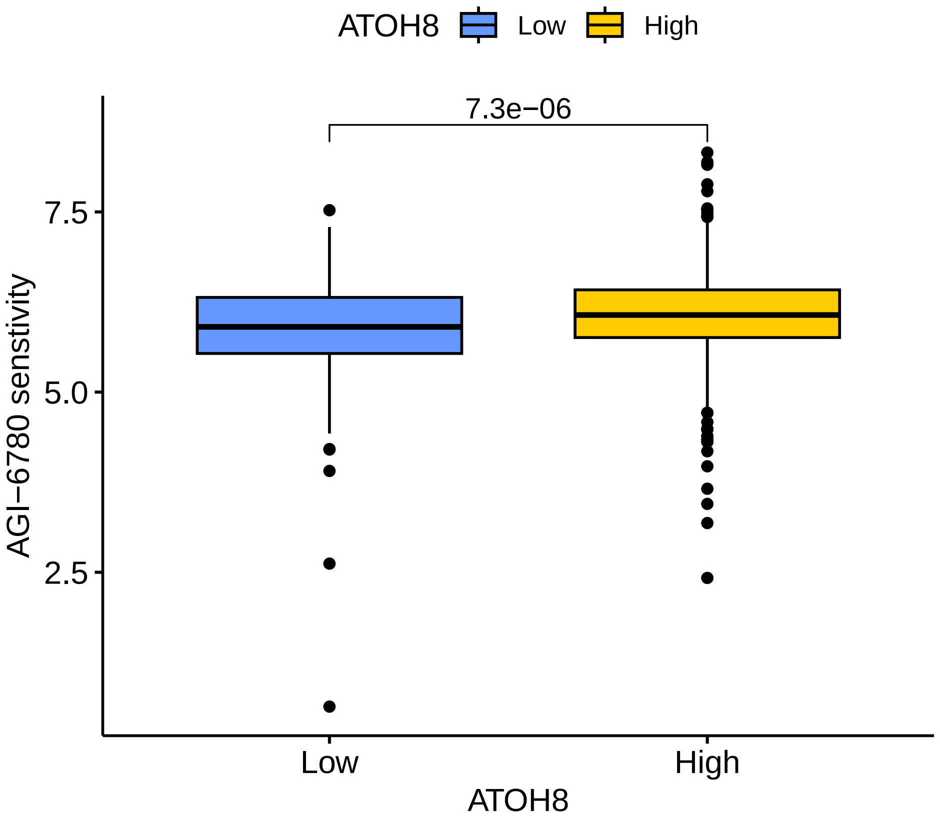

ATOH8
Low
High
7.3e−06
AGI−6780 senstivity
7.5
5.0
2.5
Low
High
ATOH8

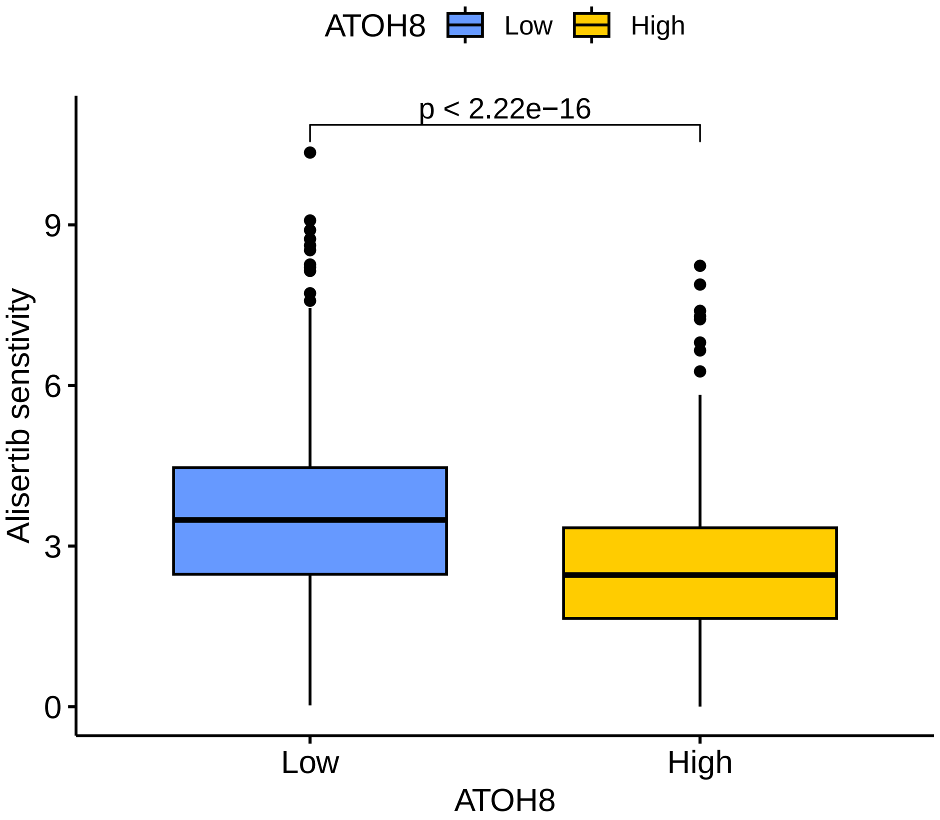
ATOH8
Low
High
p < 2.22e−16
Alisertib senstivity
9
6
3
0
Low
High
ATOH8

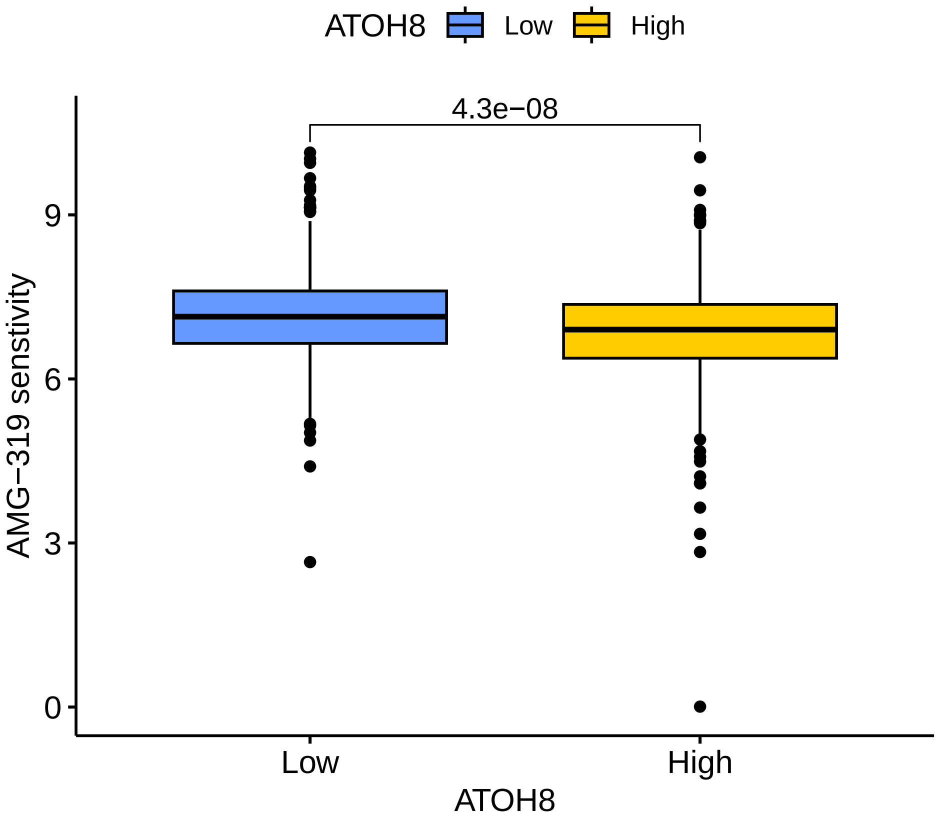

ATOH8
Low
High
4.3e−08
AMG−319 senstivity
9
6
3
0
Low
High
ATOH8

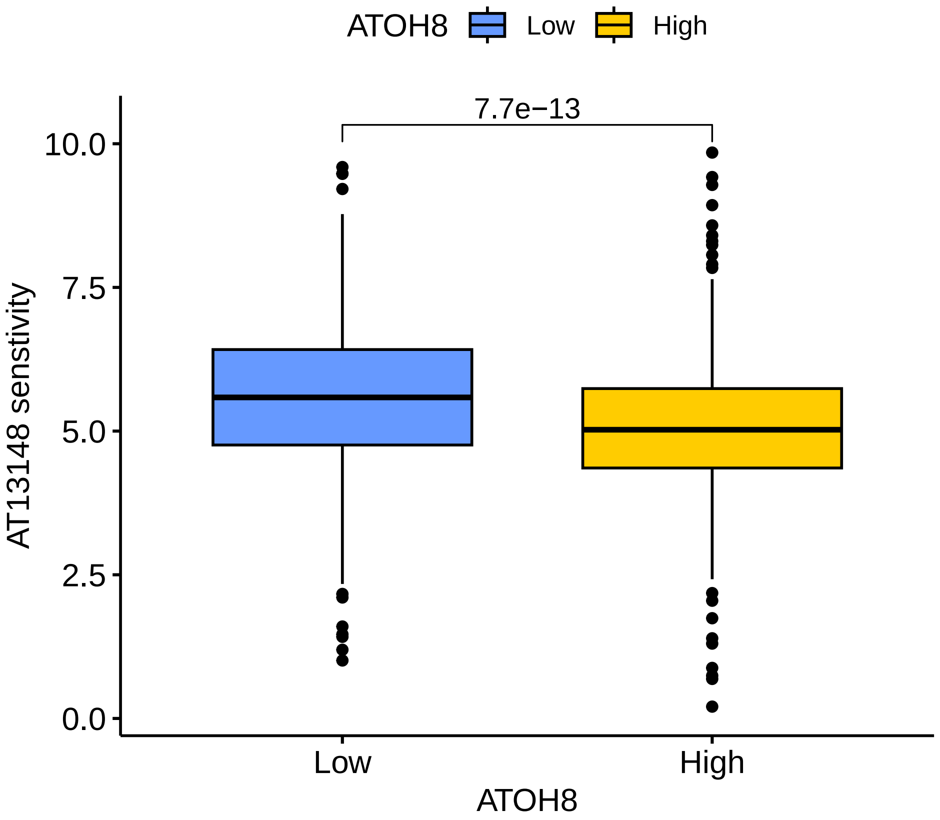
ATOH8
Low
High
7.7e−13
10.0
7.5
5.0
2.5
0.0
AT13148 senstivity
Low
High
ATOH8

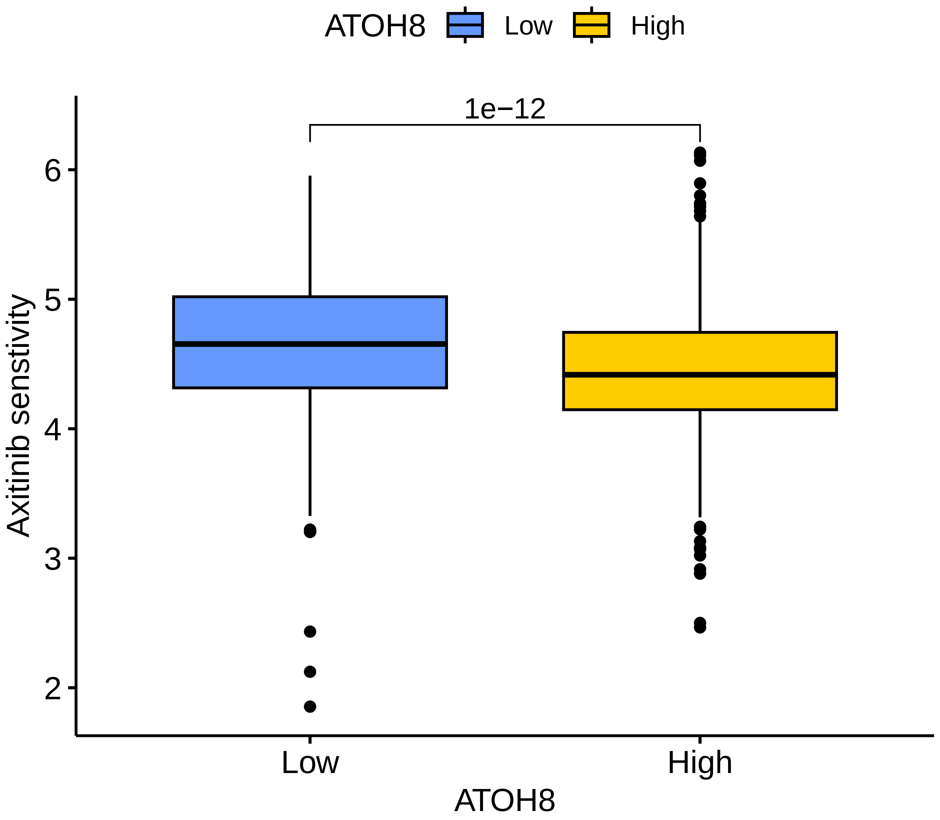
ATOH8
Low
High
1e−12
Axitinib senstivity
6
5
4
3
2
Low
High
ATOH8

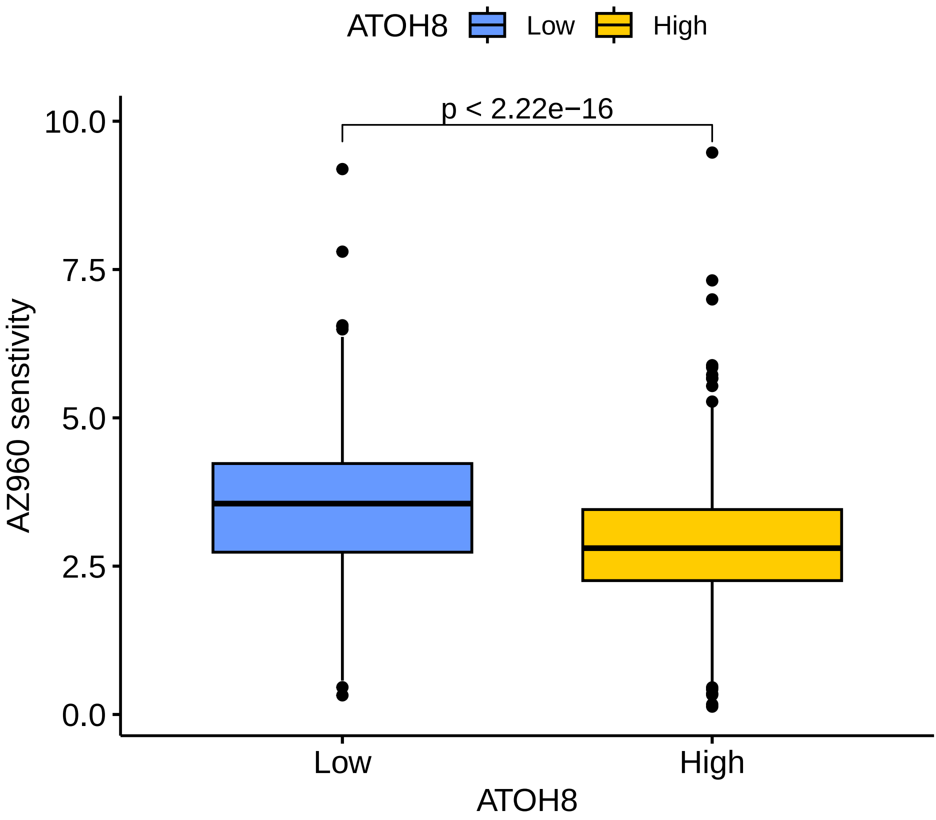
ATOH8
Low
High
p < 2.22e−16
10.0
7.5
5.0
2.5
0.0
AZ960 senstivity
Low
High
ATOH8

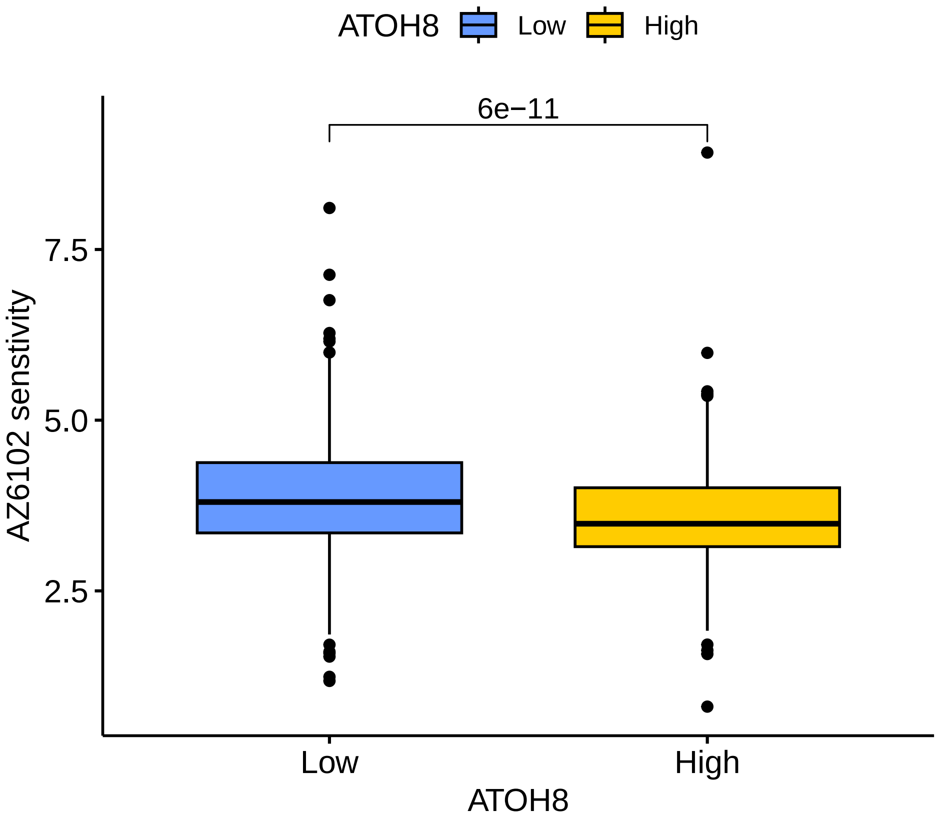

ATOH8
Low
High
6e−11
AZ6102 senstivity
7.5
5.0
2.5
Low
High
ATOH8

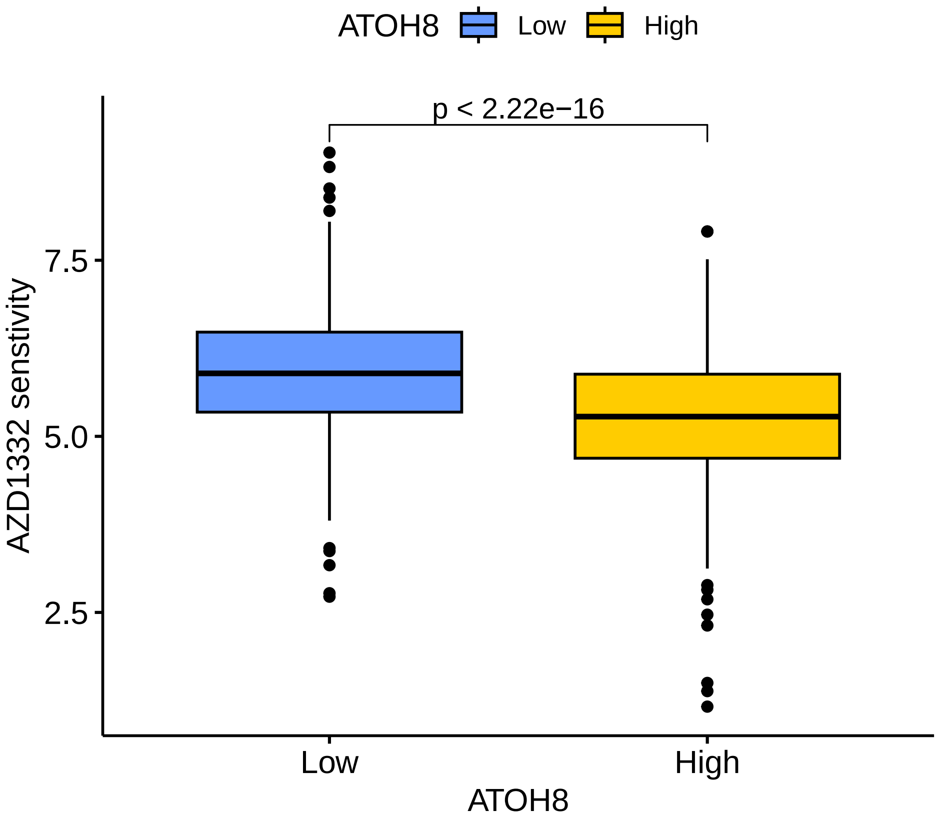
ATOH8
Low
High
p < 2.22e−16
7.5
5.0
2.5
AZD1332 senstivity
Low
High
ATOH8

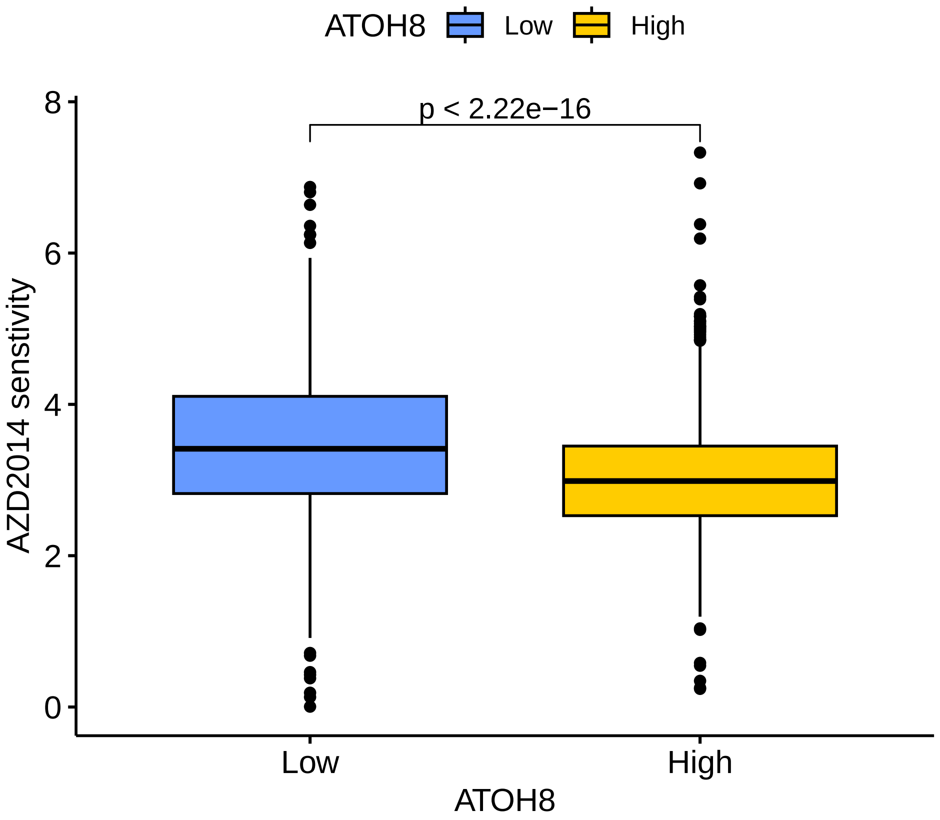
ATOH8
Low
High
p < 2.22e−16
AZD2014 senstivity
8
6
4
2
0
Low
High
ATOH8

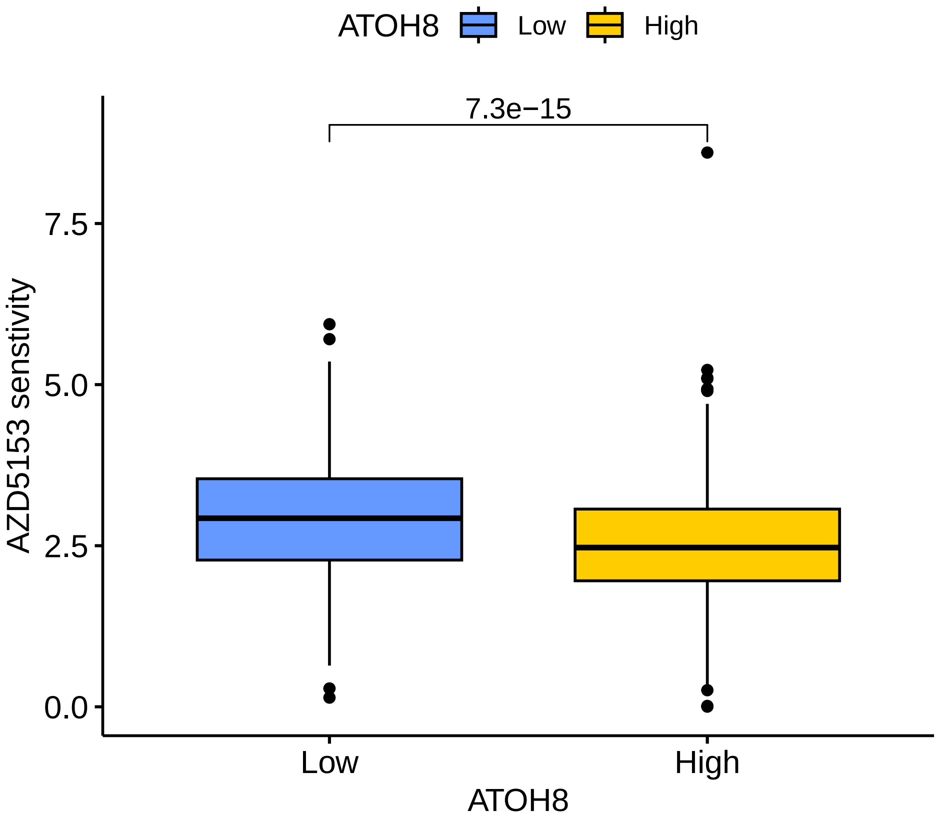
ATOH8
Low
High
7.3e−15
AZD5153 senstivity
7.5
5.0
2.5
0.0
Low
High
ATOH8

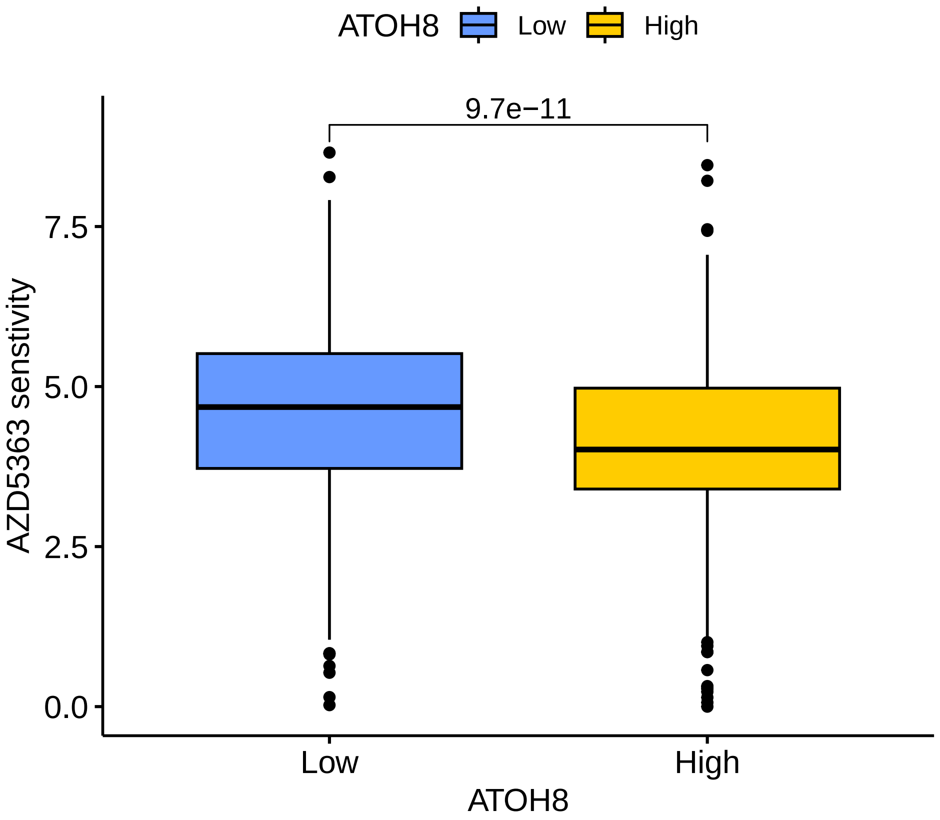

ATOH8
Low
High
9.7e−11
AZD5363 senstivity
7.5
5.0
2.5
0.0
Low
High
ATOH8

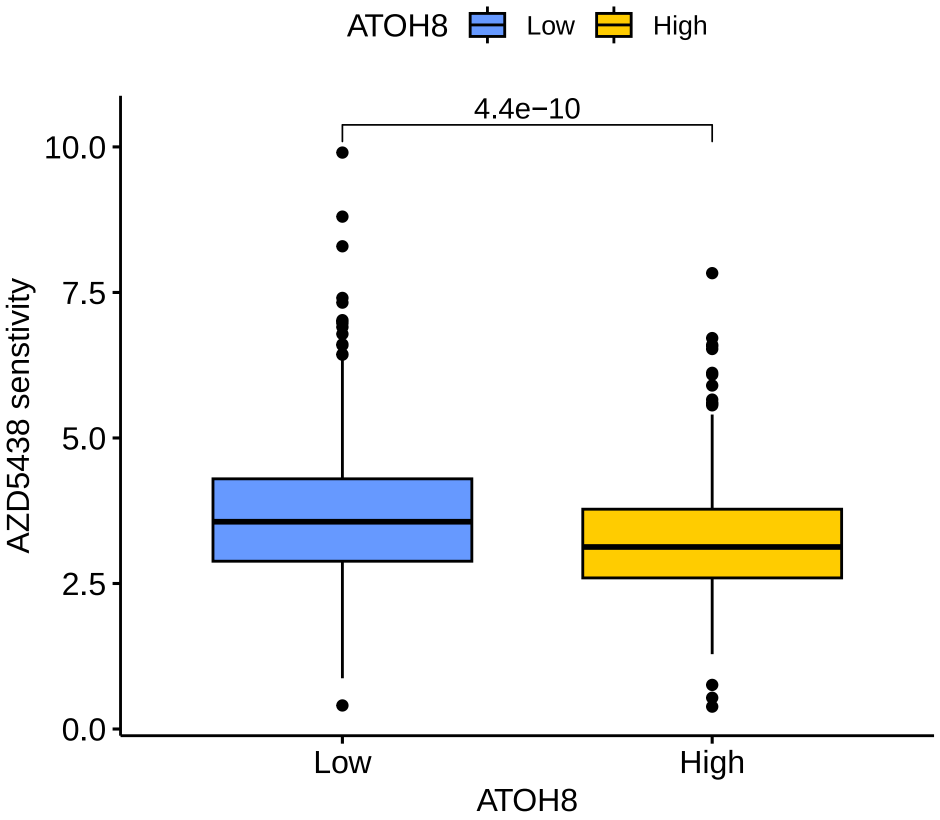
ATOH8
Low
High
4.4e−10
10.0
7.5
5.0
2.5
0.0
AZD5438 senstivity
Low
High
ATOH8

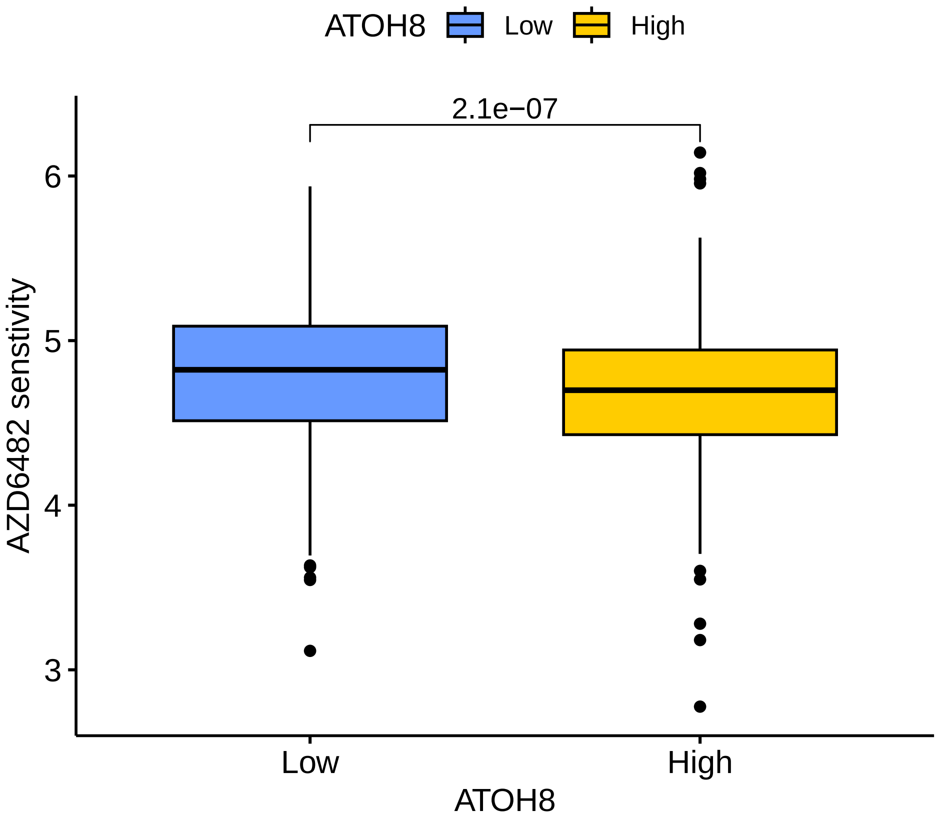

ATOH8
Low
High
2.1e−07
AZD6482 senstivity
6
5
4
3
Low
High
ATOH8

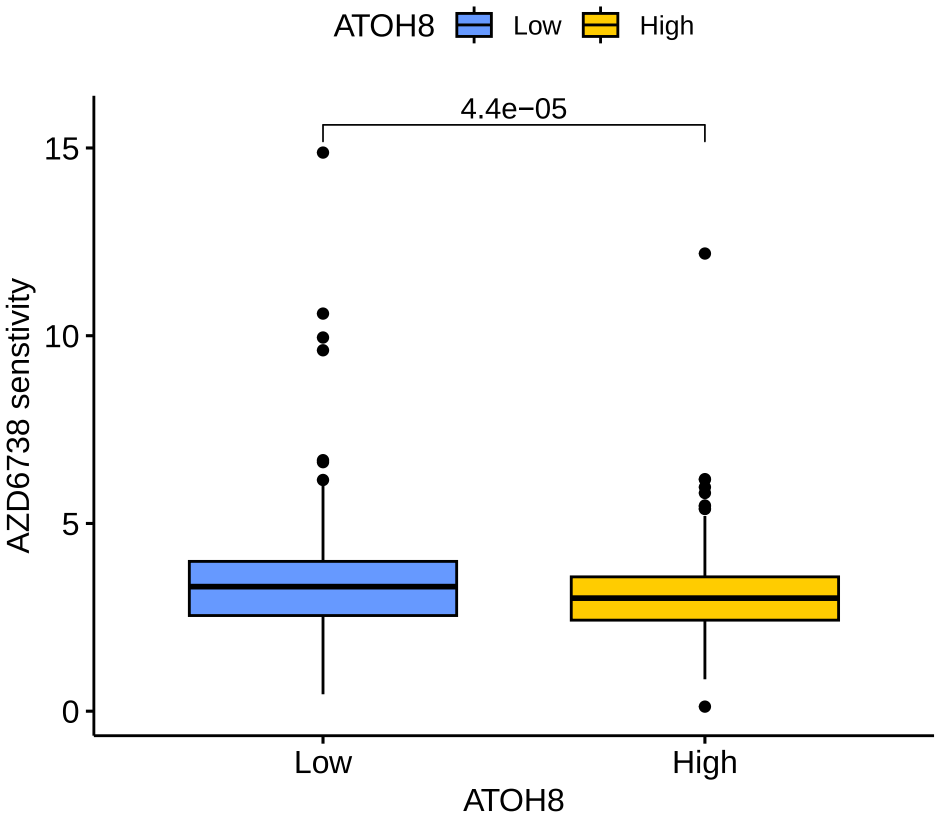
ATOH8
Low
High
4.4e−05
15
10
5
0
AZD6738 senstivity
Low
High
ATOH8

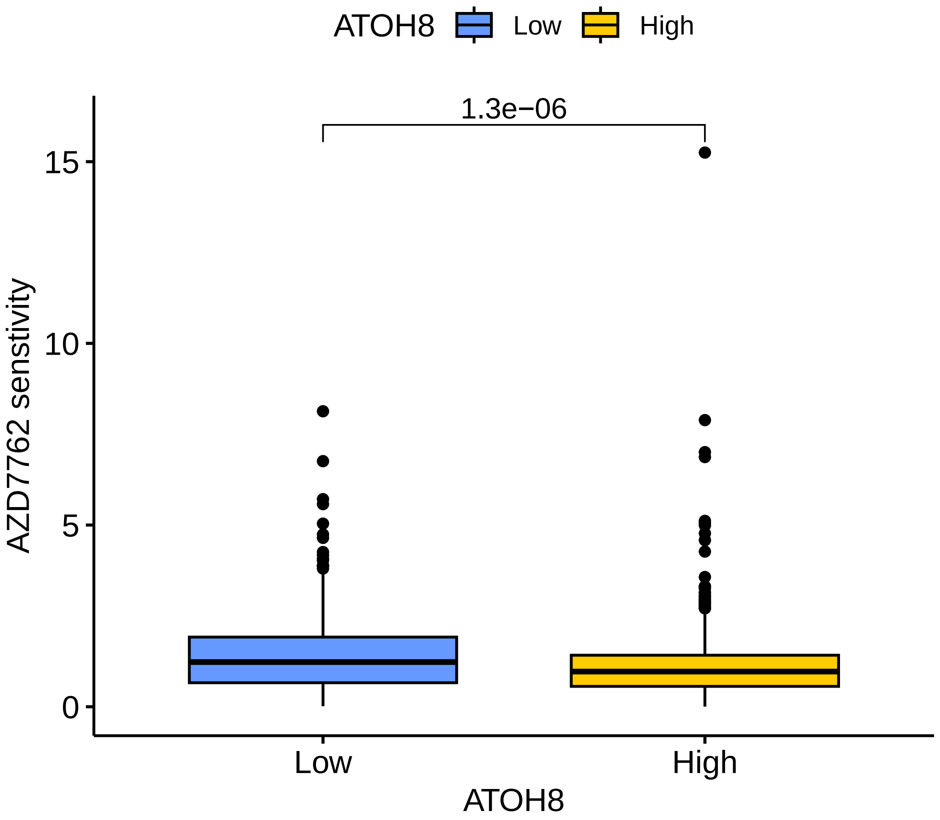

ATOH8
Low
High
1.3e−06
15
10
5
0
AZD7762 senstivity
Low
High
ATOH8

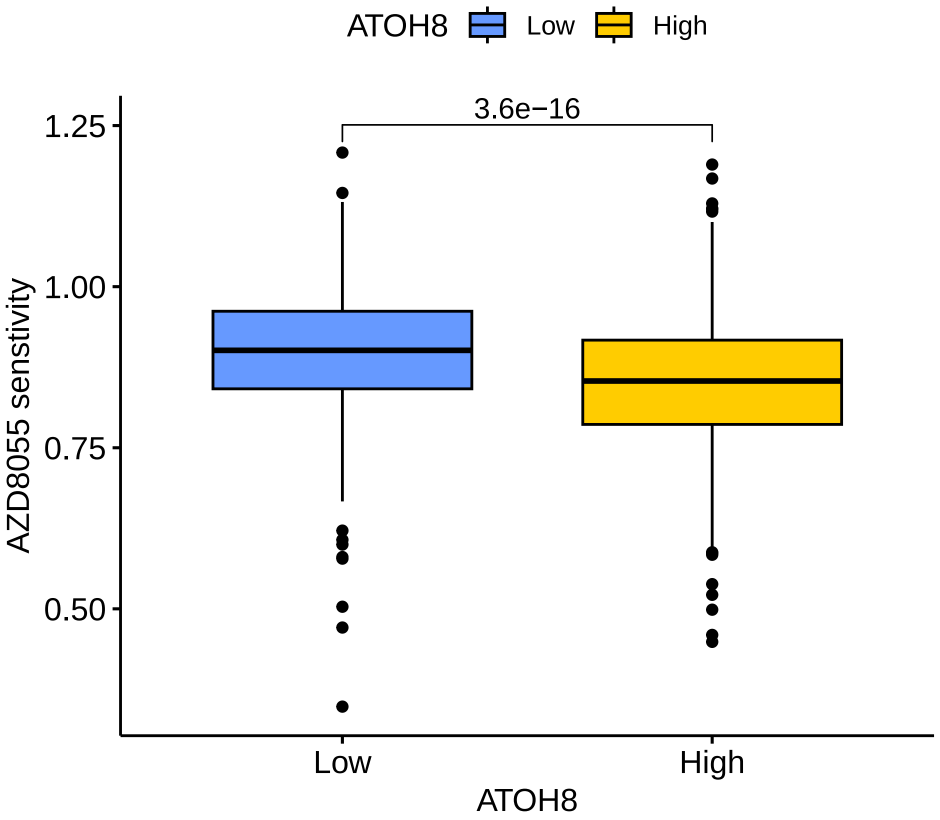

ATOH8
Low
High
3.6e−16
1.25
1.00
0.75
0.50
AZD8055 senstivity
Low
High
ATOH8

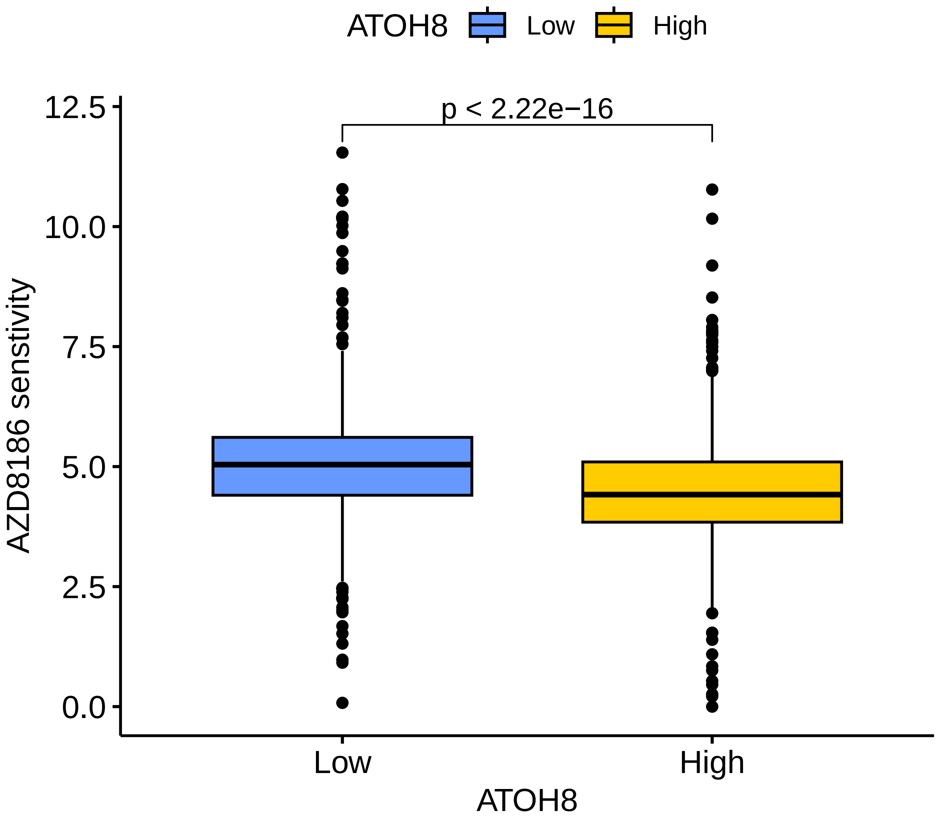
ATOH8
Low
High
p < 2.22e−16
12.5
10.0
7.5
5.0
2.5
0.0
AZD8186 senstivity
Low
High
ATOH8

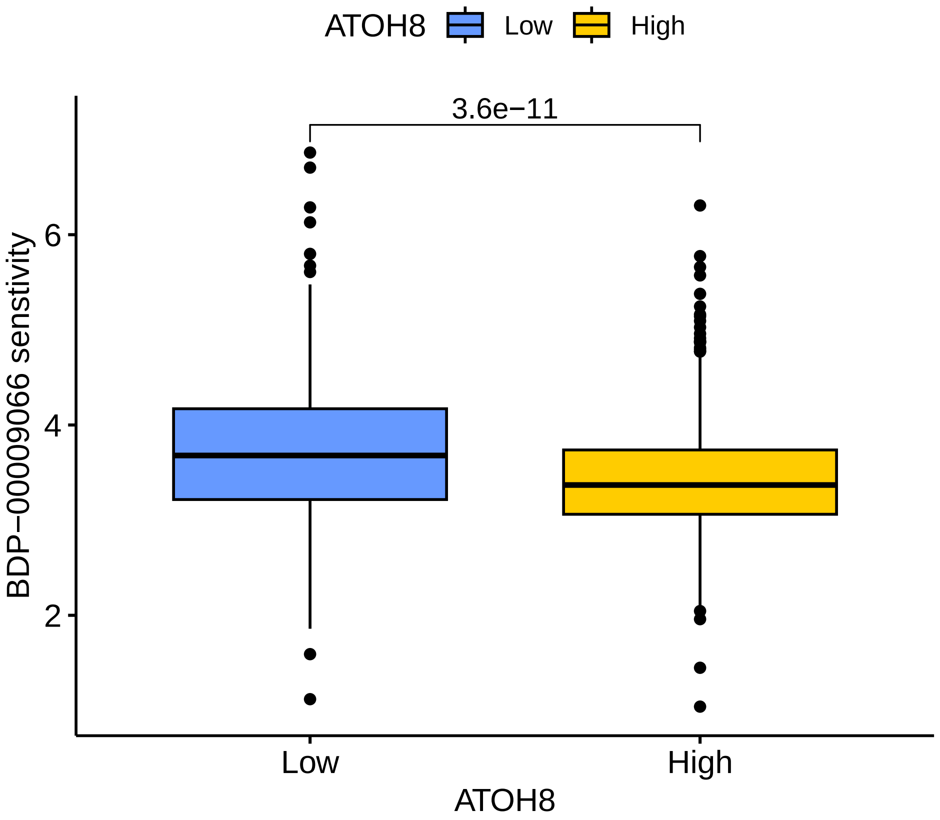

ATOH8
Low
High
3.6e−11
BDP−00009066 senstivity
6
4
2
Low
High
ATOH8

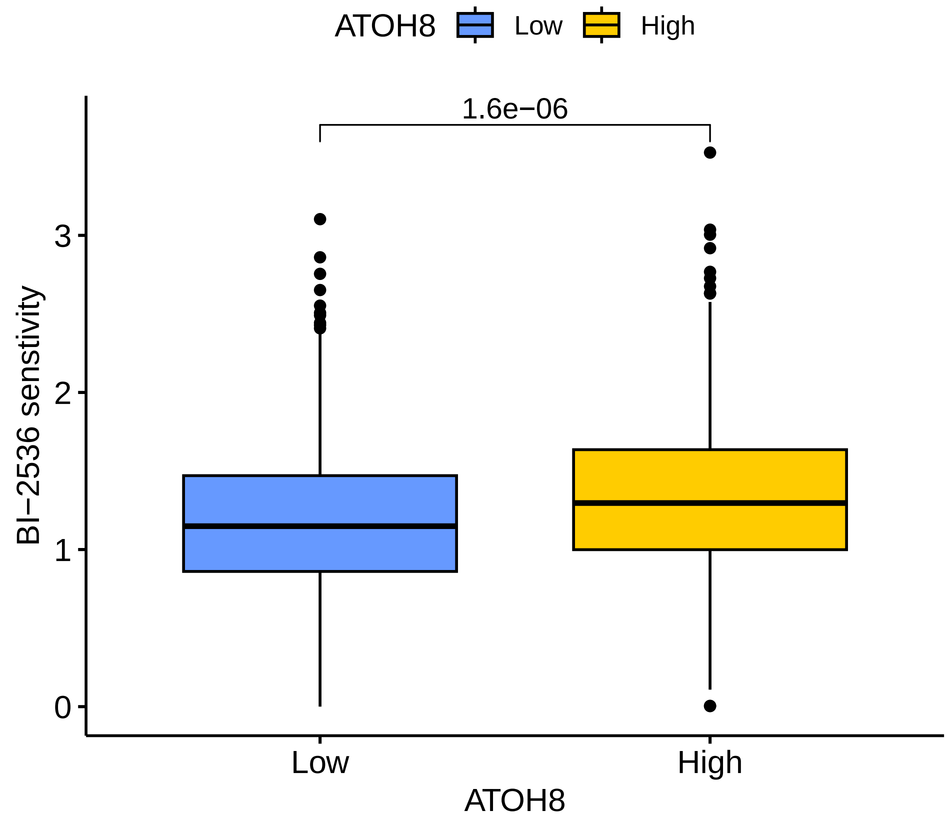

ATOH8
Low
High
1.6e−06
BI−2536 senstivity
0
1
2
3
Low
High
ATOH8

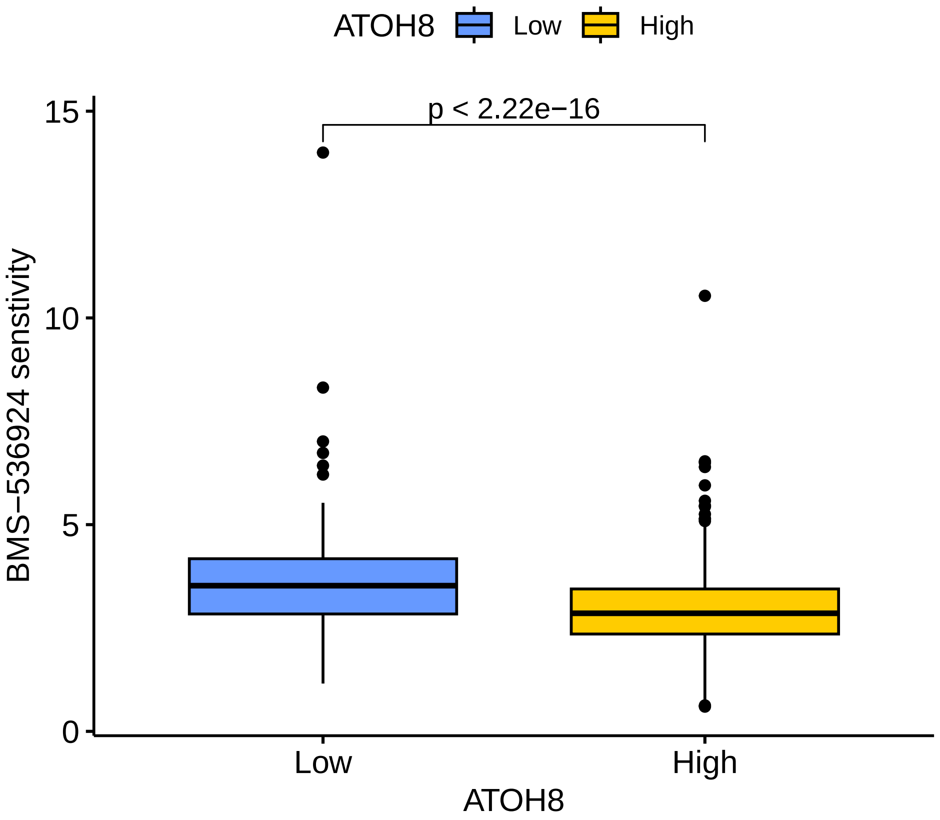
ATOH8
Low
High
p < 2.22e−16
15
10
5
0
BMS−536924 senstivity
Low
High
ATOH8

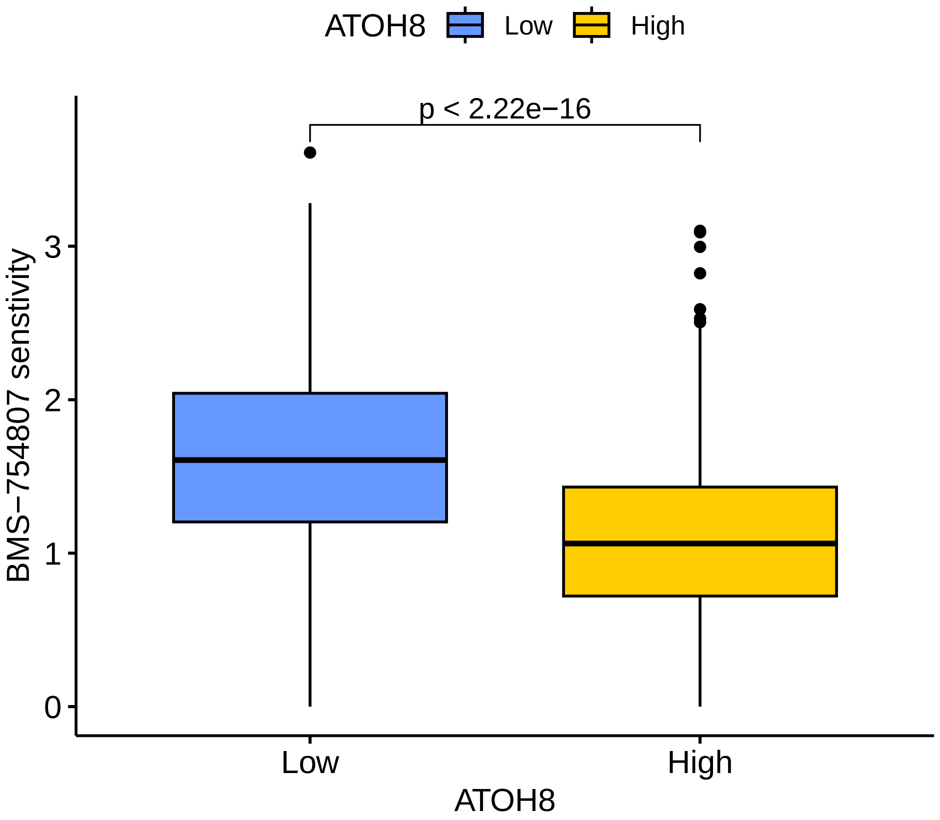

ATOH8
Low
High
p < 2.22e−16
BMS−754807 senstivity
0
1
2
3
Low
High
ATOH8

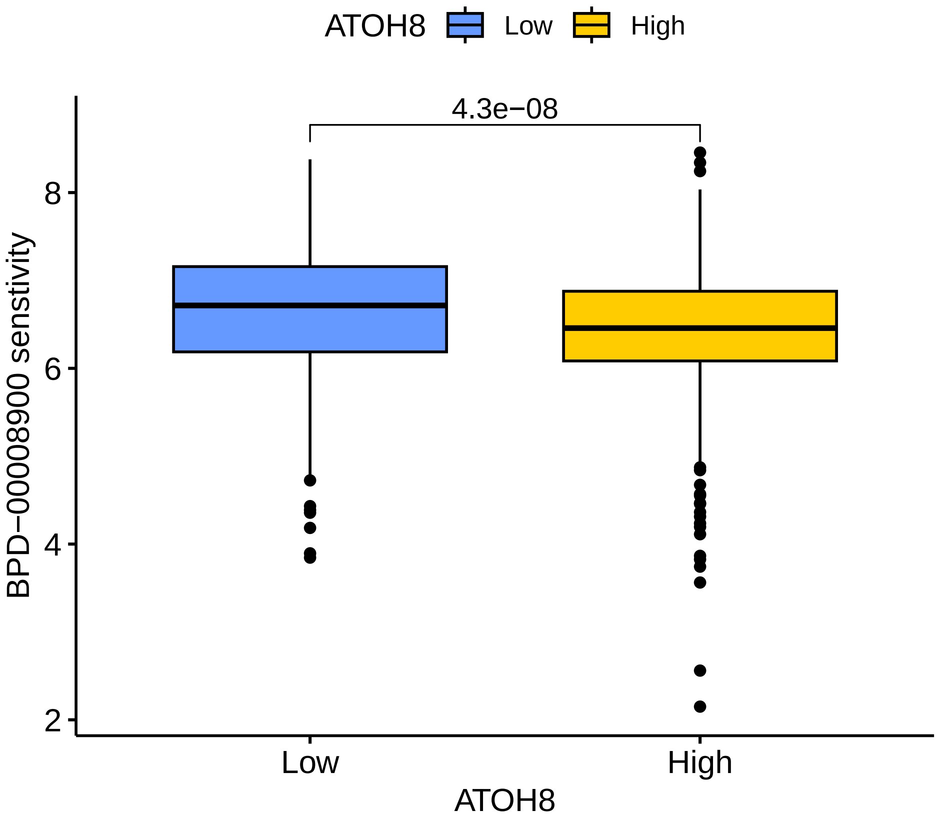
ATOH8
Low
High
4.3e−08
BPD−00008900 senstivity
8
6
4
2
Low
High
ATOH8

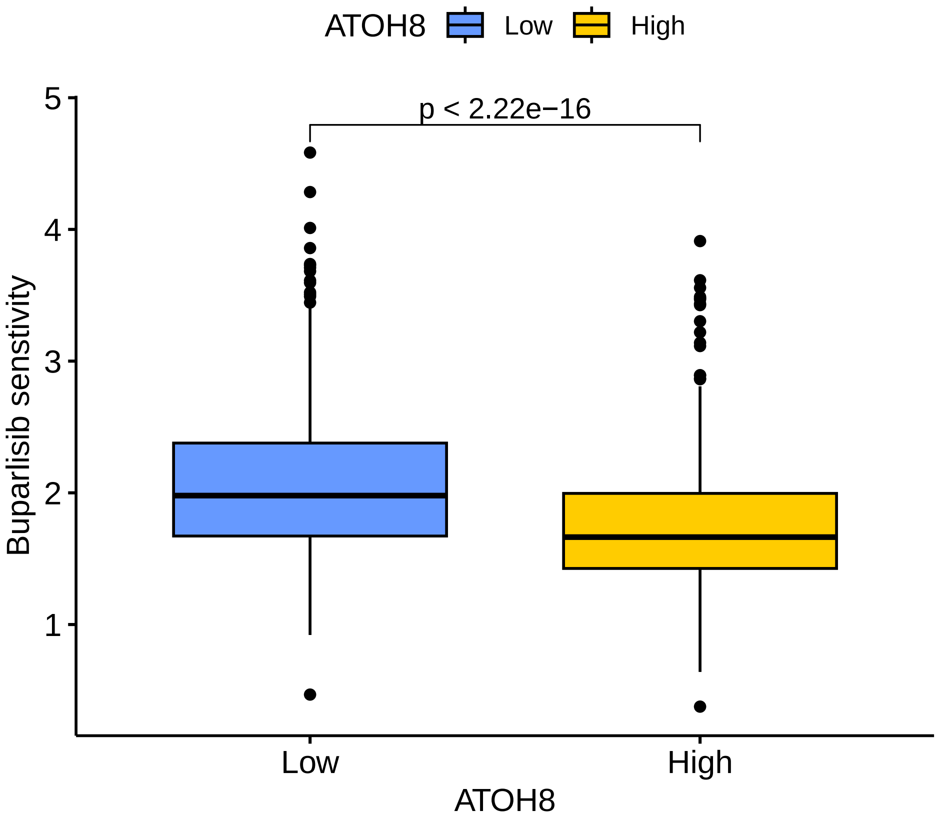
ATOH8
Low
High
p < 2.22e−16
Buparlisib senstivity
5
4
3
2
1
Low
High
ATOH8

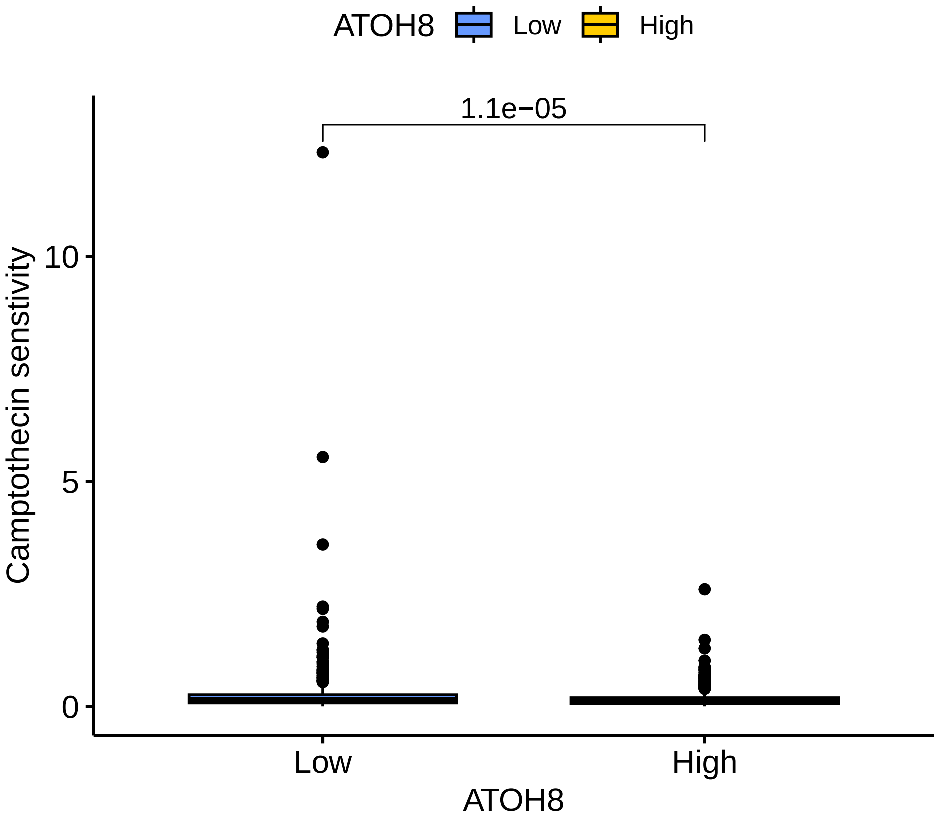
ATOH8
Low
High
1.1e−05
Camptothecin senstivity
10
5
0
Low
High
ATOH8

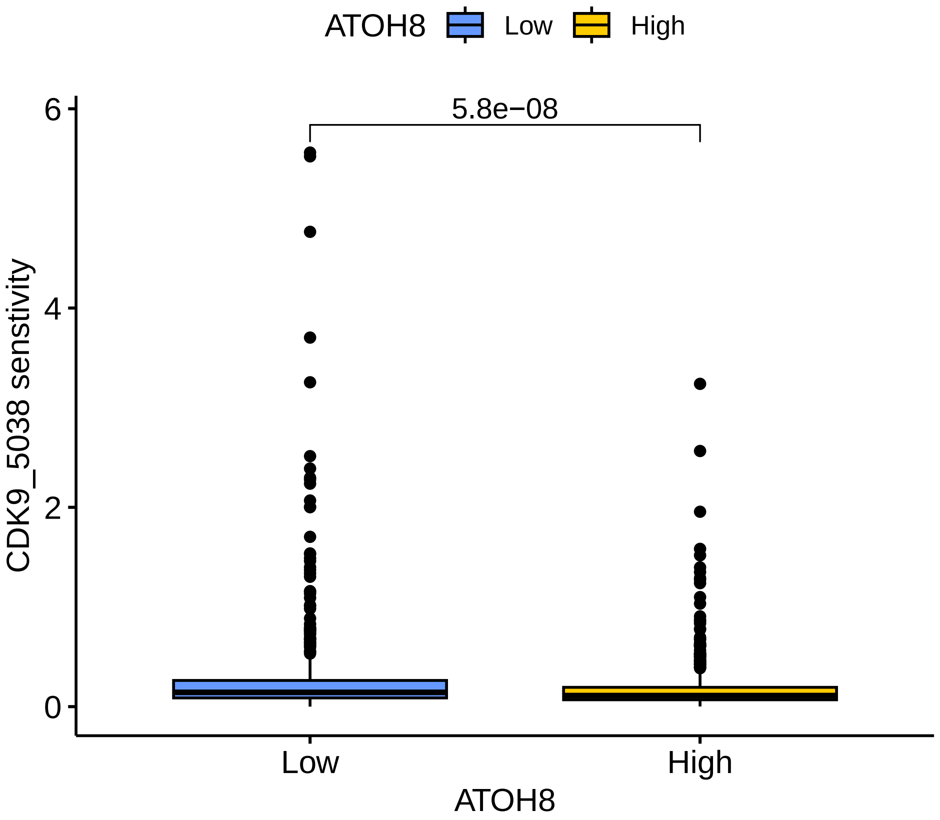

ATOH8
Low
High
5.8e−08
CDK9_5038 senstivity
6
4
2
0
Low
High
ATOH8

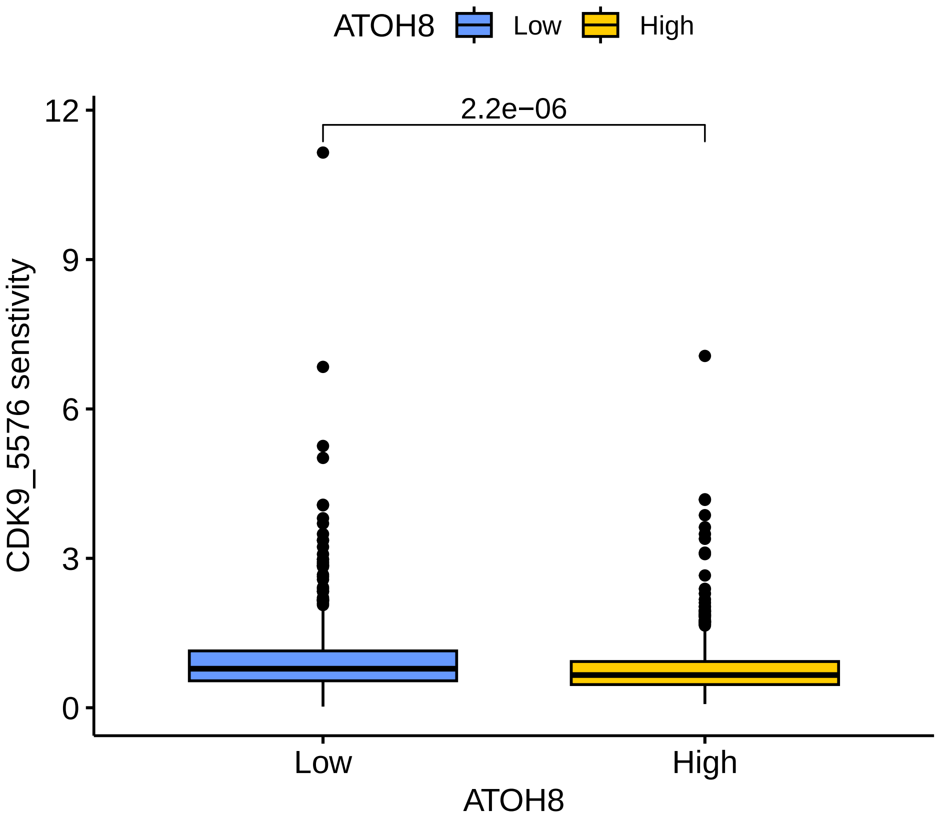

ATOH8
Low
High
2.2e−06
CDK9_5576 senstivity
12
9
6
3
0
Low
High
ATOH8

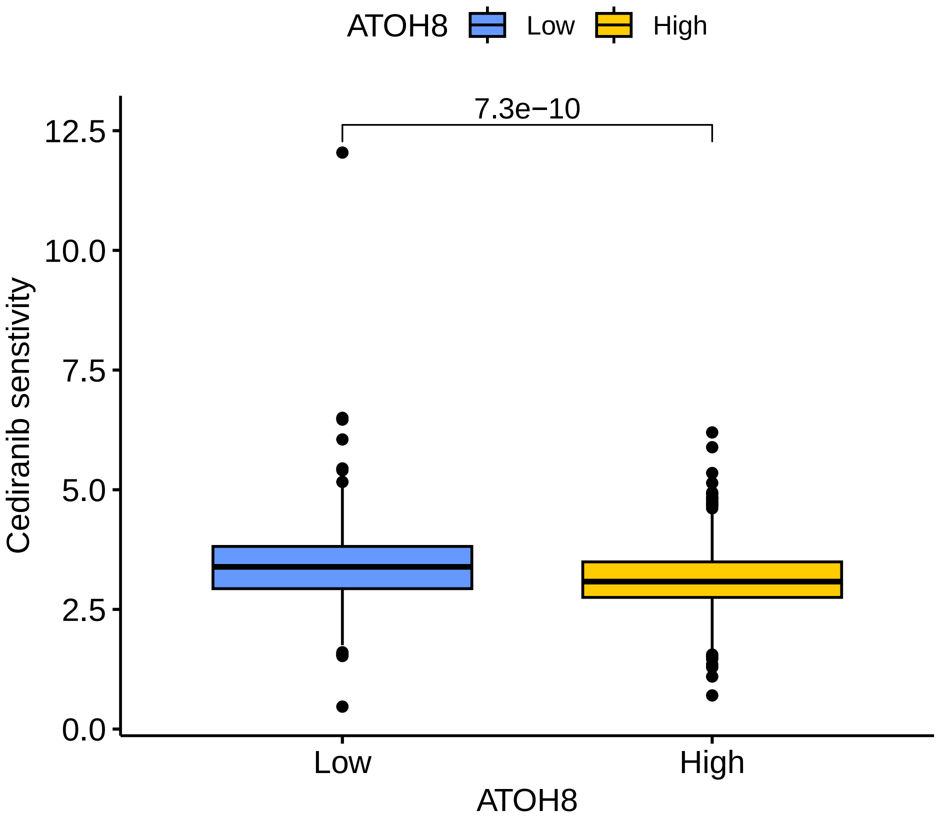
ATOH8
Low
High
7.3e−10
12.5
10.0
7.5
5.0
2.5
0.0
Cediranib senstivity
Low
High
ATOH8

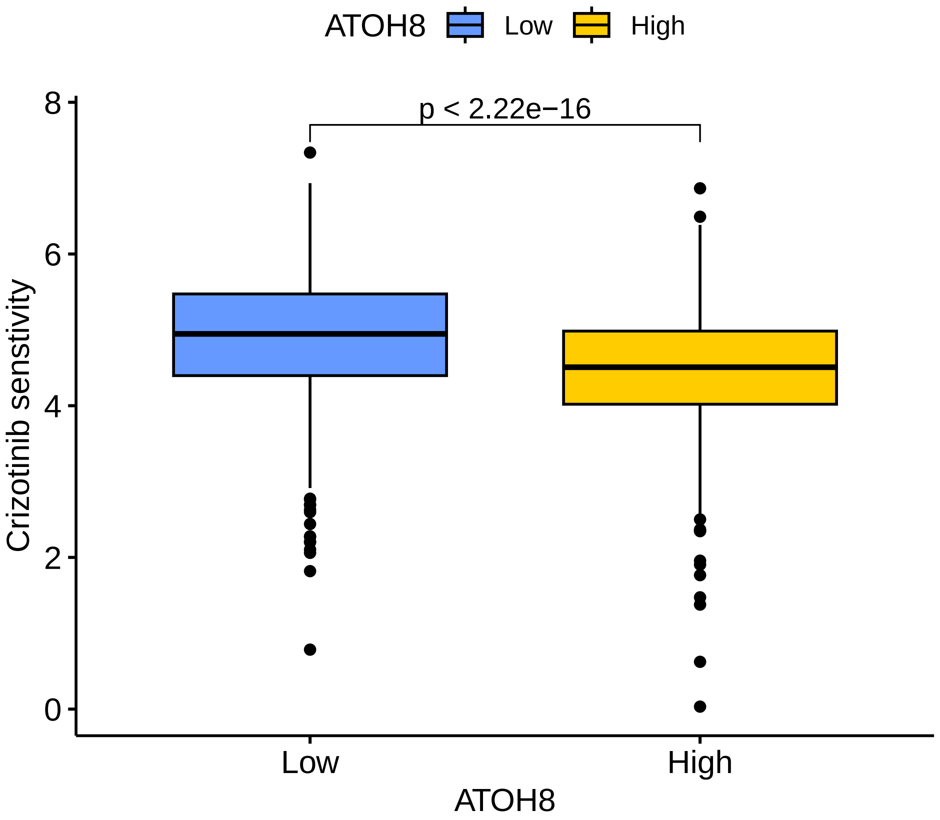

ATOH8
Low
High
p < 2.22e−16
8
6
4
2
0
Crizotinib senstivity
Low
High
ATOH8

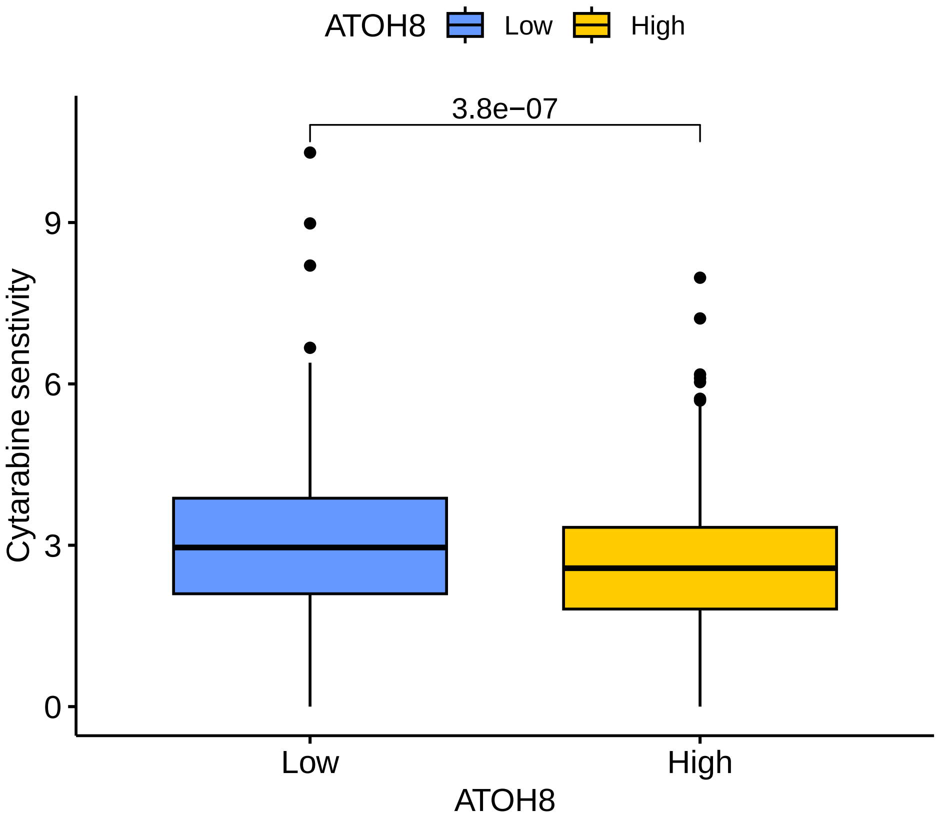

ATOH8
Low
High
3.8e−07
Cytarabine senstivity
9
6
3
0
Low
High
ATOH8

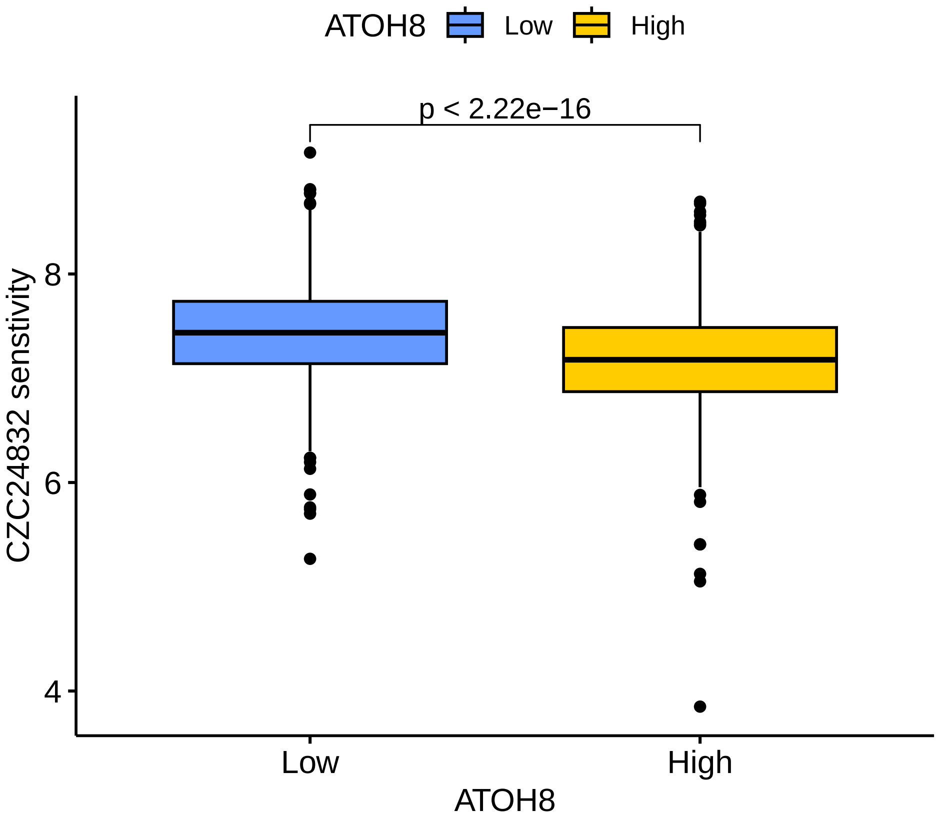
ATOH8
Low
High
p < 2.22e−16
CZC24832 senstivity
8
6
4
Low
High
ATOH8

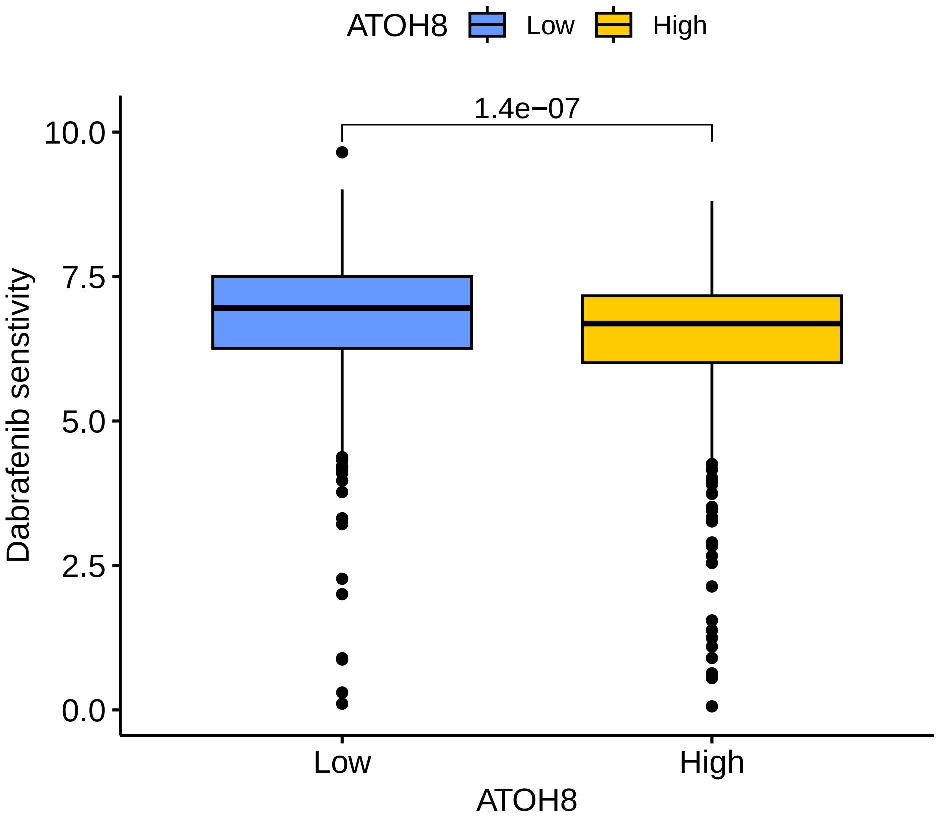

ATOH8
Low
High
1.4e−07
10.0
7.5
5.0
2.5
0.0
Dabrafenib senstivity
Low
High
ATOH8

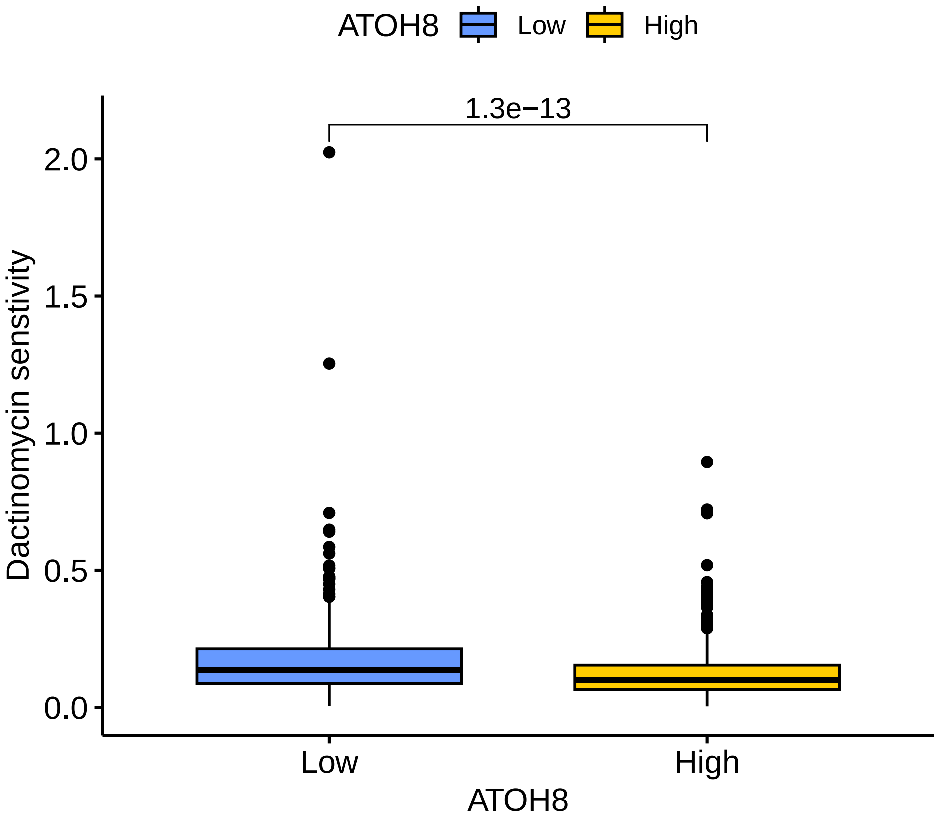

ATOH8
Low
High
1.3e−13
2.0
1.5
1.0
0.5
0.0
Dactinomycin senstivity
Low
High
ATOH8

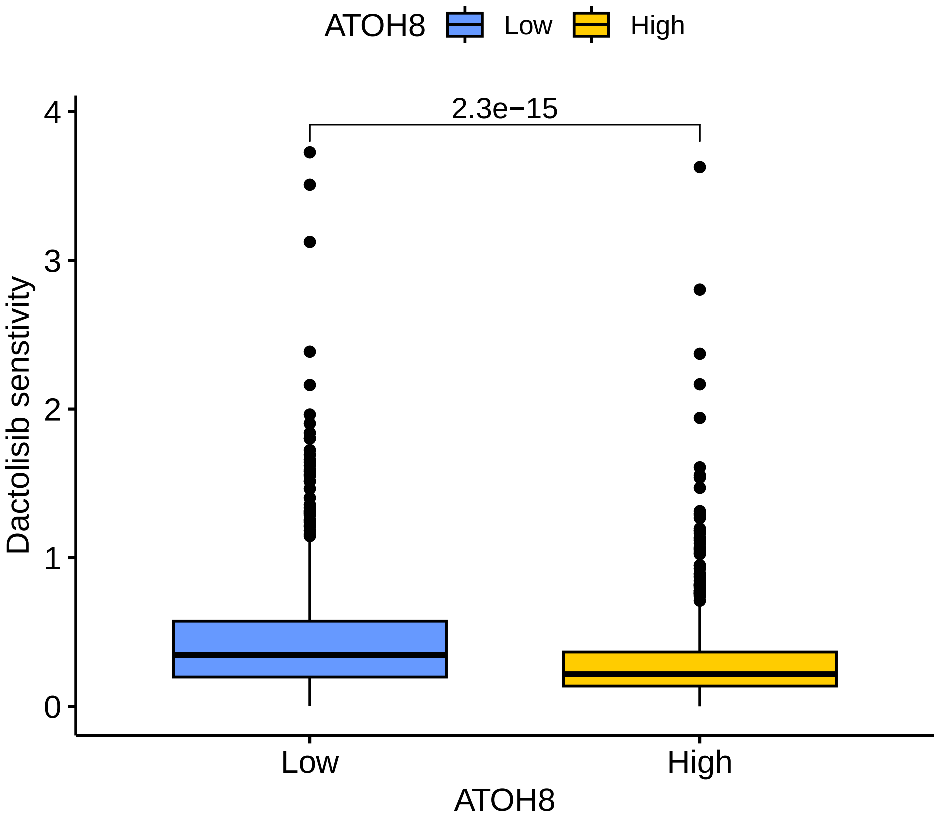
ATOH8
Low
High
2.3e−15
4
3
2
1
0
Dactolisib senstivity
Low
High
ATOH8

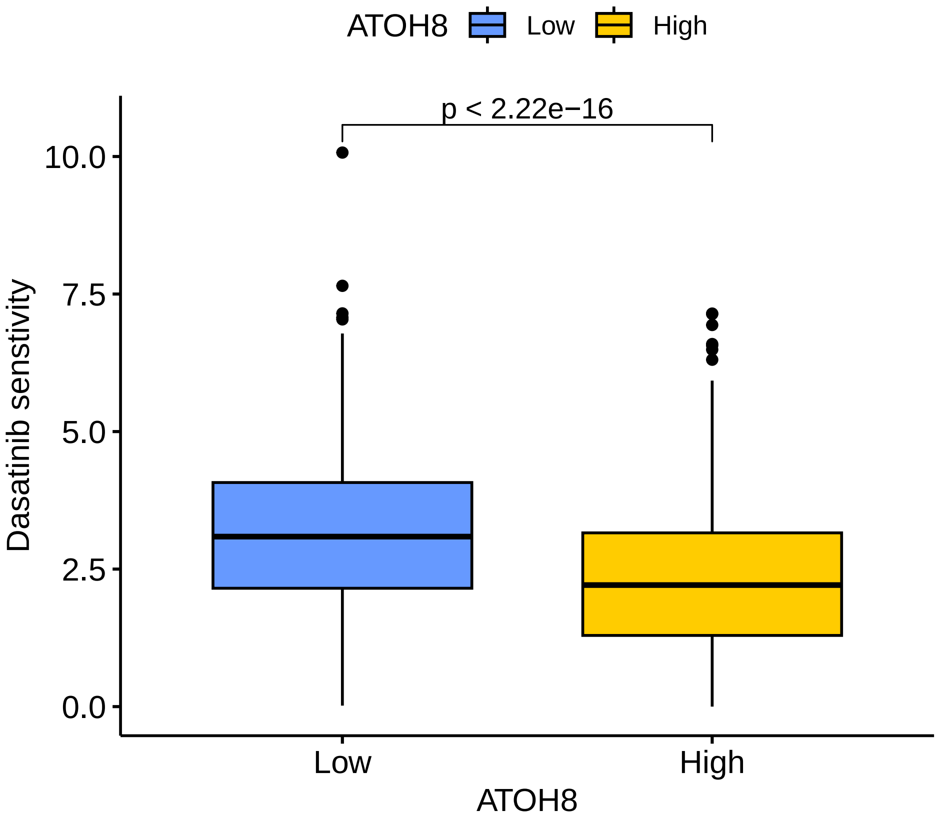

ATOH8
Low
High
p < 2.22e−16
10.0
7.5
5.0
2.5
0.0
Dasatinib senstivity
Low
High
ATOH8

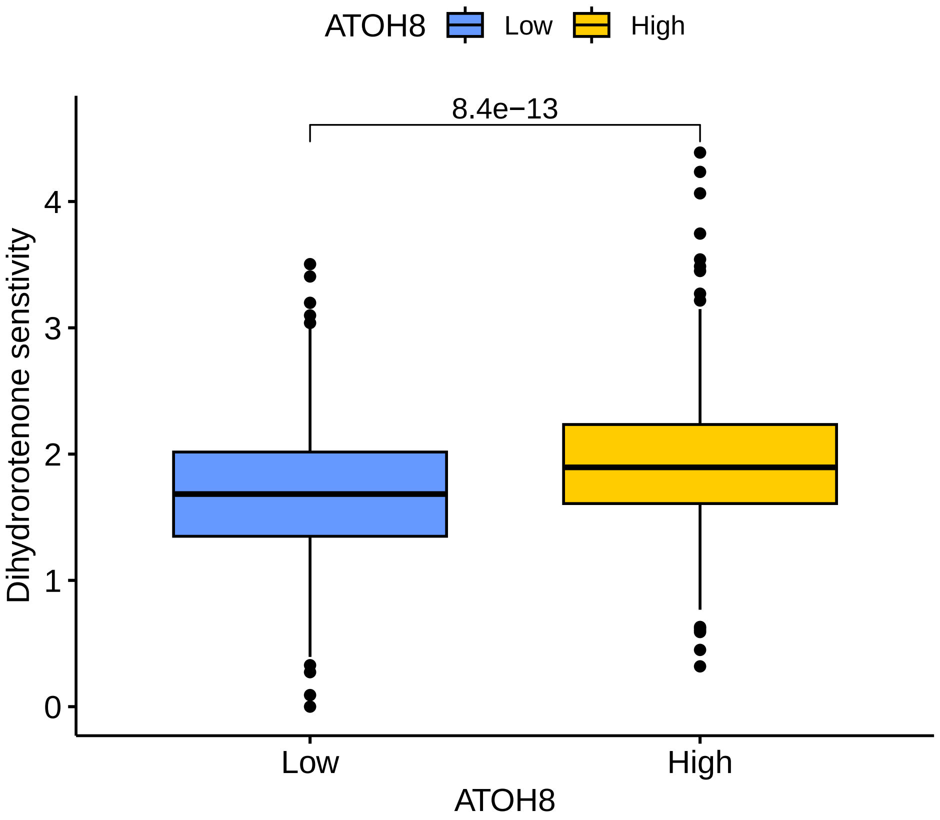

ATOH8
Low
High
8.4e−13
Dihydrorotenone senstivity
0
1
2
3
4
Low
High
ATOH8

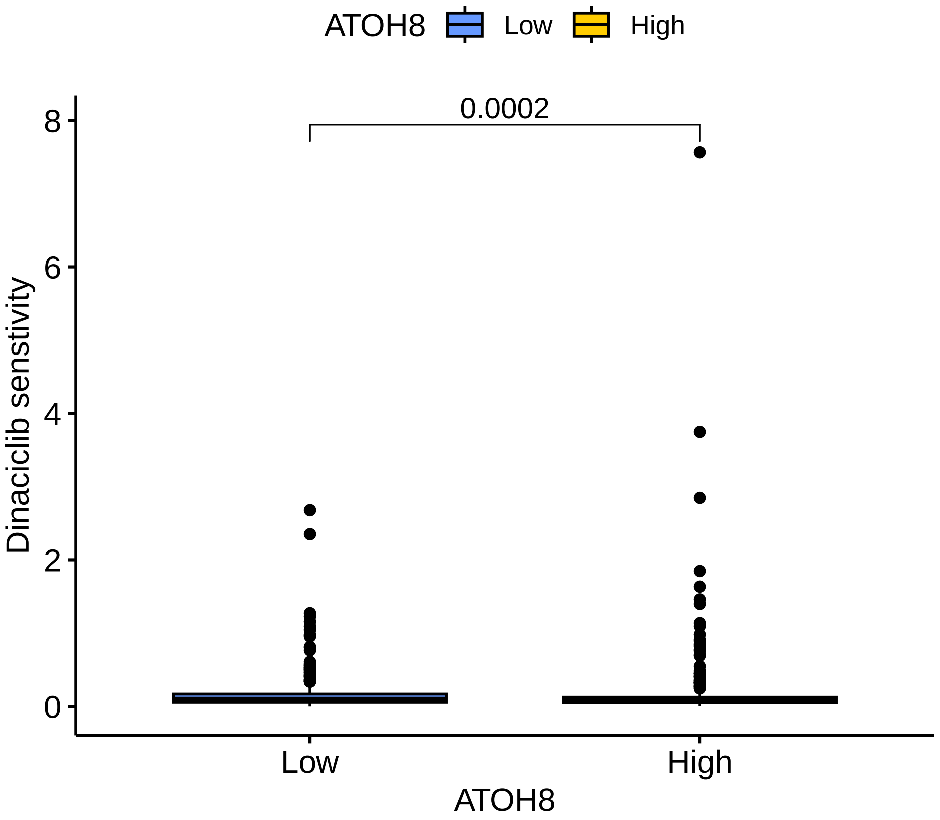
ATOH8
Low
High
0.0002
8
6
4
2
0
Dinaciclib senstivity
Low
High
ATOH8

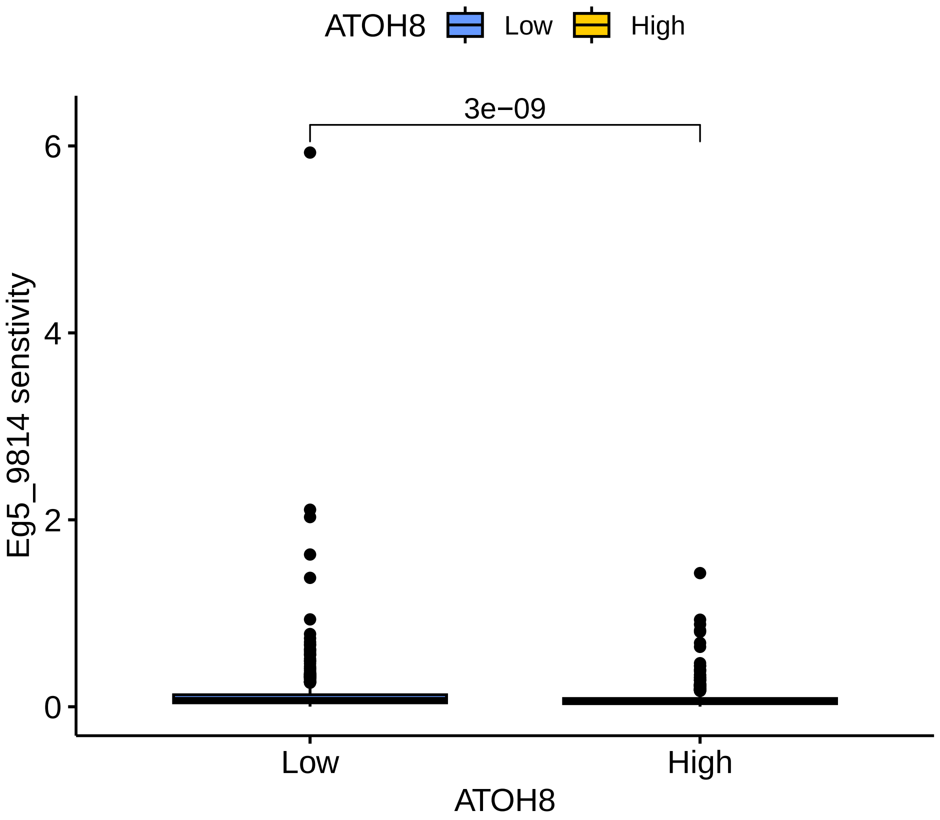

ATOH8
Low
High
3e−09
Eg5_9814 senstivity
6
4
2
0
Low
High
ATOH8

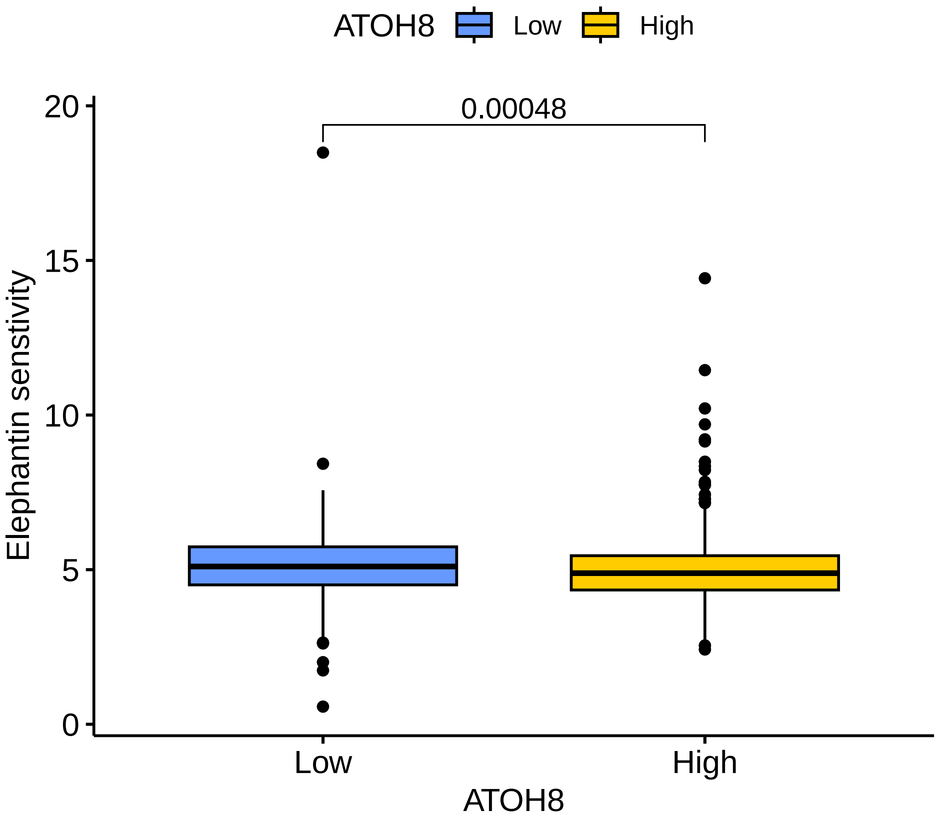
ATOH8
Low
High
0.00048
Elephantin senstivity
20
15
10
5
0
Low
High
ATOH8

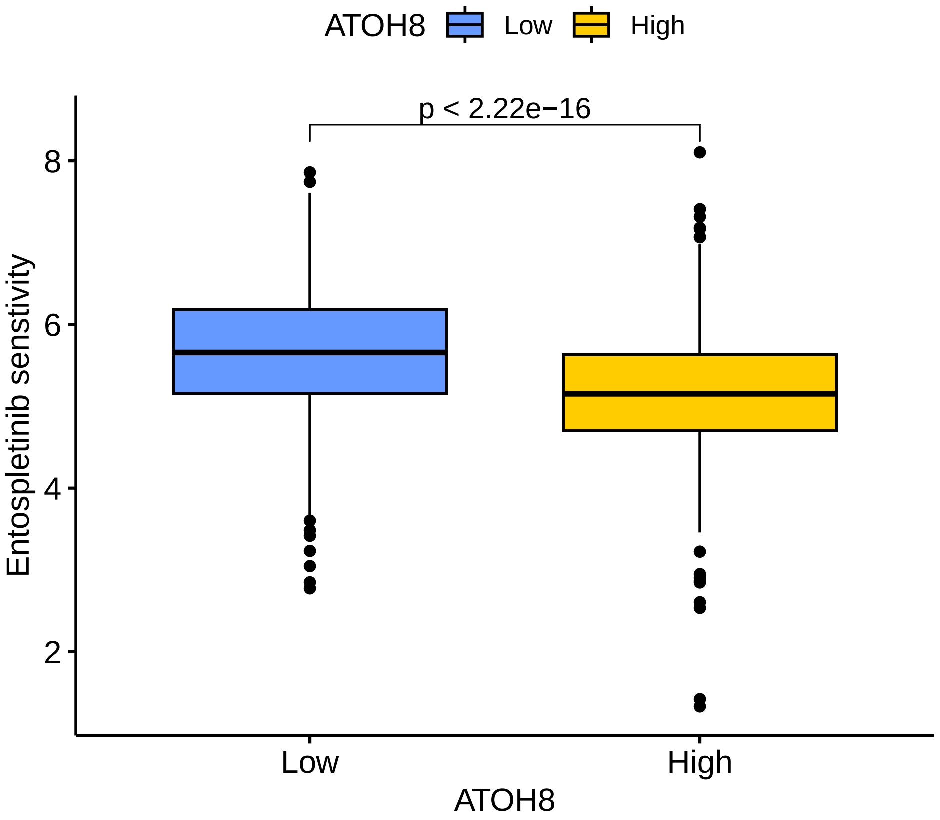

ATOH8
Low
High
p < 2.22e−16
8
6
4
2
Entospletinib senstivity
Low
High
ATOH8

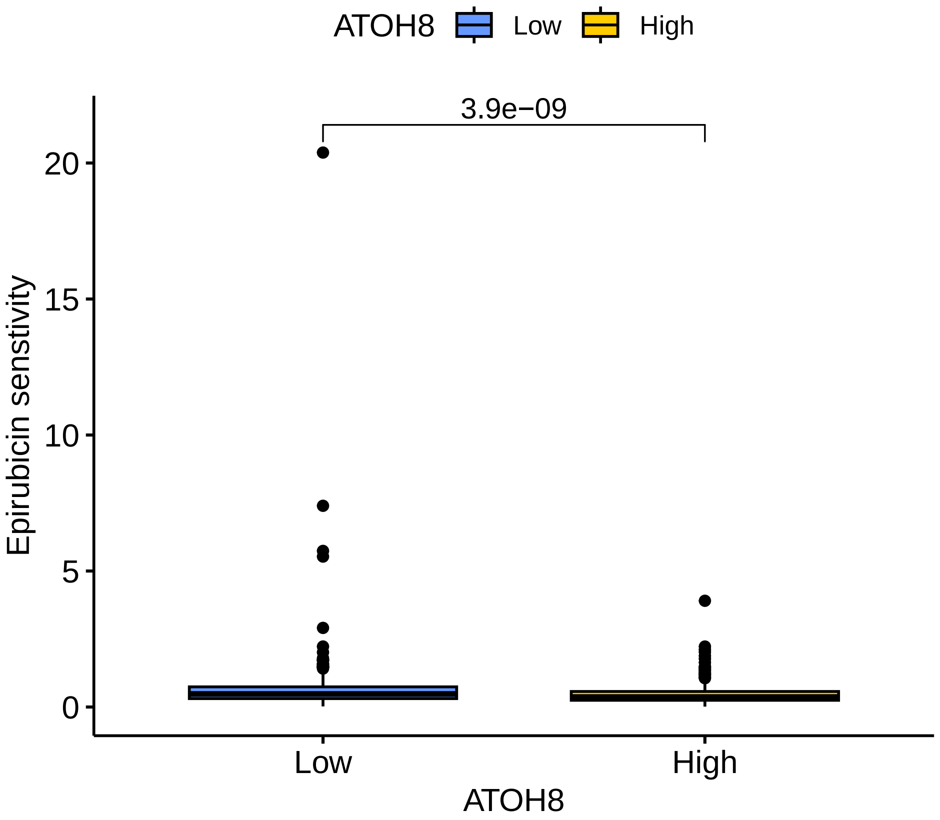
ATOH8
Low
High
3.9e−09
20
15
10
5
0
Epirubicin senstivity
Low
High
ATOH8

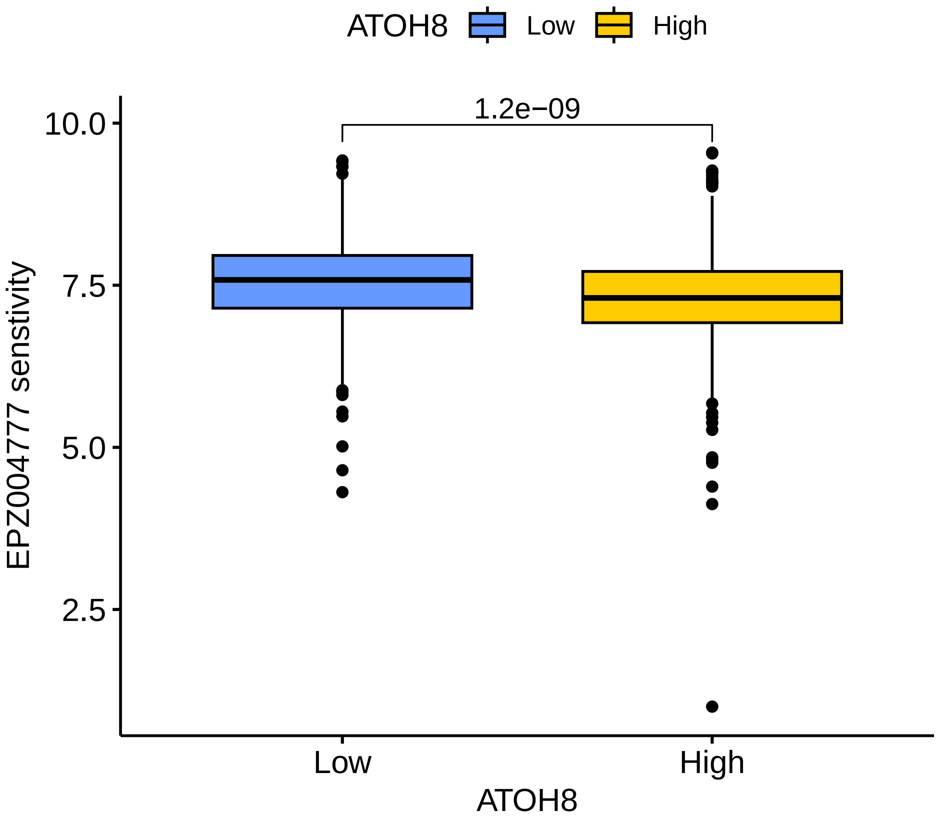
ATOH8
Low
High
1.2e−09
10.0
7.5
5.0
2.5
EPZ004777 senstivity
Low
High
ATOH8

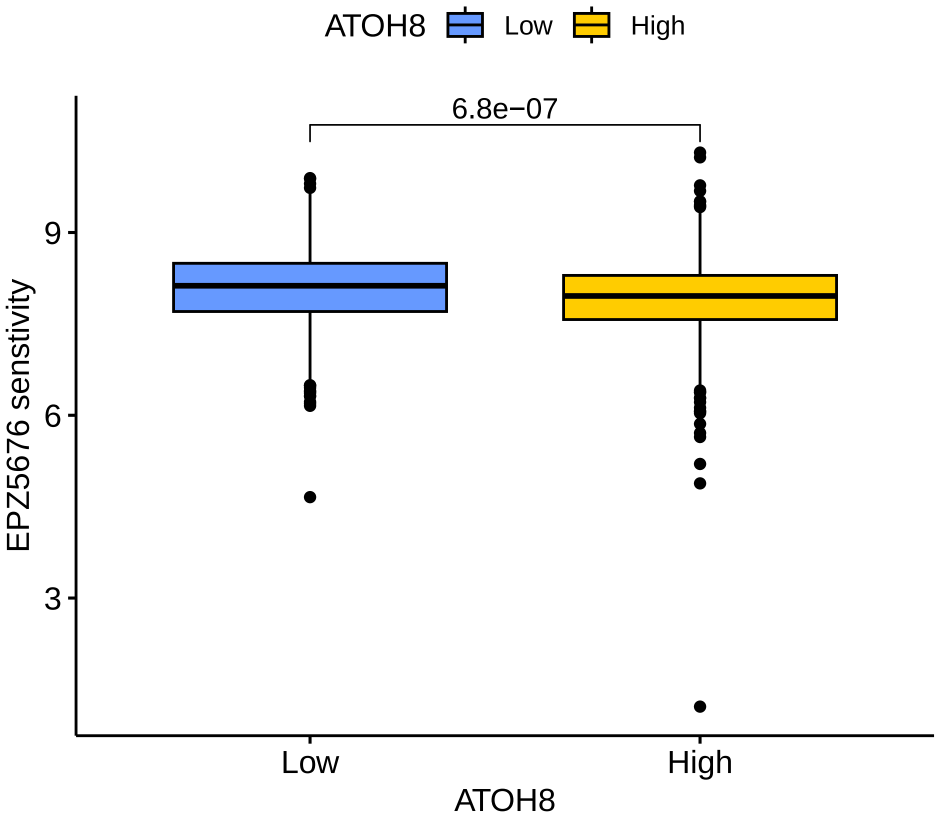
ATOH8
Low
High
6.8e−07
9
6
3
EPZ5676 senstivity
Low
High
ATOH8

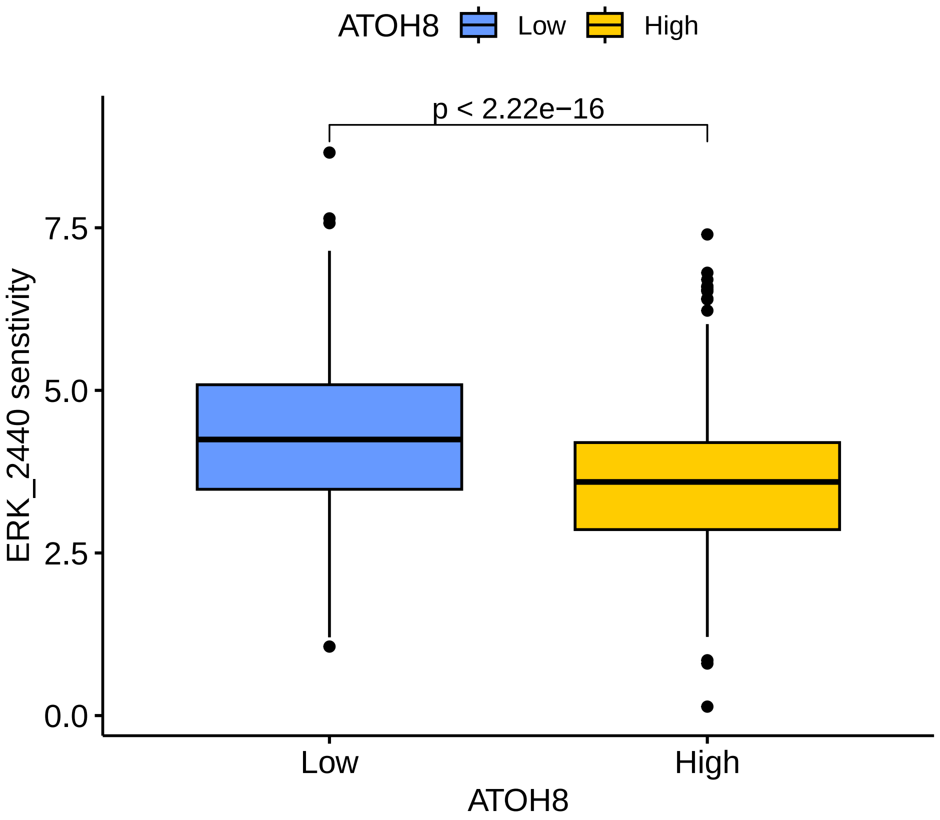

ATOH8
Low
High
p < 2.22e−16
ERK_2440 senstivity
7.5
5.0
2.5
0.0
Low
High
ATOH8

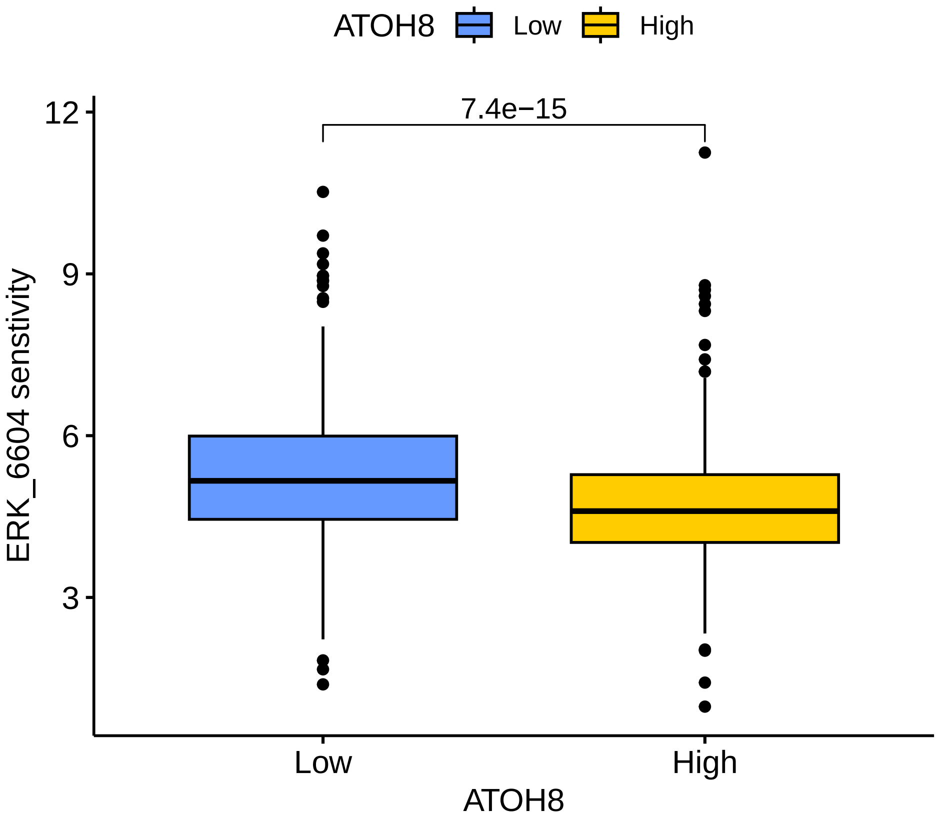
ATOH8
Low
High
7.4e−15
12
9
6
3
ERK_6604 senstivity
Low
High
ATOH8

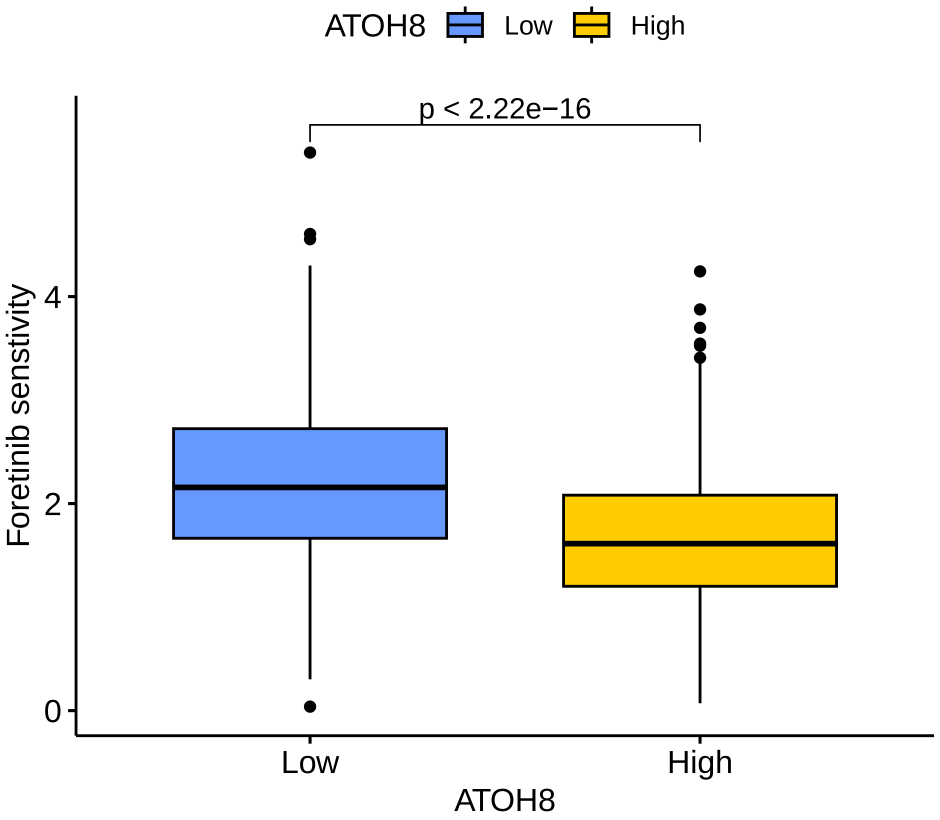

ATOH8
Low
High
p < 2.22e−16
Foretinib senstivity
4
2
0
Low
High
ATOH8

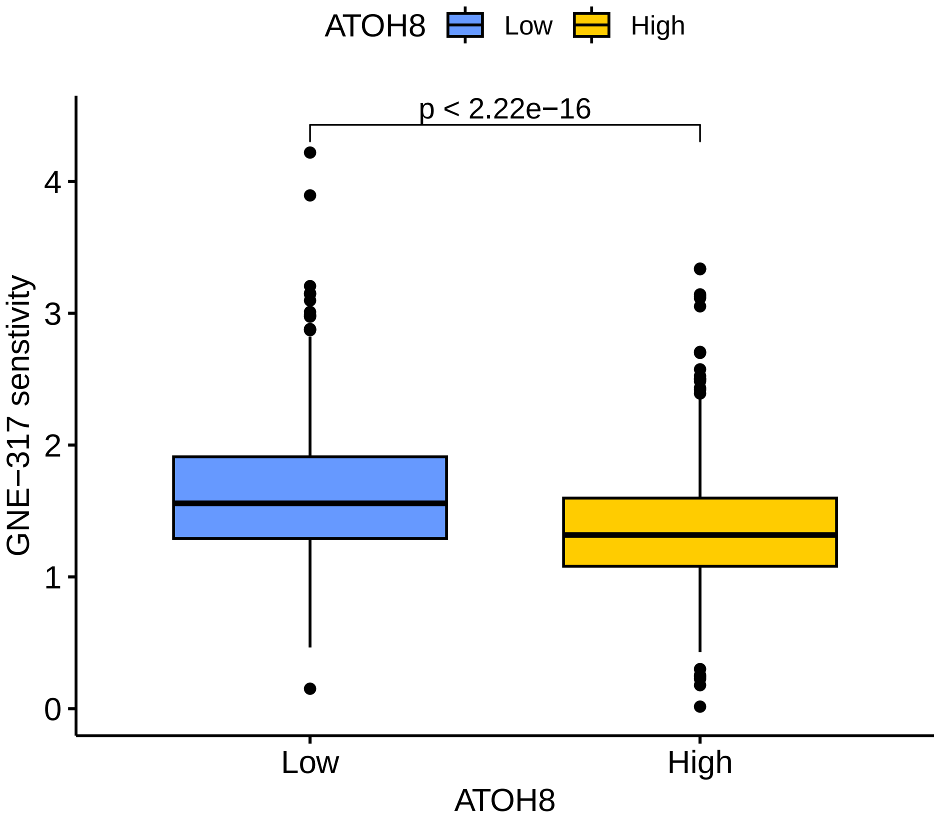

ATOH8
Low
High
p < 2.22e−16
GNE−317 senstivity
0
1
2
3
4
Low
High
ATOH8

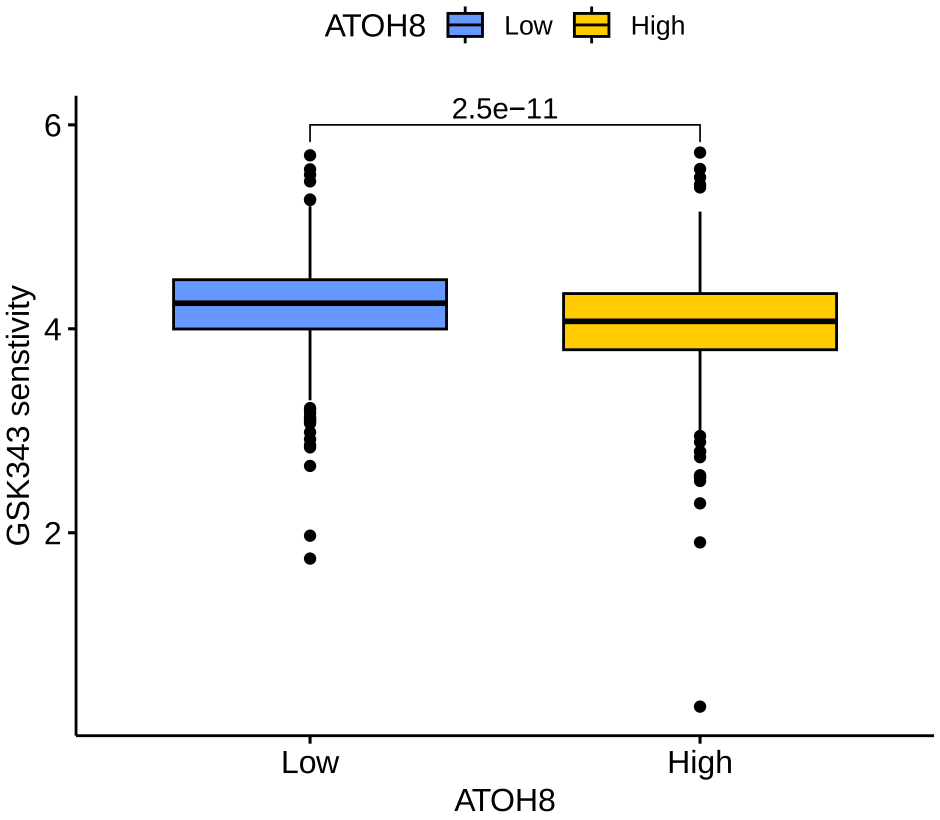
ATOH8
Low
High
2.5e−11
6
4
2
GSK343 senstivity
Low
High
ATOH8

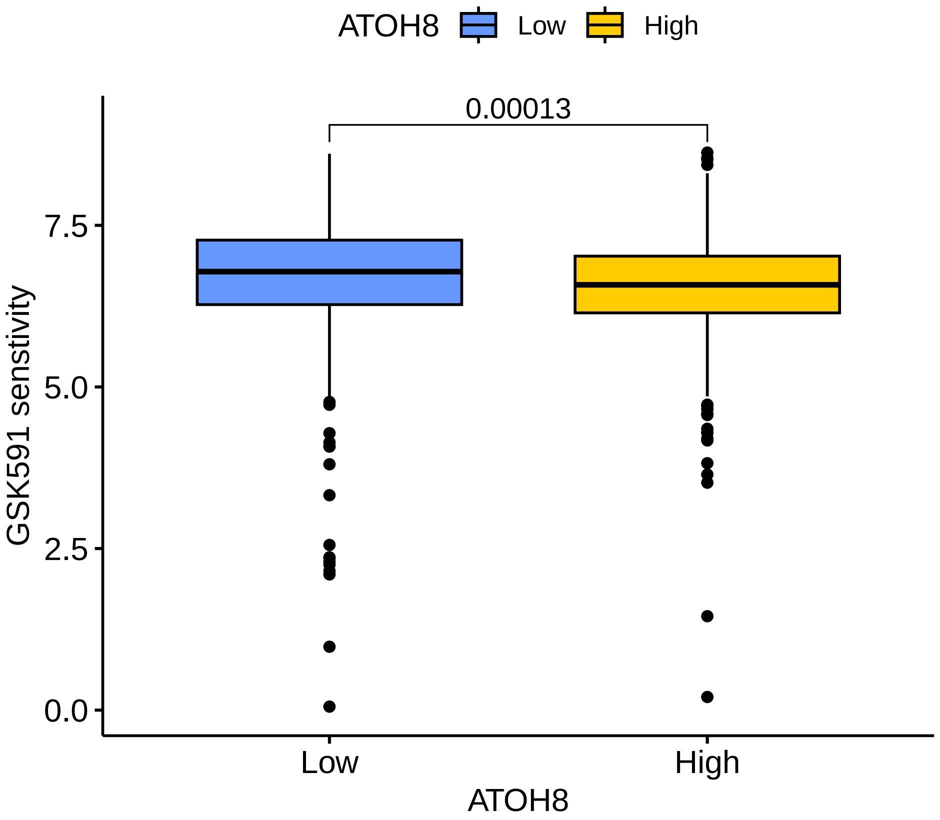
ATOH8
Low
High
0.00013
7.5
5.0
2.5
0.0
GSK591 senstivity
Low
High
ATOH8

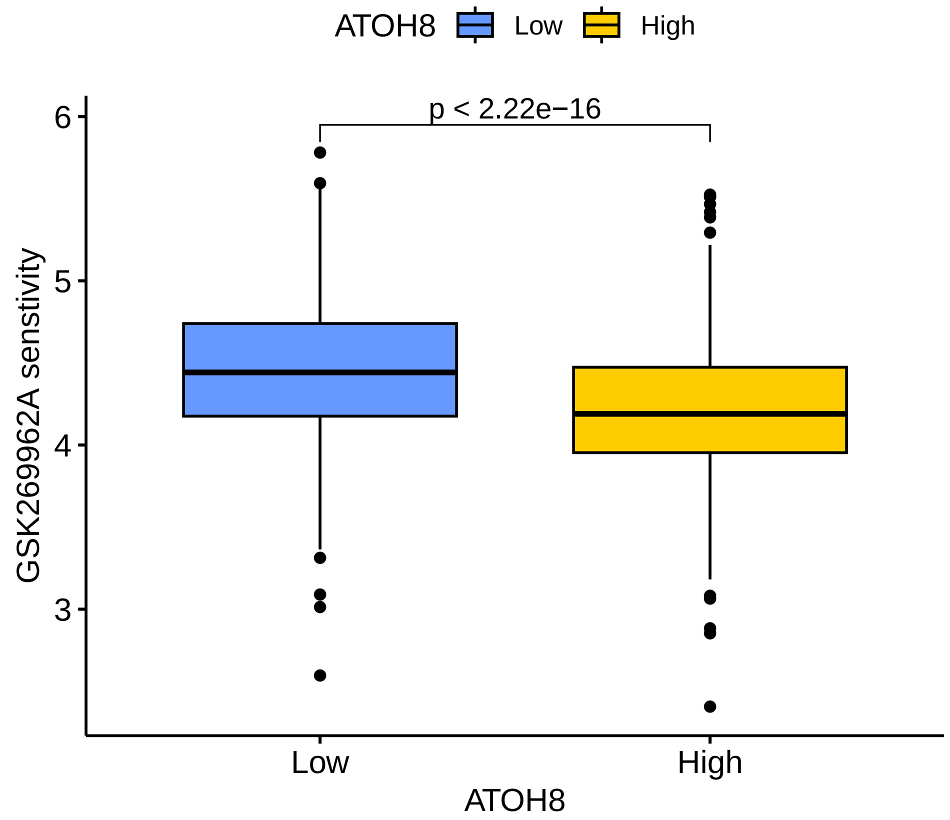

ATOH8
Low
High
p < 2.22e−16
GSK269962A senstivity
6
5
4
3
Low
High
ATOH8

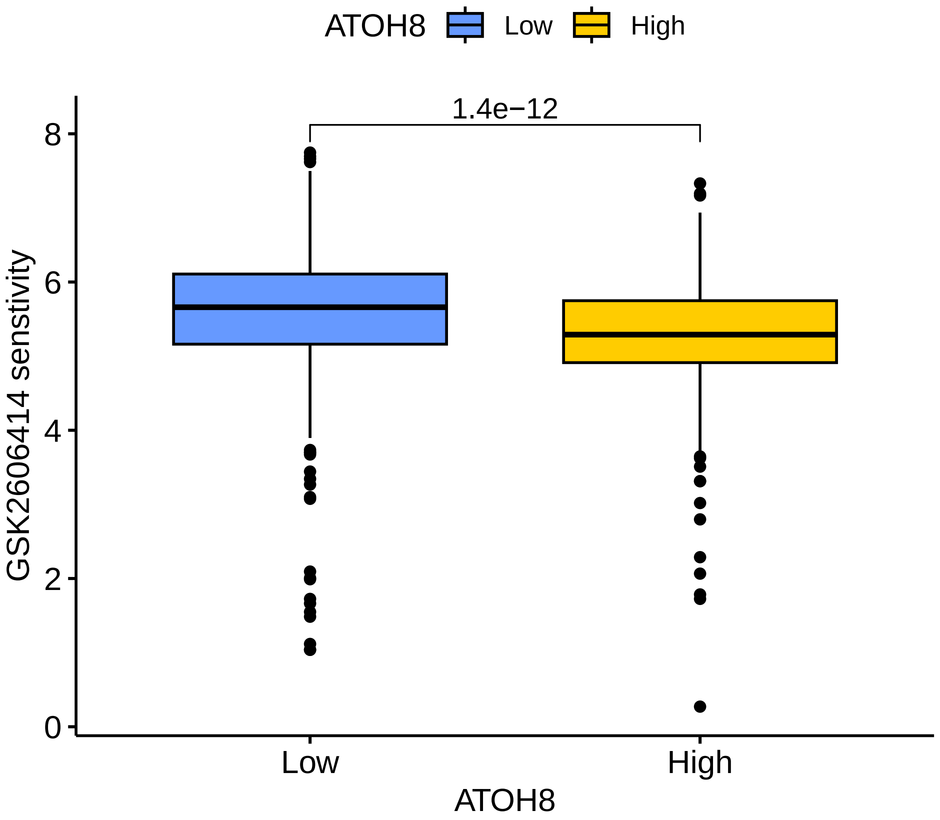
ATOH8
Low
High
1.4e−12
8
6
4
2
0
GSK2606414 senstivity
Low
High
ATOH8

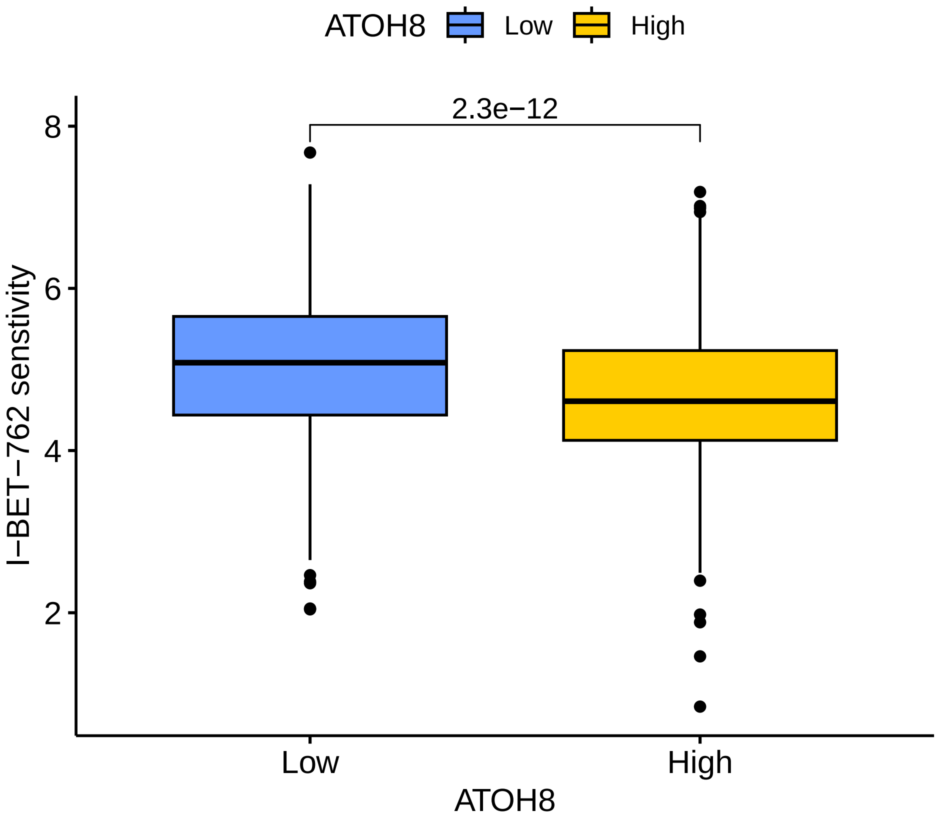

ATOH8
Low
High
2.3e−12
I−BET−762 senstivity
8
6
4
2
Low
High
ATOH8

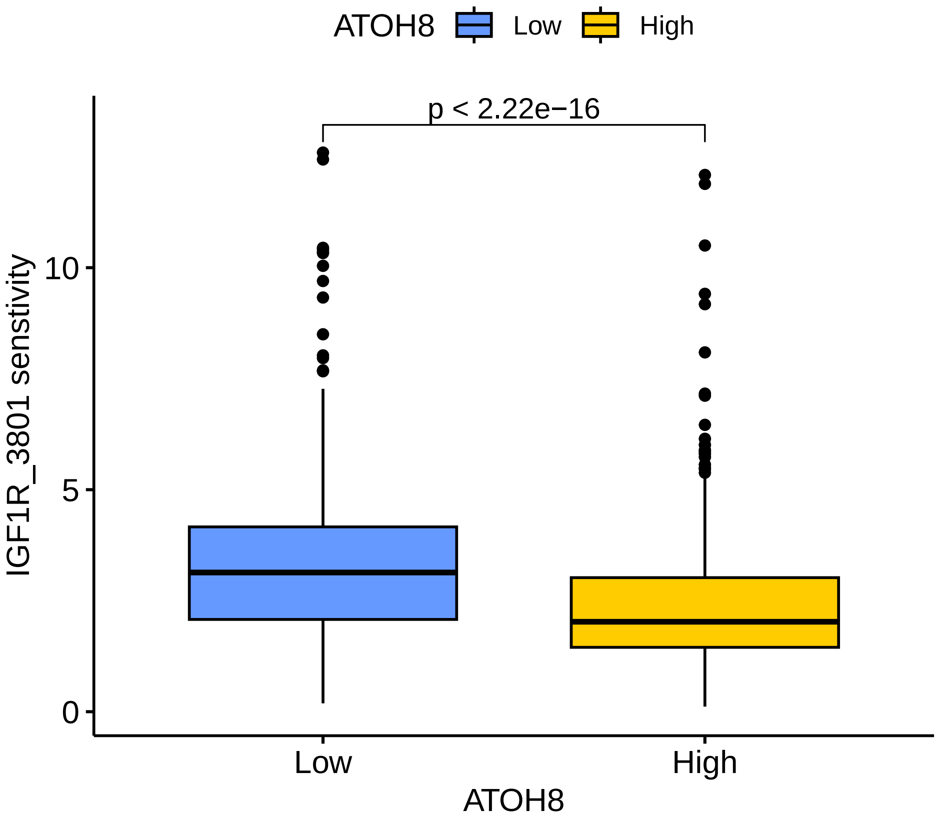
ATOH8
Low
High
p < 2.22e−16
IGF1R_3801 senstivity
10
5
0
Low
High
ATOH8

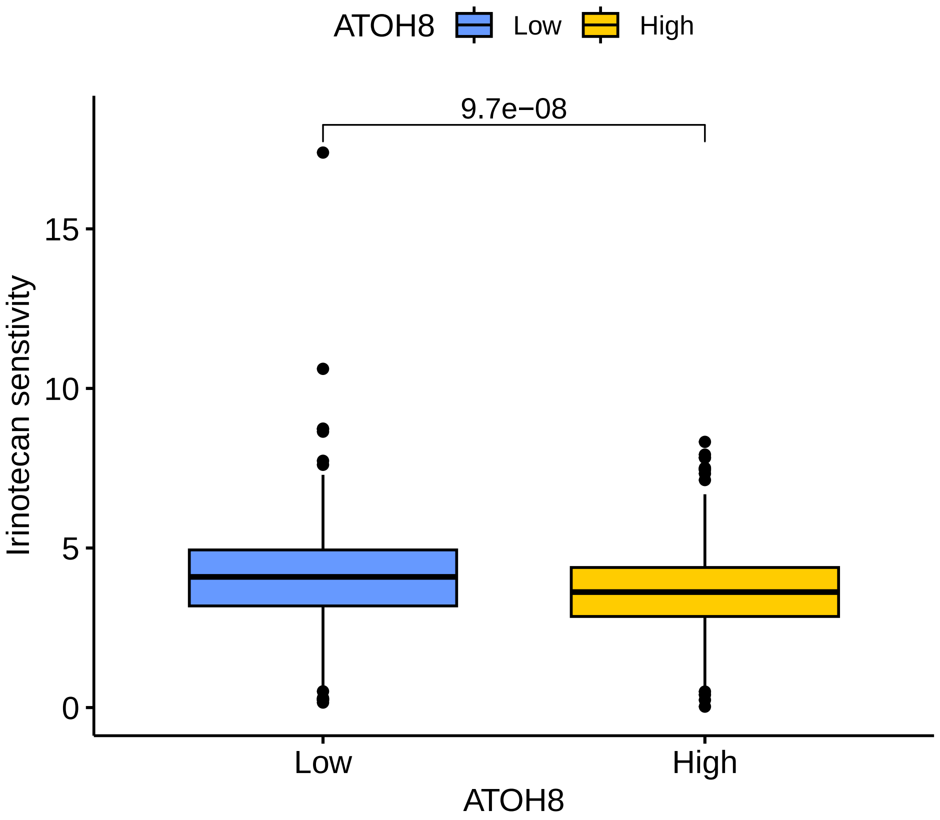
ATOH8
Low
High
9.7e−08
Irinotecan senstivity
15
10
5
0
Low
High
ATOH8

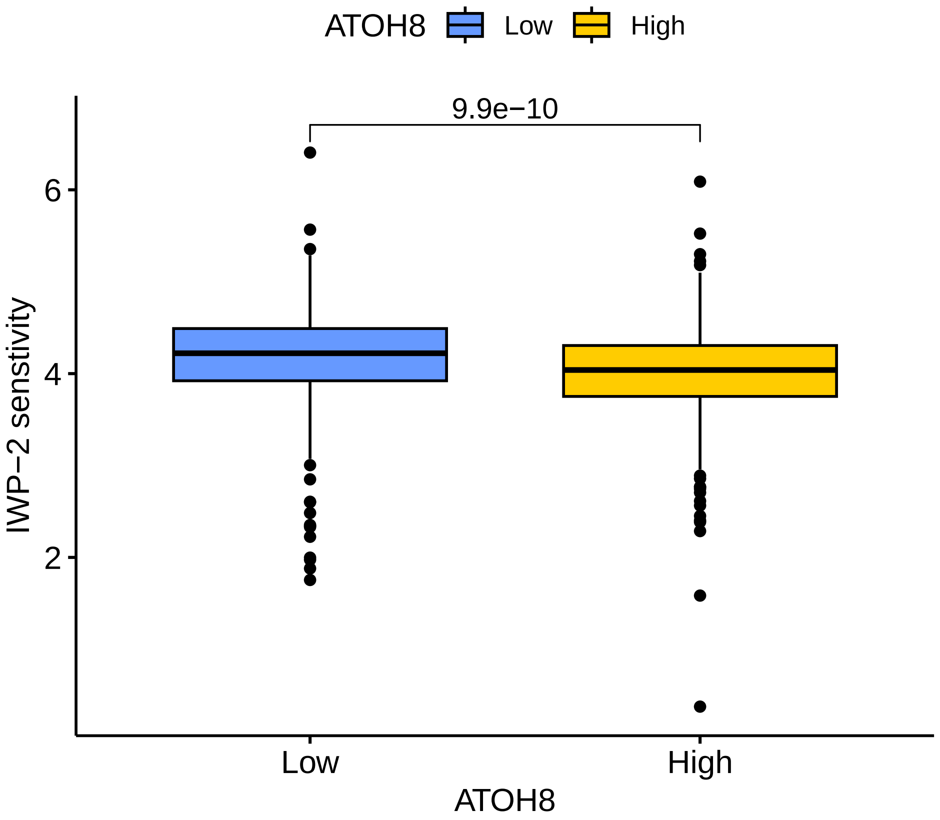

ATOH8
Low
High
9.9e−10
6
4
2
IWP−2 senstivity
Low
High
ATOH8

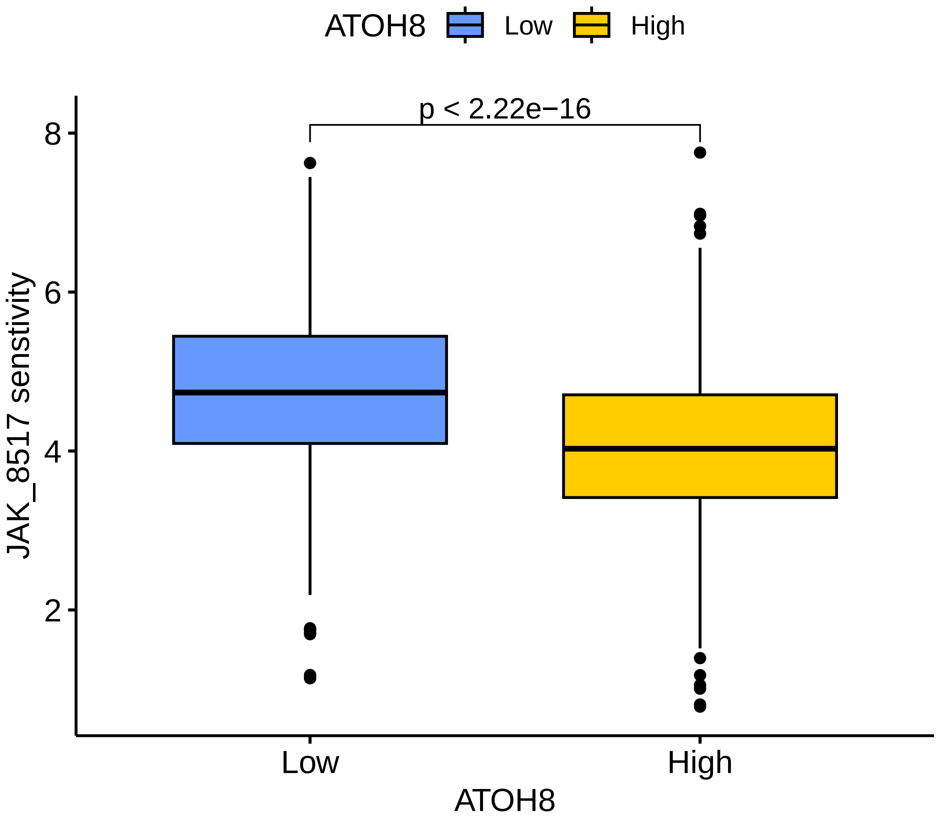

ATOH8
Low
High
p < 2.22e−16
8
6
4
2
JAK_8517 senstivity
Low
High
ATOH8

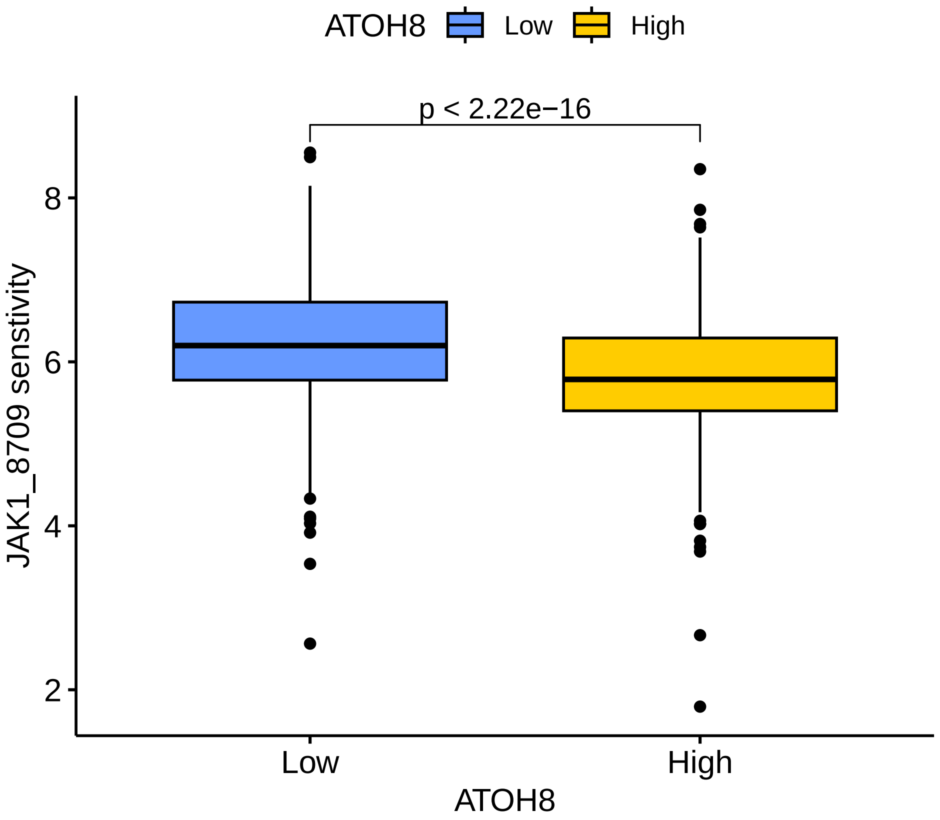

ATOH8
Low
High
p < 2.22e−16
8
6
4
2
JAK1_8709 senstivity
Low
High
ATOH8

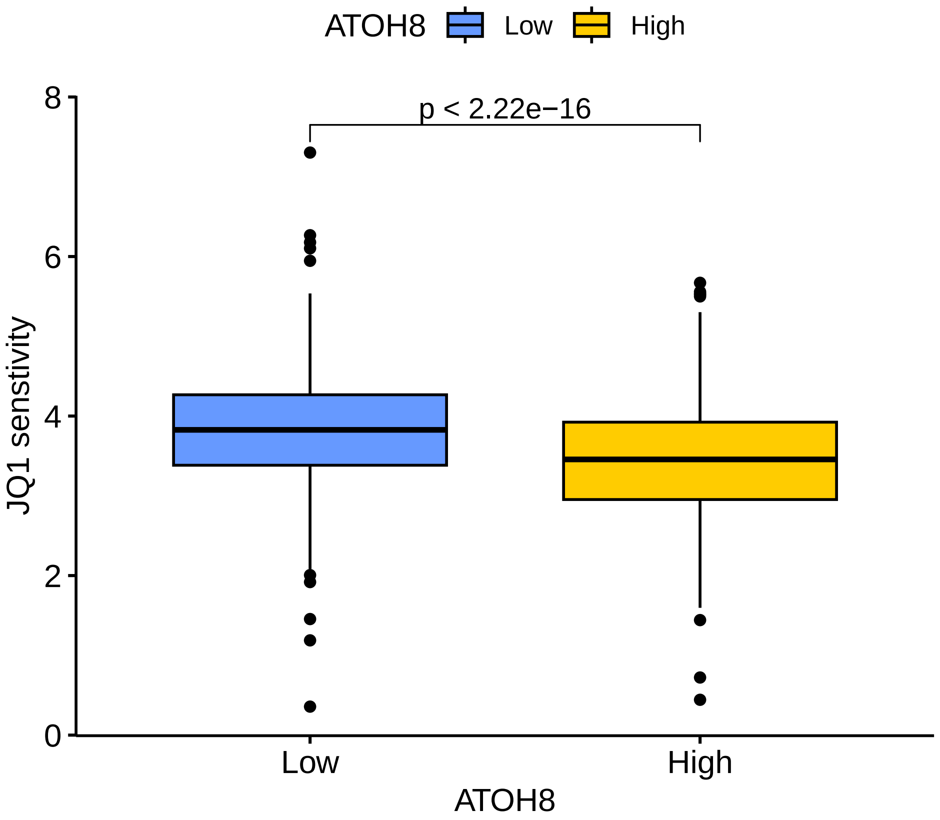

ATOH8
Low
High
p < 2.22e−16
JQ1 senstivity
8
6
4
2
0
Low
High
ATOH8

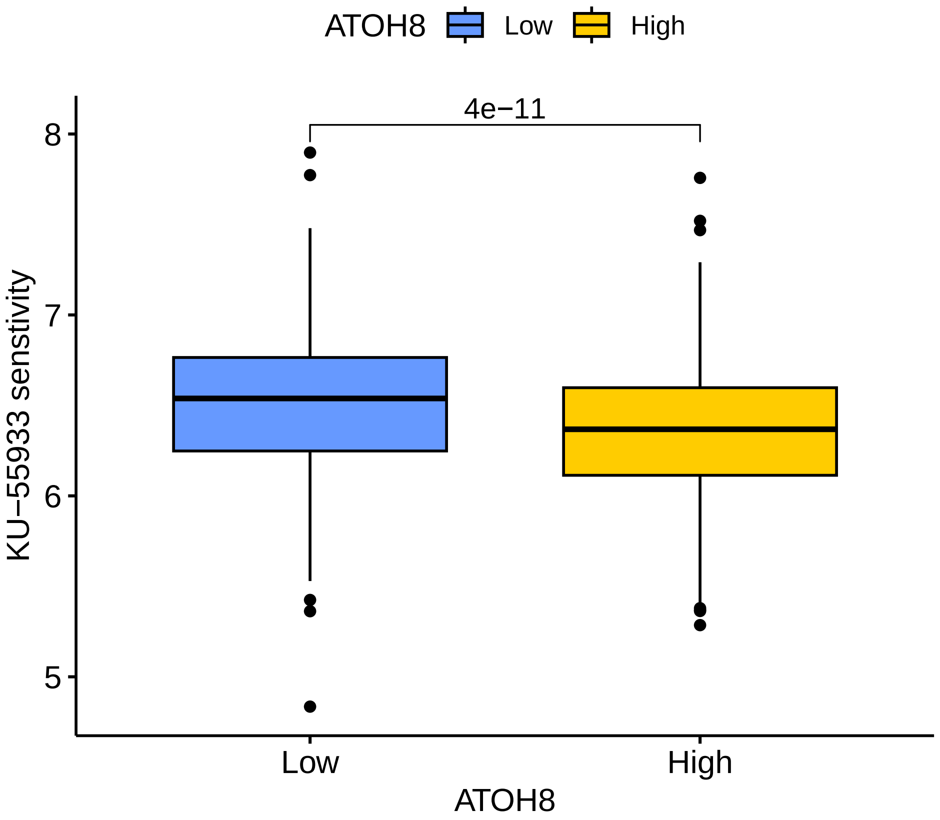
ATOH8
Low
High
4e−11
8
7
6
5
KU−55933 senstivity
Low
High
ATOH8

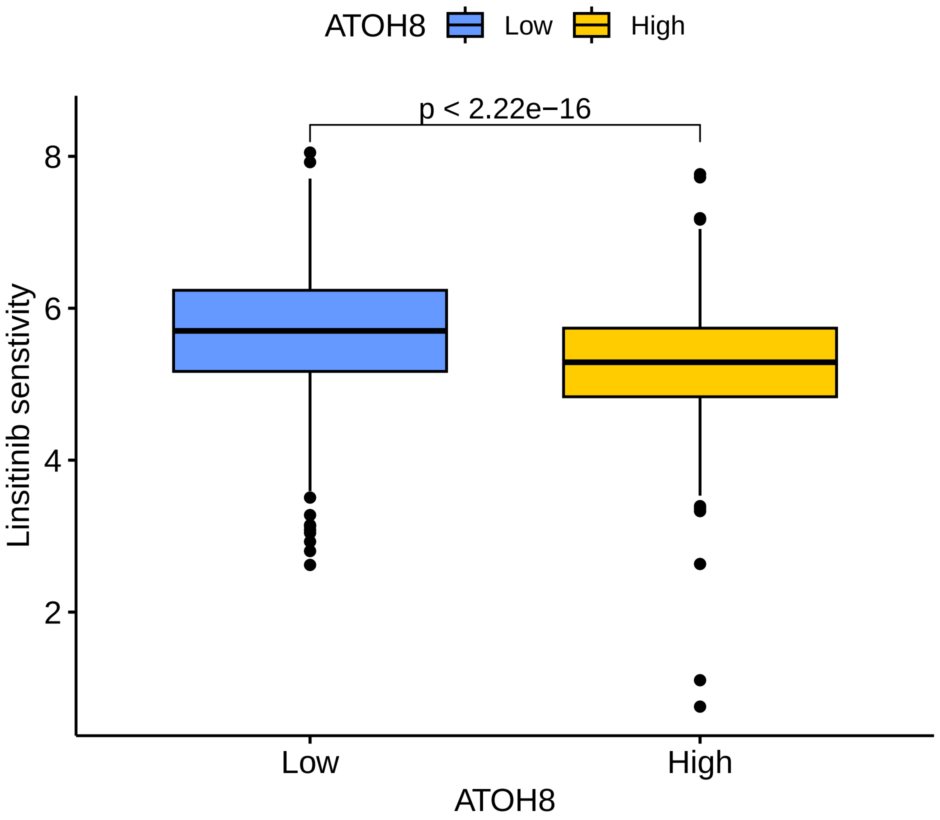
ATOH8
Low
High
p < 2.22e−16
8
6
4
2
Linsitinib senstivity
Low
High
ATOH8

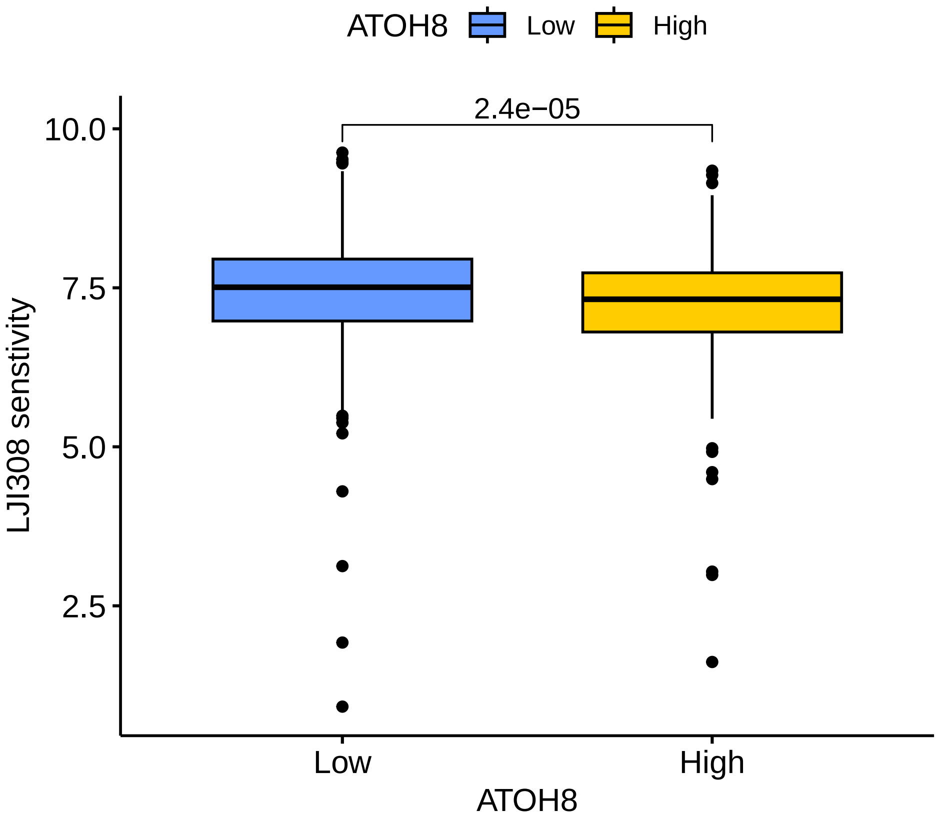

ATOH8
Low
High
2.4e−05
10.0
7.5
5.0
2.5
LJI308 senstivity
Low
High
ATOH8

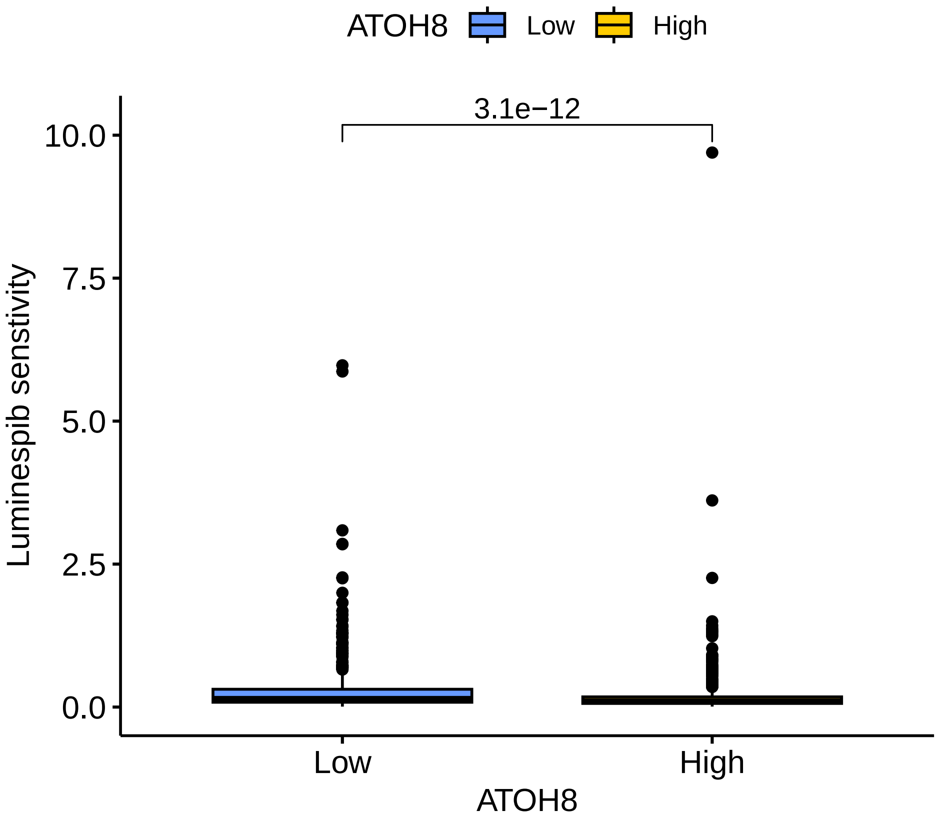
ATOH8
Low
High
3.1e−12
10.0
7.5
5.0
2.5
0.0
Luminespib senstivity
Low
High
ATOH8

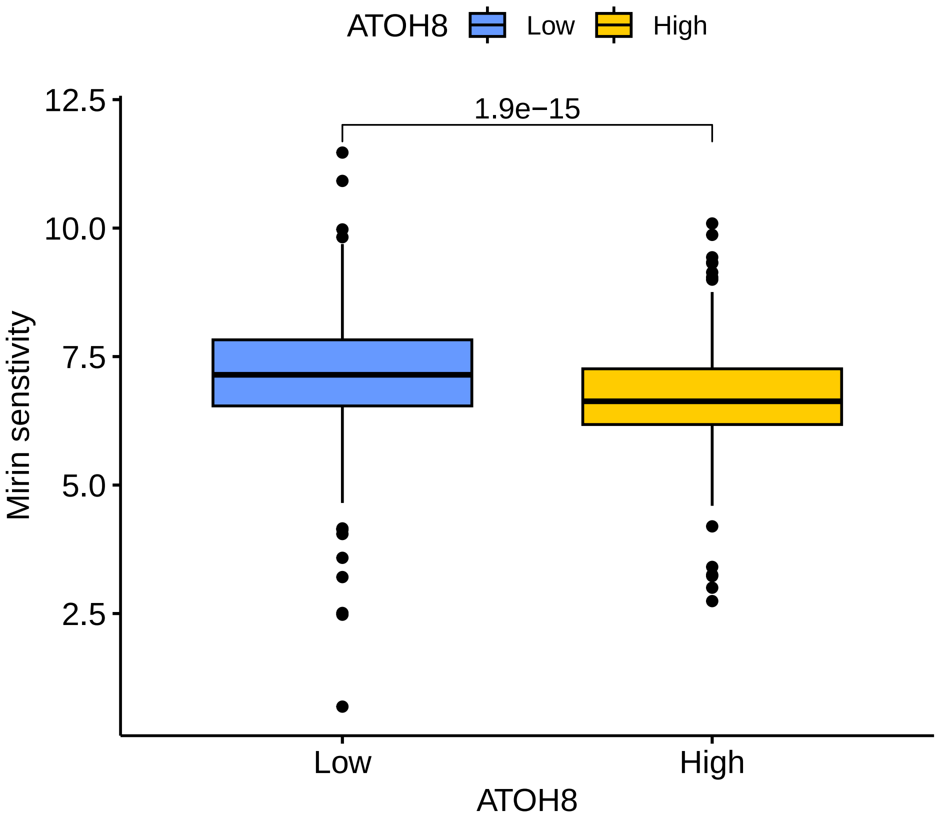
ATOH8
Low
High
1.9e−15
12.5
10.0
7.5
5.0
2.5
Mirin senstivity
Low
High
ATOH8

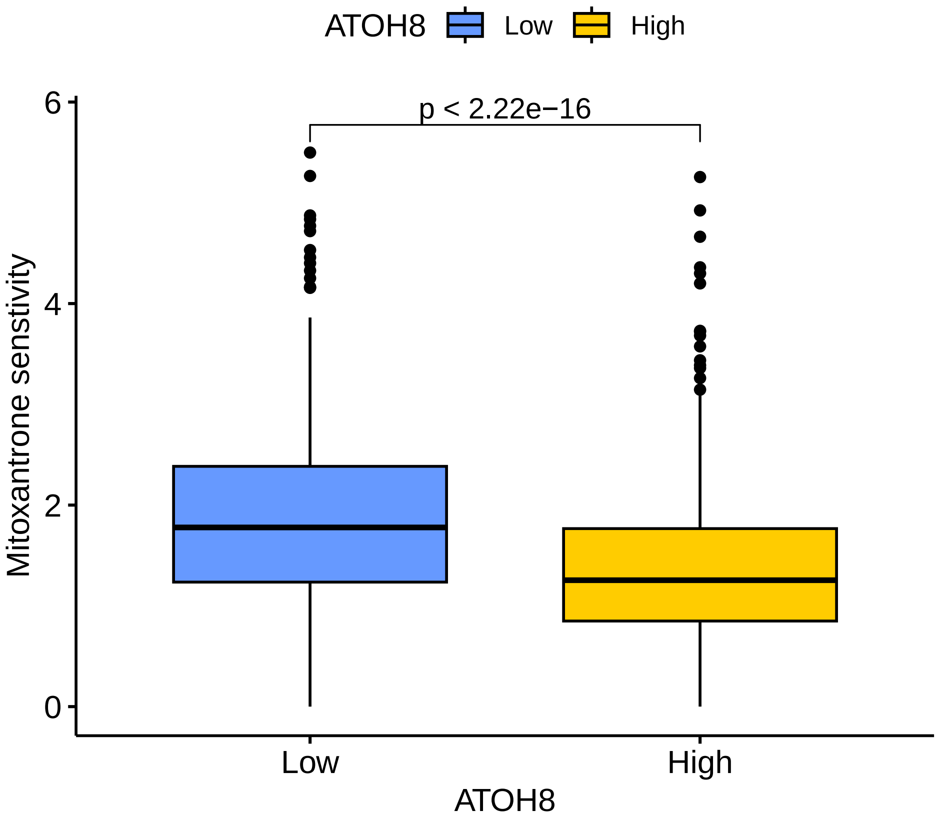

ATOH8
Low
High
p < 2.22e−16
6
4
2
0
Mitoxantrone senstivity
Low
High
ATOH8

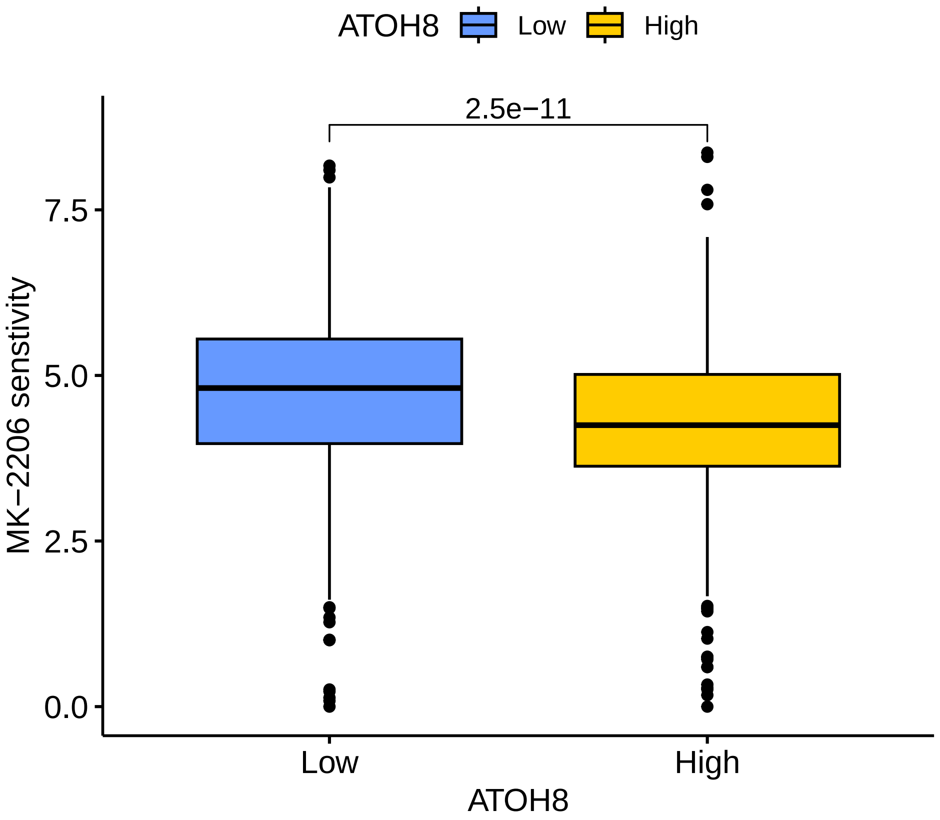

ATOH8
Low
High
2.5e−11
7.5
5.0
2.5
0.0
MK−2206 senstivity
Low
High
ATOH8

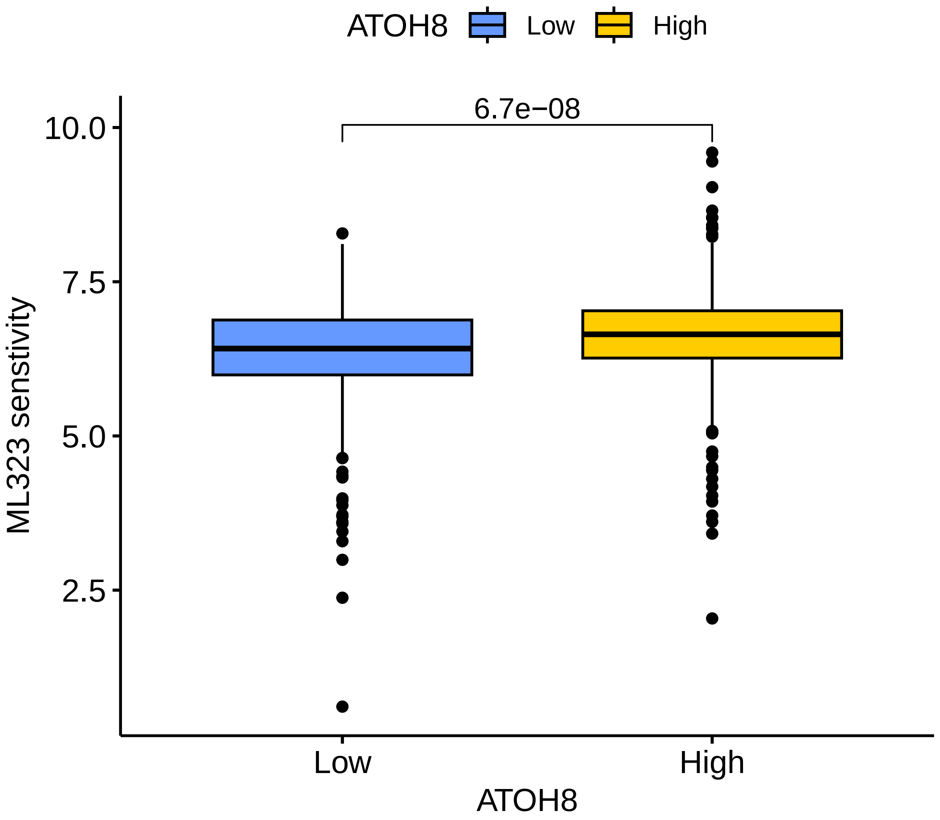
ATOH8
Low
High
6.7e−08
10.0
7.5
5.0
2.5
ML323 senstivity
Low
High
ATOH8

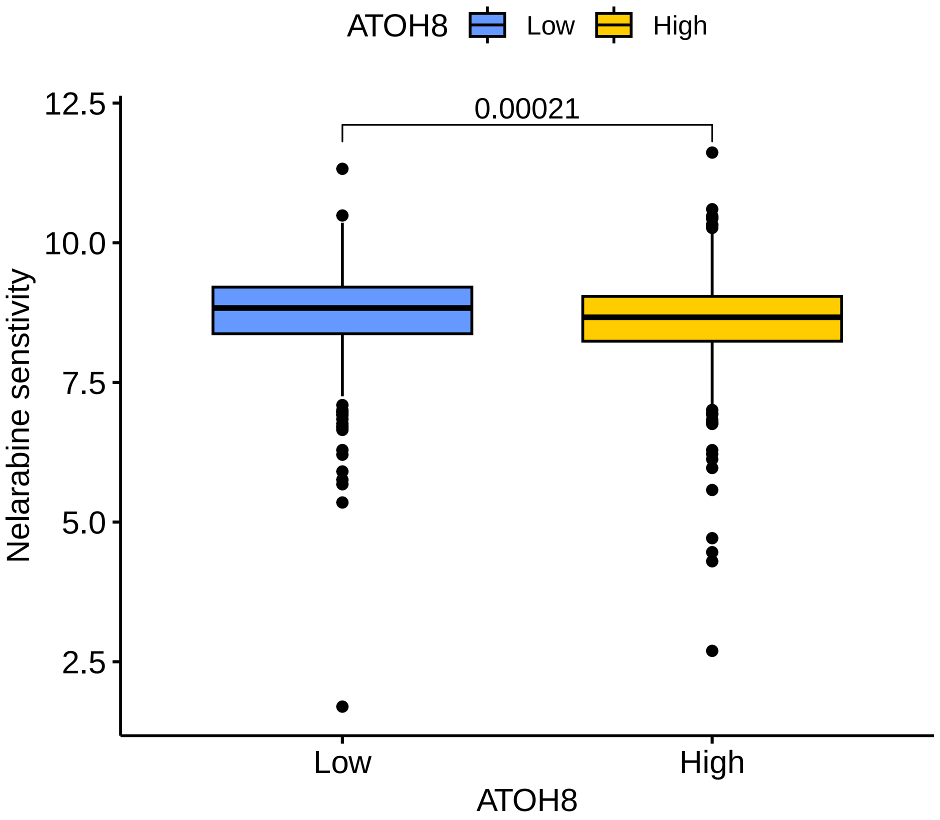
ATOH8
Low
High
12.5
10.0
7.5
5.0
2.5
0.00021
Nelarabine senstivity
Low
High
ATOH8

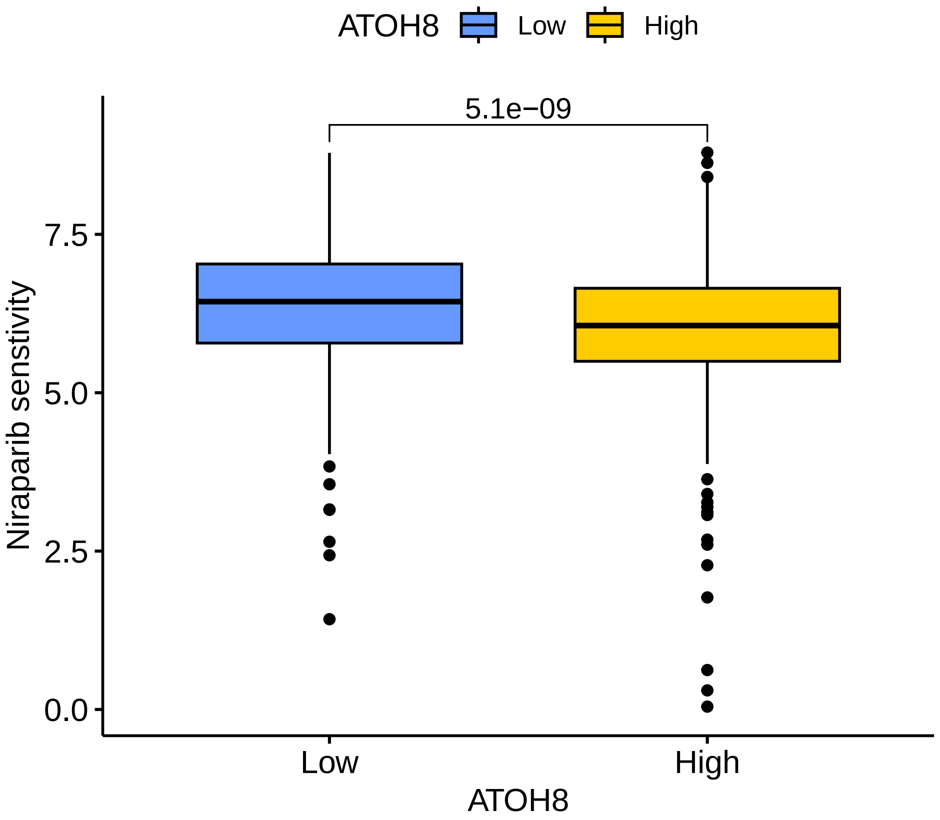
ATOH8
Low
High
5.1e−09
Niraparib senstivity
7.5
5.0
2.5
0.0
Low
High
ATOH8

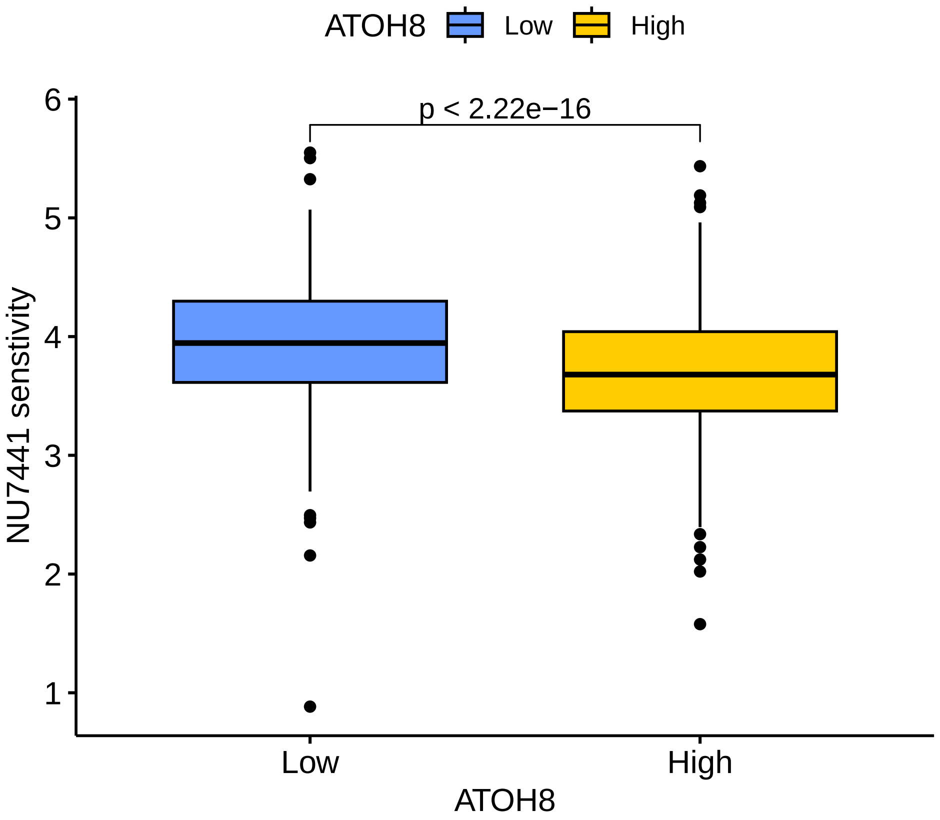

ATOH8
Low
High
p < 2.22e−16
NU7441 senstivity
6
5
4
3
2
1
Low
High
ATOH8

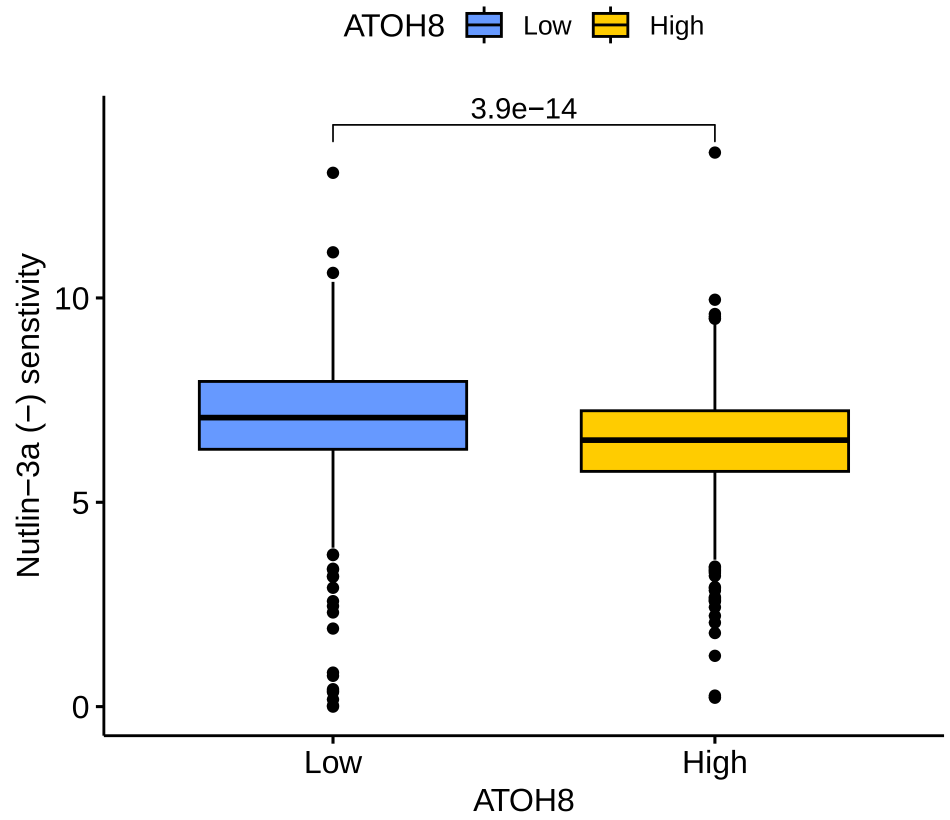
ATOH8
Low
High
3.9e−14
Nutlin−3a (−) senstivity
10
5
0
Low
High
ATOH8

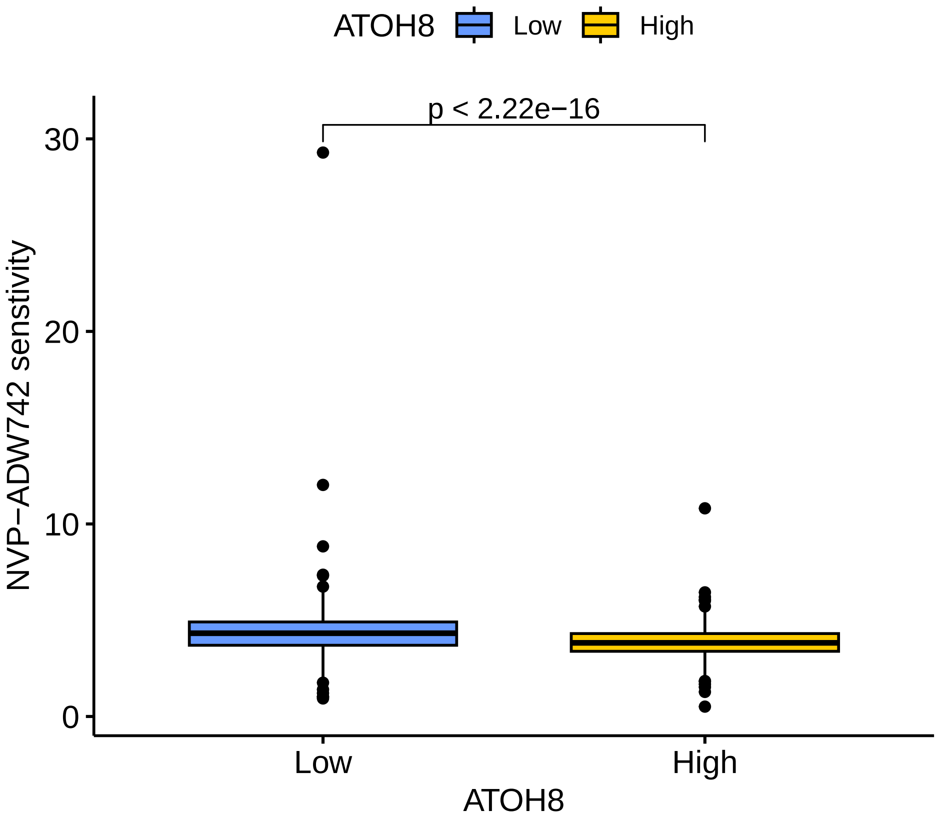

ATOH8
Low
High
p < 2.22e−16
30
20
10
0
NVP−ADW742 senstivity
Low
High
ATOH8

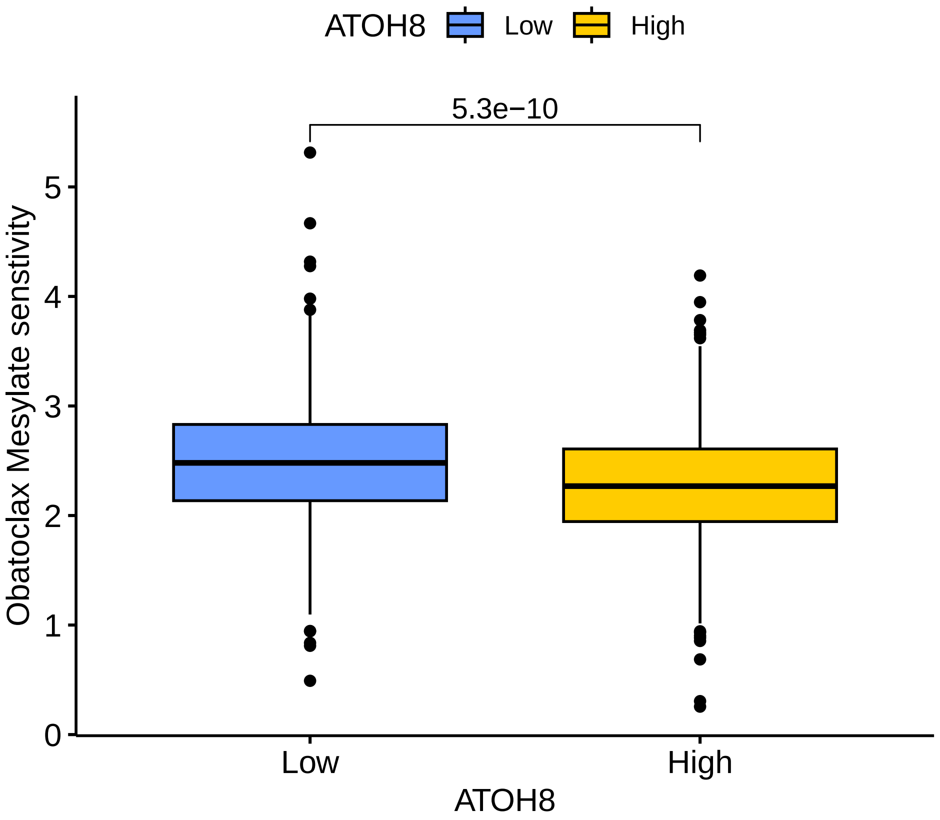

ATOH8
Low
High
5.3e−10
Obatoclax Mesylate senstivity
0
1
2
3
4
5
Low
High
ATOH8

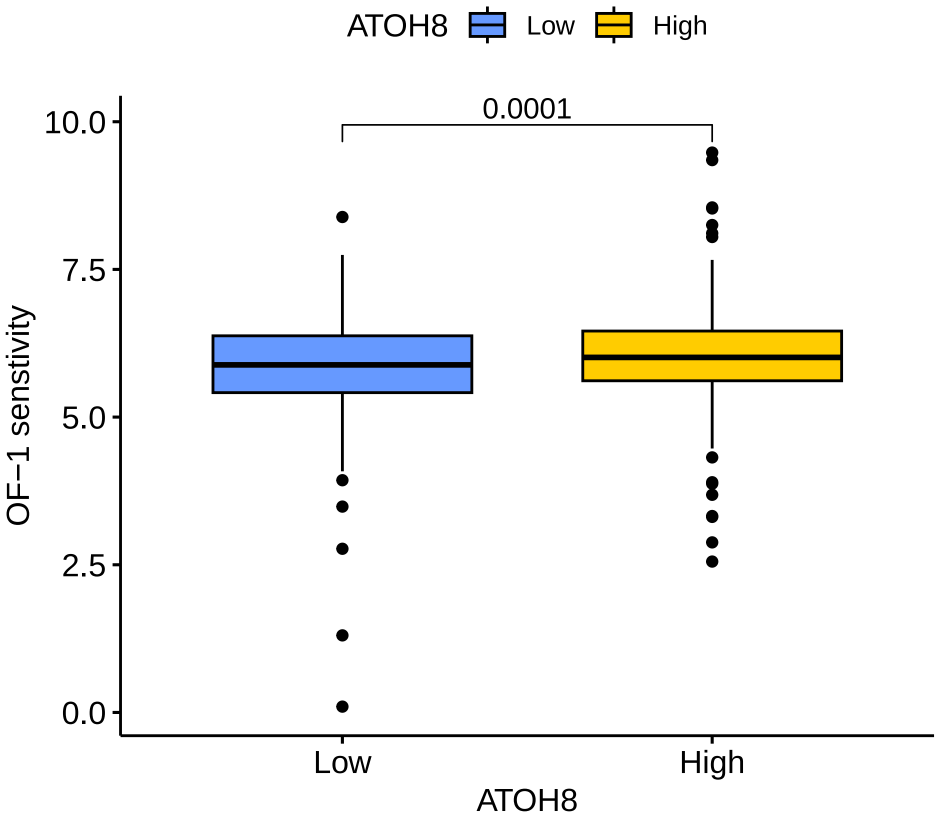
ATOH8
Low
High
0.0001
10.0
7.5
5.0
2.5
0.0
OF−1 senstivity
Low
High
ATOH8

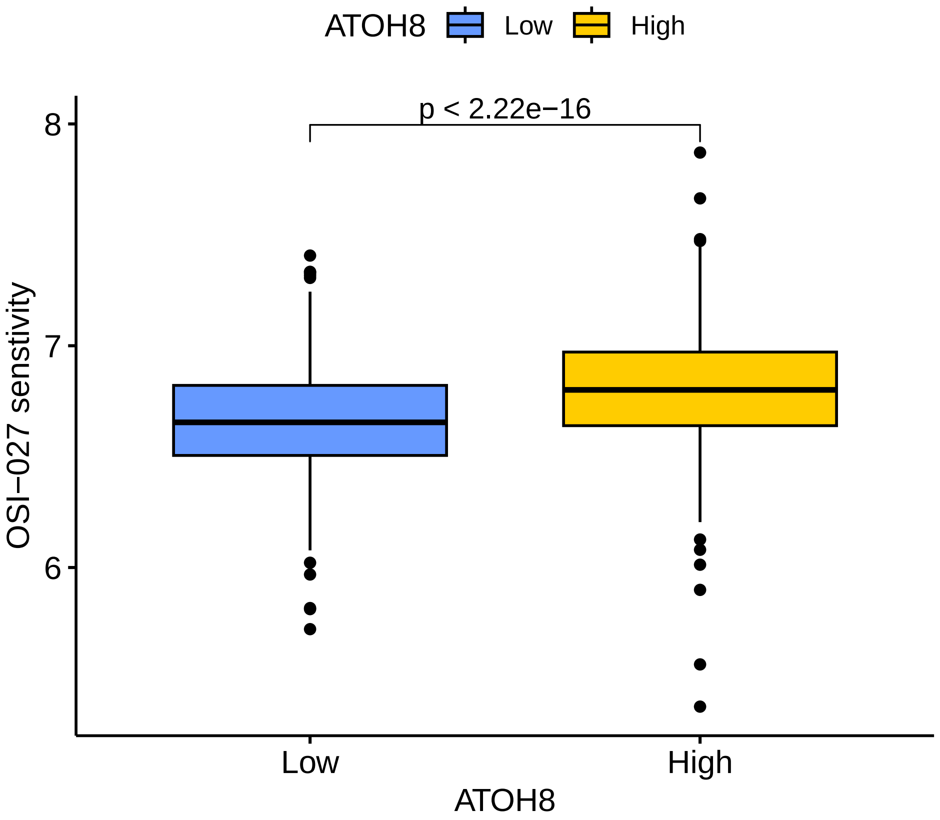
ATOH8
Low
High
p < 2.22e−16
8
7
6
OSI−027 senstivity
Low
High
ATOH8

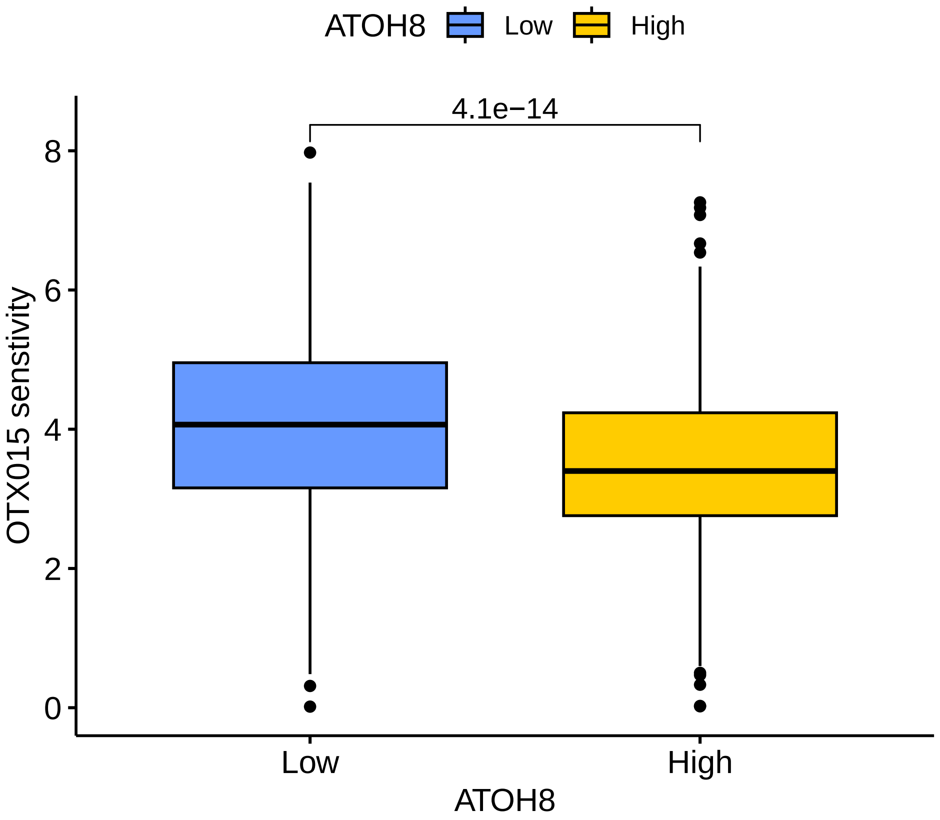
ATOH8
Low
High
4.1e−14
OTX015 senstivity
0
2
4
6
8
Low
High
ATOH8

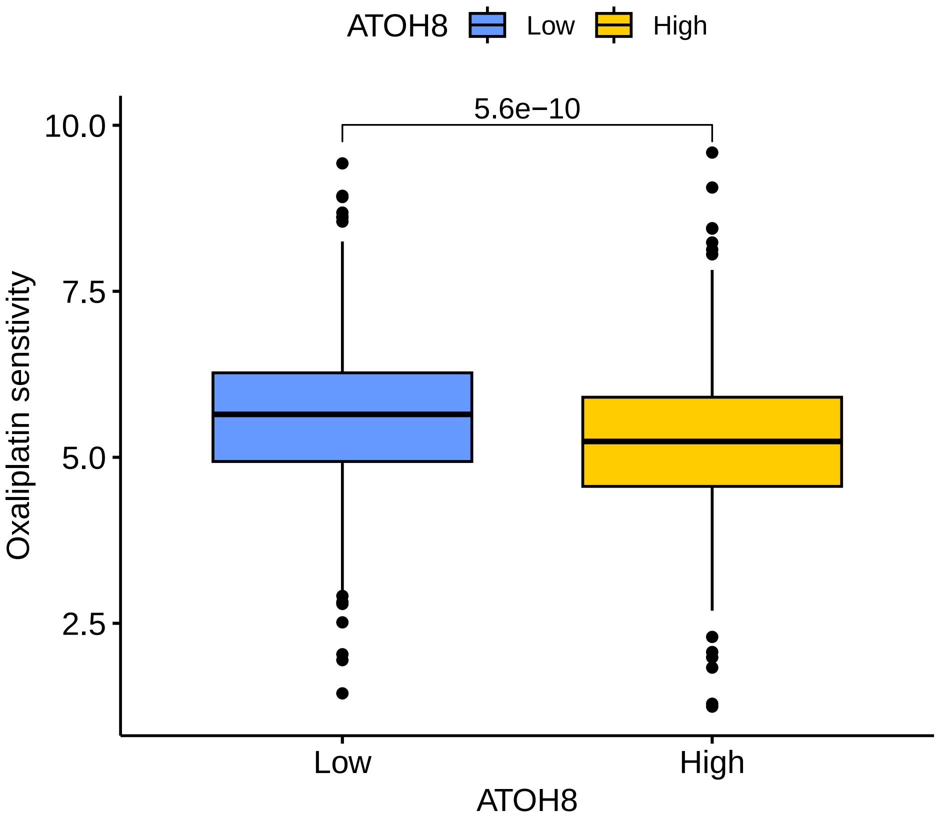

ATOH8
Low
High
5.6e−10
10.0
7.5
5.0
2.5
Oxaliplatin senstivity
Low
High
ATOH8

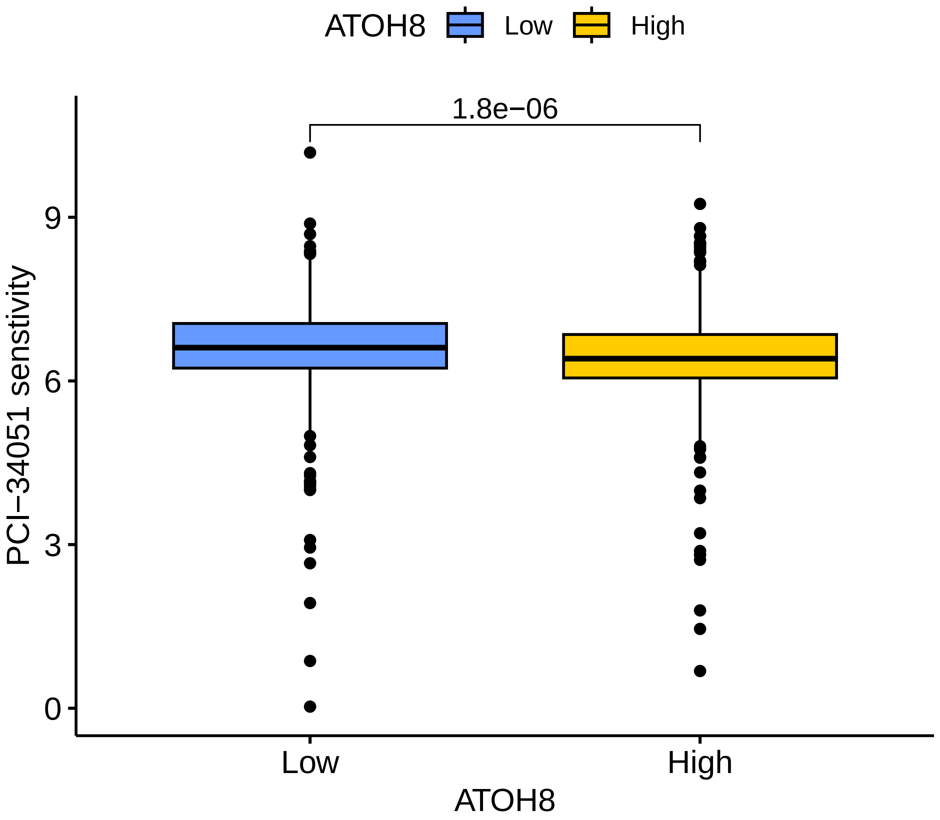

ATOH8
Low
High
1.8e−06
PCI−34051 senstivity
9
6
3
0
Low
High
ATOH8

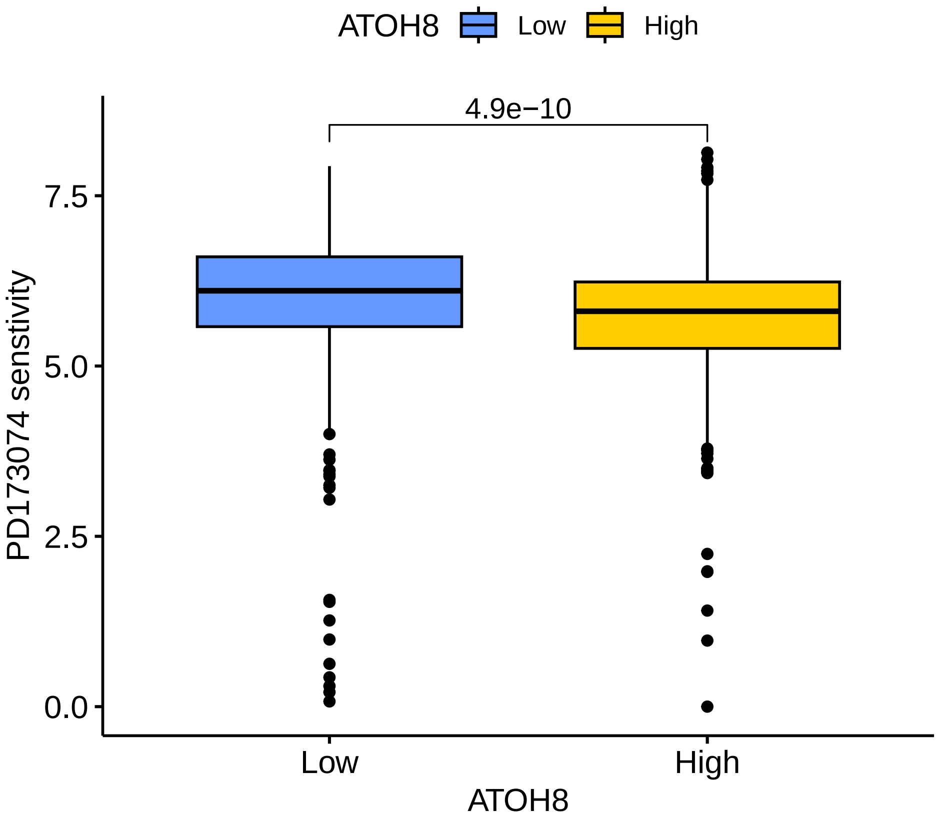

ATOH8
Low
High
4.9e−10
7.5
5.0
2.5
0.0
PD173074 senstivity
Low
High
ATOH8

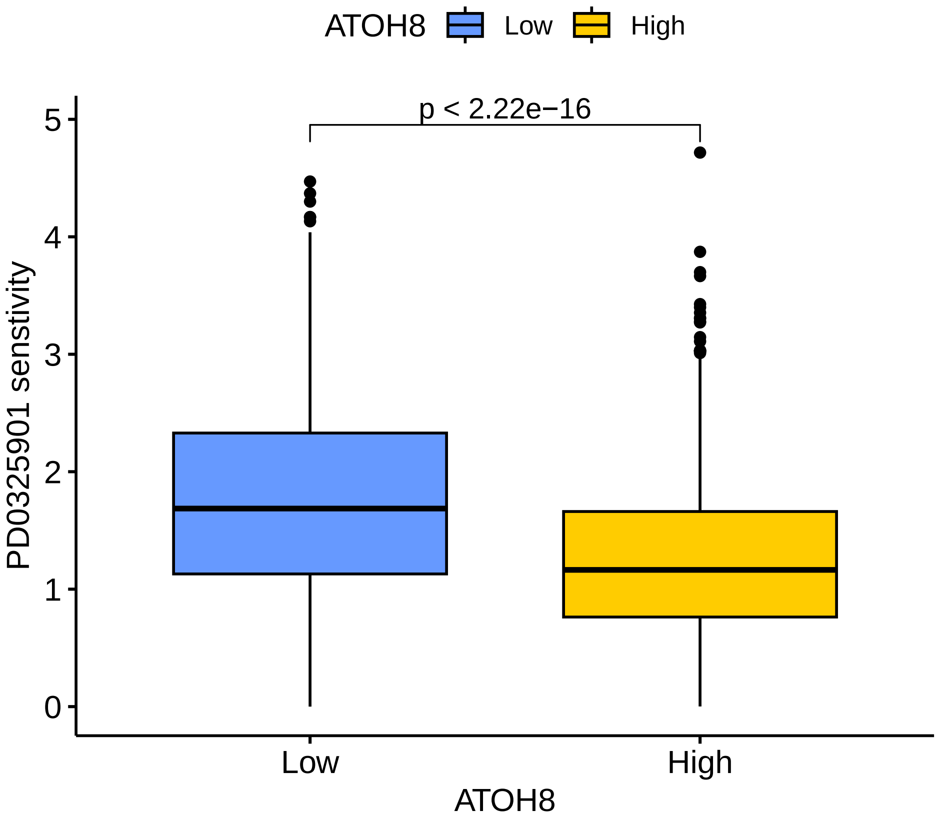
ATOH8
Low
High
p < 2.22e−16
PD0325901 senstivity
5
4
3
2
1
0
Low
High
ATOH8

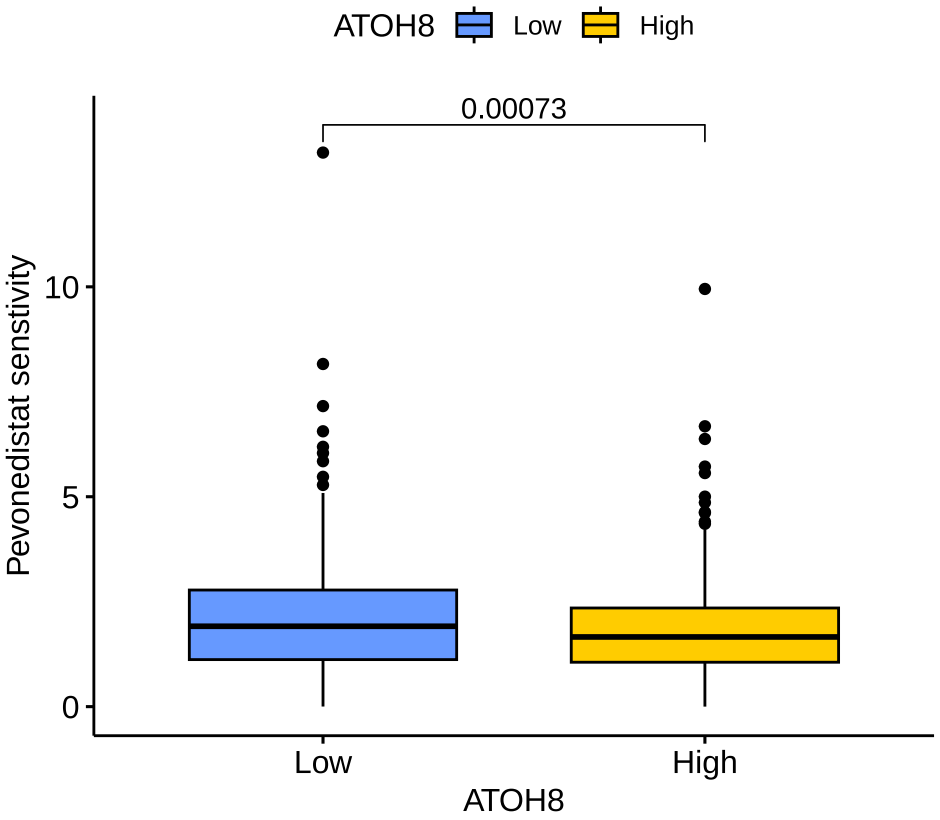
ATOH8
Low
High
0.00073
Pevonedistat senstivity
10
5
0
Low
High
ATOH8

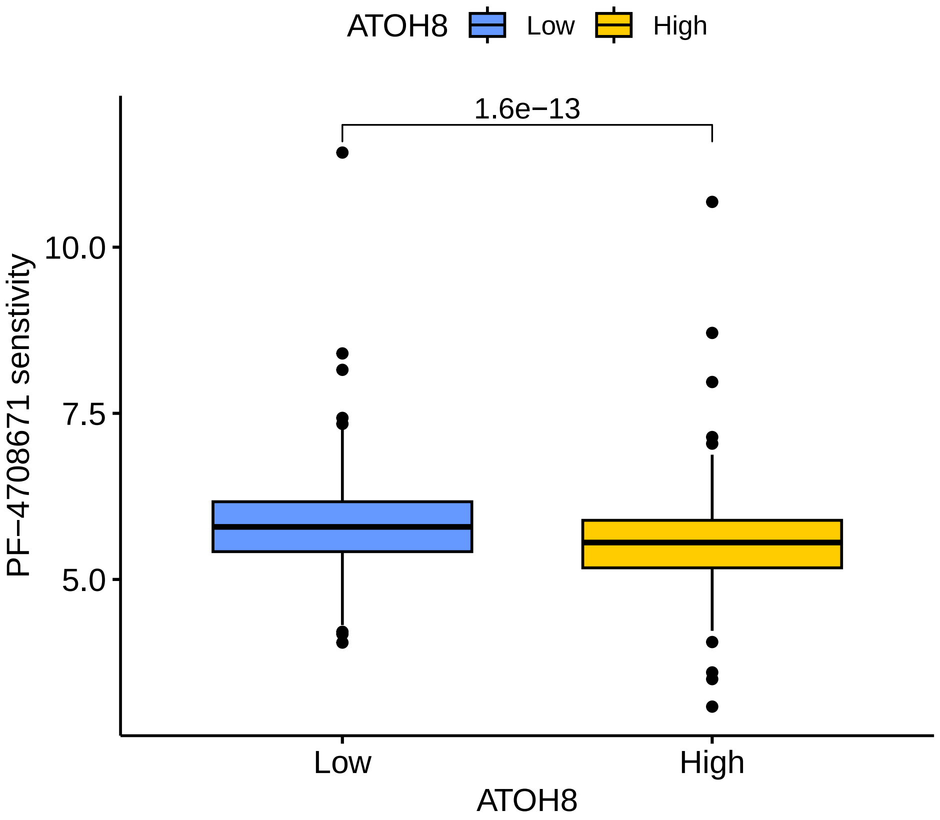

ATOH8
Low
High
1.6e−13
10.0
7.5
5.0
PF−4708671 senstivity
Low
High
ATOH8

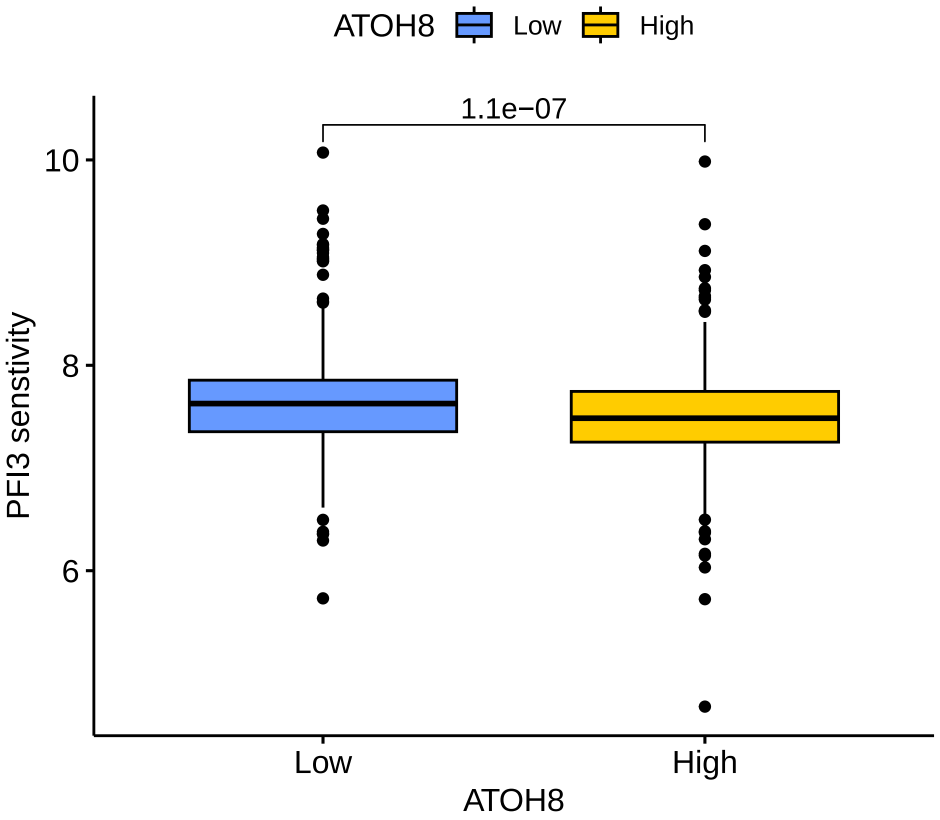

ATOH8
Low
High
1.1e−07
10
8
6
PFI3 senstivity
Low
High
ATOH8

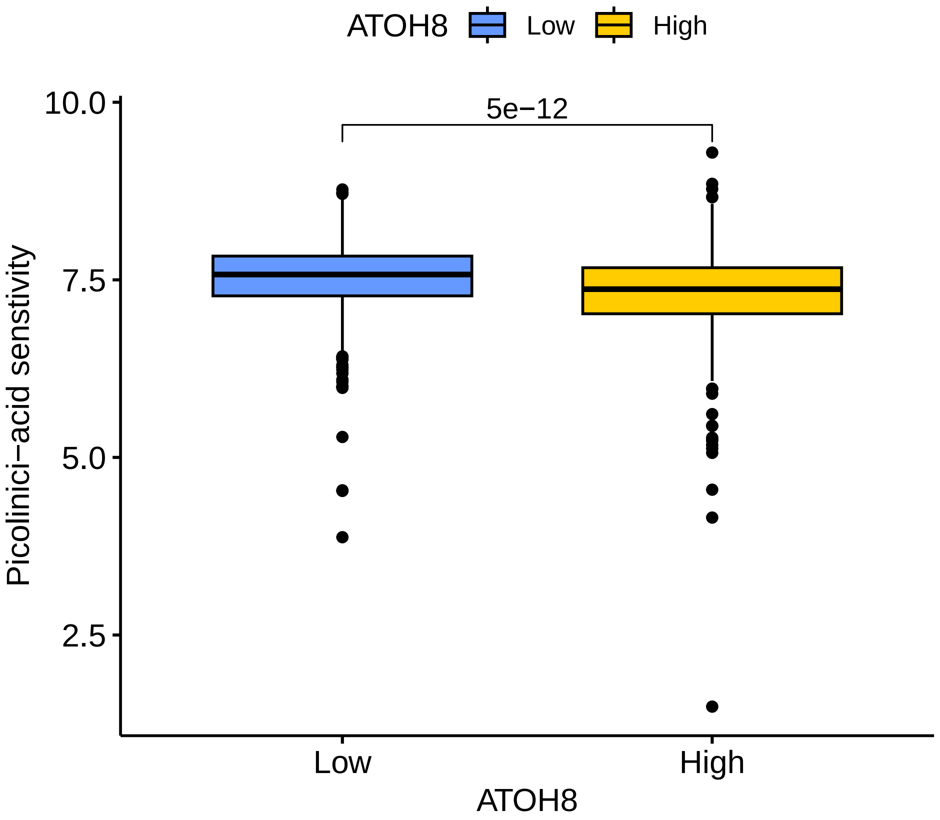
ATOH8
Low
High
5e−12
10.0
7.5
5.0
2.5
Picolinici−acid senstivity
Low
High
ATOH8

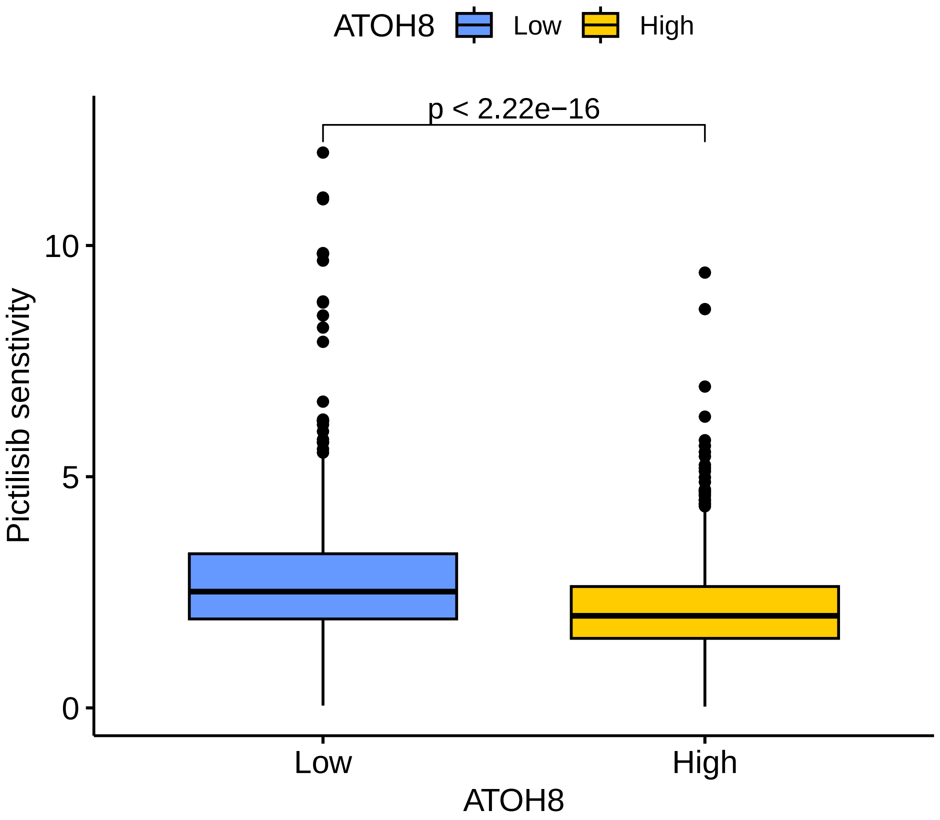
ATOH8
Low
High
p < 2.22e−16
Pictilisib senstivity
10
5
0
Low
High
ATOH8

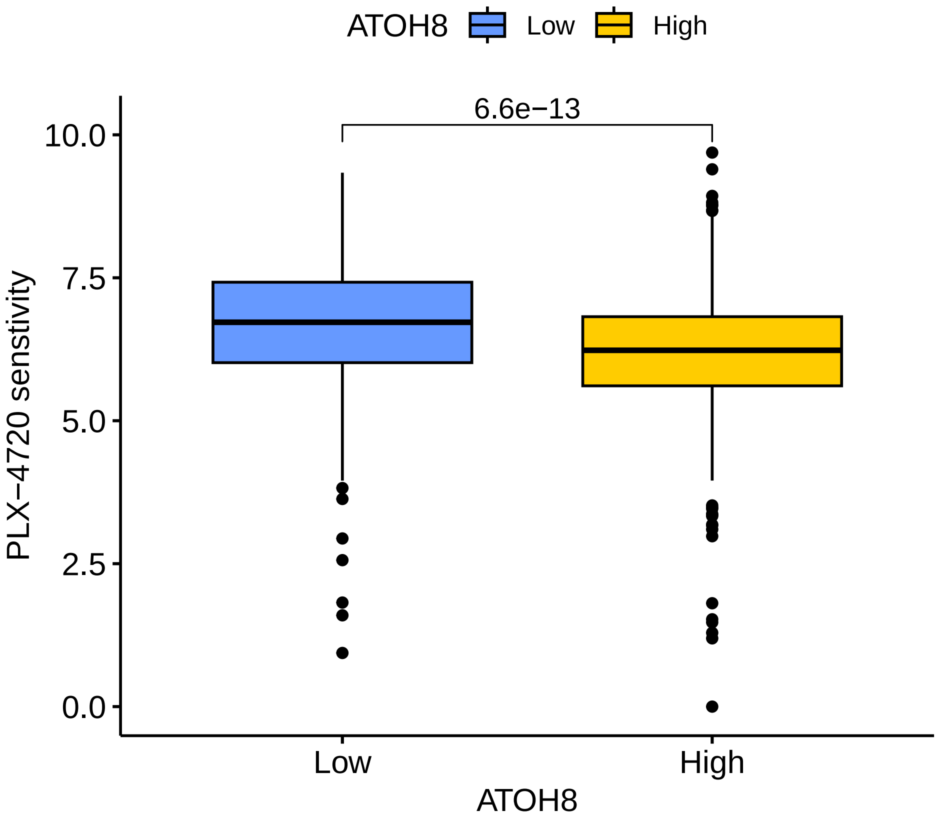
ATOH8
Low
High
6.6e−13
10.0
7.5
5.0
2.5
0.0
PLX−4720 senstivity
Low
High
ATOH8

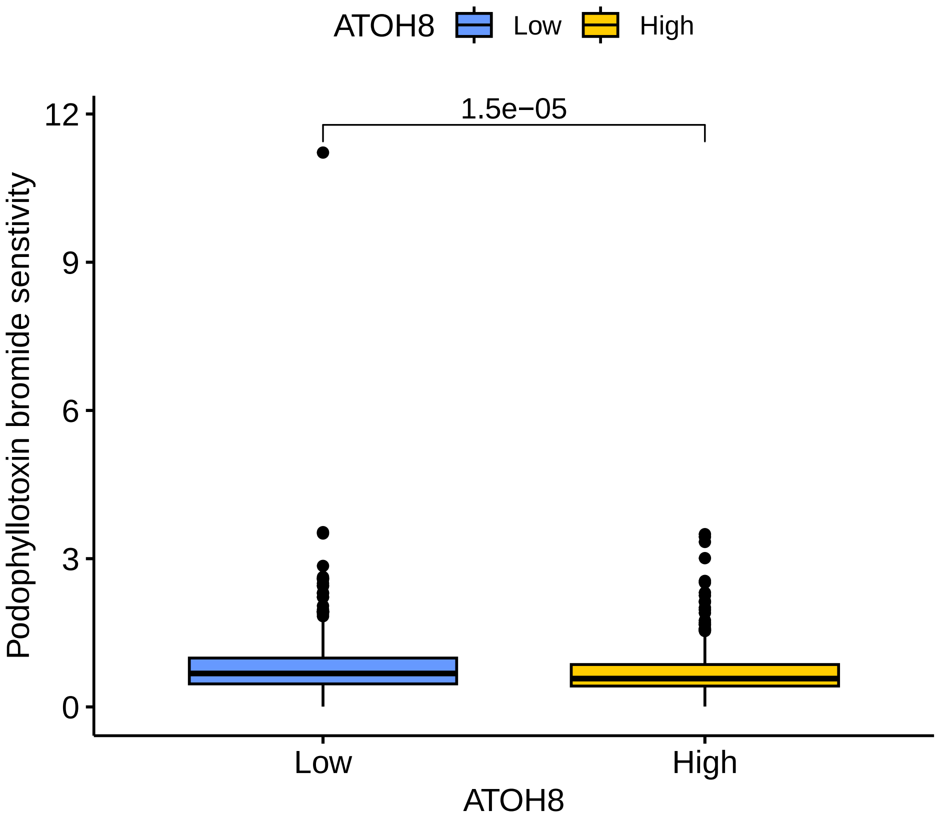

ATOH8
Low
High
1.5e−05
12
9
6
3
0
Podophyllotoxin bromide senstivity
Low
High
ATOH8

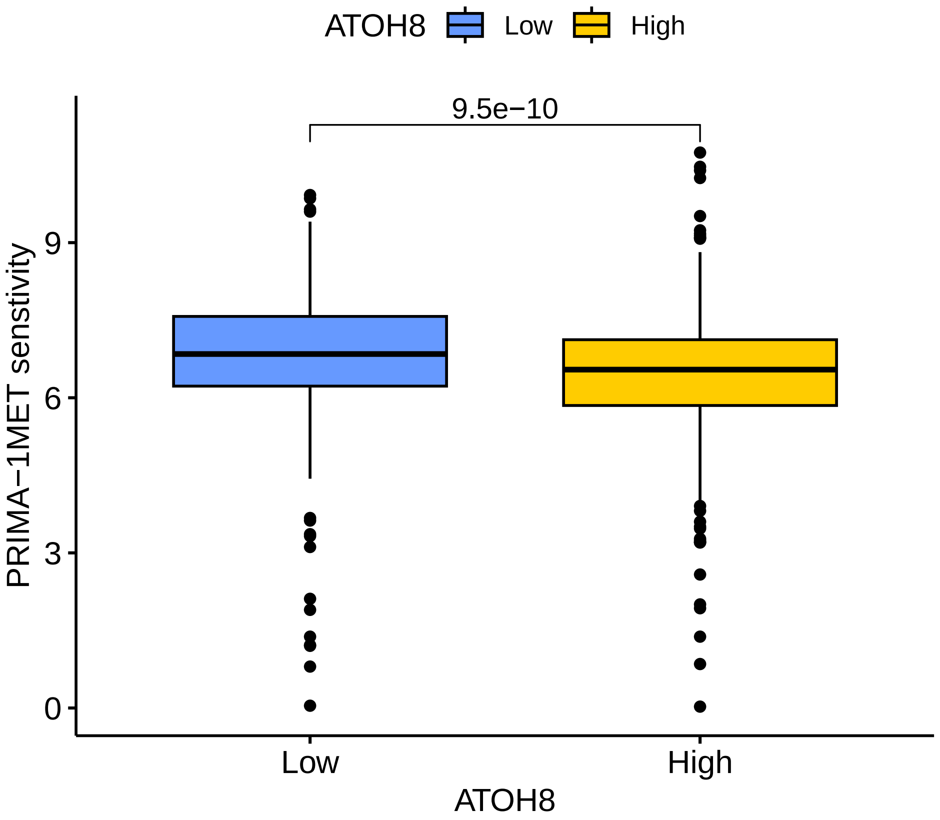
ATOH8
Low
High
9.5e−10
PRIMA−1MET senstivity
9
6
3
0
Low
High
ATOH8

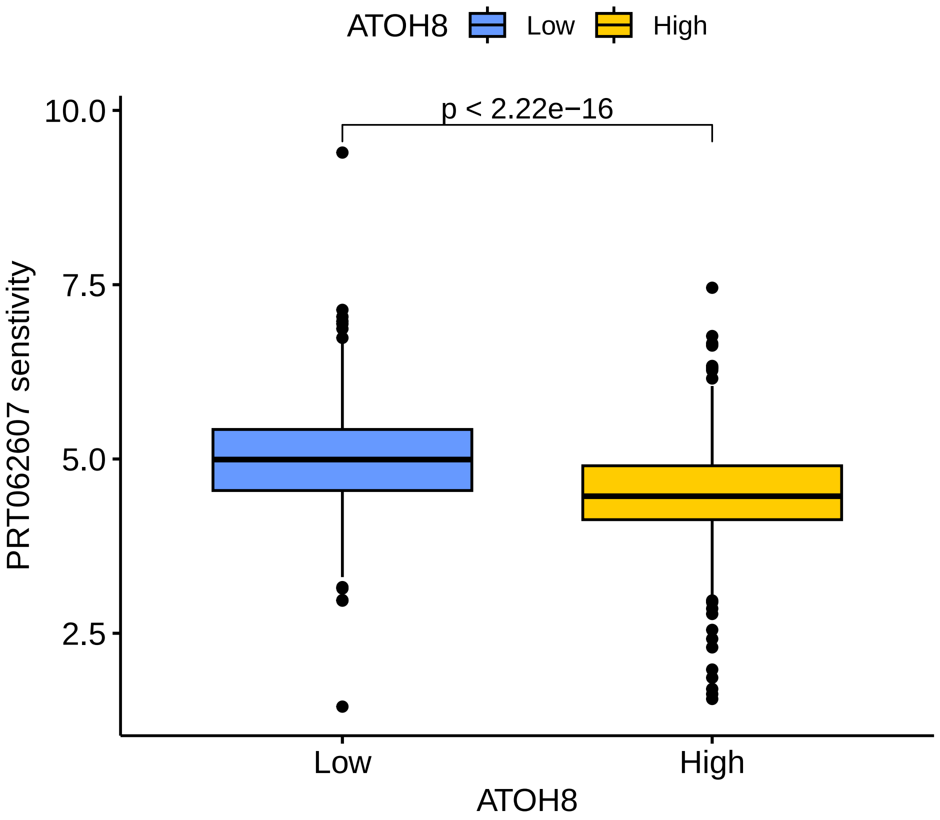
ATOH8
Low
High
p < 2.22e−16
10.0
7.5
5.0
2.5
PRT062607 senstivity
Low
High
ATOH8

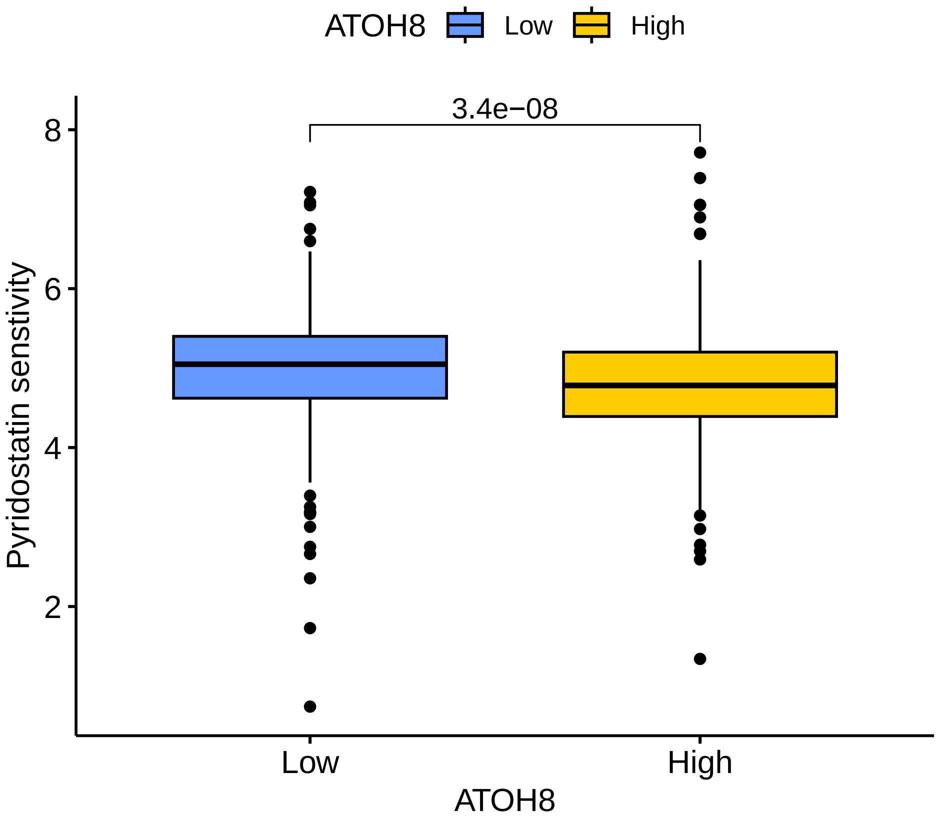

ATOH8
Low
High
3.4e−08
8
6
4
2
Pyridostatin senstivity
Low
High
ATOH8

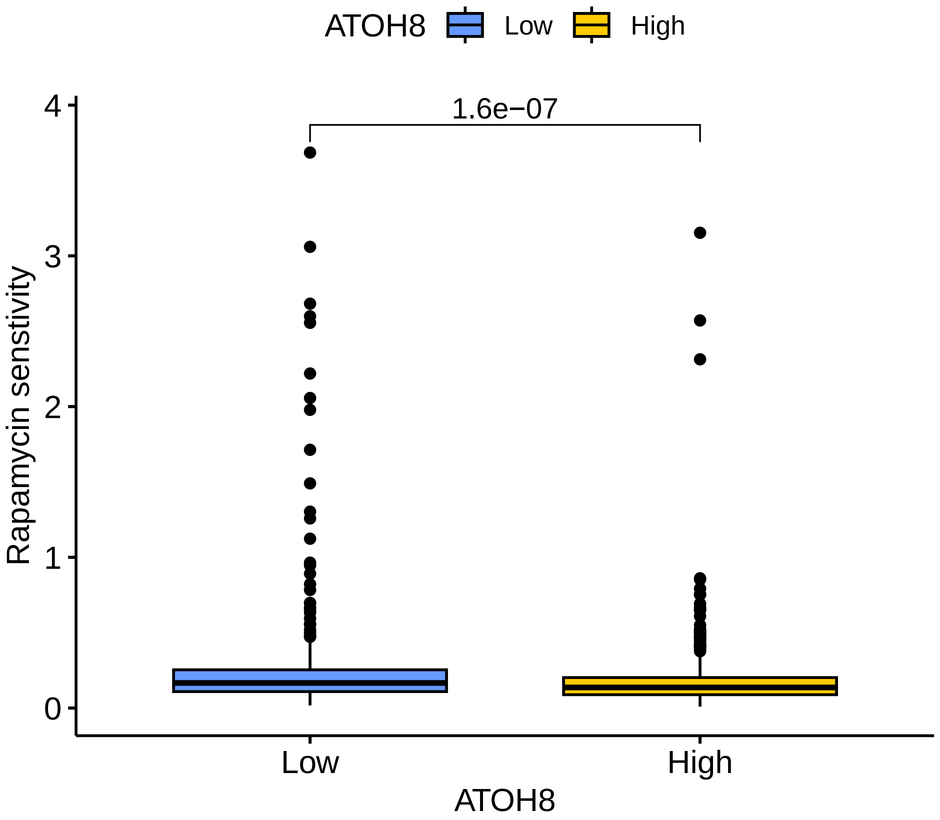

ATOH8
Low
High
1.6e−07
Rapamycin senstivity
4
3
2
1
0
Low
High
ATOH8

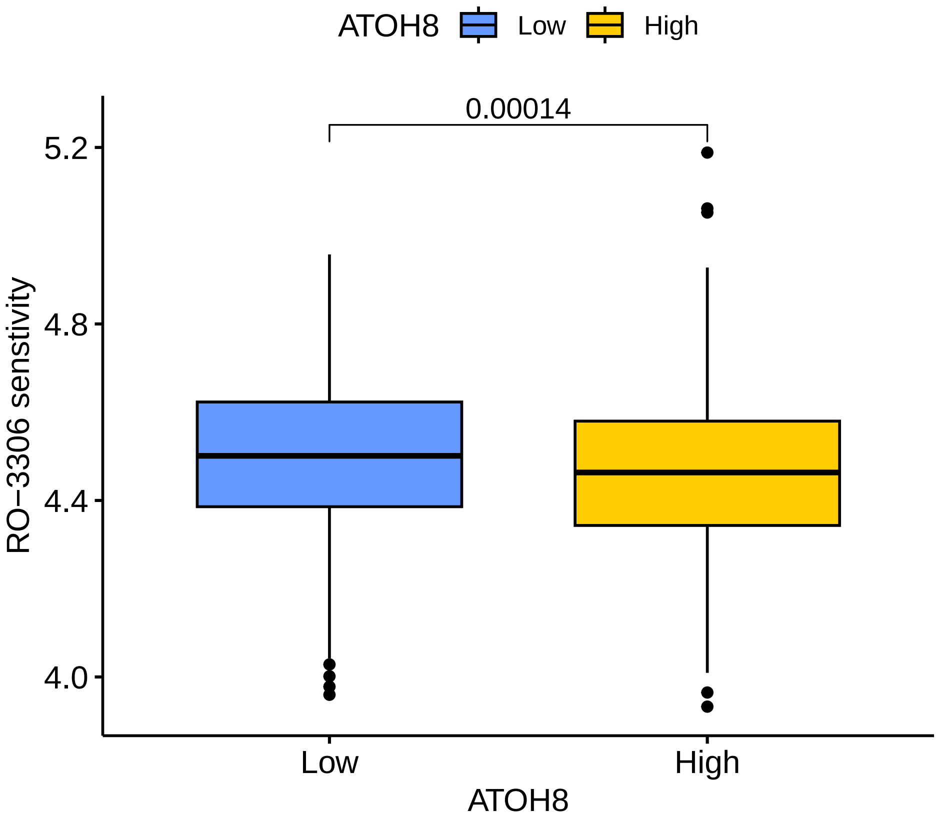
ATOH8
Low
High
0.00014
5.2
4.8
4.4
4.0
RO−3306 senstivity
Low
High
ATOH8

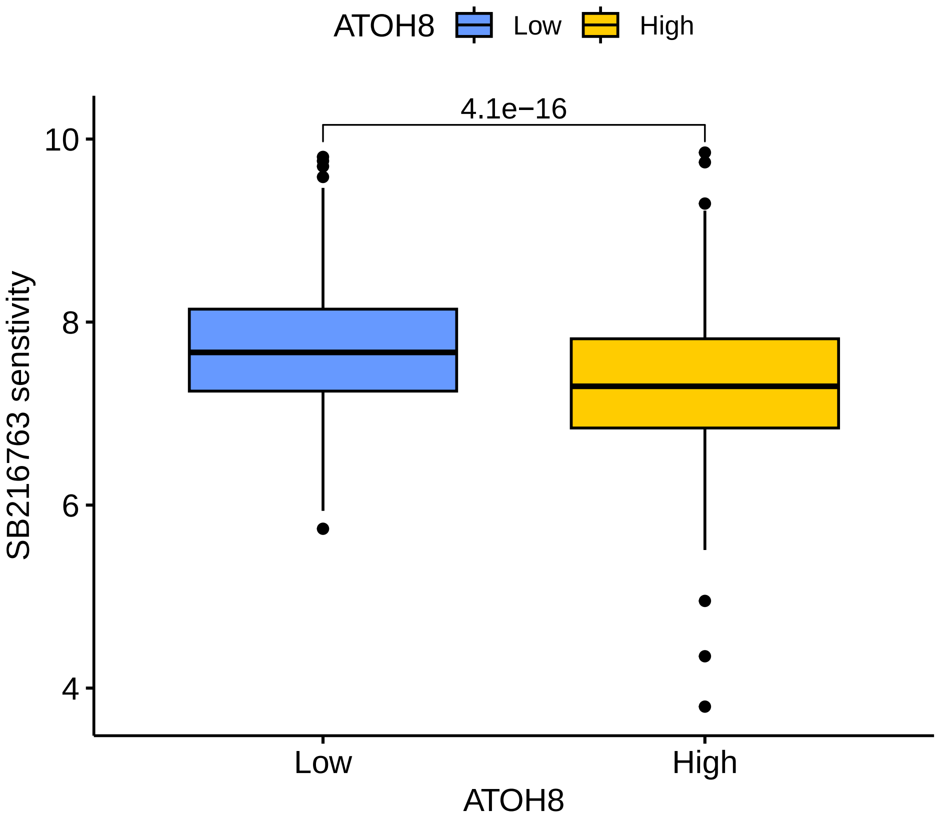

ATOH8
Low
High
4.1e−16
10
8
6
4
SB216763 senstivity
Low
High
ATOH8

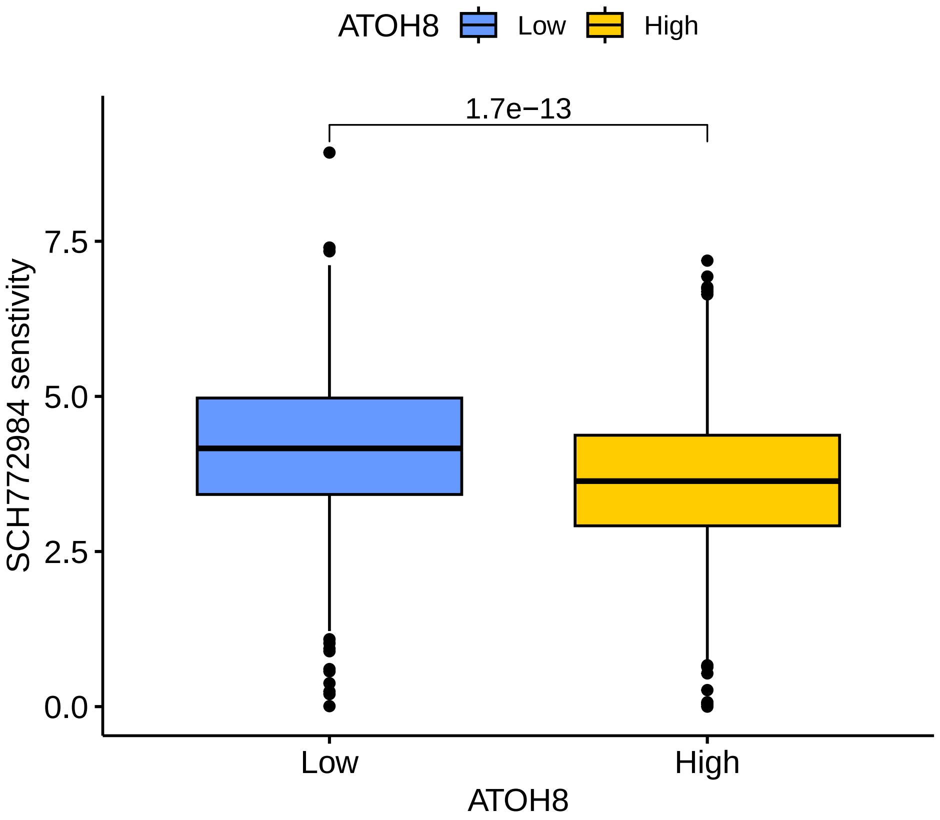
ATOH8
Low
High
1.7e−13
SCH772984 senstivity
7.5
5.0
2.5
0.0
Low
High
ATOH8

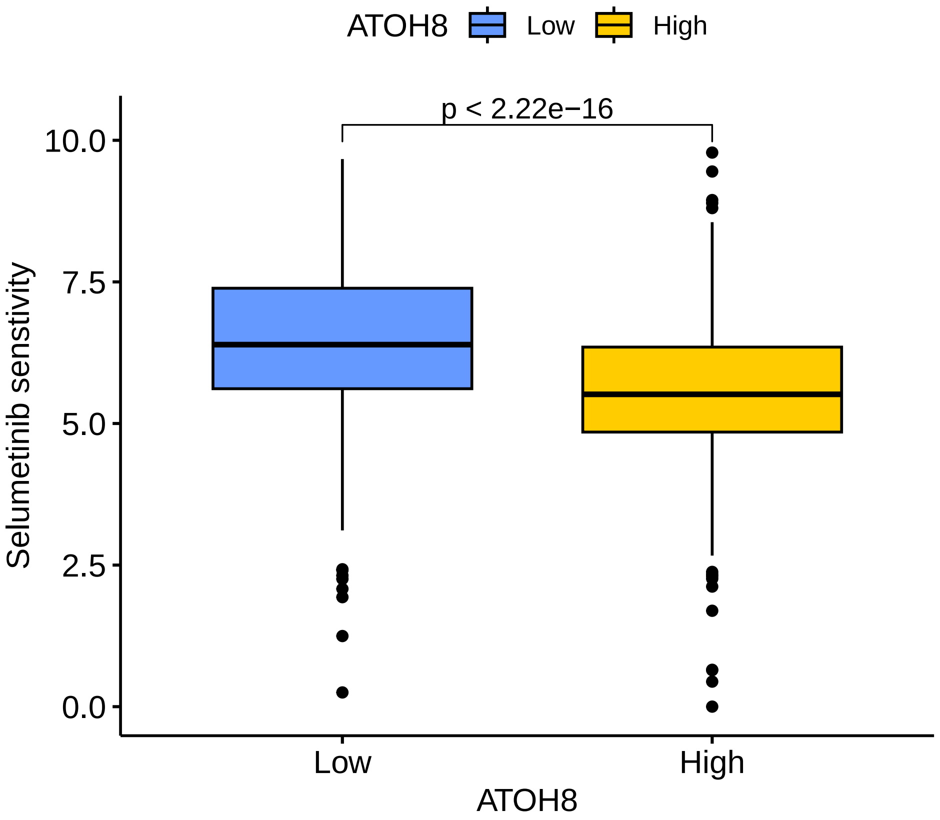
ATOH8
Low
High
p < 2.22e−16
10.0
7.5
5.0
2.5
0.0
Selumetinib senstivity
Low
High
ATOH8

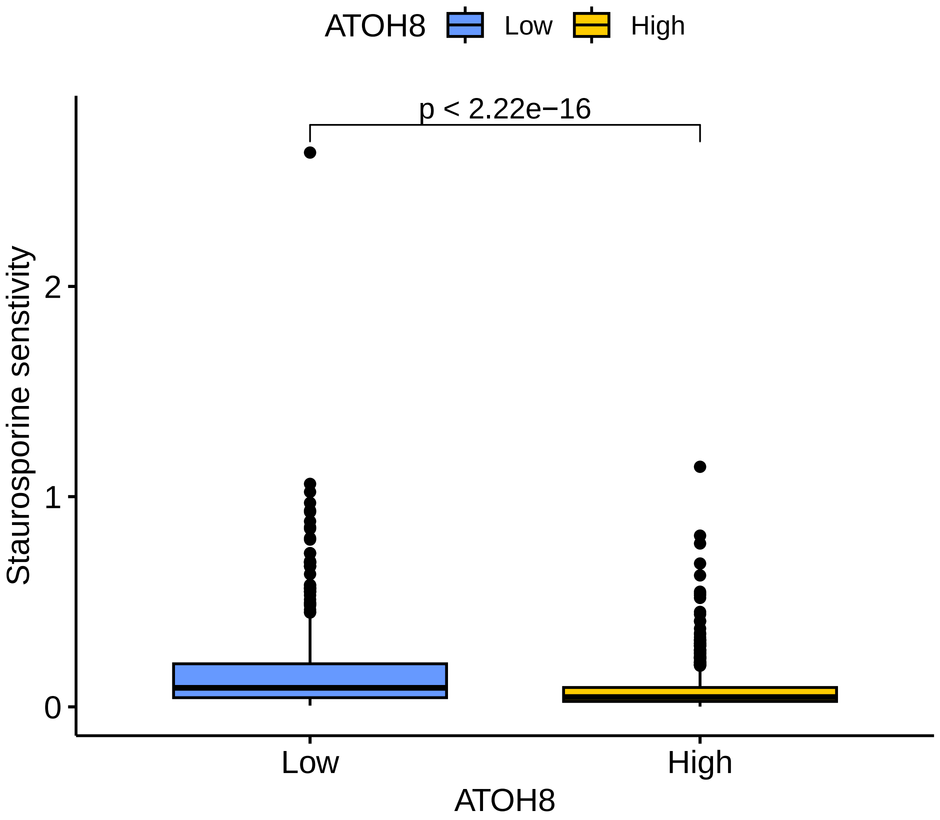

ATOH8
Low
High
p < 2.22e−16
Staurosporine senstivity
2
1
0
Low
High
ATOH8

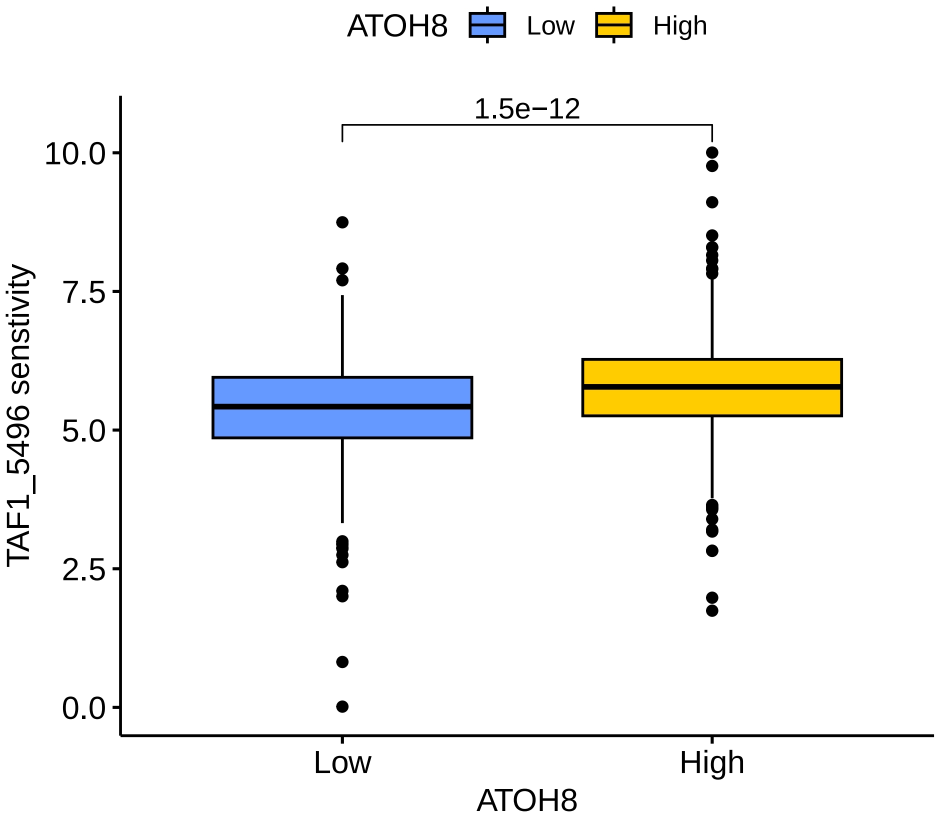

ATOH8
Low
High
1.5e−12
10.0
7.5
5.0
2.5
0.0
TAF1_5496 senstivity
Low
High
ATOH8

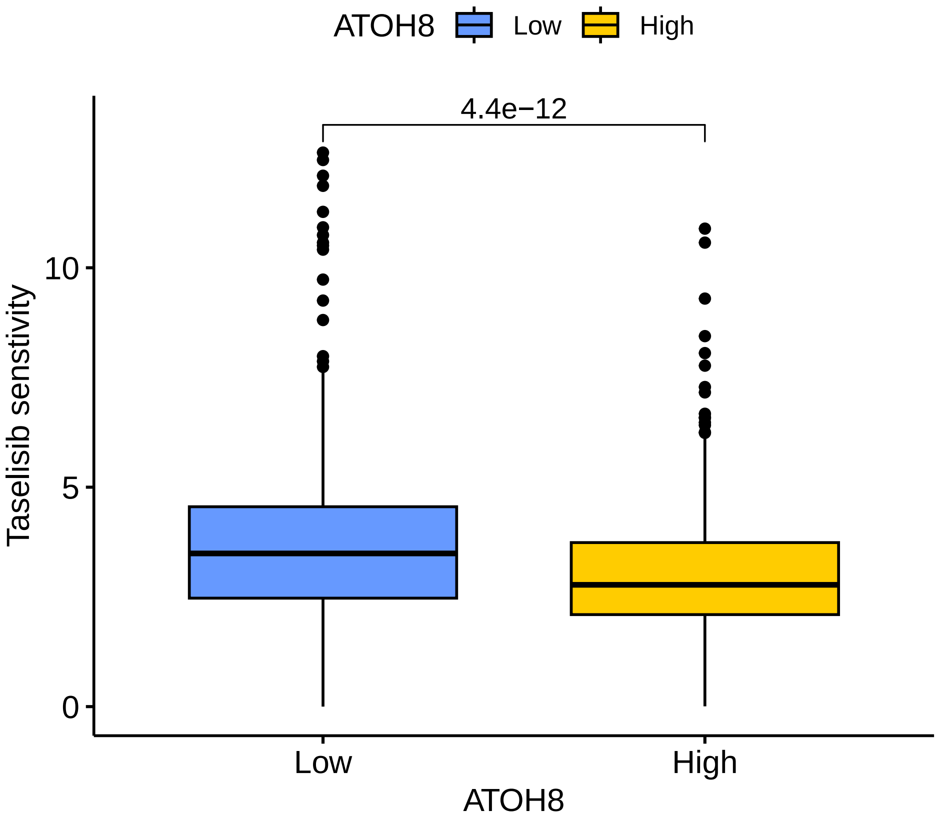
ATOH8
Low
High
4.4e−12
Taselisib senstivity
10
5
0
Low
High
ATOH8

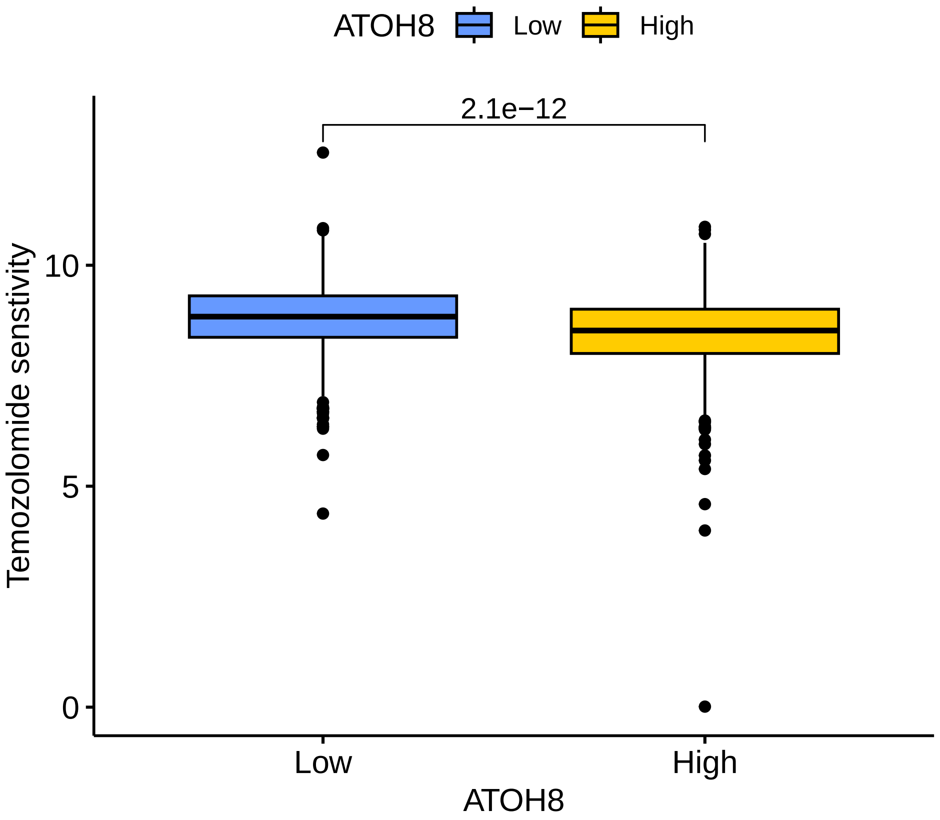

ATOH8
Low
High
2.1e−12
Temozolomide senstivity
10
5
0
Low
High
ATOH8

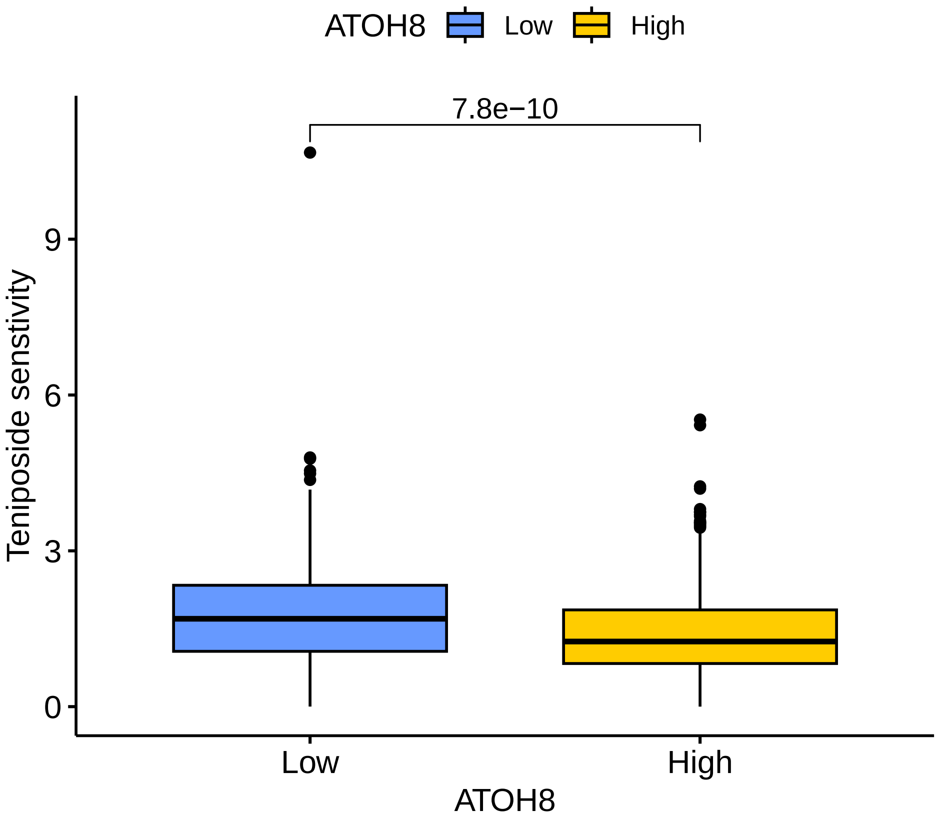
ATOH8
Low
High
7.8e−10
Teniposide senstivity
9
6
3
0
Low
High
ATOH8

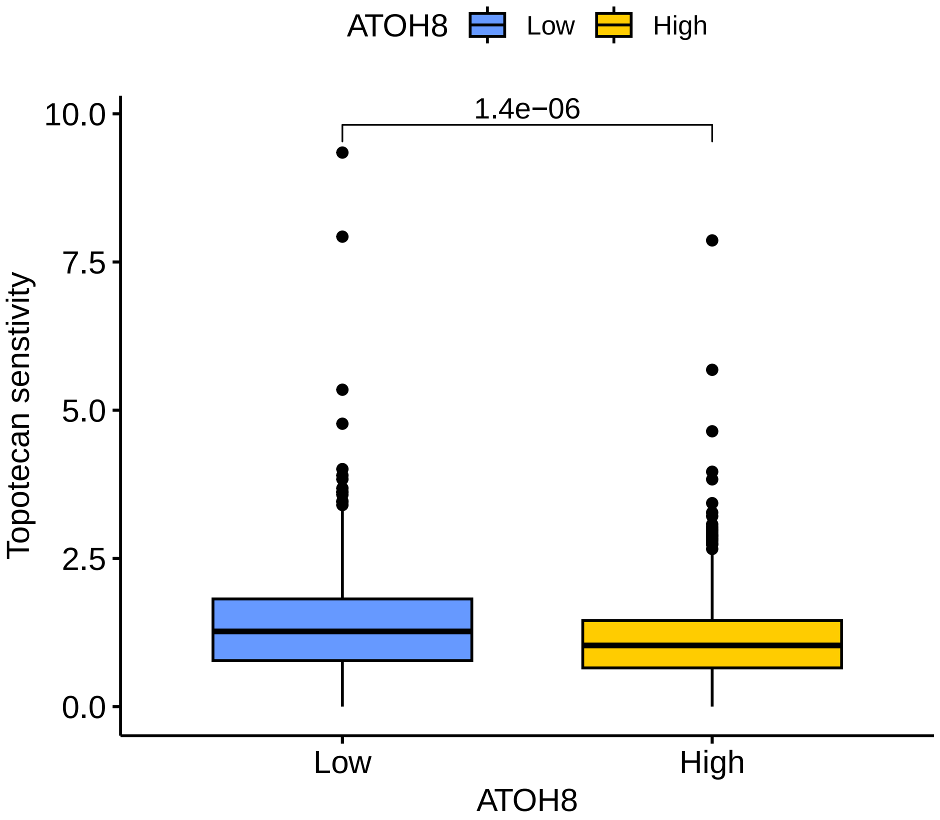

ATOH8
Low
High
1.4e−06
10.0
7.5
5.0
2.5
0.0
Topotecan senstivity
Low
High
ATOH8

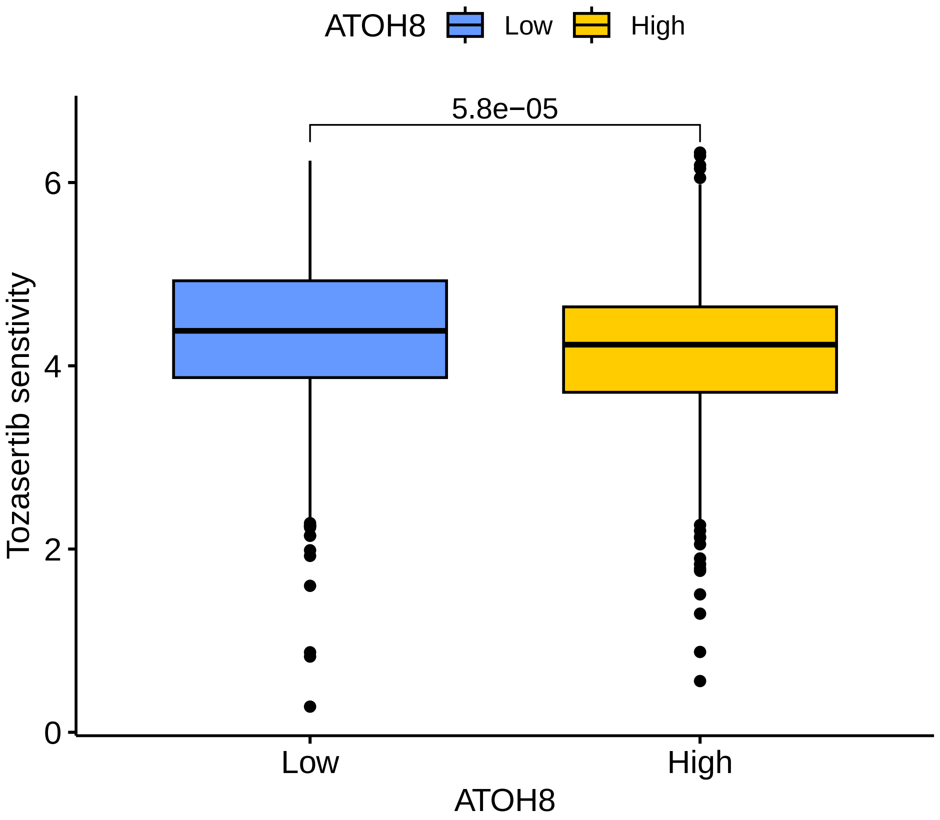
ATOH8
Low
High
5.8e−05
Tozasertib senstivity
6
4
2
0
Low
High
ATOH8

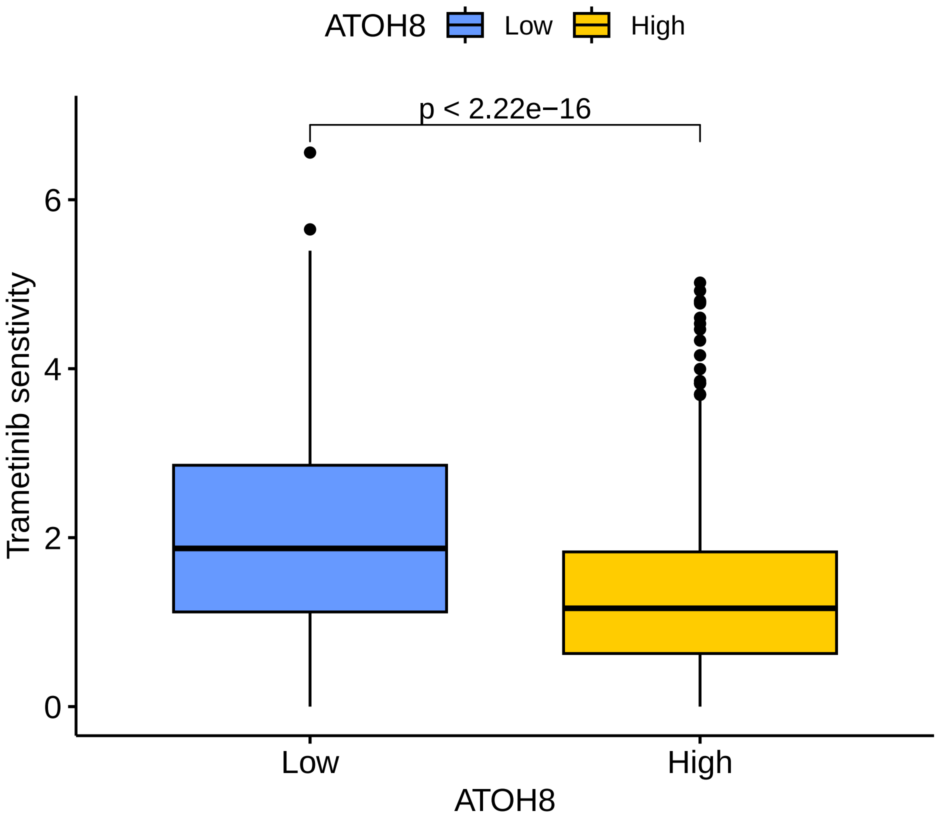
ATOH8
Low
High
p < 2.22e−16
Trametinib senstivity
6
4
2
0
Low
High
ATOH8

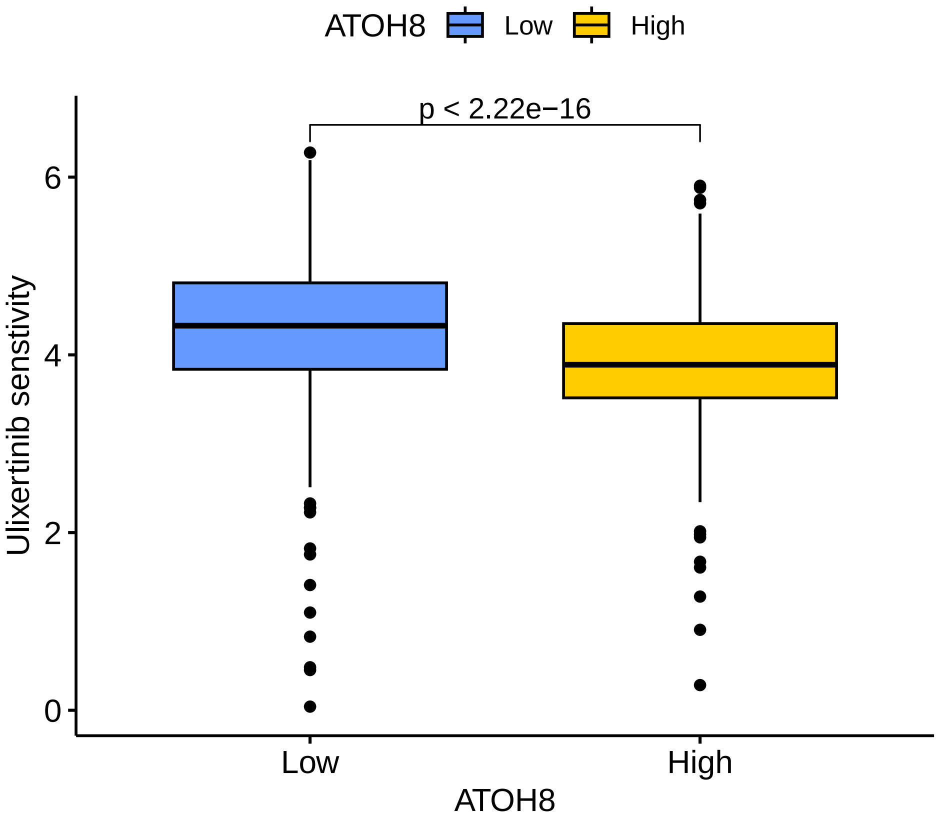

ATOH8
Low
High
p < 2.22e−16
Ulixertinib senstivity
6
4
2
0
Low
High
ATOH8

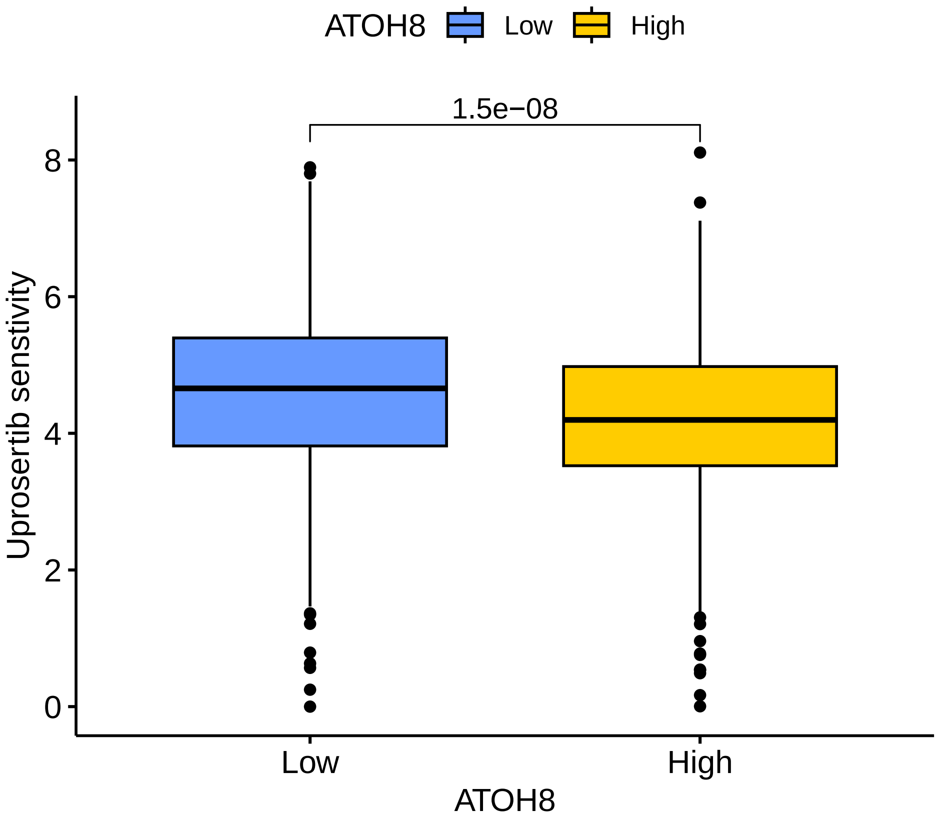

ATOH8
Low
High
1.5e−08
Uprosertib senstivity
8
6
4
2
0
Low
High
ATOH8

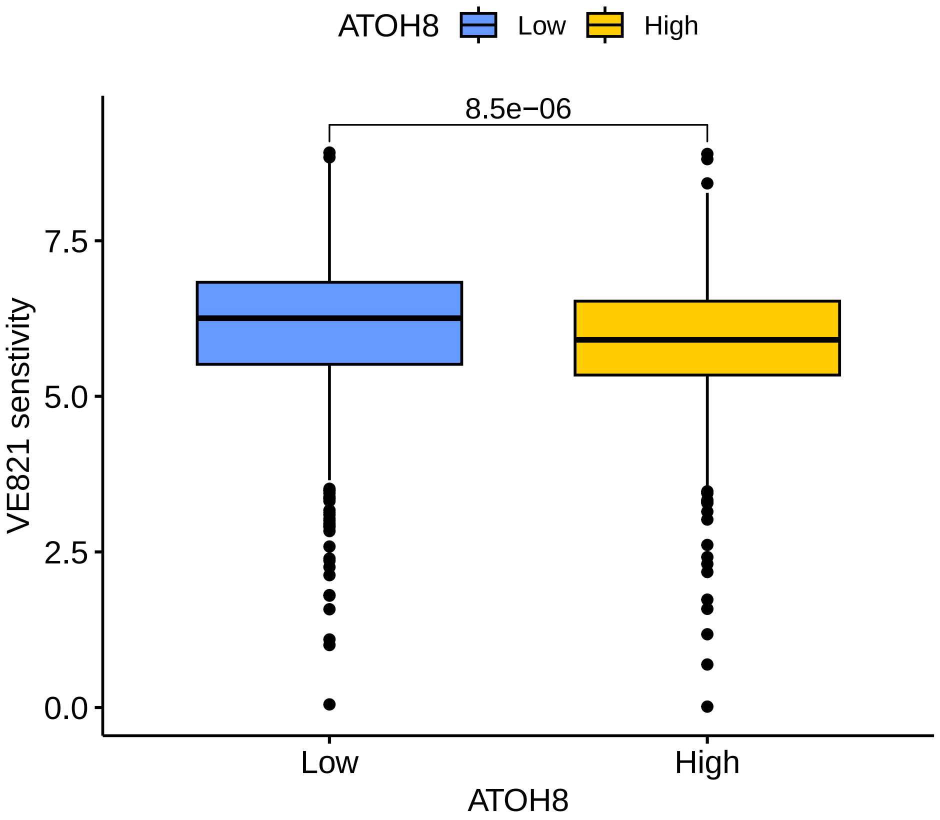

ATOH8
Low
High
8.5e−06
VE821 senstivity
7.5
5.0
2.5
0.0
Low
High
ATOH8

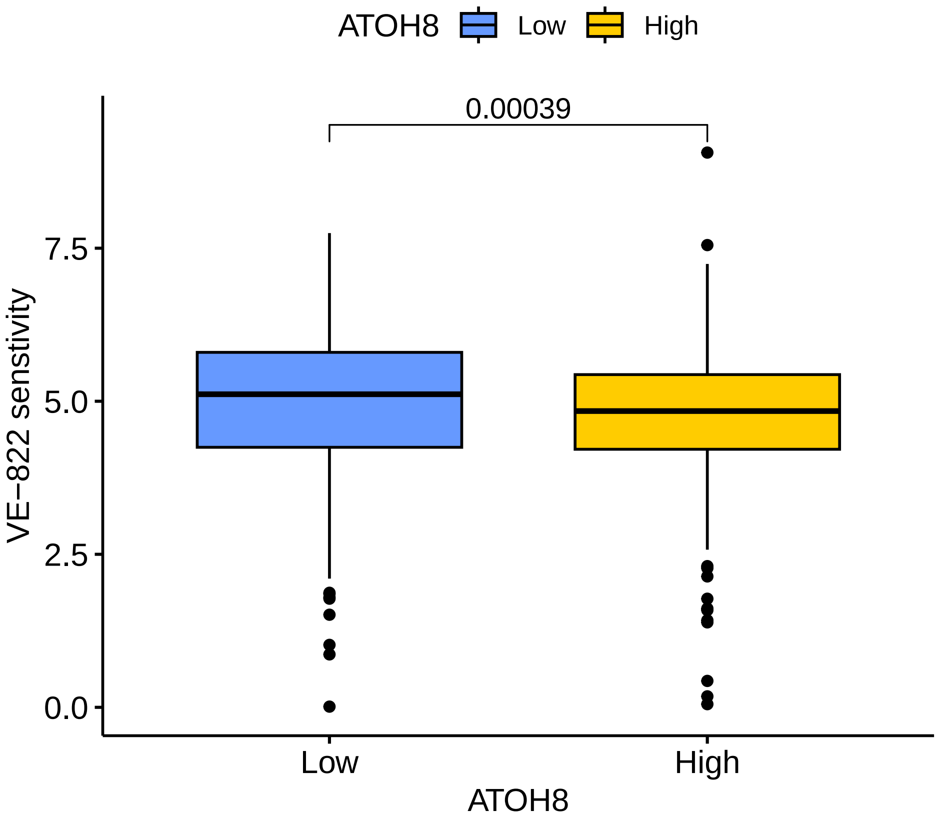

ATOH8
Low
High
0.00039
VE−822 senstivity
7.5
5.0
2.5
0.0
Low
High
ATOH8

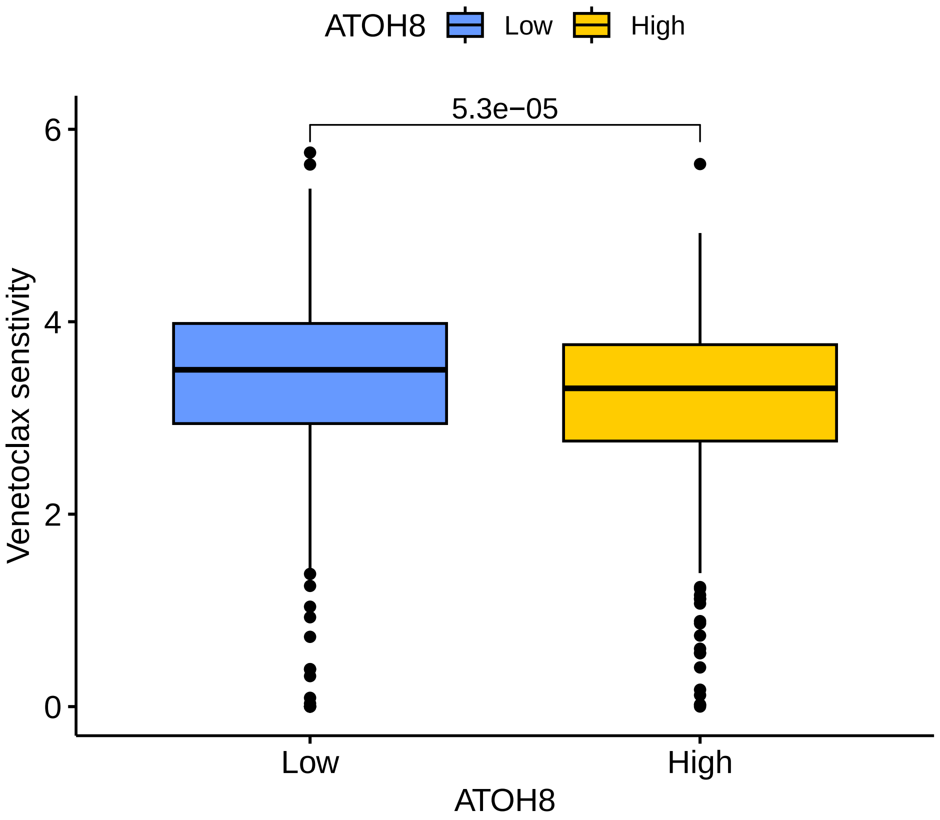
ATOH8
Low
High
5.3e−05
6
4
2
0
Venetoclax senstivity
Low
High
ATOH8

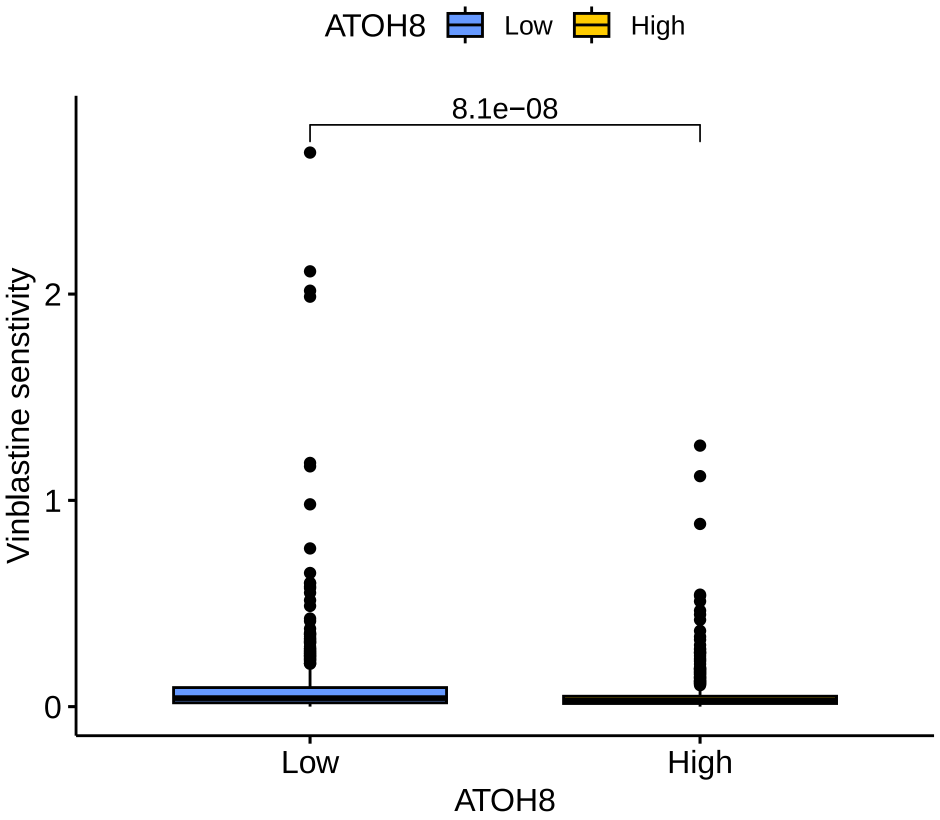
ATOH8
Low
High
8.1e−08
Vinblastine senstivity
2
1
0
Low
High
ATOH8

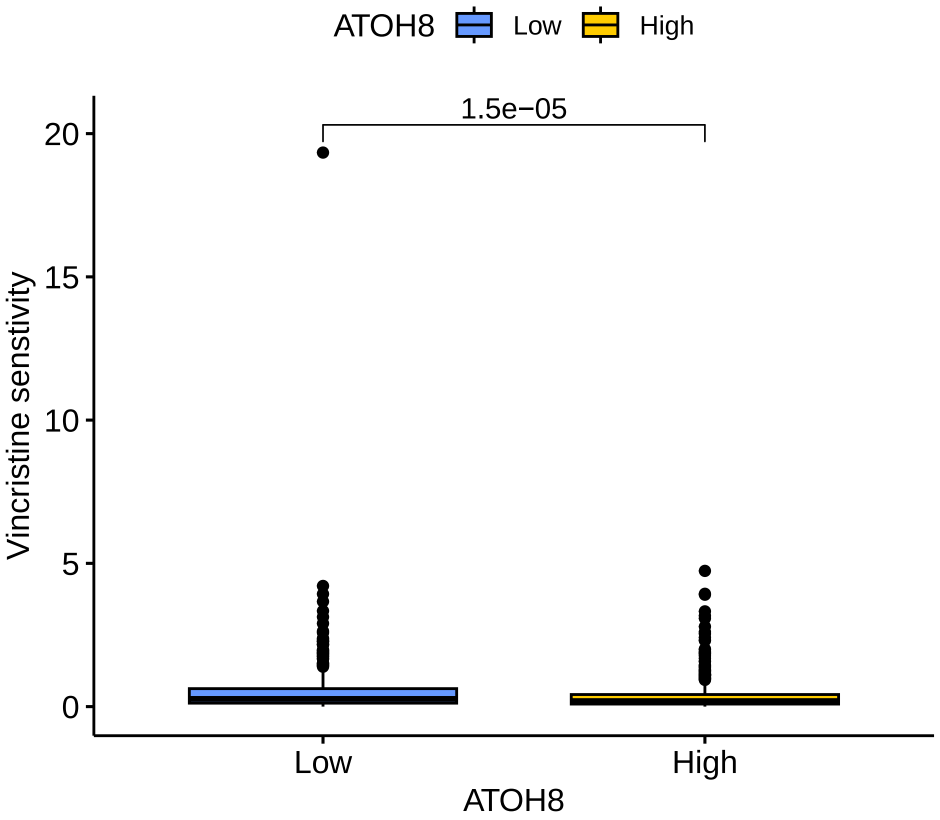

ATOH8
Low
High
1.5e−05
Vincristine senstivity
20
15
10
5
0
Low
High
ATOH8

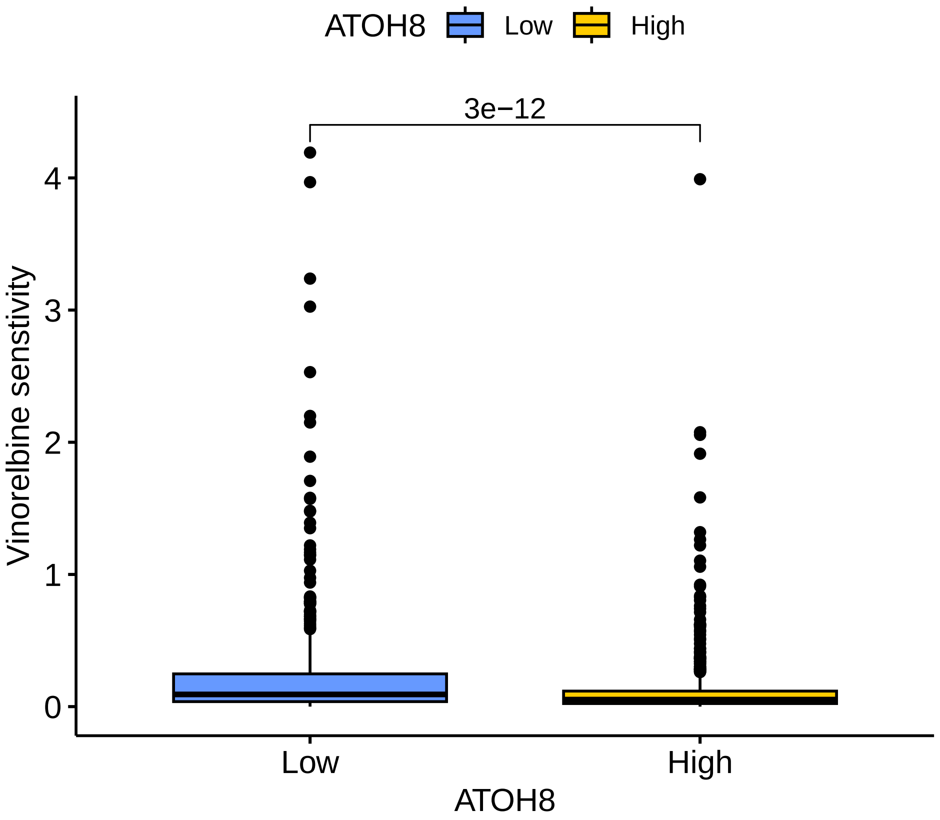

ATOH8
Low
High
3e−12
Vinorelbine senstivity
4
3
2
1
0
Low
High
ATOH8

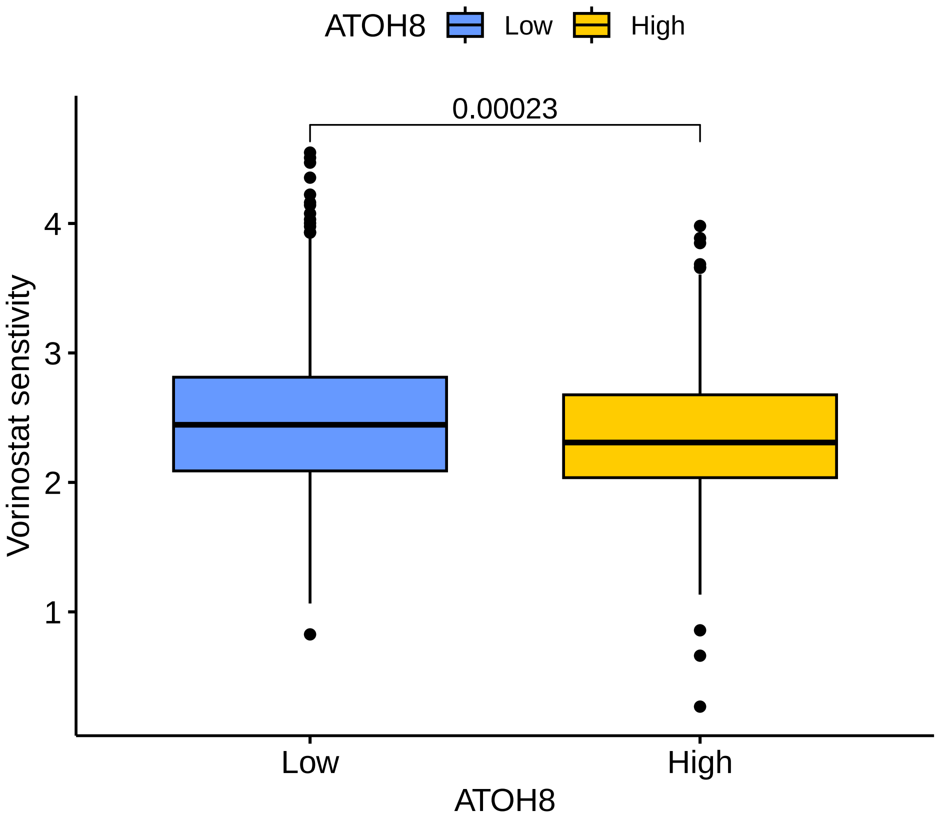
ATOH8
Low
High
0.00023
Vorinostat senstivity
4
3
2
1
Low
High
ATOH8

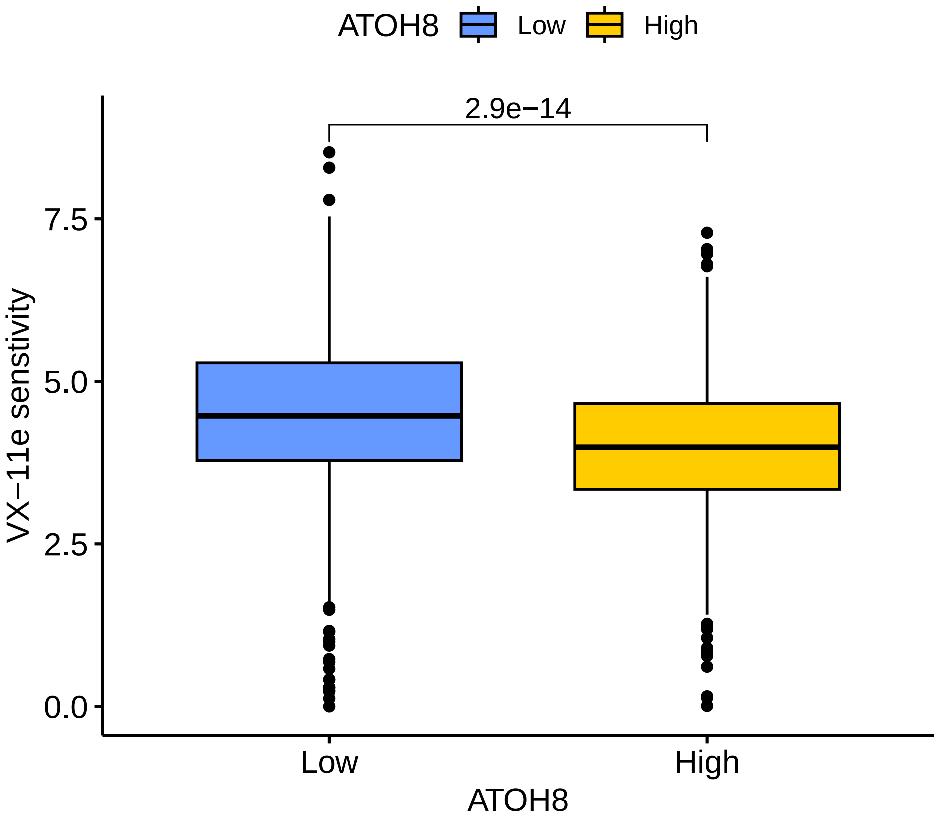

ATOH8
Low
High
2.9e−14
7.5
5.0
2.5
0.0
VX−11e senstivity
Low
High
ATOH8

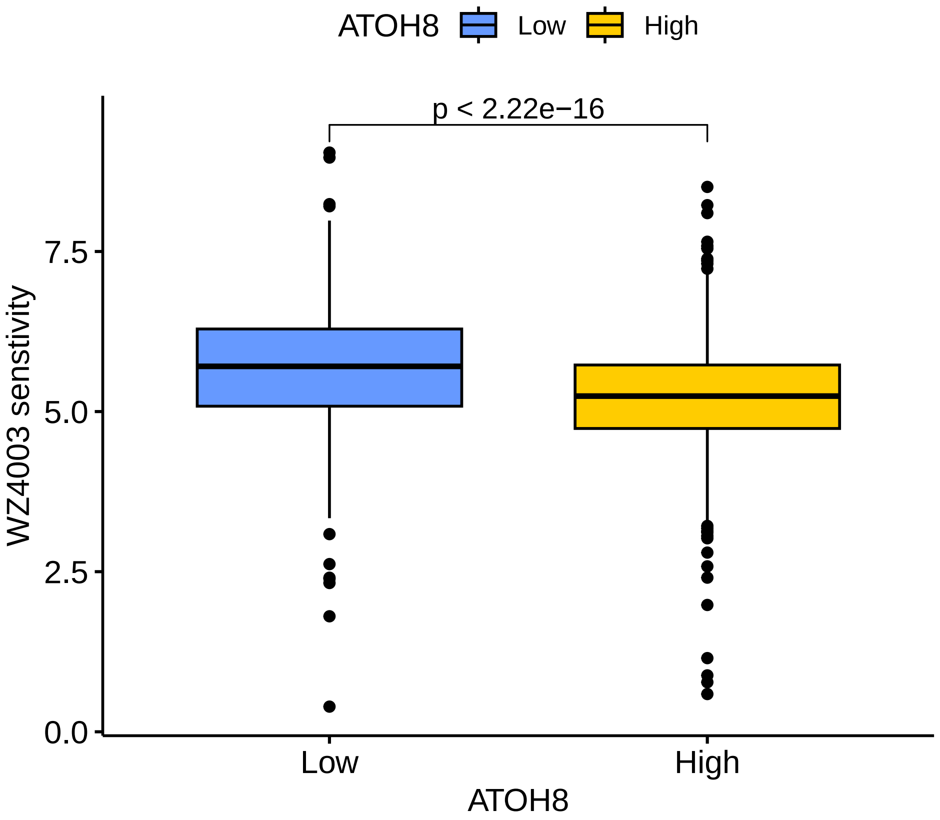

ATOH8
Low
High
p < 2.22e−16
WZ4003 senstivity
7.5
5.0
2.5
0.0
Low
High
ATOH8

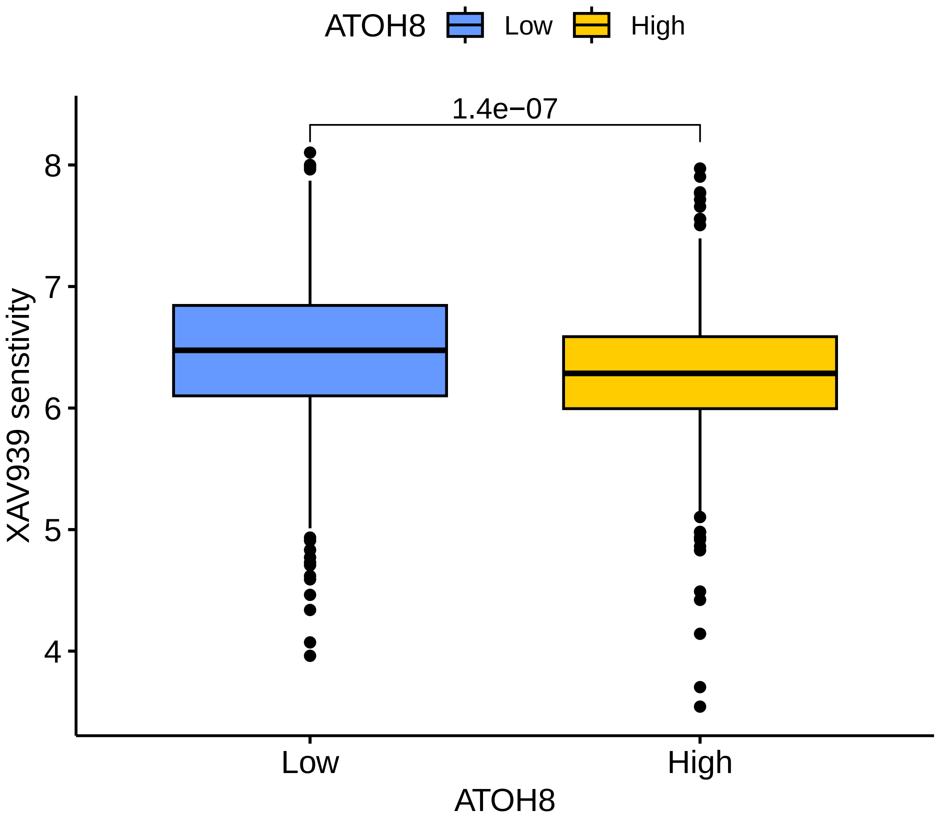

ATOH8
Low
High
1.4e−07
8
7
6
5
4
XAV939 senstivity
Low
High
ATOH8

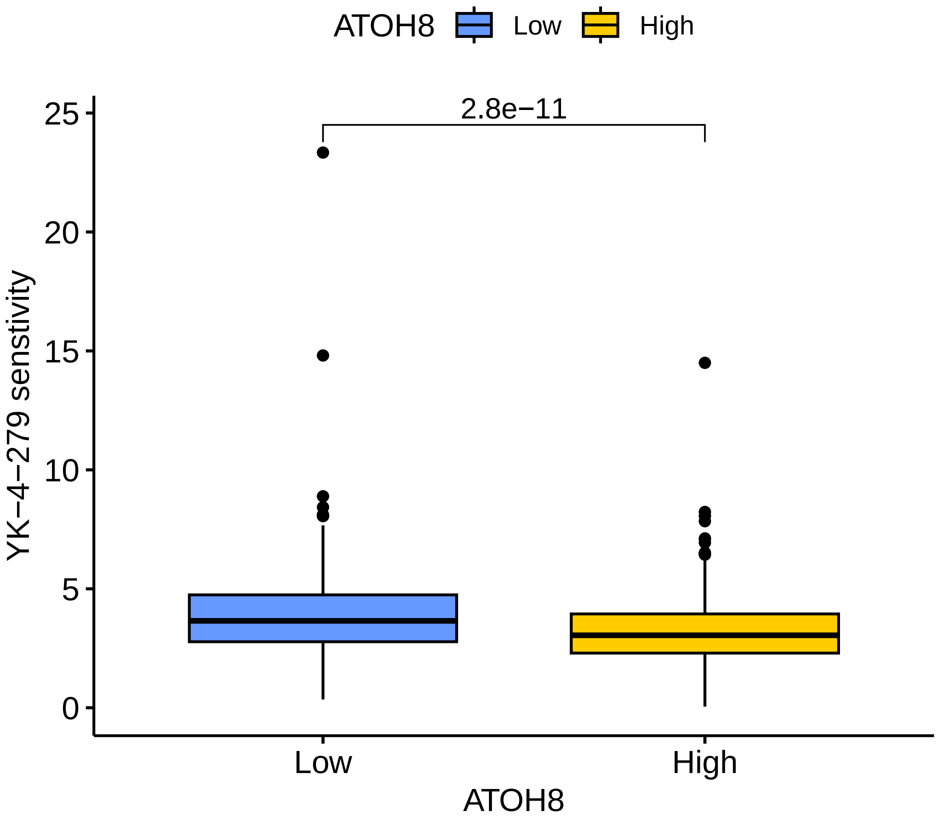
ATOH8
Low
High
2.8e−11
25
20
15
10
5
0
YK−4−279 senstivity
Low
High
ATOH8

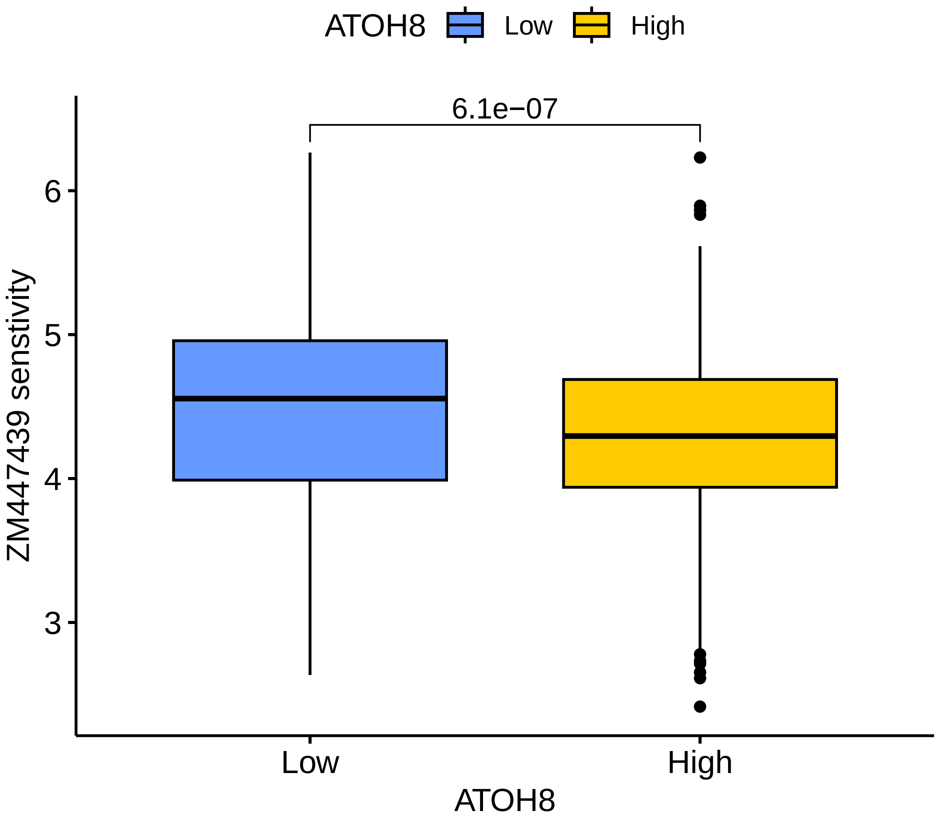
ATOH8
Low
High
6.1e−07
ZM447439 senstivity
6
5
4
3
Low
High
ATOH8

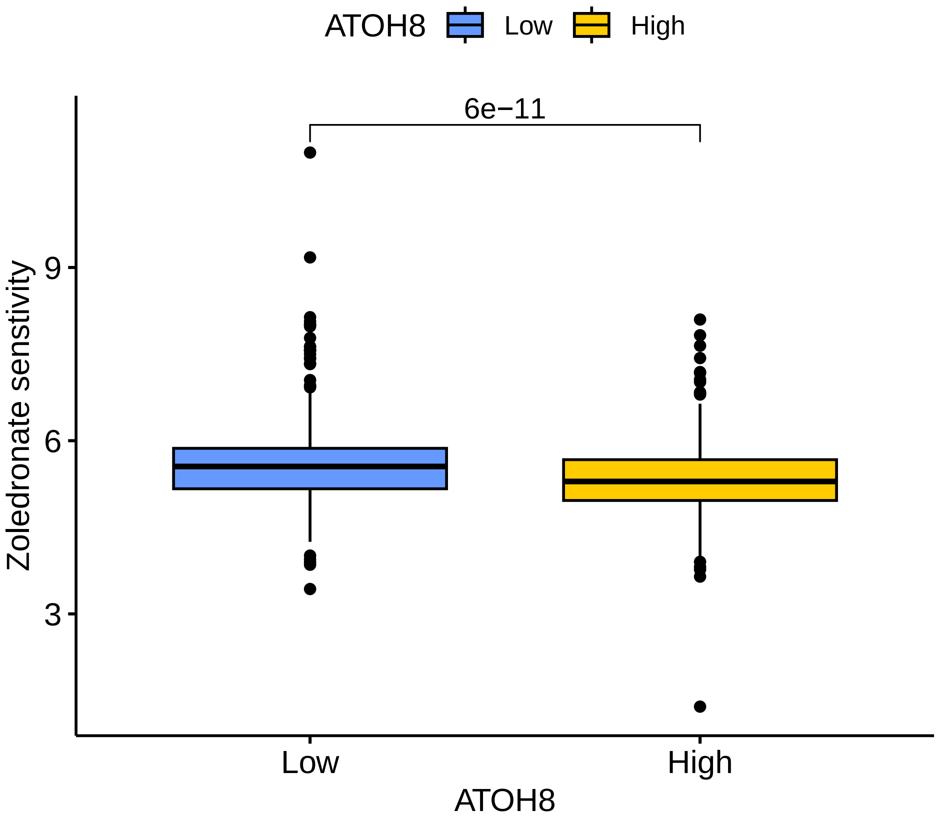

ATOH8
Low
High
6e−11
Zoledronate senstivity
9
6
3
Low
High
ATOH8

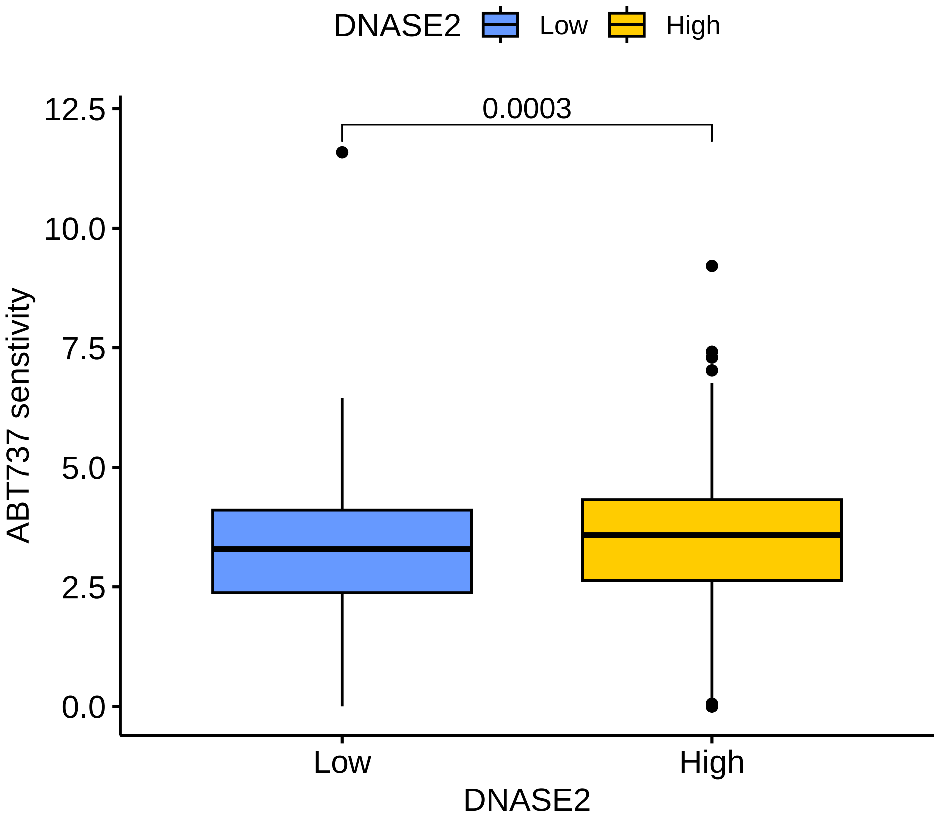
DNASE2
Low
High
0.0003
12.5
10.0
7.5
5.0
2.5
0.0
ABT737 senstivity
Low
High
DNASE2

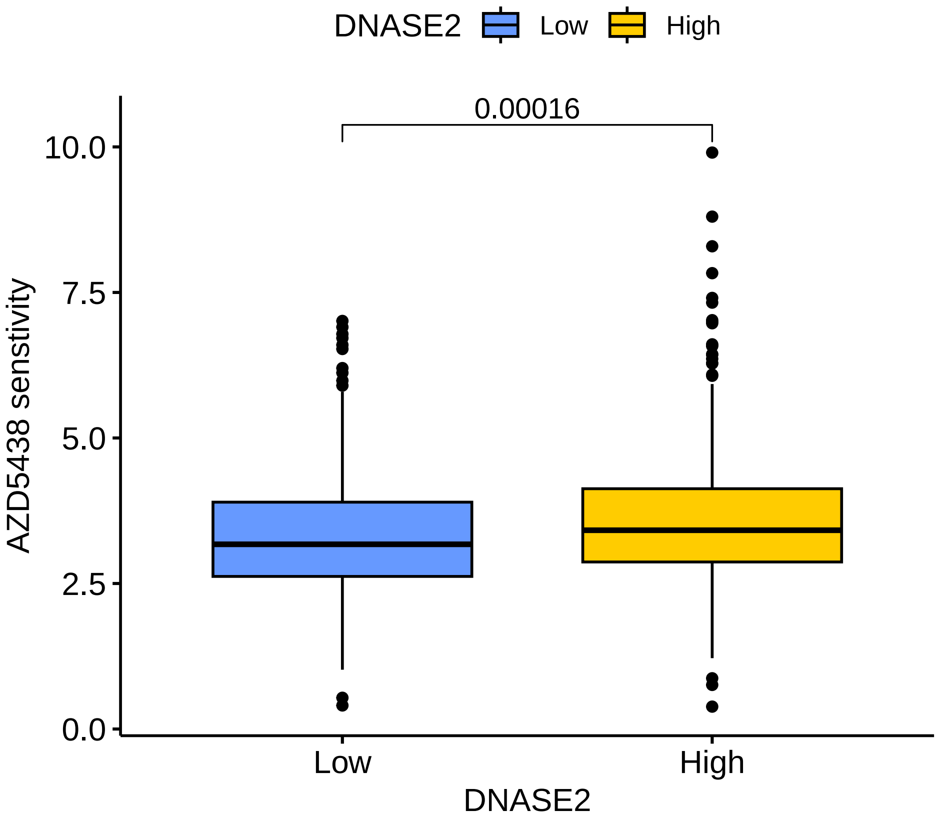

DNASE2
Low
High
0.00016
10.0
7.5
5.0
2.5
0.0
AZD5438 senstivity
Low
High
DNASE2

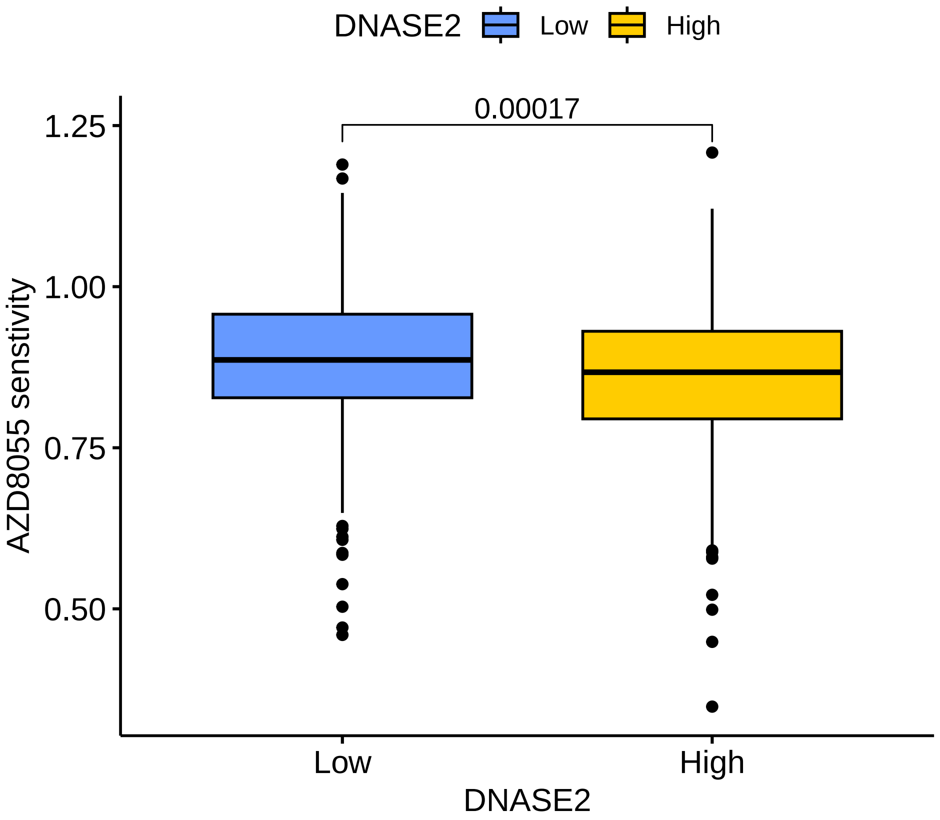
DNASE2
Low
High
0.00017
1.25
1.00
0.75
0.50
AZD8055 senstivity
Low
High
DNASE2

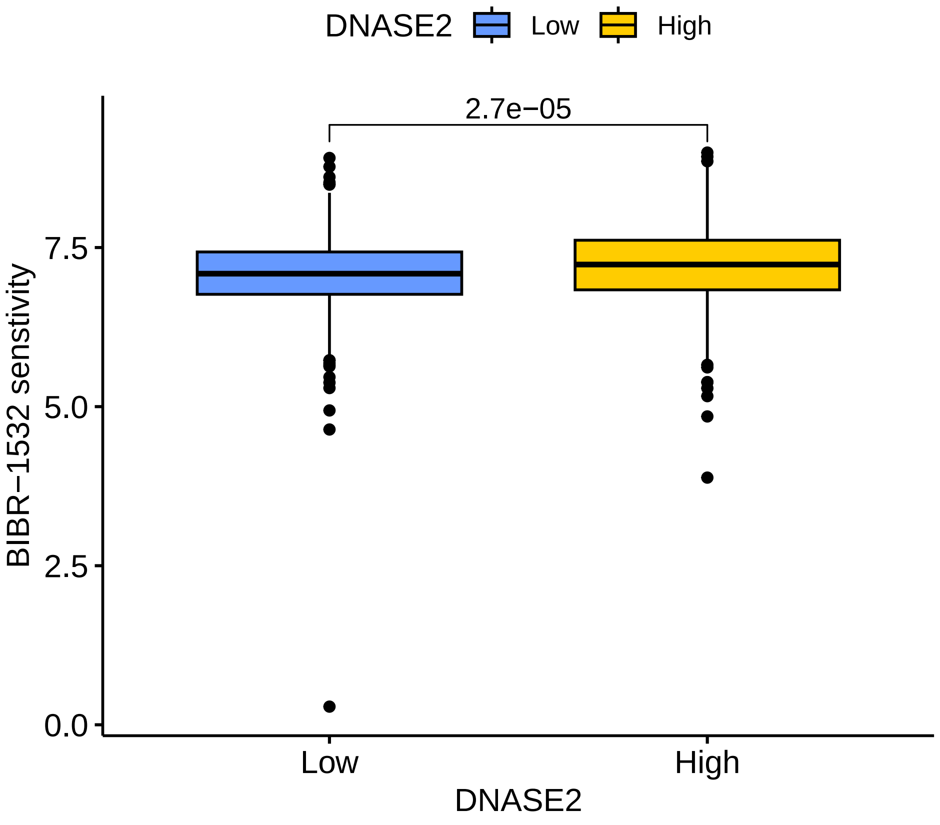

DNASE2
Low
High
2.7e−05
BIBR−1532 senstivity
7.5
5.0
2.5
0.0
Low
High
DNASE2

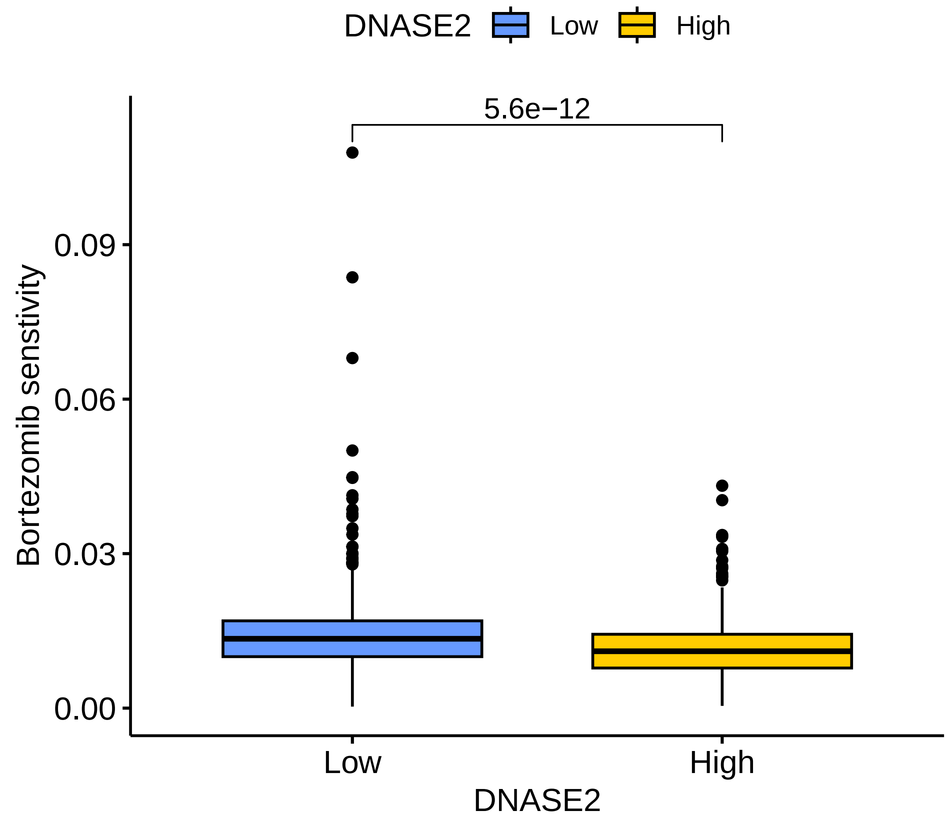
DNASE2
Low
High
5.6e−12
Bortezomib senstivity
0.09
0.06
0.03
0.00
Low
High
DNASE2

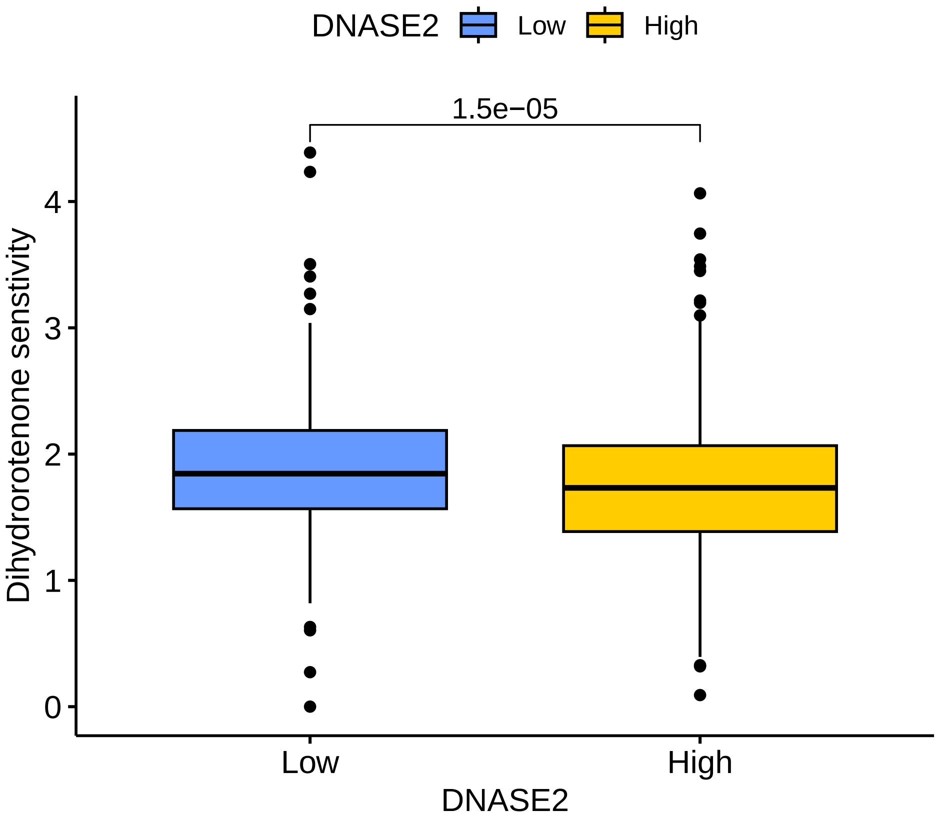

DNASE2
Low
High
1.5e−05
Dihydrorotenone senstivity
0
1
2
3
4
Low
High
DNASE2

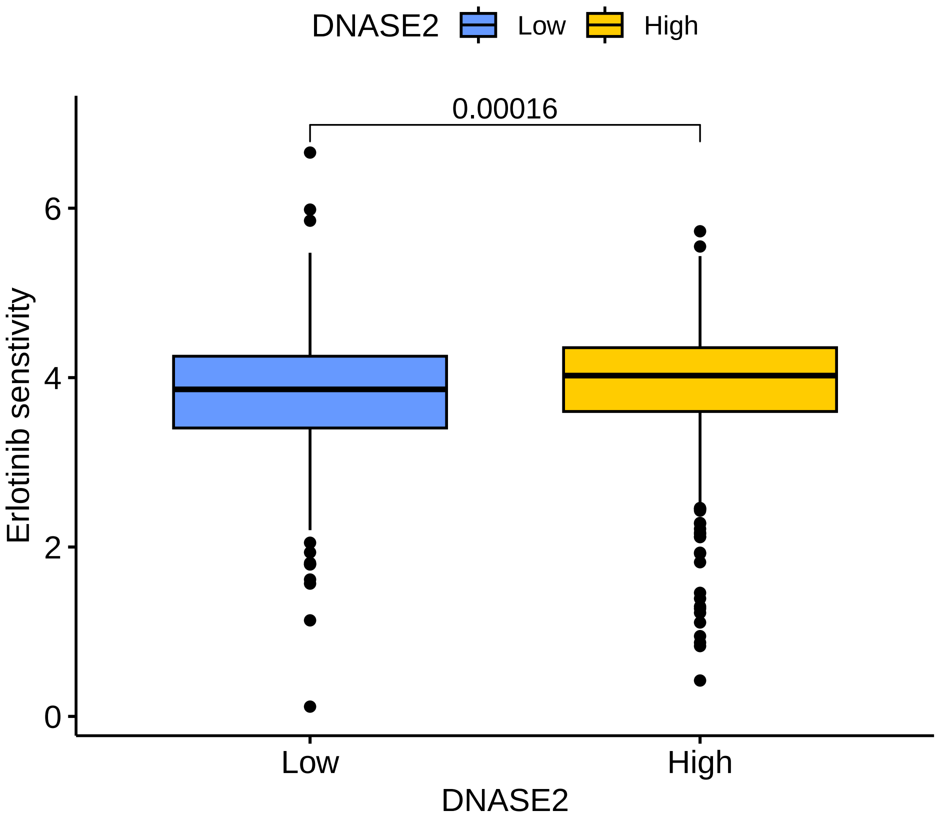
DNASE2
Low
High
0.00016
Erlotinib senstivity
6
4
2
0
Low
High
DNASE2

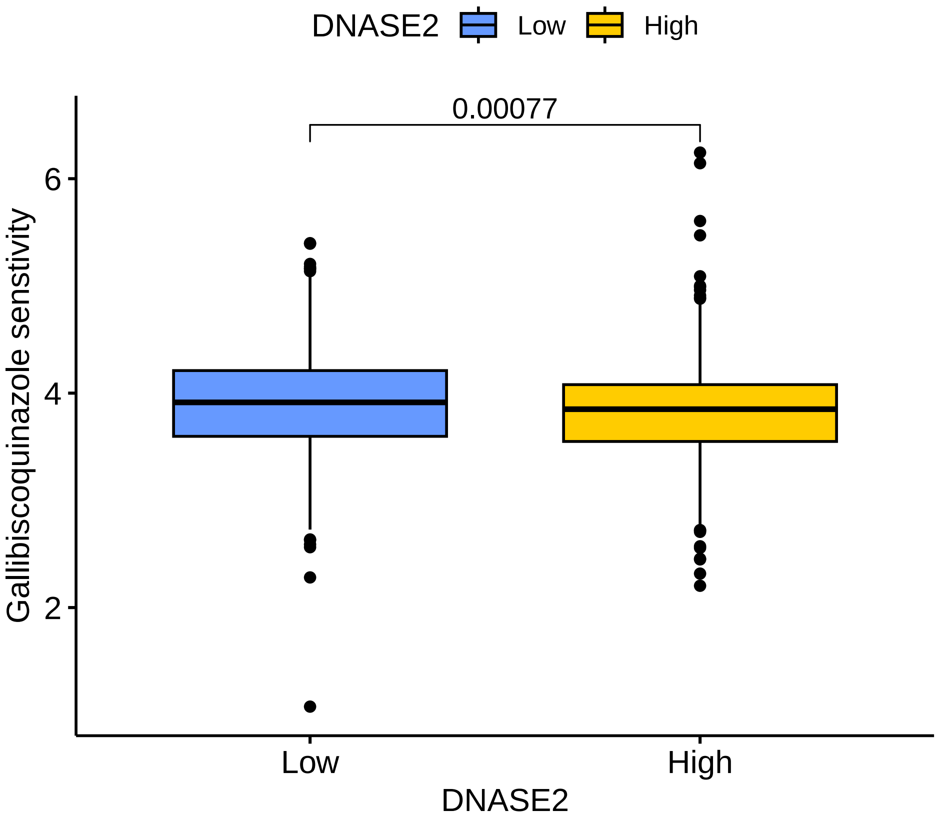
DNASE2
Low
High
0.00077
6
4
2
Gallibiscoquinazole senstivity
Low
High
DNASE2

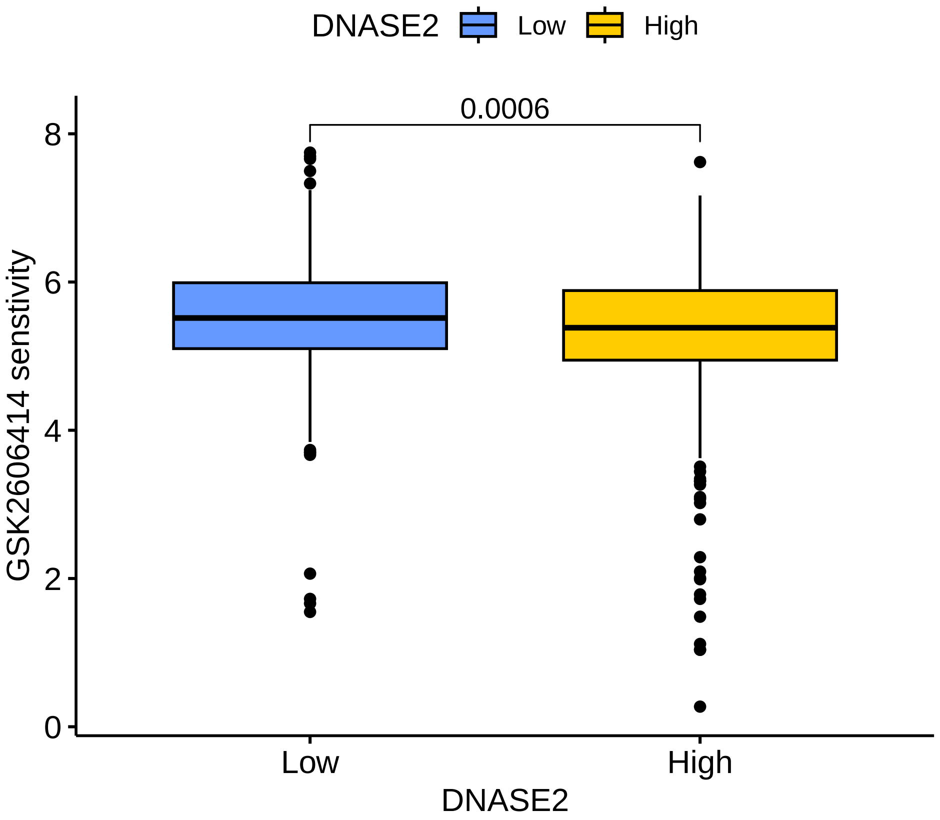

DNASE2
Low
High
0.0006
GSK2606414 senstivity
8
6
4
2
0
Low
High
DNASE2

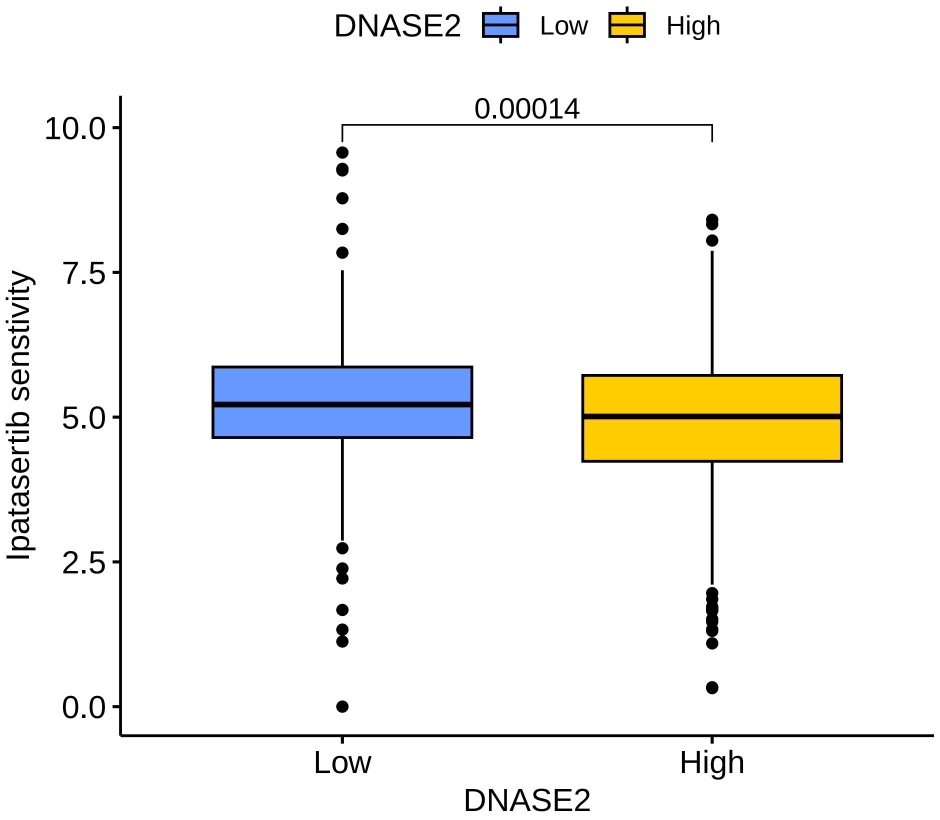

DNASE2
Low
High
0.00014
10.0
7.5
5.0
2.5
0.0
Ipatasertib senstivity
Low
High
DNASE2

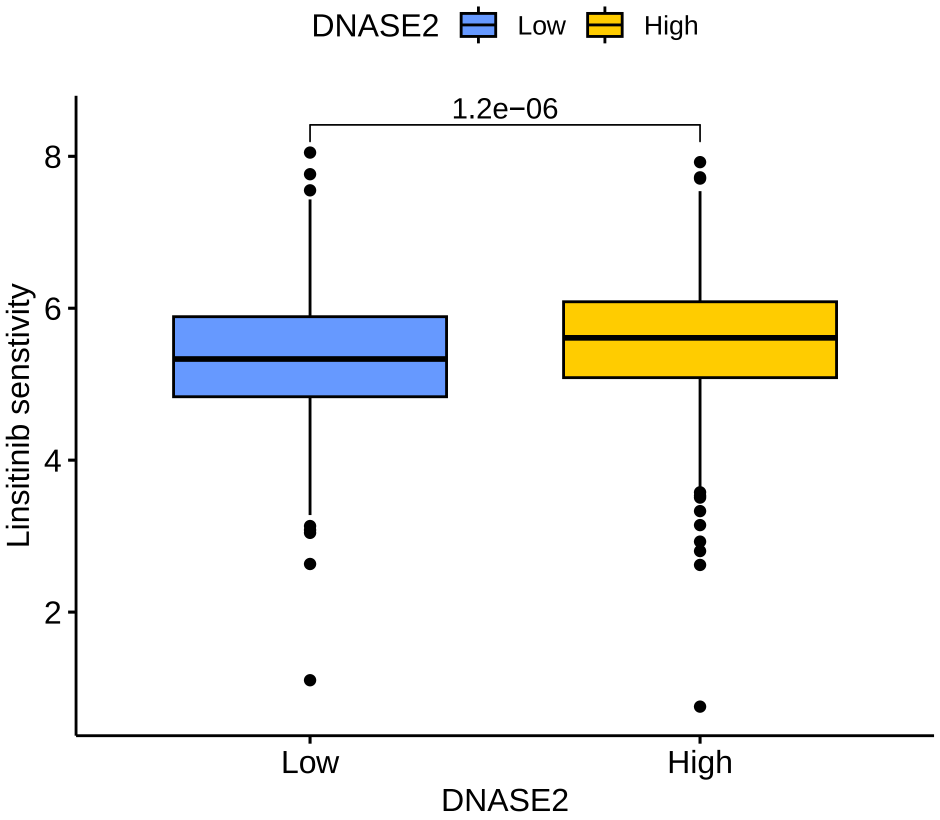
DNASE2
Low
High
1.2e−06
Linsitinib senstivity
8
6
4
2
Low
High
DNASE2

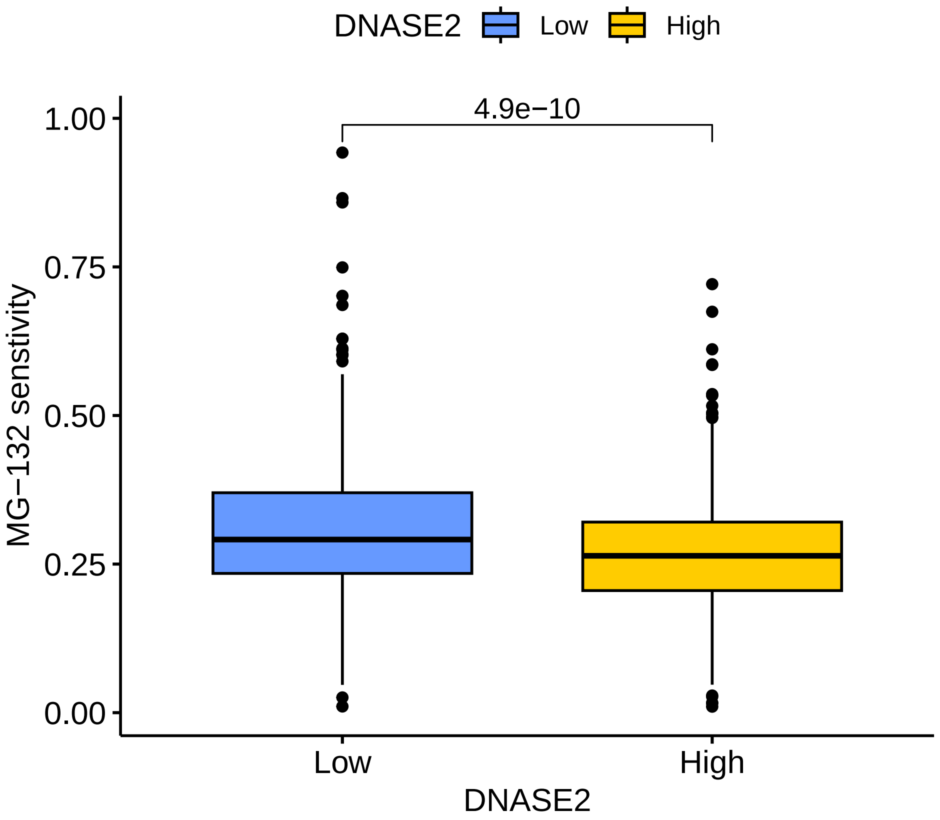

DNASE2
Low
High
4.9e−10
1.00
0.75
0.50
0.25
0.00
MG−132 senstivity
Low
High
DNASE2

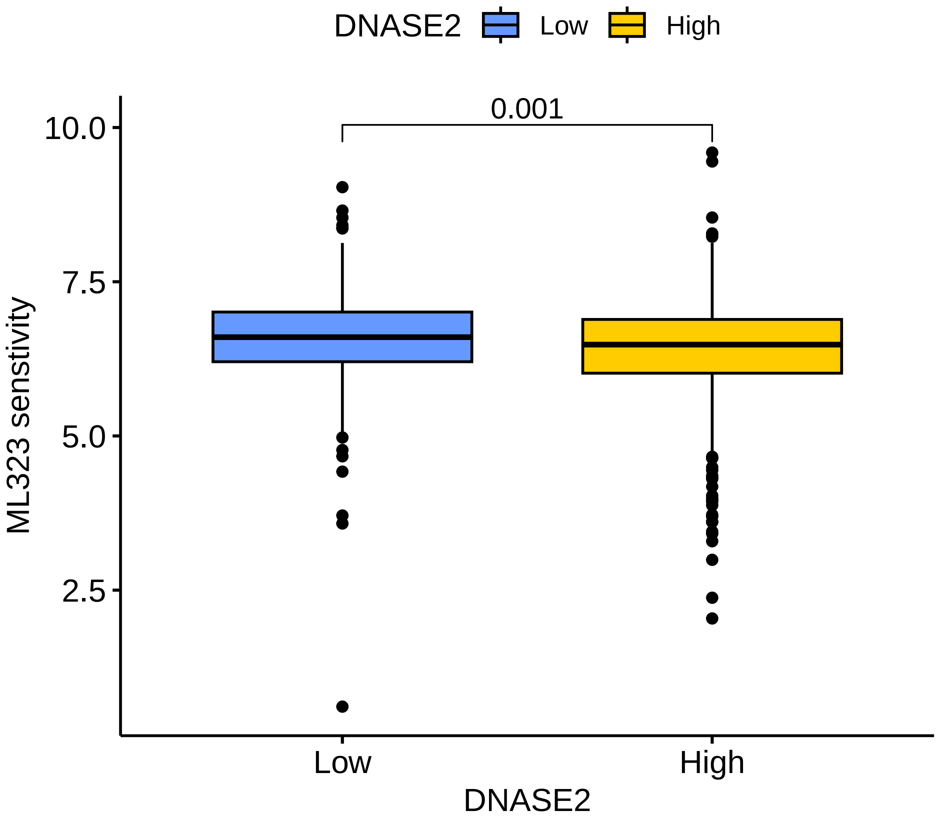

DNASE2
Low
High
0.001
10.0
7.5
5.0
2.5
ML323 senstivity
Low
High
DNASE2

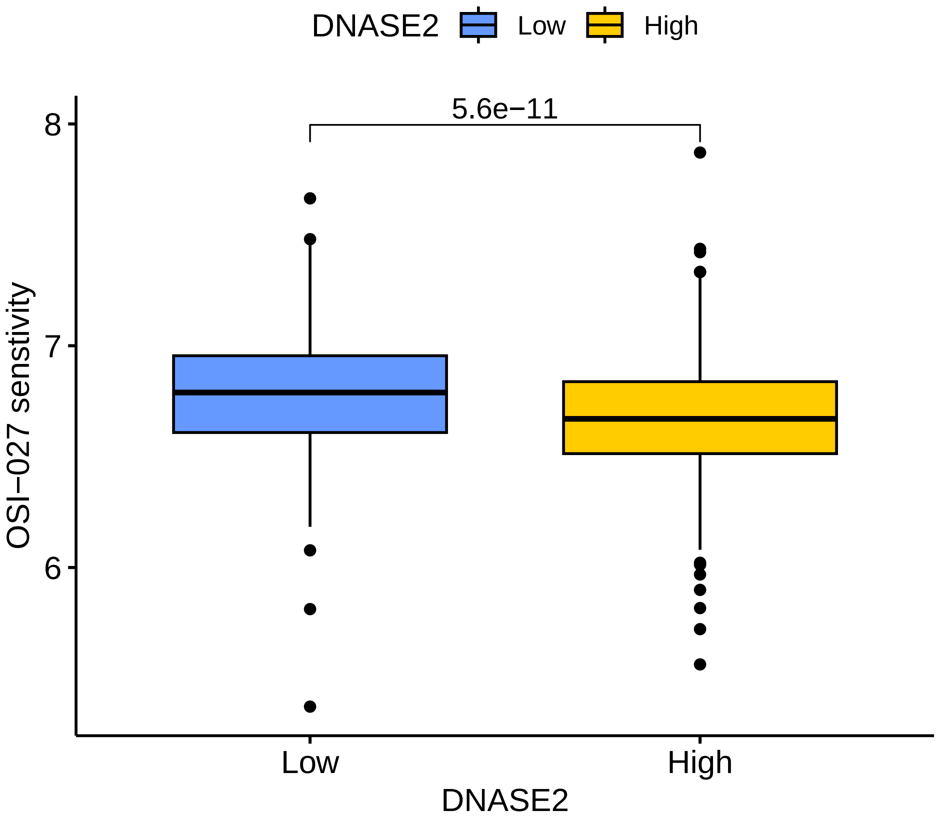

DNASE2
Low
High
5.6e−11
8
7
6
OSI−027 senstivity
Low
High
DNASE2

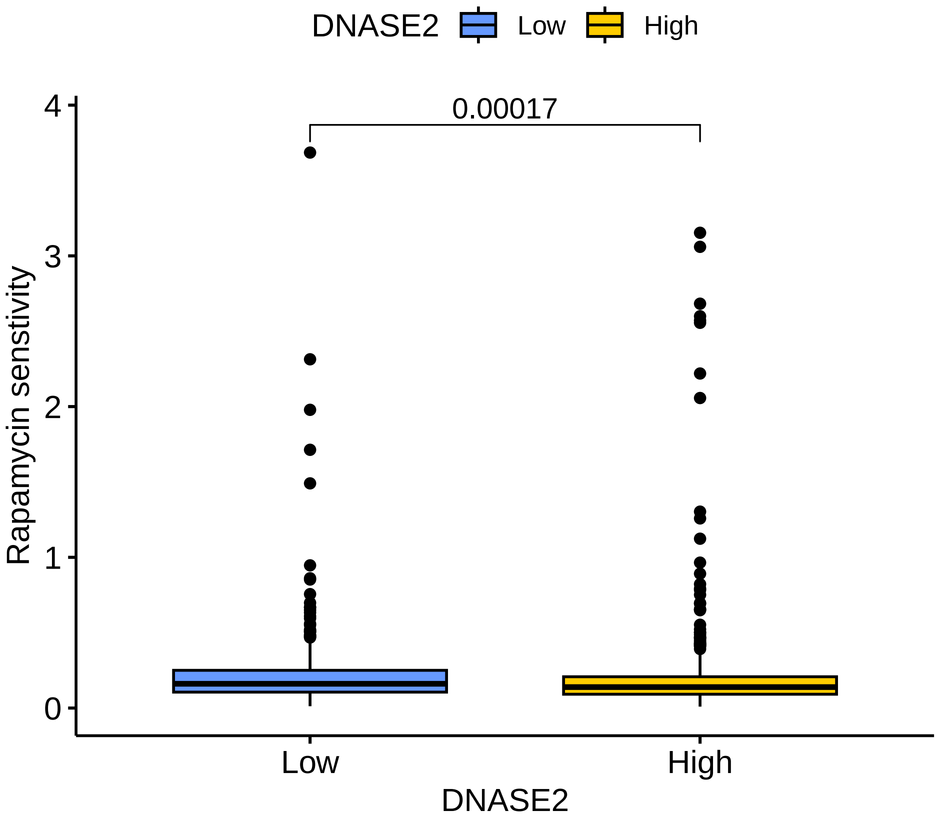
DNASE2
Low
High
0.00017
Rapamycin senstivity
0
1
2
3
4
Low
High
DNASE2

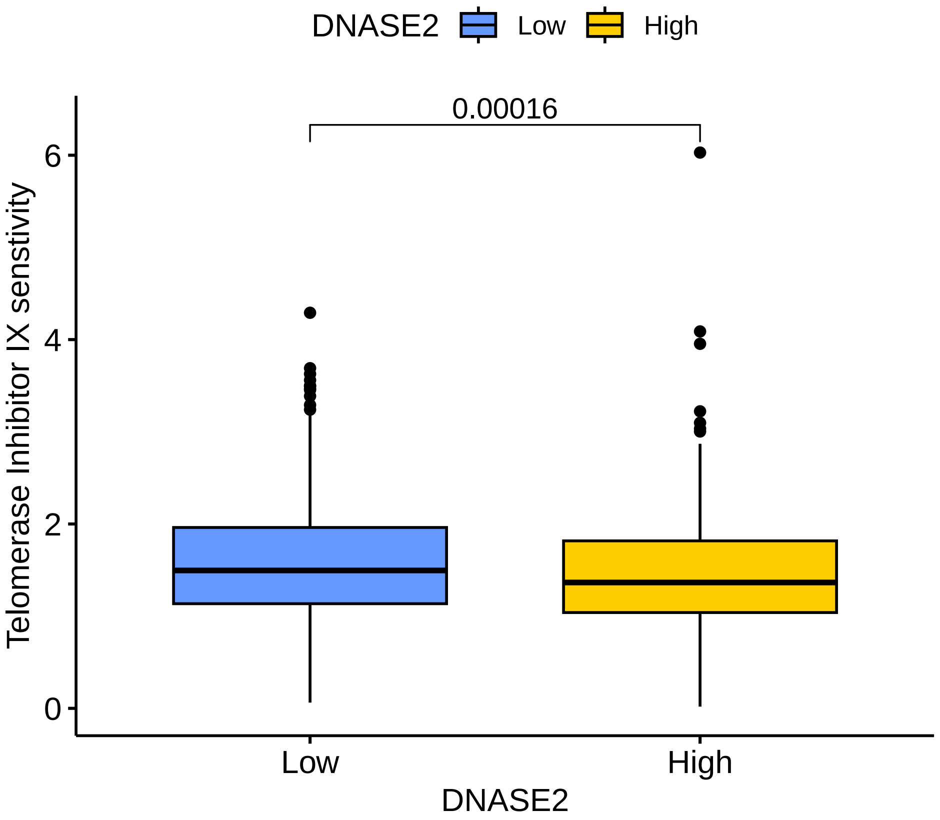
DNASE2
Low
High
0.00016
Telomerase Inhibitor IX senstivity
6
4
2
0
Low
High
DNASE2

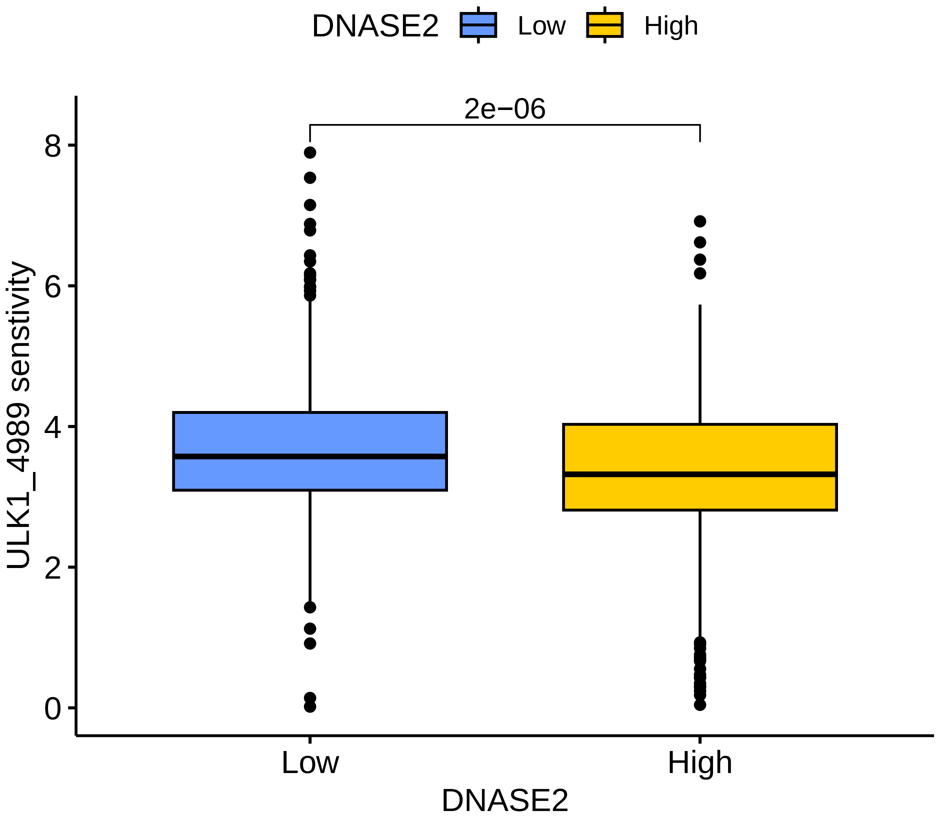
DNASE2
Low
High
2e−06
8
6
4
2
0
ULK1_4989 senstivity
Low
High
DNASE2

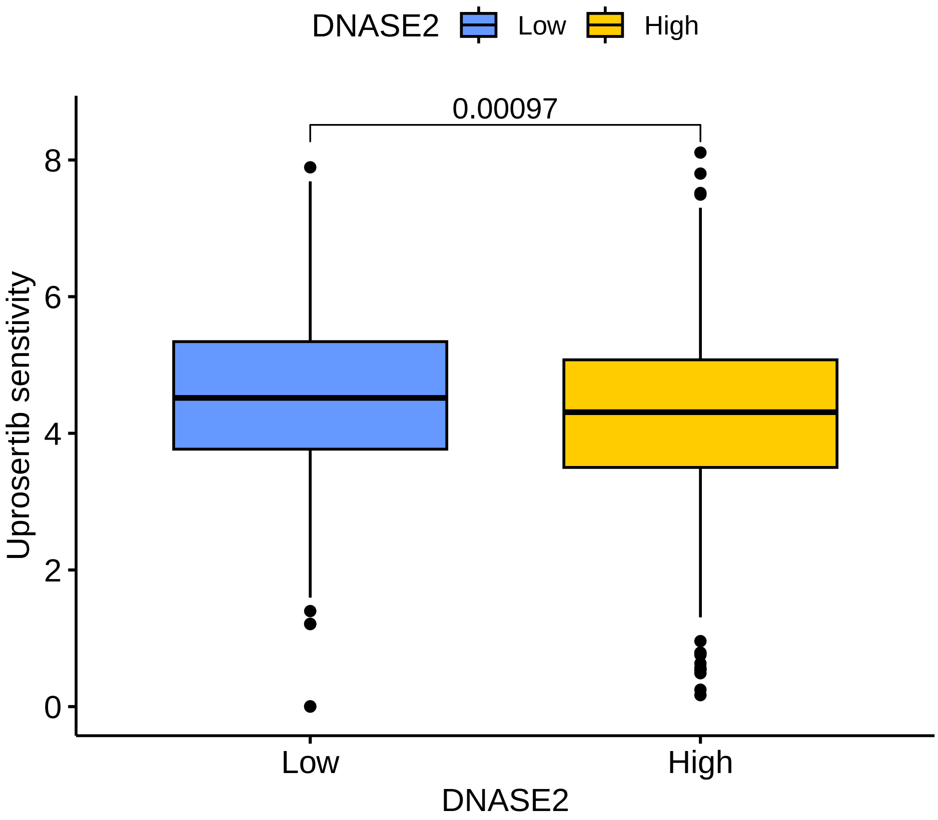

DNASE2
Low
High
0.00097
8
6
4
2
0
Uprosertib senstivity
Low
High
DNASE2

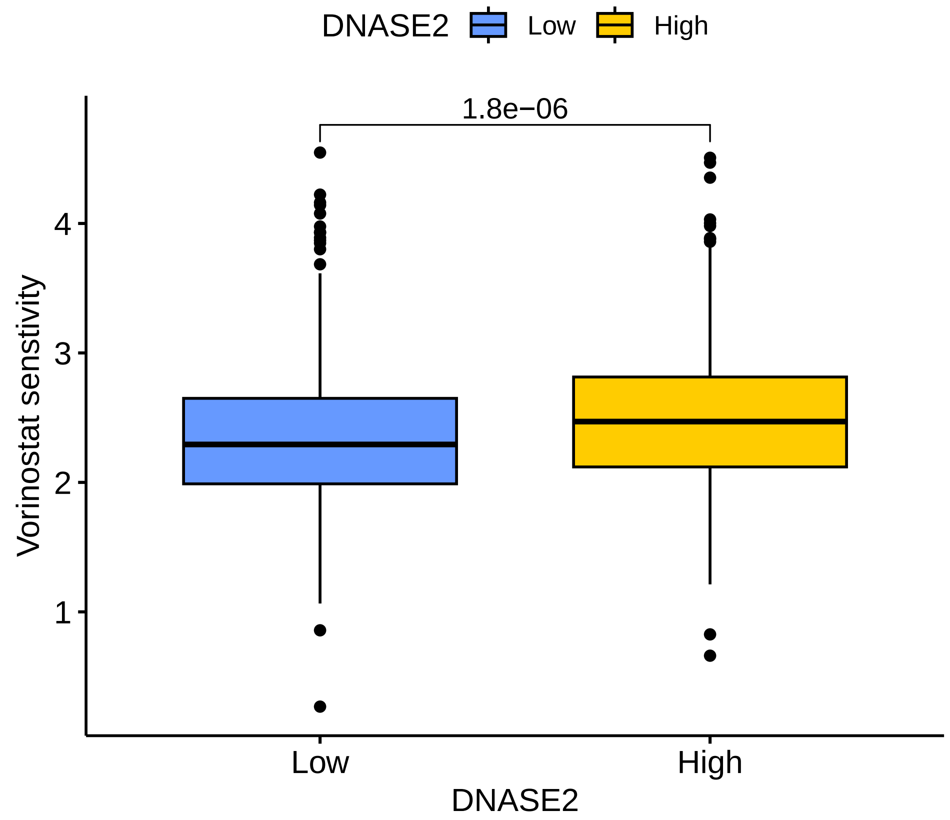

DNASE2
Low
High
1.8e−06
Vorinostat senstivity
4
3
2
1
Low
High
DNASE2

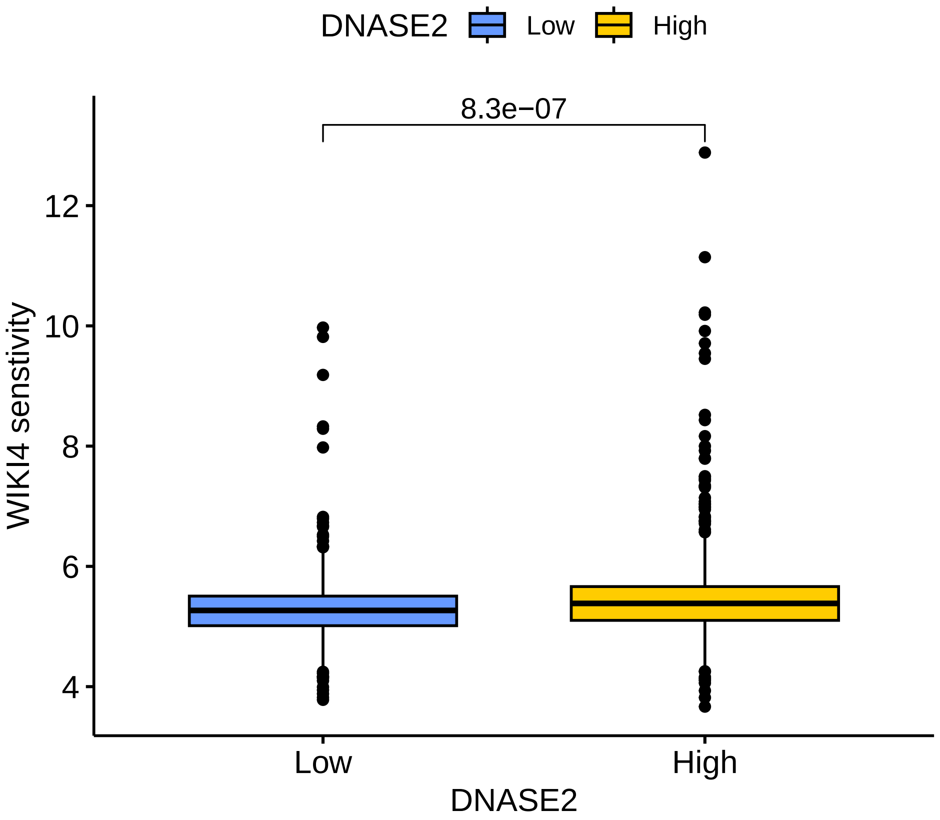

DNASE2
Low
High
8.3e−07
12
10
8
6
4
WIKI4 senstivity
Low
High
DNASE2
